# Supplementary material for: Cryo-EM structures of Trypanosoma brucei gambiense ISG65 with human complement C3 and C3b and their roles in alternative pathway restriction
Source: Nat Commun. 2023 Apr 27;14:2403. doi: 10.1038/s41467-023-37988-7 (PMC10140031; doi:10.1038/s41467-023-37988-7)
Supplement: Supplementary file 1 — Supplementary Information [file 41467_2023_37988_MOESM1_ESM.pdf]

Cryo-EM structures of *Trypanosoma brucei gambiense* ISG65 with human complement C3 and C3b and their roles in alternative pathway restriction

Supplementary Information

Supplementary Figures and Tables

a

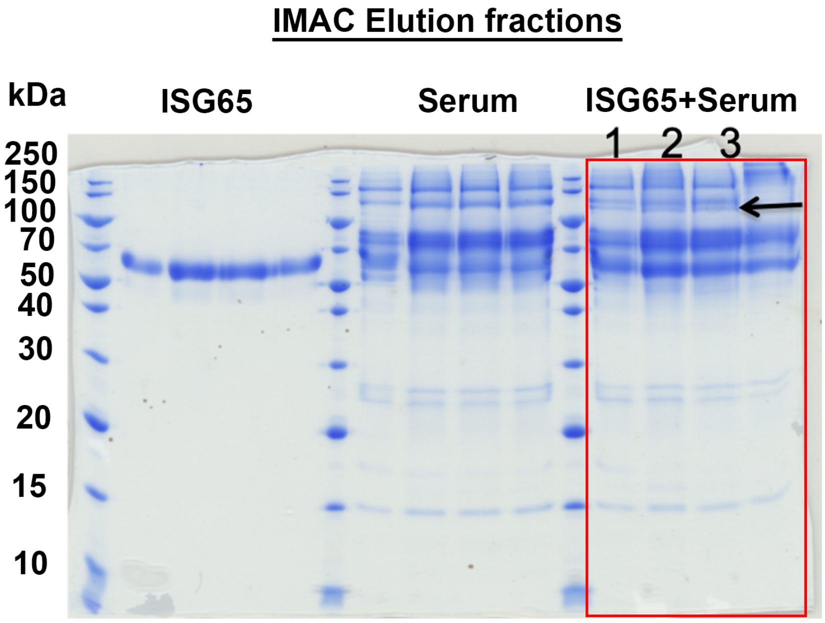

b

Band 1

Checked	Master	Accession	Description	Coverage [%]	Contaminant	# Peptides	# PSMs	# Unique Peptides	# AAs	MW [kDa]	calc. pI
FALSE	Master Protein	P01024	Complement C3 [OS=Homo sapiens]	5	FALSE	8	17	8	1663	187	6.4
	Annotated Sequence	Modifications	Positions in Master Proteins	Theo. MH+ [Da]							
	[R].CAEENCFIQK.[S]	2xCarbamidomethyl [C1; C6]	P01024 [1513-1522]	1298.55038							
	[K].DICEEQVNSLPGSITK.[A]	1xCarbamidomethyl [C3]	P01024 [1156-1171]	1789.86365							
	[K].ENEGFTVTAEGK.[G]		P01024 [1326-1337]	1281.59574							
	[R].HQQTVTIPPK.[S]		P01024 [882-891]	1148.64223							
	[R].LDKACEPGVDYVYK.[T]	1xCarbamidomethyl [C5]	P01024 [1533-1546]	1656.79378							
	[R].LDKACEPGVDYVYKTR.[L]	1xCarbamidomethyl [C5]	P01024 [1533-1548]	1913.94257							
	[R].QGALELIK.[K]		P01024 [1043-1050]	871.52474							
	[K].SGSDEVQVGQQR.[T]		P01024 [1571-1582]	1289.60803							
FALSE	Master Protein	P04264	Keratin, type II cytoskeletal 1 [OS=Homo sapiens]	10	TRUE	5	8	4	644	66	8.12
FALSE	Master Protein	P13645	Keratin, type I cytoskeletal 10 [OS=Homo sapiens]	7	TRUE	3	5	3	584	58.8	5.21
FALSE	Master Protein	P01023	alpha-2-macroglobulin [OS=Homo sapiens]	3	FALSE	4	7	4	1474	163.2	6.46
FALSE	Master Protein	P01042	kininogen-1 [OS=Homo sapiens]	2	FALSE	1	4	1	644	71.9	6.81
FALSE	Master Protein	P35908	Keratin, type II cytoskeletal 2 epidermal [OS=Homo sapiens]	4	TRUE	2	4	1	639	65.4	8
FALSE	Master Protein	P00751-1	Complement factor B [OS=Homo sapiens]	4	FALSE	3	5	3	764	85.5	7.06
FALSE	Master Protein	P02768-1	Serum albumin [OS=Homo sapiens]	4	TRUE	2	3	2	609	69.3	6.28
FALSE	Master Protein	P0DOY2	immunoglobulin lambda constant 2 [OS=Homo sapiens]	9	FALSE	1	2	1	106	11.3	7.24
FALSE	Master Protein	P35527	Keratin, type I cytoskeletal 9 [OS=Homo sapiens]	7	TRUE	2	3	2	623	62	5.24
FALSE	Master Protein	P00760	Cationic trypsin OS=Bos taurus PE=1 SV=3	14	TRUE	3	3	3	246	25.8	8.07
FALSE	Master Protein	Q61726	Keratin type II (Fragment) OS=Mus musculus GN=5430421N21Rik PE=2 SV=1	3	TRUE	1	1	1	495	54.9	6.46
FALSE	Master Protein	Q9UPV9	Trafficking kinesin-binding protein 1 [OS=Homo sapiens]	3	FALSE	1	1	1	953	106	5.85
FALSE	Master Protein	Q2M3C7	A-kinase anchor protein SPHKAP [OS=Homo sapiens]	2	FALSE	1	1	1	1700	186.3	5.14
FALSE	Master Protein	P21675	transcription initiation factor TFIID subunit 1 [OS=Homo sapiens]	2	FALSE	1	1	1	1872	212.5	5.07
FALSE	Master Protein	O95219	sorting nexin-4 [OS=Homo sapiens]	3	FALSE	1	1	1	450	51.9	5.99

Band 2

Checked	Master	Accession	Description	Coverage [%]	Contaminant	# Peptides	# PSMs	# Unique Peptides	# AAs	MW [kDa]	calc. pI
FALSE	Master Protein	P01024	Complement C3 [OS=Homo sapiens]	18	FALSE	27	91	27	1663	187	6.4
	Annotated Sequence	Modifications	Positions in Master Proteins	Theo. MH+ [Da]							
	[R].CAEENCFIQK.[S]	2xCarbamidomethyl [C1; C6]	P01024 [1513-1522]	1298.55038							
	[K].DICEEQVNSLPGSITK.[A]	1xCarbamidomethyl [C3]	P01024 [1156-1171]	1789.86365							
	[K].DSCVGSLVVK.[S]	1xCarbamidomethyl [C3]	P01024 [557-566]	1063.54522							
	[R].EGVQKEDIPPADLSDQVPDTESETR.[I]		P01024 [955-979]	2755.29045							
	[R].FISLGEACK.[K]	1xCarbamidomethyl [C8]	P01024 [713-721]	1024.51319							
	[R].FISLGEACKK.[V]	1xCarbamidomethyl [C8]	P01024 [713-722]	1152.60816							
	[K].FLTTAKDKNR.[W]		P01024 [1210-1219]	1193.6637							
	[K].GYTQQLAFR.[Q]		P01024 [1052-1060]	1083.55817							
	[R].HQQTVTIPPK.[S]		P01024 [882-891]	1148.64223							
	[R].LDKACEPGVDYVYK.[T]	1xCarbamidomethyl [C5]	P01024 [1533-1546]	1656.79378							
	[R].IPIEDGSGEVVLSR.[K]		P01024 [291-304]	1470.77985							
	[R].IPIEDGSGEVVLSRK.[V]		P01024 [291-305]	1598.87481							
	[K].NTMILEICTR.[Y]	1xCarbamidomethyl [C8]	P01024 [1382-1391]	1250.62315							
	[R].QGALELIK.[K]		P01024 [1043-1050]	871.52474							
	[K].QLANGVDR.[Y]		P01024 [1420-1427]	872.45846							
	[R].RHQQTVTIPPK.[S]		P01024 [881-891]	1304.74334							
	[K].RIPIEDGSGEVVLSR.[K]		P01024 [290-304]	1626.88096							
	[K].SDDKVTLEER.[L]		P01024 [1523-1532]	1191.58517							
	[R].SEETKENEGFTVTAEGK.[G]		P01024 [1321-1337]	1855.85559							
	[K].SGSDEVQVGQQR.[T]		P01024 [1571-1582]	1289.60803							
	[K].SLKVVPEGIR.[M]		P01024 [928-937]	1097.66772							
	[R].SNLDEDIIAEENIVSR.[S]		P01024 [749-764]	1816.89231							
	[K].SSLSVPYVIVPLK.[T]		P01024 [892-904]	1401.83518							
	[R].TFISPIKCR.[E]	1xCarbamidomethyl [C8]	P01024 [1583-1591]	1121.61358							
	[R].TKKQELSEAEQATR.[T]		P01024 [426-439]	1618.83949							
	[R].VPVAVQGEDTVQSLTQGDGVAK.[L]		P01024 [387-408]	2198.12992							
	[K].VTIKPAPETEKRPQDAK.[N]		P01024 [1365-1381]	1908.0549							
FALSE	Master Protein	P02787	Serotransferrin [OS=Homo sapiens]	11	TRUE	7	13	7	698	77	7.12
FALSE	Master Protein	P01023	alpha-2-macroglobulin [OS=Homo sap	4	FALSE	5	11	5	1474	163.2	6.46
FALSE	Master Protein	P04264	Keratin, type II cytoskeletal 1 [OS=Hom	11	TRUE	6	8	5	644	66	8.12
FALSE	Master Protein	P00760	Cationic trypsin OS=Bos taurus PE=1 S\	25	TRUE	5	9	5	246	25.8	8.07
FALSE	Master Protein	P01042	kininogen-1 [OS=Homo sapiens]	4	FALSE	2	6	2	644	71.9	6.81
FALSE	Master Protein	P02768-1	Serum albumin [OS=Homo sapiens]	8	TRUE	5	7	3	609	69.3	6.28
FALSE	Master Protein	P35527	Keratin, type I cytoskeletal 9 [OS=Hom	6	TRUE	3	5	3	623	62	5.24
FALSE	Master Protein	P35908	Keratin, type II cytoskeletal 2 epiderm	15	TRUE	5	7	4	639	65.4	8
FALSE	Master Protein	P00751-1	Complement factor B [OS=Homo sapie	5	FALSE	3	4	3	764	85.5	7.06
FALSE	Master Protein	P00734	Prothrombin [OS=Homo sapiens]	6	FALSE	3	4	3	622	70	5.9
FALSE	Master Protein	P02769	Serum albumin OS=Bos taurus GN=ALE	4	TRUE	3	4	1	607	69.2	6.18
FALSE	Master Protein	P00747	Plasminogen [OS=Homo sapiens]	4	FALSE	2	4	2	810	90.5	7.24
FALSE	Master Protein	P01860	Immunoglobulin heavy constant gamr	16	FALSE	2	4	1	377	41.3	7.9
FALSE	Master Protein	P01857	Immunoglobulin heavy constant gamr	10	FALSE	2	4	1	330	36.1	8.19
FALSE	Master Protein	P0DOY2	immunoglobulin lambda constant 2 [(	24	FALSE	2	3	2	106	11.3	7.24
FALSE	Master Protein	P04259	keratin, type II cytoskeletal 6B [OS=Ho	6	TRUE	2	2	2	564	60	8
FALSE	Master Protein	P01876	immunoglobulin heavy constant alph	8	FALSE	2	2	2	353	37.6	6.51
FALSE	Master Protein	P01834	immunoglobulin kappa constant [OS=	16	FALSE	1	2	1	107	11.8	6.52
FALSE	Master Protein	P13671	Complement component c6 [OS=Hom	1	FALSE	1	2	1	934	104.7	6.76
FALSE	Master Protein	P13645	Keratin, type I cytoskeletal 10 [OS=Hor	2	TRUE	1	2	1	584	58.8	5.21
FALSE	Master Protein	P13647	keratin, type II cytoskeletal 5 [OS=Hom	3	TRUE	1	1	1	590	62.3	7.74
FALSE	Master Protein	P04196	Histidine-rich glycoprotein [OS=Homo	2	FALSE	1	2	1	525	59.5	7.5
FALSE	Master Protein	P0DOX6	immunoglobulin mu heavy chain [OS=	3	FALSE	2	2	2	576	63.4	7.87
FALSE	Master Protein	P01763	Immunoglobulin heavy variable 3-48 [	9	FALSE	1	1	1	117	12.8	5.36
FALSE	Master Protein	Q5D862	Filaggrin-2 [OS=Homo sapiens]	1	TRUE	1	1	1	2391	247.9	8.31
FALSE	Master Protein	Q04756	Hepatocyte growtH factor activator [O	1	FALSE	1	2	1	655	70.6	7.24
FALSE	Master Protein	P81605	Dermcidin [OS=Homo sapiens]	10	FALSE	1	1	1	110	11.3	6.54
FALSE	Master Protein	P03952	Plasma kallikrein [OS=Homo sapiens]	2	FALSE	1	1	1	638	71.3	8.22
FALSE	Master Protein	P00748	Coagulation factor XII [OS=Homo sapie	3	FALSE	1	2	1	615	67.7	7.74
FALSE	Master Protein	P10909-1	Clusterin [OS=Homo sapiens]	7	FALSE	2	2	2	449	52.5	6.27
FALSE	Master Protein	Q61726	Keratin type II (Fragment) OS=Mus mus	3	TRUE	1	1	1	495	54.9	6.46
FALSE	Master Protein	O75592-1	E3 ubiquitin-protein ligase MYCBP2 [O	1	FALSE	1	1	1	4640	509.8	7.03

Band 3

Checked	Master	Accession	Description	Coverage [%]	Contaminant	# Peptides	# PSMs	# Unique Peptides	# AAs	MW [kDa]	calc. pI
FALSE	Master Protein	P01024	Complement C3 [OS=Homo sapiens]	2	FALSE	4	4	4	1663	187	6.4
	Annotated Sequence	Modifications	Positions in Master Proteins	Theo. MH+ [Da]							
	[R].CAEENCFIQK.[S]	2xCarbamidomethyl [C1; C6]	P01024 [1513-1522]	1298.55038							
	[K].SGSDEVQVGQQR.[T]		P01024 [1571-1582]	1289.60803							
	[K].TGLQEVEVK.[A]		P01024 [905-913]	1002.5466							
	[R].WLNEQR.[Y]		P01024 [1255-1260]	845.42643							
FALSE	Master Protein	P01042	kininogen-1 [OS=Homo sapiens]	2	FALSE	1	2	1	644	71.9	6.81
FALSE	Master Protein	P00760	Cationic trypsin OS=Bos taurus PE=1 SV=3	12	TRUE	2	2	2	246	25.8	8.07
FALSE	Master Protein	P04264	Keratin, type II cytoskeletal 1 [OS=Homo sapiens]	3	TRUE	1	1	1	644	66	8.12
FALSE	Master Protein	Q61726	Keratin type II (Fragment) OS=Mus musculus GN=5	3	TRUE	1	1	1	495	54.9	6.46
FALSE	Master Protein	P35527	Keratin, type I cytoskeletal 9 [OS=Homo sapiens]	2	TRUE	1	1	1	623	62	5.24
FALSE	Master Protein	P01023	alpha-2-macroglobulin [OS=Homo sapiens]	1	TRUE	1	1	1	1474	163.2	6.46
FALSE	Master Protein	Q92608-1	Dedicator of cytokinesis protein 2 [OS=Homo sapi	2	FALSE	1	1	1	1830	211.8	6.87
FALSE	Master Protein	Q96PM5-6	Isoform 6 of RING finger and CHY zinc finger doma	8	FALSE	1	2	1	239	27.7	6.67
FALSE	Master Protein	Q969R2	Oxysterol-binding protein 2 [OS=Homo sapiens]	4	FALSE	1	1	1	916	101.2	6.54

c

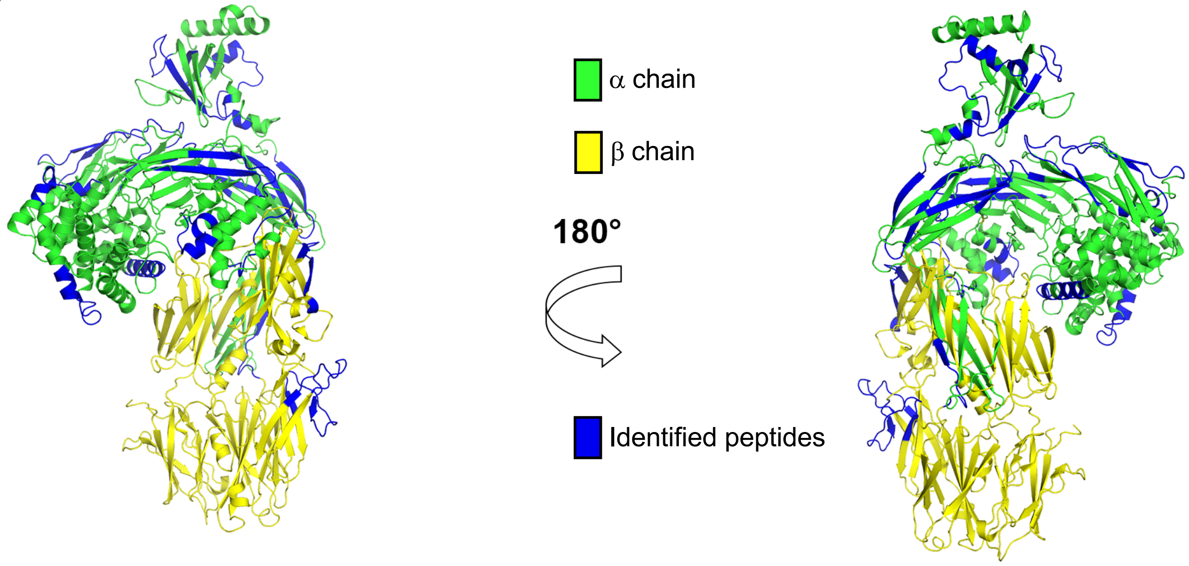


```
>sp|P01024|CO3_HUMAN Complement C3 OS=Homo sapiens OX=9606 GN=C3 PE=1 SV=2

MGPTSGPSLLLLLLTHLPLALGSPMYSIITPNILRLESEETMVLEAHDAQGDVPVTVTVHDFPGKKLVLSSEKTVLTPATNHMGNVTFTIPANREFKSEKGRNKFVTVQATFGTQVVEKV
VLVSLQSGYLFIQTDKTIYTPGSTVLYRIFTVNHKLLPVGRTVMVNIENPEGIPVKQDSLSSQNQLGVLPLSWDIPELVNMGQWKIRAYYENSPQQVFSTEFEVKEYVLPSFEVIVEPTE
KFYYIYNEKGLEVTITARFLYGKKVEGTAFVIFGIQDGEQRISLPESLKRIPIEDGSGEVVLSRKVLLDGVQNPRAEDLVGKSLYVSATVILHSGSDMVQAERSGIPIVTSPYQIHFTKT
PKYFKPGMPFDLMVFVTNPDGSPAYRVPVAVQGEDTVQSLTQGDGVAKLSINTHPSQKPLSITVRTKKQELSEAEQATRTMQALPYSTVGNSNNYLHLSVLRTELRPGETLNVNFLLRMD
RAHEAKIRYYTYLIMNKGRLLKAGRQVREPGQDLVVLPLSITTDFIPSFRLVAYYTLIGASGQREVVADSVWVDVKDSCVGSLVVKSGQSEDRQPVPGQQMTLKIEGDHGARVVLVAVDK
GVFVLNKKNKLTQSKIWDVVEKADIGCTPGSGKDYAGVFSDAGLTFTSSSGQQTAQRAELQCPQPAARRRRSVQLTEKRMDKVGKYPKELRKCCEDGMRENPMRFSCQRRTRFISLGEAC
KKVFLDCCNYITELRRQHARASHLGLARSNLDEDIIAEENIVSRSEFPESWLWNVEDLKEPPKNGISTKLMNIFLKDSITTWEILAVSMSDKKGICVADPFEVTVMQDFFIDLRLPYSVV
RNEQVEIRAVLYNYRQNQELKVRVELLHNPAFCSLATTKRRHQQTVTIPPKSSLSVPYVIVPLKTGLQEVEVKAAVYHHFISDGVRKSLKVVPEGIRMNKTVAVRTLDPERLGREGVQKE
DIPPADLSDQVPDTESETRILLQGTPVAQMTEDAVDAERLKHLIVTPSGCGEQNMIGMTPTVIAVHYLDETEQWEKFGLEKRQGALELIKKGYTQQLAFRQPSSAFAAFVKRAPSTWLTA
YVVKVFSLAVNLIAIDSQVLCGAVKWLILEKQKPDGVFQEDAPVIHQEMIGGLRNNNEKDMALTAFVLISLQEAKDICEEQVNSLPGSITKAGDFLEANYMNLQRSYTVAIAGYALAQMG
RLKGPLLNKFLTTAKDKNRWEDPGKQLYNVEATSYALLALLQLKDFDFVPPVVRWLNEQRYYGGGYGSTQATFMVFQALAQYQKDAPDHQELNLDVSLQLPSRSSKITHRIHWESASLLR
SEETKENEGFTVTAEGKGQGTLSVVTMYHAKAKDQLTCNKFDLKVTIKPAPETEKRPQDAKNTMILEICTRYRGDQDATMSILDISMMTGFAPDTDDLKQLANGVDRYISKYELDKAFSD
RNTLIIYLDKVSHSEDDCLAFKVHQYFNVELIQPGAVKVYAYYNLEESCTRFYHPEKEDGKLNKLCRDELCRCAEENCFIQKSDDKVTLEERLDKACEPGVDYVYKTRLVKVQLSNDFDE
YIMAIEQTIKSGSDEVQVGQQRTFISPIKCREALKLEEKKHYLMWGLSSDFWGEKPNLSYIIGKDTWVEHWPEEDECQDEENQKQCQDLGAFTESMVVFGCPN
```

Supplementary Fig. 1: Identification of ISG65 interaction partners in human serum using pull-down assays. a Coomassie-stained, reducing SDS-gel, representative of 3 independent experiments, showing IMAC elution fractions of ISG65 alone (left, negative control), human serum without bait (middle, negative control) and ISG65 with human serum (right). The arrow marks the positions of bands that are not present in both negative controls thus indicating a specifically enriched protein. Numbers indicate different elution fractions. Bands were excised and subjected to tryptic digestion and LC MS/MS analysis. **b** Results of the MS analysis. Databases of *Homo sapiens* and common contaminates were searched. Proteins are ranked by the number peptide spectrum matches (PSMs). Enriched fractions on the gel are highlighted with a red box. The gel bands from the enriched fractions, marked with an arrow, were analysed independently. In all 3 cases (BAND 1, 2 and 3) complement C3 was identified with the highest number of PSMs confirming the specific interaction of ISG65 and complement C3. **c** Identified peptides (blue) mapped onto the structure (2 orientations, top) and the sequence of C3 (bottom). The alpha chain is depicted in green, the beta chain in yellow. The majority of identified peptides are localised in the alpha chain incl. the ANA domain. Few peptides are also localised in the beta chain of C3. Only gel bands corresponding to the alpha chain were excised and analysed. Beta chain specific peptides likely originate from inaccurate manual cutting and spill over form the adjacent beta chain band. The mass spectrometry proteomics data have been deposited to the ProteomeXchange Consortium via the PRIDE[1] partner repository with the dataset identifier PXD036611

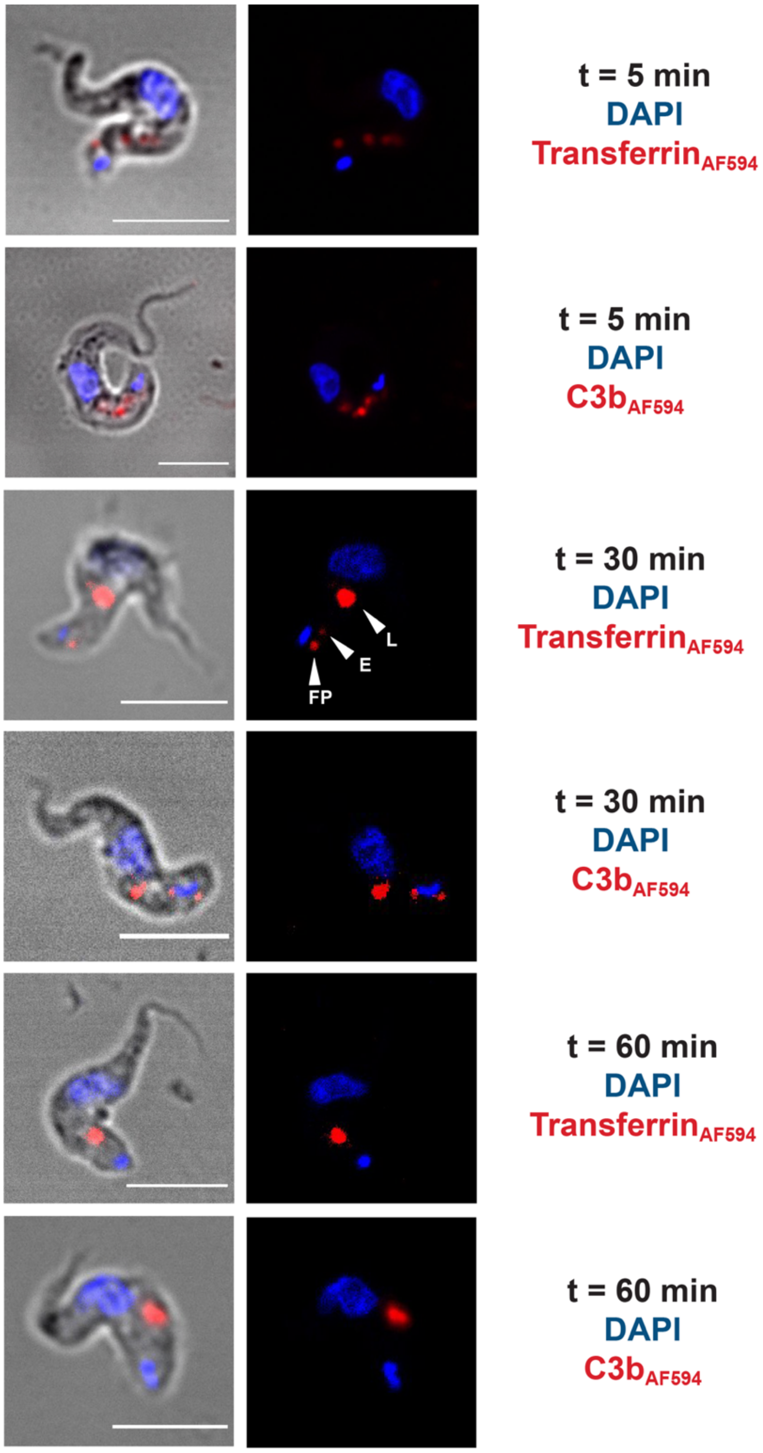

Supplementary Fig. 2: C3b is efficiently internalised by endocytosis in *T. b. gambiense* cells similar to transferrin. Representative fluorescence micrographs of cells incubated with AF_{594} labelled human transferrin or AF_{594} labelled human C3b for 5, 30 or 60 min, respectively (blue=DAPI, red= AF_{594}, left images are merged with brightfield channel; scale bar = 5 µm; each experiment was performed in triplicate). At t=5 min and t=30 min red signals are detectable at the flagellar pocket (FP; a defined membrane region at the flagellar base, dedicated to incoming and outgoing membrane traffic[2] the endosomal compartment (E) and the lysosome (L) (compartments indicated by white arrows for TF)). At t=60 min red signals are largely restricted to the lysosome.

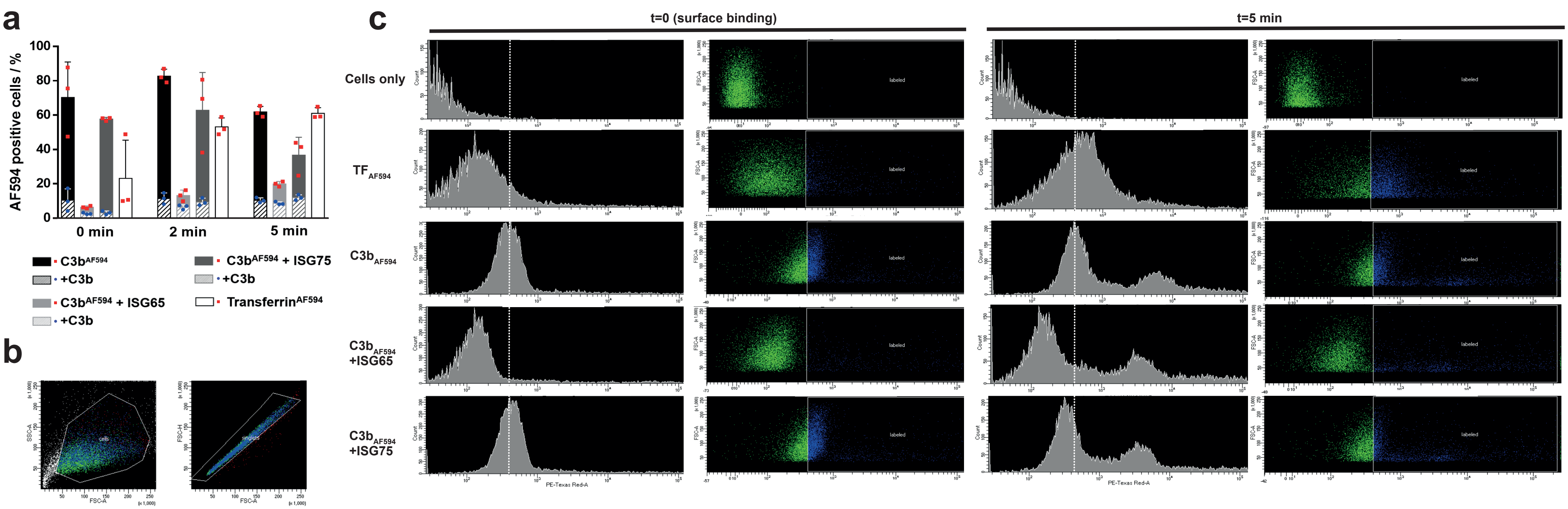

Supplementary Fig. 3: Flow cytometry analysis of C3b surface binding and uptake. *T.b. gambiense* cells were incubated with AF594 labelled human transferrin or AF594 labelled human C3b for 0 min at 4°C (surface binding conditions; see Methods part), for 2 min or 5 min at 37°C, respectively. Cells were then fixed and subjected to flow cytometry using an excitation wavelength of 560 nm in combination with a PE-Texas Red filter. **a** Bar graph plotting counts of AF594 positive cells (mean of three biological replicates with standard deviation) at 0, 2 and 5 min for C3b$_{AF593}$ alone (black) and in the presence of ISG65 (light gray) or ISG75 (dark gray). Respective experiments with transferrin$_{AF593}$ (white) are shown for comparison. Non-labelled C3b added at 4-fold molar excess (superimposed, hatched bars) competes with C3b $_{AF593}$ surface binding and uptake. Individual replicate values are indicated as red boxes or, for non-labelled C3b competition experiments, as blue circles. Source data are provided as a Source Data file. **b** A refined gate based on size versus granularity (FSC-A/SSC-A plot) was applied to exclude cell debris. Secondly singlets were gated (FSC-A/FSC-H plot). **c** Lastly, a gate ('labelled', blue) was defined for AF$_{594}$ positive cells (PE-Texas Red-A/FSC-A plot) based on exclusion of signals from non-treated control cells (upper panel; cells only), to define AF$_{594}$ positive cells. Histogram plots of cell count versus fluorescence intensity (Count/PE-Texas Red-A) are shown, with the gate indicated as dashed line. For flow cytometry plots of additional timepoints, competition experiments with non-labelled C3b and all replicates see 'Supplementary Data 1'.

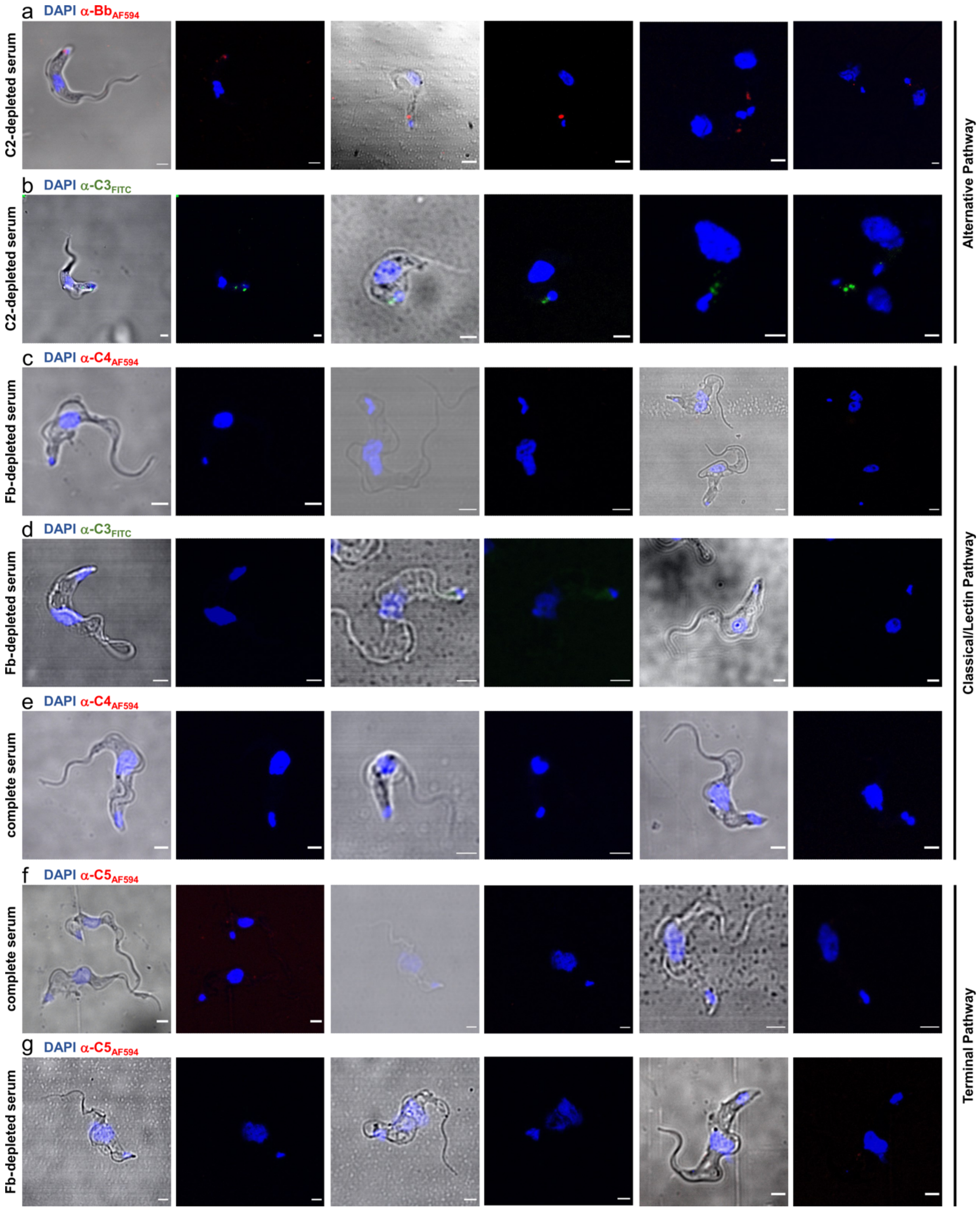
a DAPI α-Bb AF594
C2-depleted serum
b DAPI α-C3 FITC
C2-depleted serum
Alternative Pathway
c DAPI α-C4 AF594
Fb-depleted serum
d DAPI α-C3 FITC
Fb-depleted serum
e DAPI α-C4 AF594
complete serum
Classical/Lectin Pathway
f DAPI α-C5 AF594
complete serum
g DAPI α-C5 AF594
Fb-depleted serum
Terminal Pathway

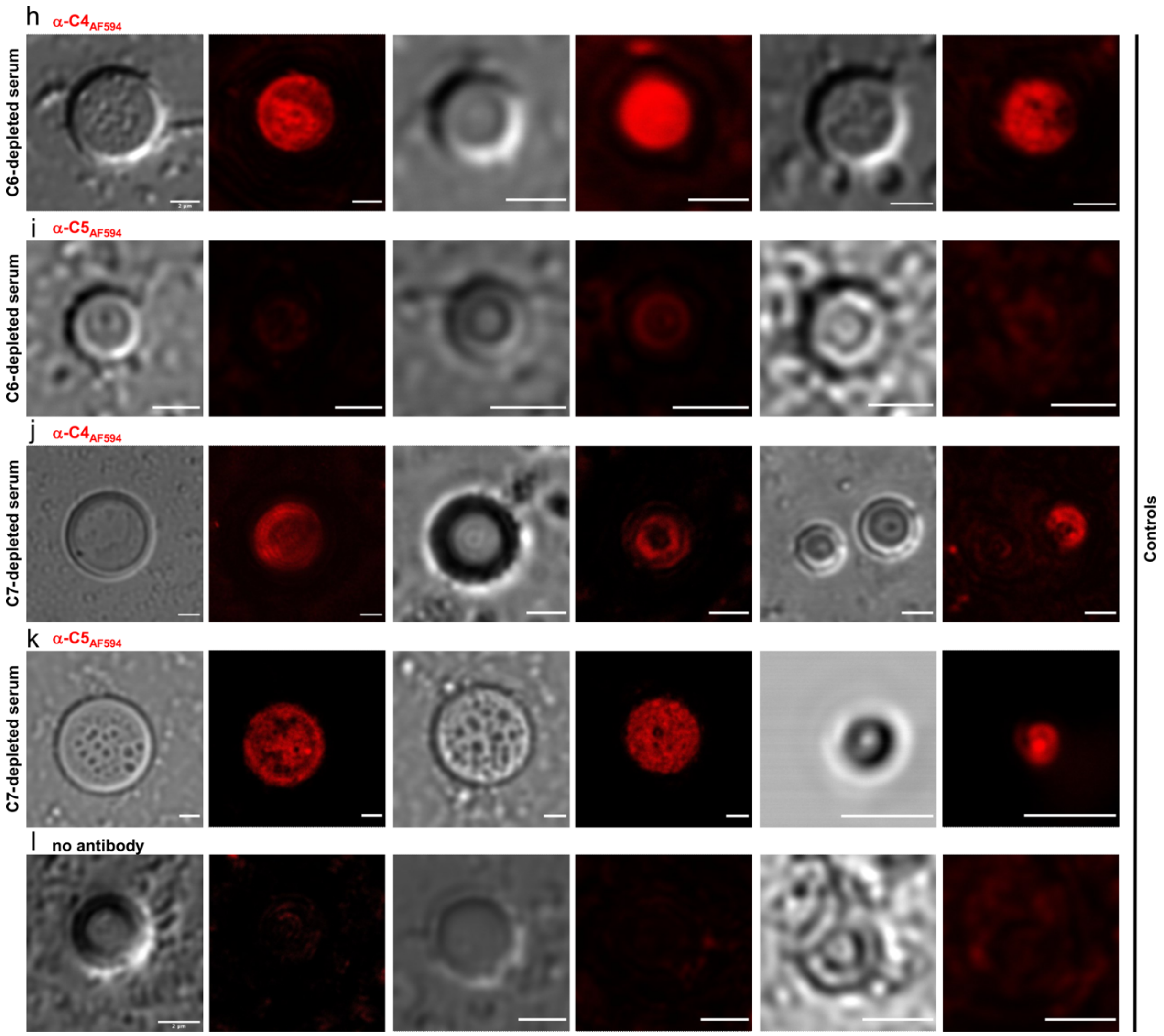

Supplementary Fig. 4: Bb and C3b are deposited on the surface of T. brucei gambiense in a C2-independent manner, while C4b and C5b are not detected by immunofluorescence. Representative fluorescence micrographs of trypanosomes incubated in depleted or complete human serum tested for deposition of complement factors using fluorescently-labelled antibodies as indicated (red= AF594, green=FITC; blue=DAPI stain; for most images a merge with the brightfield channel is shown; scale bar = 2 µm; independent experiments were performed at least in triplicate). Note, that even at low temperatures, molecules bound to the trypanosome surface are rapidly internalized, accumulating in the flagellar pocket before delivery to the early endosome.

Complement Pathways corresponding to the detected antigen are indicated. **a, b** When incubated in C2-depleted serum, C3b and Bb are robustly detected at the flagellar pocket of *T. b. gambiense*. **c** C4b is not detectable upon incubation in fB-depleted serum. **d** In fB-depleted serum, C3b is not detectable on most cells. A residual, faint stain is visible in a small minority of cells (middle) and could be attributed to binding of fluid-phase C3b that does not get amplified. **e** C4b is also not detectable upon incubation in complete serum. **f, g** C5b is not detectable upon incubation in fB-depleted or complete serum. **h-l** Control experiments demonstrating functionality of fluorescently-labelled antibodies. Sensitised sheep erythrocytes were incubated in human serum depleted for complement factors C6 or C7 in order to prevent progression of the TP and complement lysis (brightfield (left) and AF594 fluorescence (right); scale bar = 2 µm).

After incubation in C6-depleted human serum only C4b can be detected (**h**) while the anti-C5-AF594 signal is largely absent (**i**), comparable to control experiments omitting antibody addition (**l**). This is likely due to the prerequisite of C6 binding to C5b for membrane deposition[3].

Both, C4b and C5b are detected upon incubation of erythrocytes in C7-depleted serum (**j, k**).

a

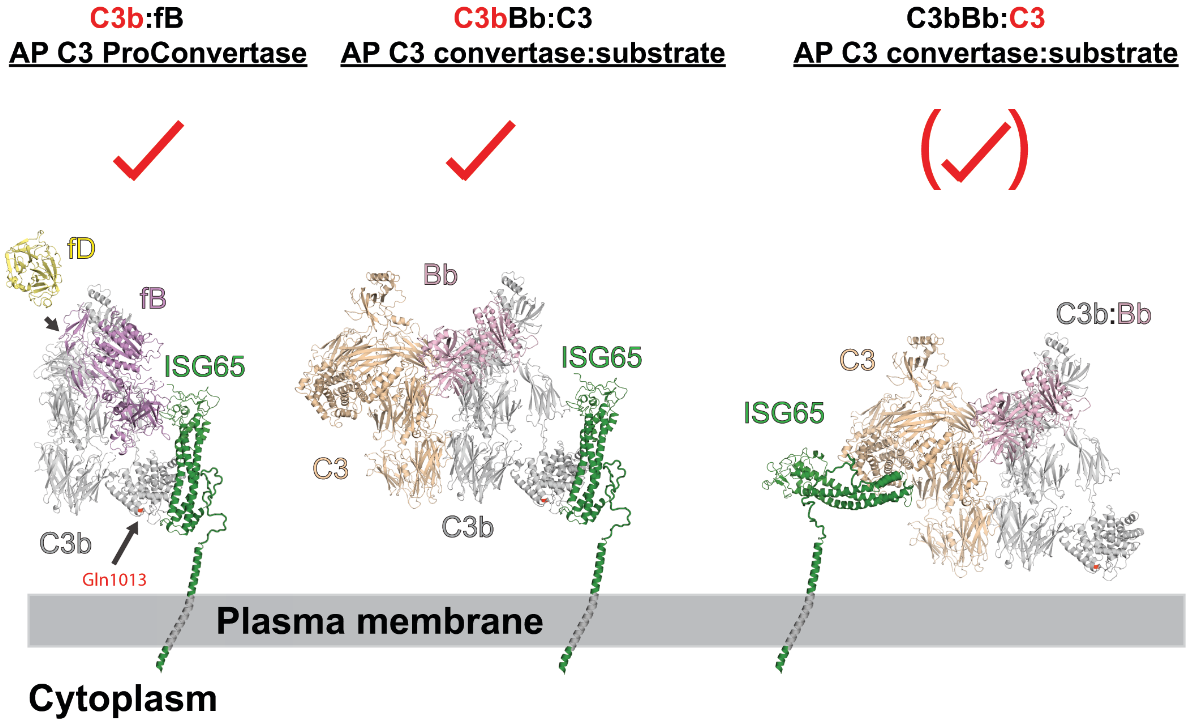

b

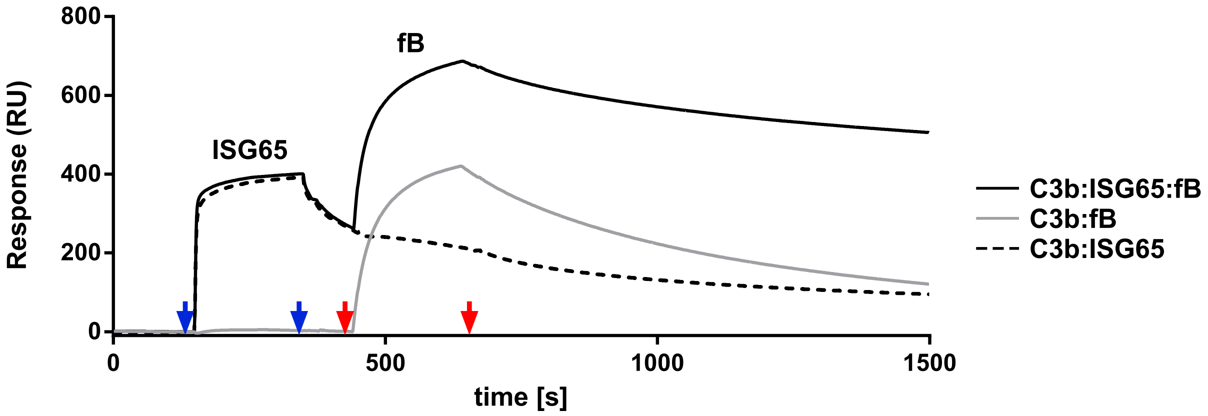

Supplementary Fig. 5: AP pro-convertase formation and potential organisation of AP C3 convertase complexes in presence of ISG65. a Possible assemblies are marked with a red tick, possible, but unlikely interactions with a red tick in brackets. The ISG65 interacting C3 fragment is indicated with red letters. Notably, C3 binding to the parasite surface has not been demonstrated. ISG65-bound C3b (left) can interact with factor B (fB) followed by factor D (fD), allowing the formation of the C3 ProConvertase C3bfB. Gln1013 of the former thioester bond, which attaches to hydroxy groups on the cell surface, is labelled in red and marked with an arrow. ISG65-bound C3 convertase (middle) would be able to bind the C3 substrate according to the model proposed by Rooijakkers *et al.*[4]. ISG65-bound C3 substrate would be able to interact with surface-bound C3 convertase only if the C3-binding domain of ISG65 would be oriented parallel to the plasma membrane (right). While its flexible C-terminal linker would in principle allow such an orientation, it contradicts the current view on the organisation of the VSG coat as well as the orientation of embedded surface receptors. VSGs constituting the coat are tightly packed and thus are unlikely to allow for a space-filling orientation of a receptor with a bulky ligand bound. Moreover, the loop-rich head domains of VSGs and other surface proteins act as immune decoys and have to be membrane-distal in order to exert their function.

The C3bBb:C3 complex is based on molecular modelling. C3 substrate hereby replaces C3b in an inhibitor-mediated, dimeric structure of C3 convertase (PDB: 2WIN). All other C3 complexes are crystal structures. The ISG65-interacting C3 species within the complexes are depicted in red letters. **b** SPR sensorgrams showing the AP pro-convertase formation in presence of ISG65. C3b was immobilised onto the chip and ISG65 and factor B subsequently injected (solid black). Control injections were performed with ISG65 (black dashes) or factor B (solid grey) only. Beginning and end of the injections are marked with arrows (ISG65: blue, factor B: red). Source data are provided as a Source Data file.

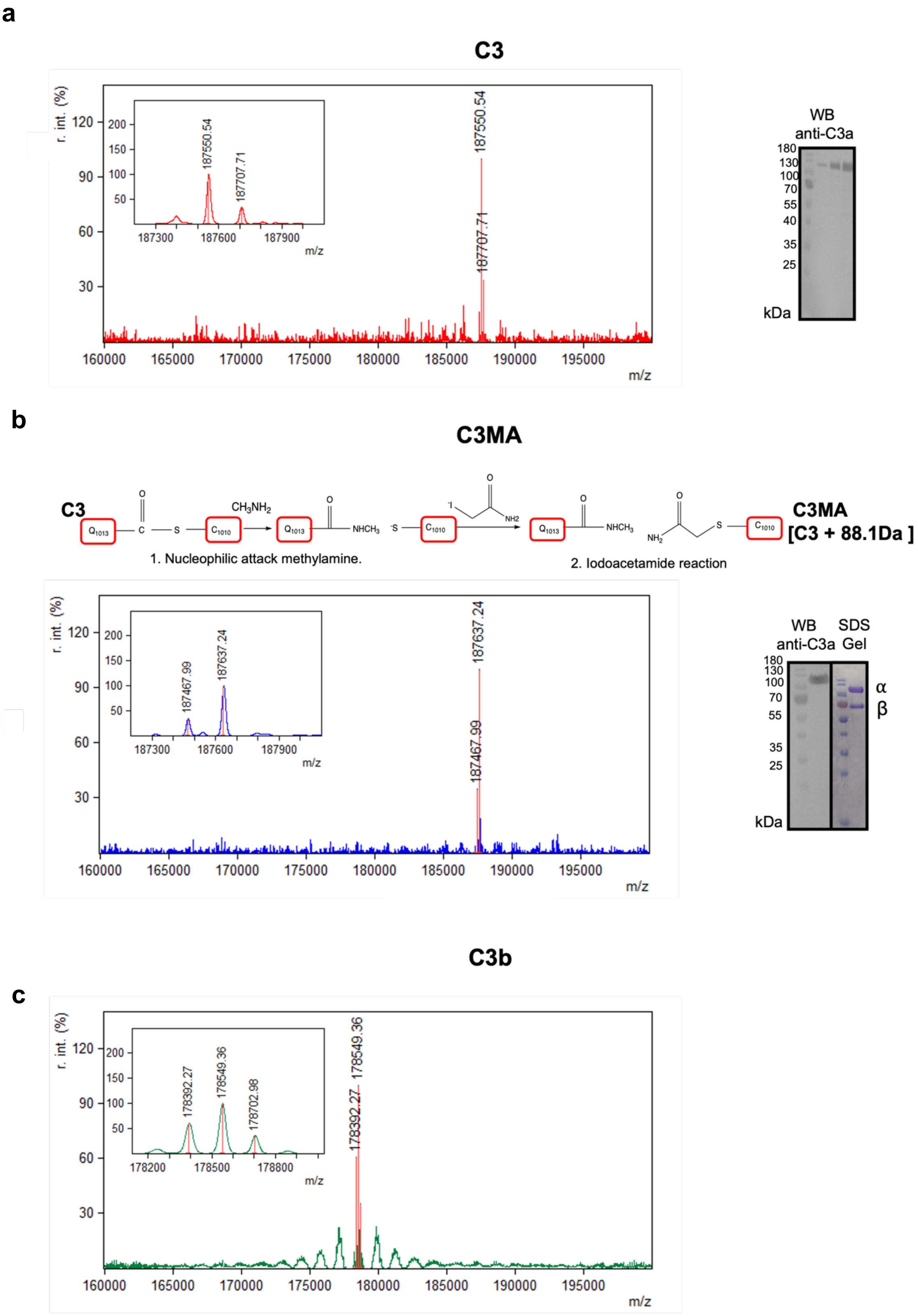
a
C3
r. int. (%)
120
90
60
30
200
150
100
50
187550.54
187707.71
187300
187600
187900
m/z
160000
165000
170000
175000
180000
185000
190000
195000
WB
anti-C3a
180
130
100
70
55
40
35
25
kDa
b
C3MA
C3
Q_{1013}
C
S
C_{1010}
CH_3NH_2
$NHCH_3$
NH_2
1. Nucleophilic attack methylamine.
2. Iodoacetamide reaction
C3MA
[C3 + 88.1Da]
187637.24
187467.99
WB
anti-C3a
SDS
Gel
α
β
c
C3b
178549.36
178392.27
178702.98
178200
178500
178800

d

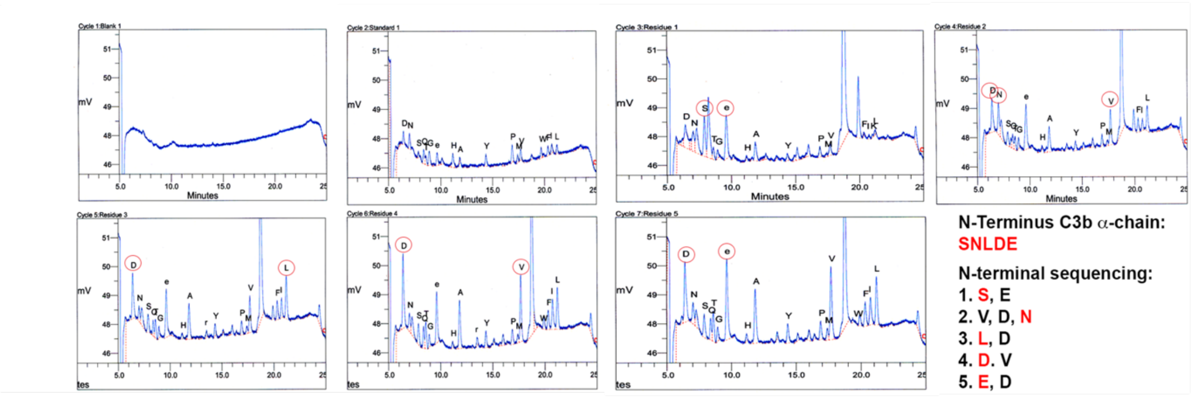

e

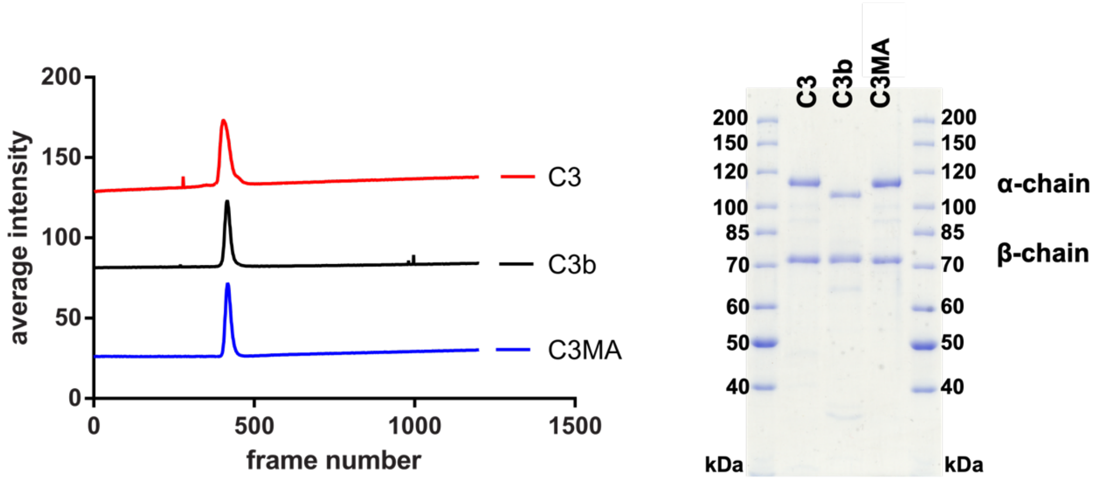

f

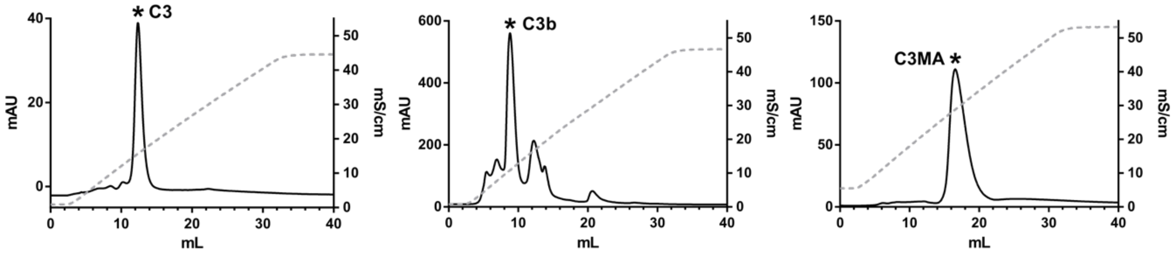

Supplementary Fig. 6: Validation of C3 fragment identities using intact mass spectrometry, Western blotting, N-terminal sequencing, small-angle X-ray scattering and ion-exchange chromatography. a C3 (theoretical mass without modifications 184,326.59) **–** The mass spectrum shows the highest-intensity peak at 187550.54 Da (left, close-up). This confirms the presence of a C3-fragment with an intact alpha chain (incl. ANA/C3a). The presence of ANA/C3a was further confirmed by western blotting (right). Displayed Western blot is representative for 3 independent experiments. **b C3MA** (theoretical mass without modifications 184,414.69) **–** Stable C3MA was generated from C3 using a 2-stage reaction. Methylamine was used to break the thioester bond and iodoacetamide to render the thiol-group unreactive. The mass spectrum shows the highest-intensity peak at 187637.24 Da (left, close-up). A mass difference of 86.7 Da was measured between C3 and C3MA. This difference agrees with the predicted mass according to the reaction scheme within a 7.5 ppm error of the measurement. The presence of ANA/C3a was further confirmed by Western blotting using an anti-C3a antibody that specifically recognises the intact alpha chain (right). Displayed Western blot and SDS-gel are representatives of 3 independent experiments. **c C3b** (theoretical mass without modifications 175,249.96) **–** C3b was generated from native C3 by proteolytic digestion. The mass spectrum shows the highest-intensity peak at 178549.36 Da, confirming the absence of ANA/C3a (close-up, left). The differences between theoretical and measured masses originate in

post-translational modifications. All spectra were produced in the mMass software (mmass.org)[5]. **d** The identity of C3b was further validated by N-terminal sequencing (bottom). The first 2 chromatograms show blank and standards, respectively. In all chromatograms peak areas were integrated to determine the most probable residue for each of the first five positions in the alpha chain. For each position residues with the largest peak area are encircled. The sequence SNLDE represents the new N-terminus after proteolytic cleavage of ANA/C3a. **e** SEC-SAXS traces of all 3 C3 fragments. No additional peaks are visible, thus confirming the monodispersity of the samples. All three samples have the same level of purity based on their scattering profiles (left) and residual impurities visible on a Coomassie-stained 8% SDS-gel (right). **f** C3 fragments have specific retention times in ion-exchange chromatography.

The mass spectrometry proteomics data have been deposited to the ProteomeXchange Consortium via the PRIDE[1] partner repository with the dataset identifier PXD036611. Source data are provided as a Source Data file.

Supplementary Table 1: Cryo-EM data collection, refinement and validation statistics.

	#1 ISG65:C3 (EMDB-14707) (PDB- 7ZGJ)	#2 ISG65:C3b (EMDB-14708) (PDB- 7ZGK)
**Data collection and processing**		
Microscope	Titan Krios	Titan Krios
Detector	K3	K2
Magnification (nominal)	105.000	165.000
Voltage (kV)	300	300
Spherical aberration	2.7 mm	2.7
Total electron dose (e–/Å^2)	41.1	60
Defocus range (μm)	-2.5 to -1.5	n.a. (merged datasets)
Pixel size (Å)	0.86	0.828
Stage tilt	25°	0° , 25°
Number of Micrographs	10380	27022
Final particle images (no.)	406.545	145.172
Map resolution (Å)<br>FSC threshold	3.58 ($FSC_{0.143}$)	3.59 ($FSC_{0.143}$)
**Refinement**		
Initial model used (PDB code)	2A73	2I07
RMSZ		
Bond lengths	0.25	0.25
Bond angles	0.47	0.46
Validation		
MolProbity score	1.12	1.34
Clashscore, all-atom	3	5
Poor rotamers	0.7%	0.5
Ramachandran plot		
Favoured	98%	98%
Allowed	2%	2%
Outliers	0%	0%

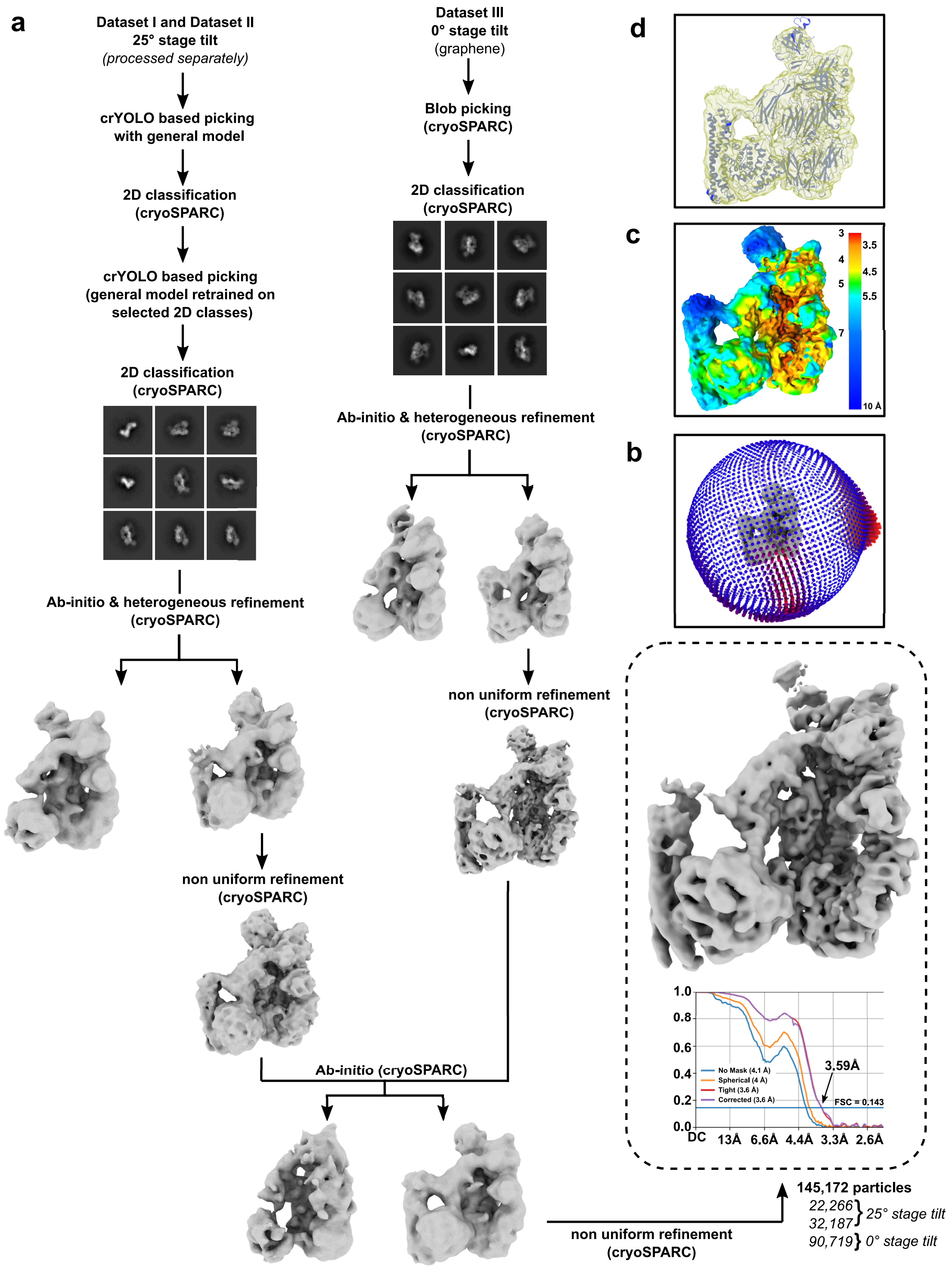
a
Dataset I and Dataset II
25° stage tilt
(processed separately)
crYOLO based picking
with general model
2D classification
(cryoSPARC)
crYOLO based picking
(general model retrained on
selected 2D classes)
2D classification
(cryoSPARC)
Ab-initio & heterogeneous refinement
(cryoSPARC)
non uniform refinement
(cryoSPARC)
Dataset III
0° stage tilt
(graphene)
Blob picking
(cryoSPARC)
2D classification
(cryoSPARC)
Ab-initio & heterogeneous refinement
(cryoSPARC)
non uniform refinement
(cryoSPARC)
Ab-initio (cryoSPARC)
non uniform refinement
(cryoSPARC)
145,172 particles
22,266
32,187
25° stage tilt
90,719
0° stage tilt
d
c
3
3.5
4
4.5
5
5.5
7
10 Å
b
1.0
0.8
0.6
0.4
0.2
0.0
No Mask (4.1 Å)
Spherical (4 Å)
Tight (3.6 Å)
Corrected (3.6 Å)
3.59Å
FSC = 0.143
DC
13Å
6.6Å
4.4Å
3.3Å
2.6Å

e

Dataset I , 25° stage tilt
C/Flat, Cu, 1.2/1.3 300 mesh

Dataset I , 25° stage tilt
C/Flat, Cu, 2/2 300 mesh

Dataset III ,0° stage tilt
Quantifoil, Au, R1.2/1.3 300 mesh, graphene coated

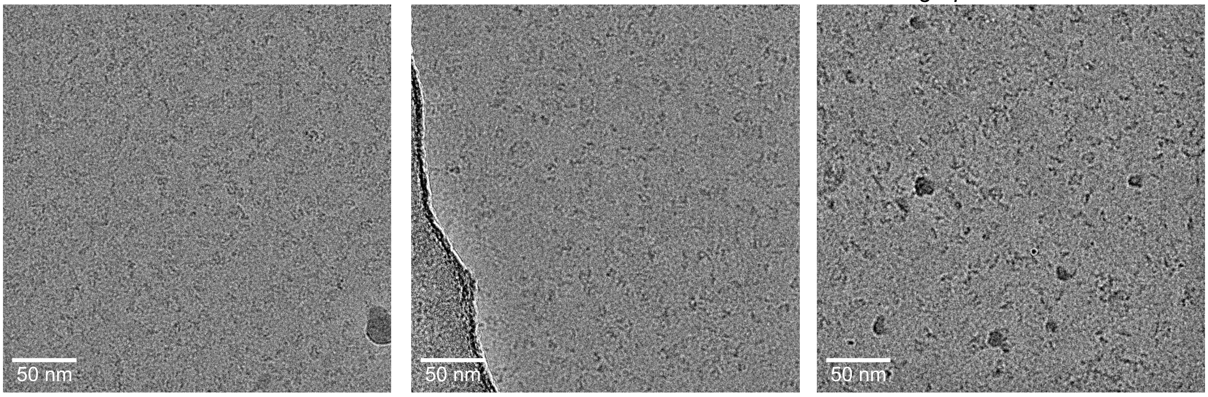

Supplementary Fig. 7: Cryo-EM single-particle analysis of ISG65 in complex with C3b. **a** Visual representation of the image processing workflow used to reconstruct the ISG65:C3b complex using three combined datasets. Datasets I and II were collected at 25° stage tilt and processed separately following the outlined schematic. Representative ab-initio models and refined maps are shown. Dataset III was collected without stage tilt and processed as outlined. Particles contributing to the refined reconstructions for each of the three datasets were combined and subjected to ab-initio modelling. A total of 145,172 particles were identified as the desired complex of ISG65 and C3b. An unsharpened map of the final reconstruction and the gold-standard FSC curve using different masks are highlighted with a dashed line. **b** Angular distribution plot of the ISG65:C3b complex. The height of the bars corresponds to the relative abundance of particle views contributing to the final reconstruction. **c** Colour-graded representation of the local resolution (in Å) of the final reconstruction. **d** Display of the overall fit of the ISG65:C3b model (blue) to the electron density of the final reconstruction (yellow). **e** Single-frame, motion-corrected micrographs representative of the indicated data collection are shown. Used TEM grid-type and stage tilt angle used for data acquisition are indicated above the corresponding micrographs.

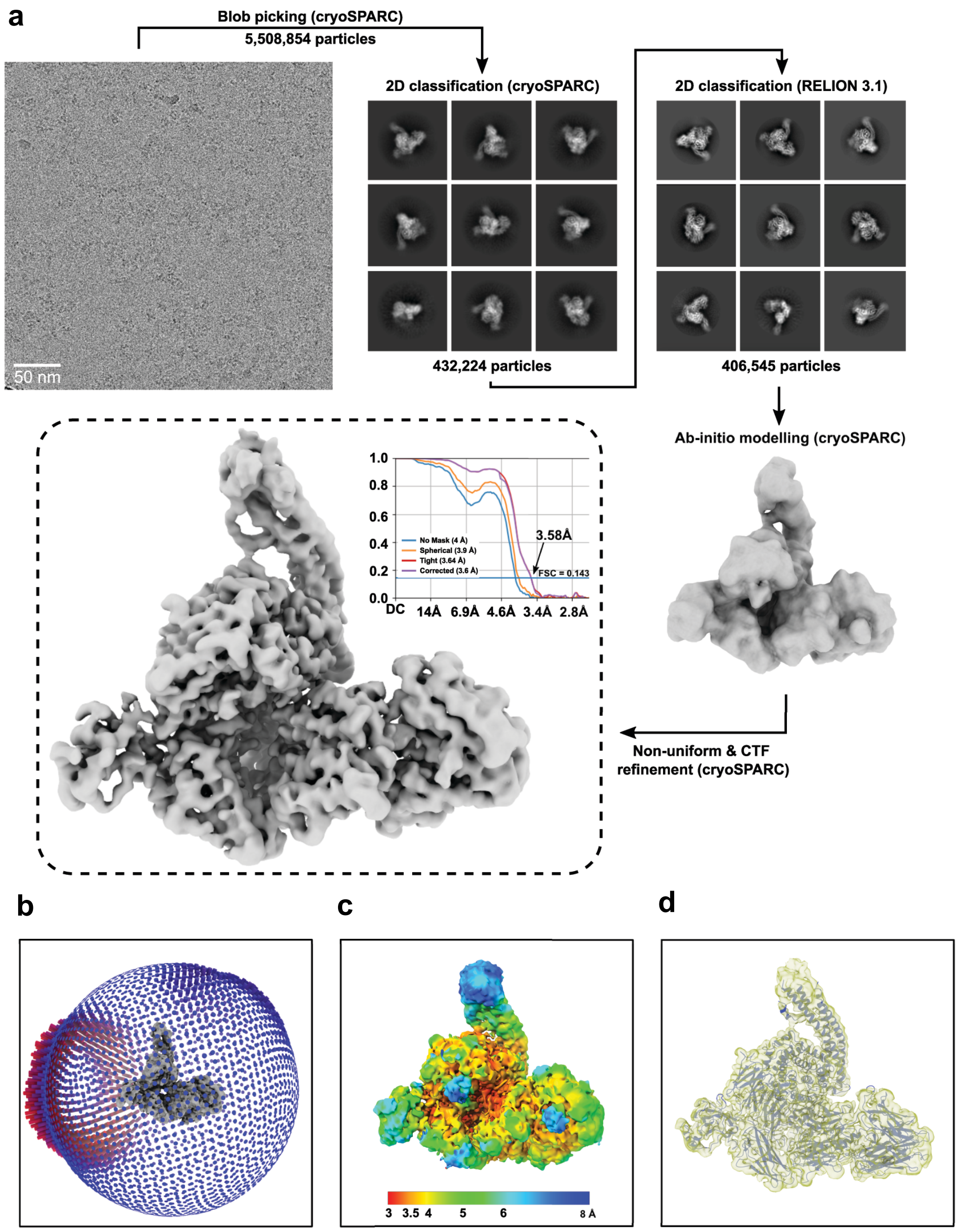

Supplementary Fig. 8: Cryo-EM single-particle analysis of ISG65 in complex with C3. **a** Visual representation of the image processing workflow used to reconstruct the ISG65:C3 complex. A representative micrograph after motion correction (top left), two sets of representative, reference-free 2D classes (top middle, top right) and an ab-initio model (bottom right) are shown. An unsharpened map of the final reconstruction and the gold-standard FSC curve using different masks are highlighted with a dashed line. **b** Angular distribution plot of the ISG65:C3 complex. The height of the bars corresponds to the relative abundance of particle views contributing to the final reconstruction. **c** Colour-graded representation of the local resolution (in Å) of the final reconstruction. **d** Display of the overall fit of the ISG65:C3 model (blue) to the electron density of the final reconstruction (yellow).

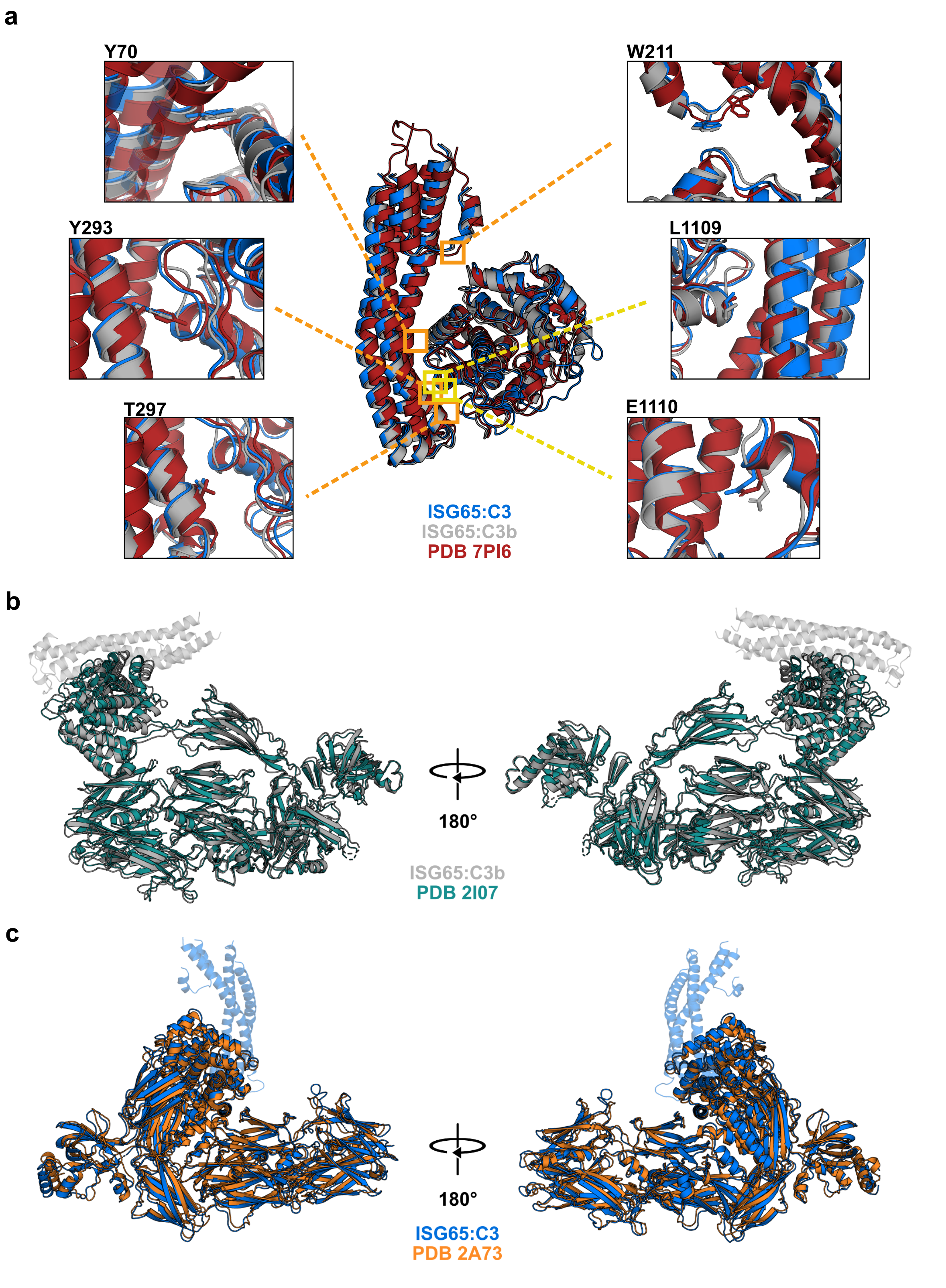

Supplementary Fig. 9: Comparison of ISG65:C3 and ISG65:C3b with related structures.
a Overlay of the minimal interacting domains of *T. b. gambiense* ISG65 in complex with C3 (blue) and C3b (grey), determined by cryo-EM, with the crystal structure of *T. b. brucei* ISG65 in complex with C3d (PDB 7PI6) (red). Residues demonstrated to facilitate the interaction are highlighted on ISG65 (orange boxes) and C3d (yellow boxes). Close-up views of the highlighted residues are shown. Residues of interest are depicted as sticks. ISG65 was modelled for the ISG65:C3 complex and docked and real-space refined in the map of ISG65:C3b. With an overall root-mean-square-deviation (RMSD) of 1.33 Å (calculated using Pymol Align[6] with 3013 atoms), the interface is highly similar in both, the ISG65:C3 and ISG65:C3b complexes. Both structures also closely resemble the reported

model of *T.b.b.* ISG65 in complex with C3d ($RMSD_{ISG65:C3\ vs\ 7PI6}$: 1.72 Å (1791 atoms), $RMSD_{ISG65:C3b\ vs\ 7PI6}$: 1.03 Å (1954 atoms) (calculated using Pymol Align). **b** Overlay of C3b from ISG65:C3b (grey) with the crystal structure of free C3b (PDB 2I07) (teal). An overall RMSD of 1.98 Å (calculated using Pymol Align, using 11068 atoms) indicates a highly similar arrangement. The most noticeable differences can be observed in the placement of alpha-helices in the thioester domain and the arrangement of the flexibly tethered C345C domain. ISG65 (transparent, no outline) has been excluded from the alignment and is only depicted for completeness. **c** Overlay of C3 from ISG65:C3 complex (blue) with the crystal structure of free C3 (PDB 2A73) (orange). An overall RMSD of 1.98 Å (calculated using Pymol Align, using 11878 atoms) indicates a highly similar arrangement. ISG65 (transparent, no outline) has been excluded from the alignment and is just depicted for completeness.

a

C43 – C200

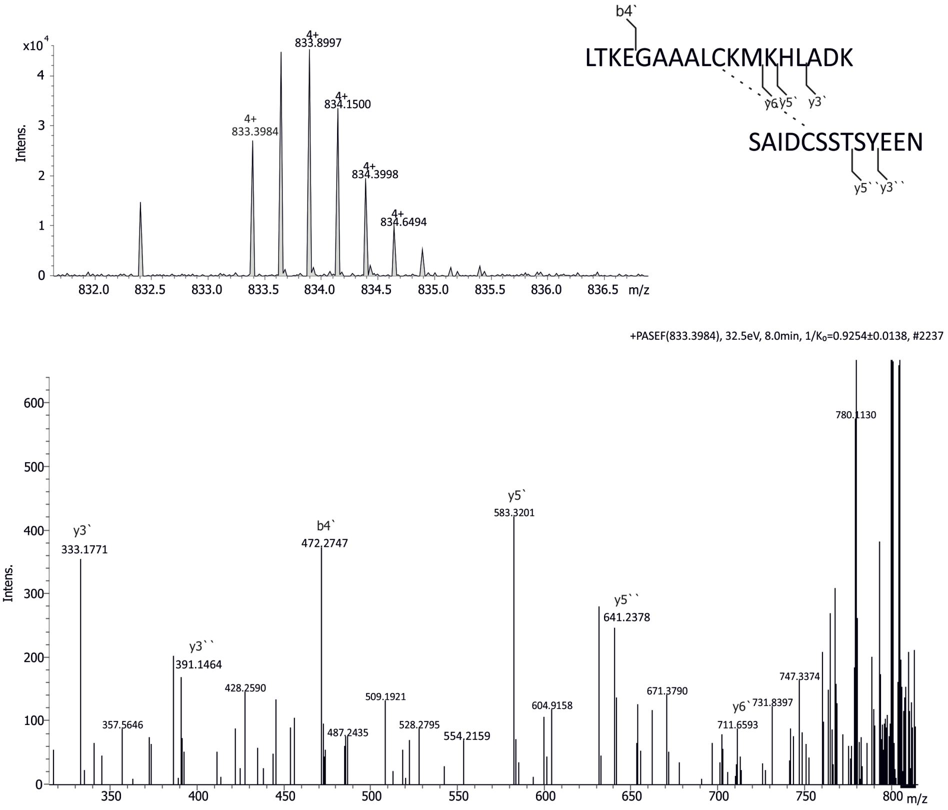

b

C168 – C181

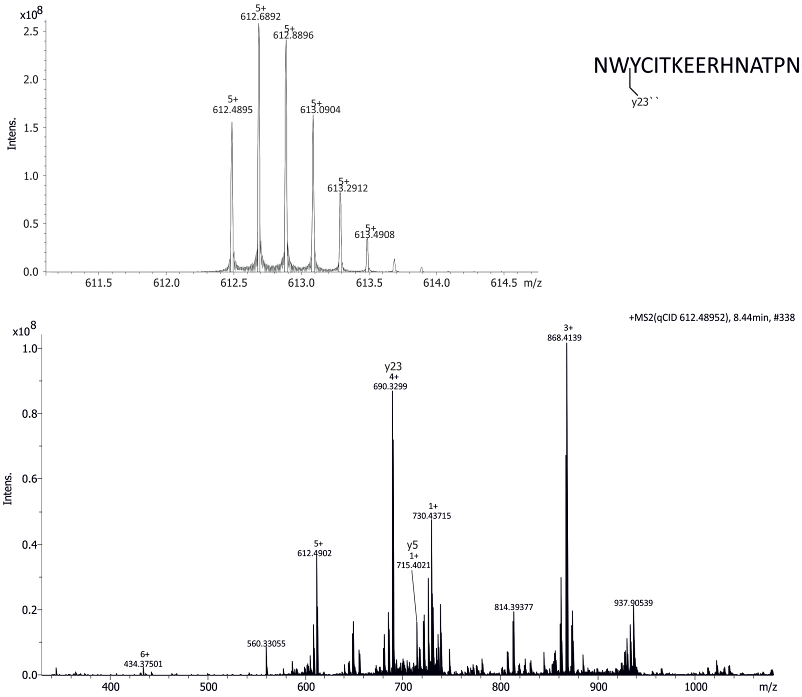

c

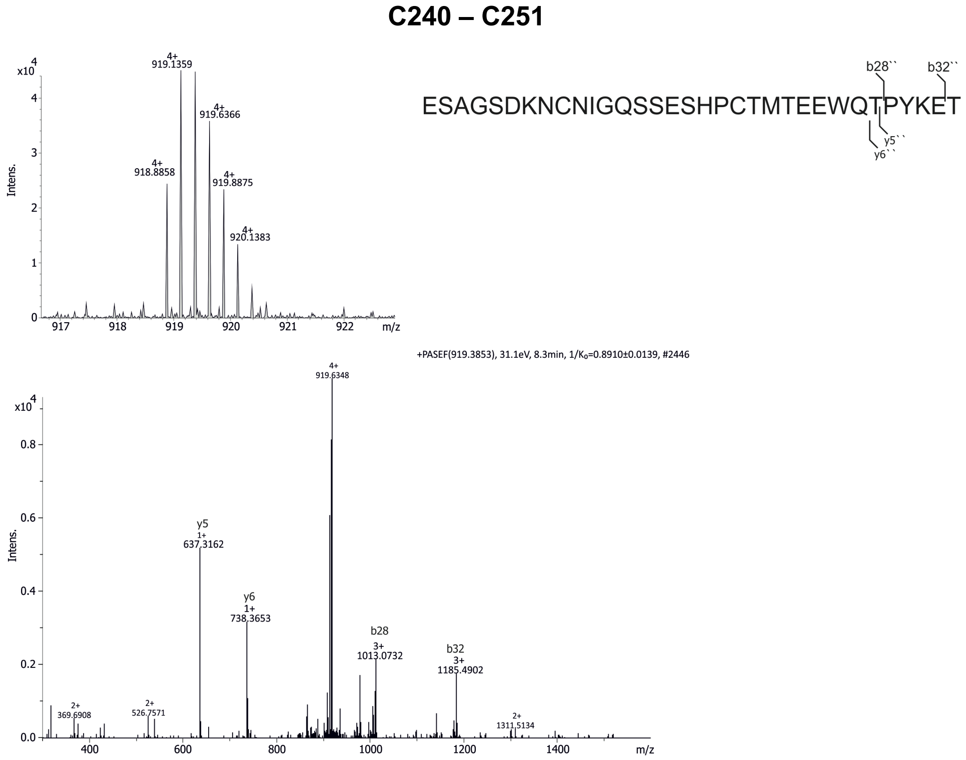
C240 – C251
ESAGSDKNCNIGQSSESHPCTMTEEWQTPYKET
b28``
b32``
y5``
y6``
Intens.
m/z
+PASEF(919.3853), 31.1eV, 8.3min, 1/K0=0.8910±0.0139, #2446
y5 1+ 637.3162
y6 1+ 738.3653
b28 3+ 1013.0732
b32 3+ 1185.4902

d

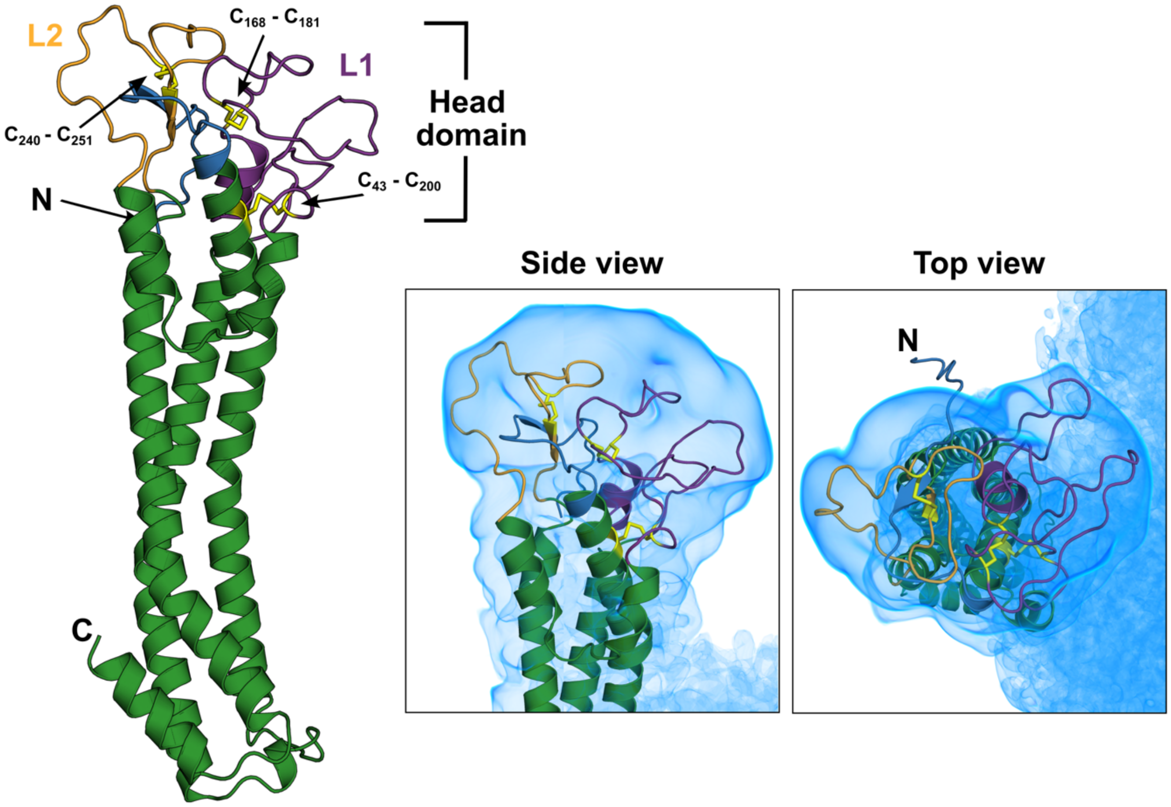
L2
L1
C168 - C181
C240 - C251
C43 - C200
N
C
Head domain
Side view
Top view

Supplementary Fig. 10: Disulphide mapping and modelling of the ISG65 head domain.
a-c Detailed MS spectra and CID MS/MS spectra of three disulphide linked peptides of ISG65. ISG65 was digested with Nepenthesin-2 under non-reducing conditions at pH 2.3. Fragments corresponding to the individual peptides of the disulphide linked peptide are marked with "`" for the first, and with "``" for the second peptide. **d** ISG65 hybrid model and locally sharpened cryo-EM density map around the head domain. The boundaries of the density cloud and disulphide positions were used as constraints in model selection. The disordered N-terminus and 2 loops in the head domain of ISG65 were modelled onto the cryo-EM structure using a template-based AlphaFold2 model[7]. The N-terminal region is coloured in blue, Loop 1 in purple and Loop 2 in orange. Disulphide bonds are coloured in yellow. Positions of N- and C-terminus are indicated. The mass spectrometry proteomics data have been deposited to the ProteomeXchange Consortium via the PRIDE[1] partner repository with the dataset identifier PXD033606.

Supplementary Table 2: List of atom-atom interactions across the ISG65:C3 interface.
The interface between ISG65 and C3 is created mainly by hydrophobic contacts and four hydrogen bonds with the thioester domain. Two hydrogen bonds (dark grey) are formed with residues in ANA/C3a. Residues that were mutated for SPR analysis are depicted in light grey.

Hydrogen bonds

	Atom name	Res name	Res nº	Chain		Atom name	Res name	Res nº	Chain	Distance
1	OH	TYR	293	C	---	O	LYS	1111	B	2.3
2	OH	TYR	293	C	---	NE2	GLN	1119	B	2.7
3	OH	TYR	293	C	---	O	GLU	1110	B	3.1
4	NH2	ARG	77	C	---	O	LEU	1109	B	1.8
5	OE1	GLU	302	C	---	N	GLY	717	B	3.0
6	NZ	LYS	97	C	---	OE1	GLU	689	B	3.0

Non-bonded contacts

	ATOM A					ATOM B				
	Atom name	Res name	Res nº	Chain		Atom name	Res name	Res nº	Chain	Distance
1	CH2	TRP	211	C	---	CA	GLY	1204	B	3.5
2	CZ2	TRP	211	C	---	CA	GLY	1204	B	3.8
3	OH	TYR	70	C	---	CG2	THR	1170	B	3.7
4	OH	TYR	70	C	---	CB	THR	1170	B	3.7
5	CZ	PHE	73	C	---	OG	SER	1164	B	3.7
6	CE1	PHE	73	C	---	OG	SER	1164	B	3.4
7	CZ	PHE	73	C	---	CB	SER	1164	B	3.4
8	CE1	PHE	73	C	---	CB	SER	1164	B	2.9
9	CD1	PHE	73	C	---	CB	SER	1164	B	3.6
10	CG2	THR	297	C	---	OE1	GLN	1119	B	3.6
11	CB	THR	297	C	---	OE1	GLN	1119	B	3.7
12	CZ	TYR	293	C	---	NE2	GLN	1119	B	3.3
13	CE2	TYR	293	C	---	NE2	GLN	1119	B	3.2
14	OH	TYR	293	C	---	CD	GLN	1119	B	3.2
15	OH	TYR	293	C	---	CG	GLN	1119	B	3.7
16	CG	TYR	293	C	---	CD	PRO	1114	B	3.9
17	OD1	ASN	290	C	---	CG	PRO	1114	B	3.3
18	OD1	ASN	290	C	---	CB	PRO	1114	B	3.6
19	OH	TYR	293	C	---	CG	LYS	1113	B	3.6
20	CZ	TYR	293	C	---	CG	LYS	1113	B	3.7
21	CE2	TYR	293	C	---	CG	LYS	1113	B	3.4
22	CZ	TYR	293	C	---	CA	LYS	1113	B	3.8
23	CZ	TYR	293	C	---	O	GLN	1112	B	3.4
24	CE1	TYR	293	C	---	O	GLN	1112	B	3.0
25	CD1	TYR	293	C	---	O	GLN	1112	B	3.8
26	OH	TYR	293	C	---	C	GLN	1112	B	3.5
27	CZ	TYR	293	C	---	C	GLN	1112	B	3.8
28	CE1	TYR	293	C	---	C	GLN	1112	B	3.8
29	CZ	TYR	293	C	---	O	LYS	1111	B	3.5
30	OH	TYR	293	C	---	C	LYS	1111	B	3.1
31	OH	TYR	293	C	---	CA	LYS	1111	B	3.7
32	CG	ASN	296	C	---	OE2	GLU	1110	B	3.8
33	CZ	TYR	293	C	---	O	GLU	1110	B	3.4
34	CE1	TYR	293	C	---	O	GLU	1110	B	3.0
35	CZ	PHE	73	C	---	CD2	LEU	1109	B	3.9
36	CZ2	TRP	81	C	---	O	LEU	1109	B	3.6
37	CZ	ARG	77	C	---	O	LEU	1109	B	3.1
38	NH2	ARG	77	C	---	C	LEU	1109	B	2.8
39	NH2	ARG	77	C	---	CA	LEU	1109	B	3.1
40	OE2	GLU	302	C	---	CA	GLY	717	B	3.4
41	OE1	GLU	302	C	---	CA	GLY	717	B	3.2
42	CD	GLU	302	C	---	CA	GLY	717	B	3.6
43	CD	GLU	302	C	---	N	GLY	717	B	3.8

a

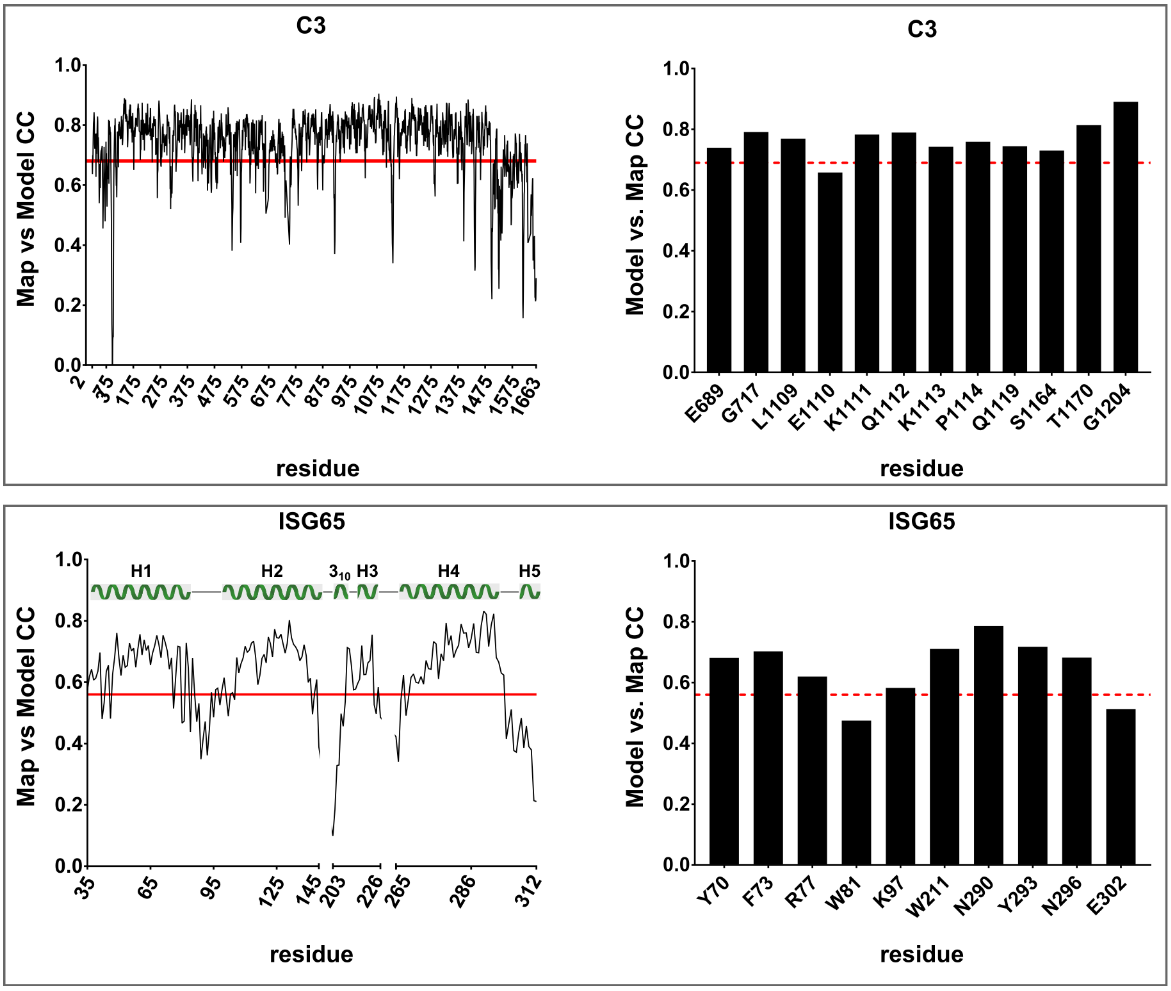

C3
Map vs Model CC
residue
C3
Model vs. Map CC
E689
G717
L1109
E1110
K1111
Q1112
K1113
P1114
Q1119
S1164
T1170
G1204
residue
ISG65
H1
H2
3_{10} H3
H4
H5
Map vs Model CC
residue
ISG65
Model vs. Map CC
Y70
F73
R77
W81
K97
W211
N290
Y293
N296
E302
residue

b

C3d (ISG65 interface)

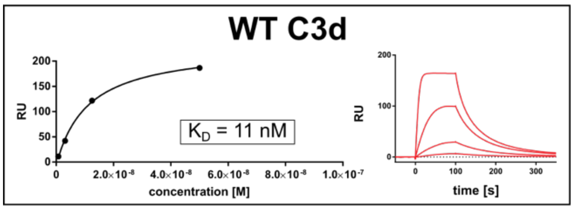

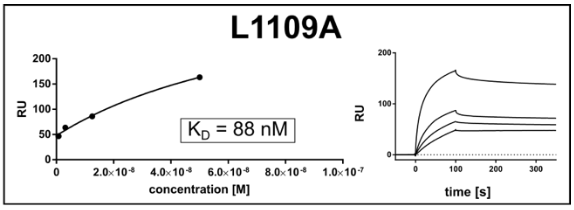

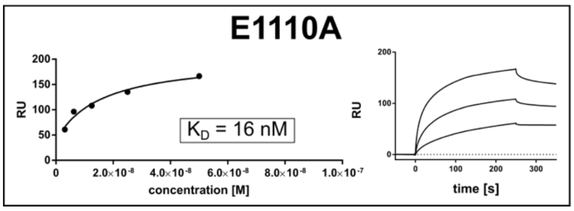

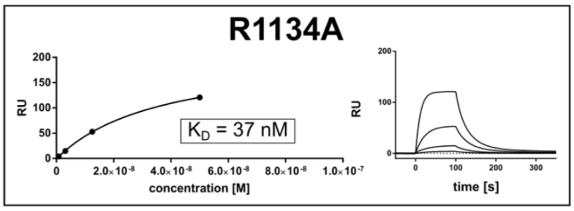

ISG65 (TED interface)

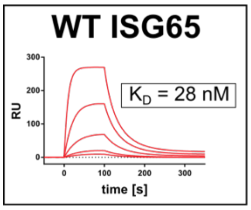

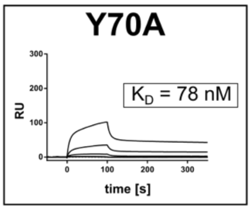

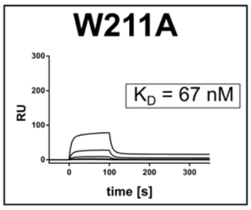

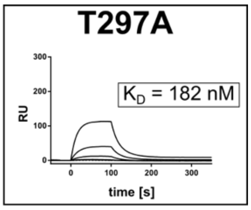

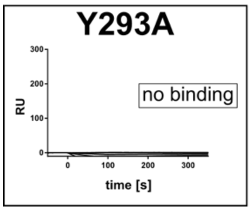

ISG65 (ANA interface)

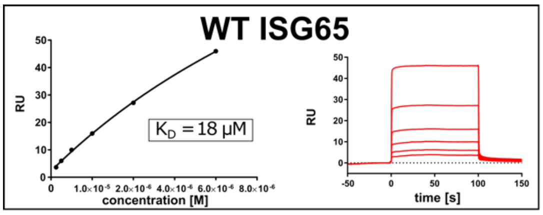

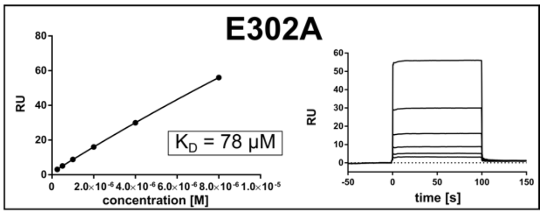

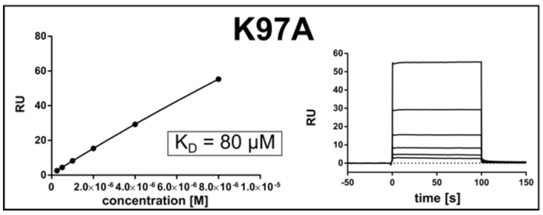

c

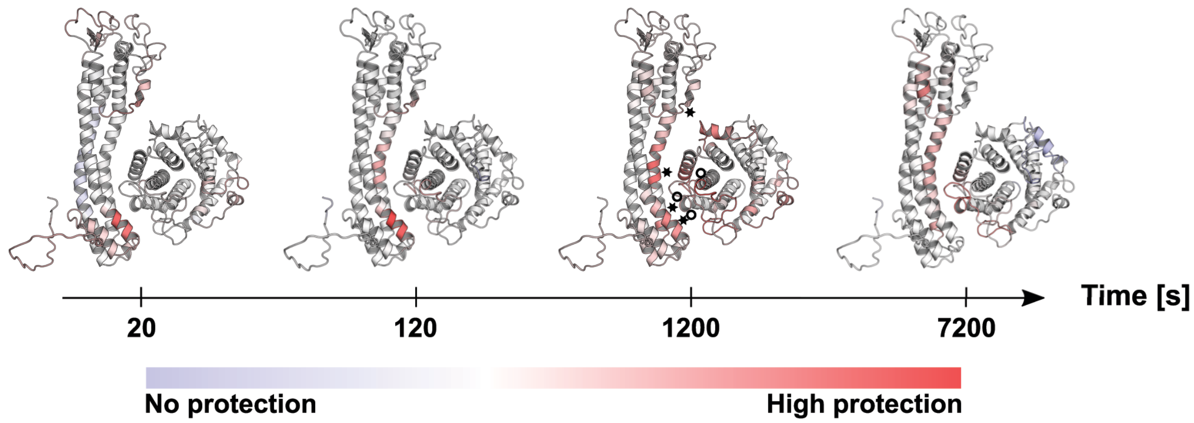

d

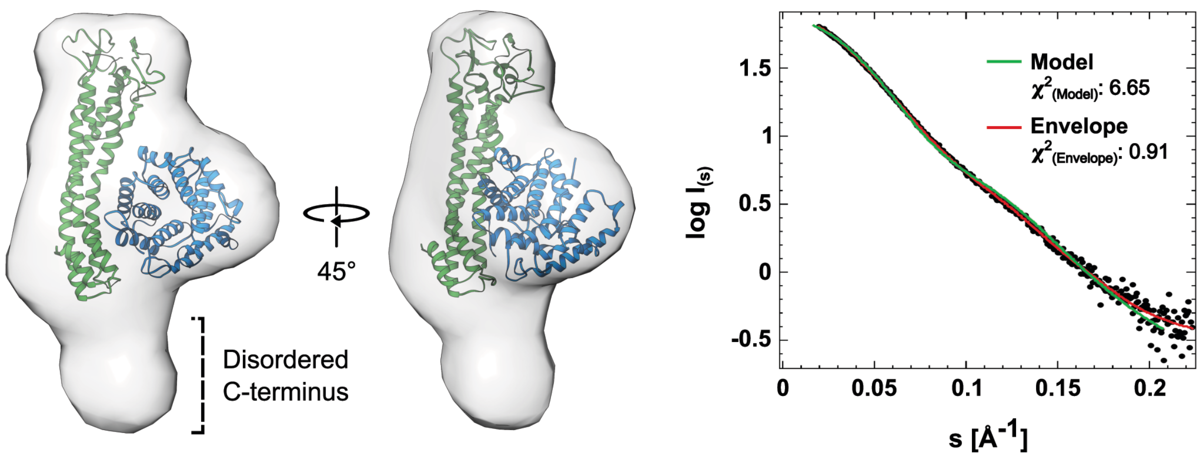

Supplementary Fig. 11: Validation of the interaction interface. a Map vs Model cross-correlation (CC) of the ISG65:C3 complex. CC has been calculated using the sharpened electron-density map in the cryo-EM validation tool in Phenix[8]. (Upper, left) Map vs Model CC plotted for each residue of the C3 model. Average cross-correlation over the whole molecule is indicated in red. (Upper, right) Map vs Model CC for individual residues in the interaction interface (see Supplementary Table 2). The average CC is indicated with a dashed, red line. (Lower, left) Map vs Model CC plotted for each residue of the ISG65 model. Average cross-correlation over the whole molecule is indicated in red. The structured, helical regions of ISG65 are indicated with green helices, unstructured regions are depicted as a black line. Large dips in the Map vs. Model CC can be observed towards the beginning and the end of unstructured regions such as the membrane distal, disordered head domain. (Lower, right) Map vs Model CC for individual residues in the interaction interface (see Supplementary Table 2). The average CC is indicated with a dashed, red line. **b** Effect of point mutations across the ISG65-TED interface on C3d (upper panels) and ISG65 (middle panels) as well as in the ISG65-ANA interface (lower panels). For each mutated residue, the blank-subtracted SPR sensorgrams are shown (right) alongside the steady-state fit if steady state analysis was used for K_D determination. In each case the K_D is displayed next to the type of analysis that was used. The binding kinetics of wt ISG65 to C3d and of wt C3d to ISG65 are represented by sensorgrams shown in red. Source data are provided as a Source Data file. **c** Deuteration levels, as measured by HDX-MS, mapped onto the interacting domains of the ISG65:C3 model. Colour gradient indicates the relative deuteration level at each point in time. Areas of low deuteration, thus protected areas, are highlighted in red, areas of high deuteration, thus low protection areas, are highlighted in blue. Especially at 1200s of the deuteration reaction, the area of low deuteration/high protection coincides well with the interaction interface as determined by cryo-EM. At 1200s, residues used in the point mutation analysis are highlighted with black stars (ISG65) and circles (C3d). For peptide coverage and deuterium uptake plots see 'Supplementary Data 2'. **d** SAXS analysis of the ISG65:C3d complex. (left) ISG65:C3d model docked

into the SAXS envelope of $ISG65_{18-363}$:C3d, calculated using DAMMIF[9]. ISG65 is shown in green, C3d in blue. The density of the envelope not explained by the model is likely caused by the disordered C-terminus of ISG65. This fact might also account for the slightly higher chi^2 fit of the model. (right) 1D scattering curve used for the calculation of the SAXS envelope. Fit of the envelope (red) and the model (green) to the experimental data is shown alongside the chi^2 of the fit.

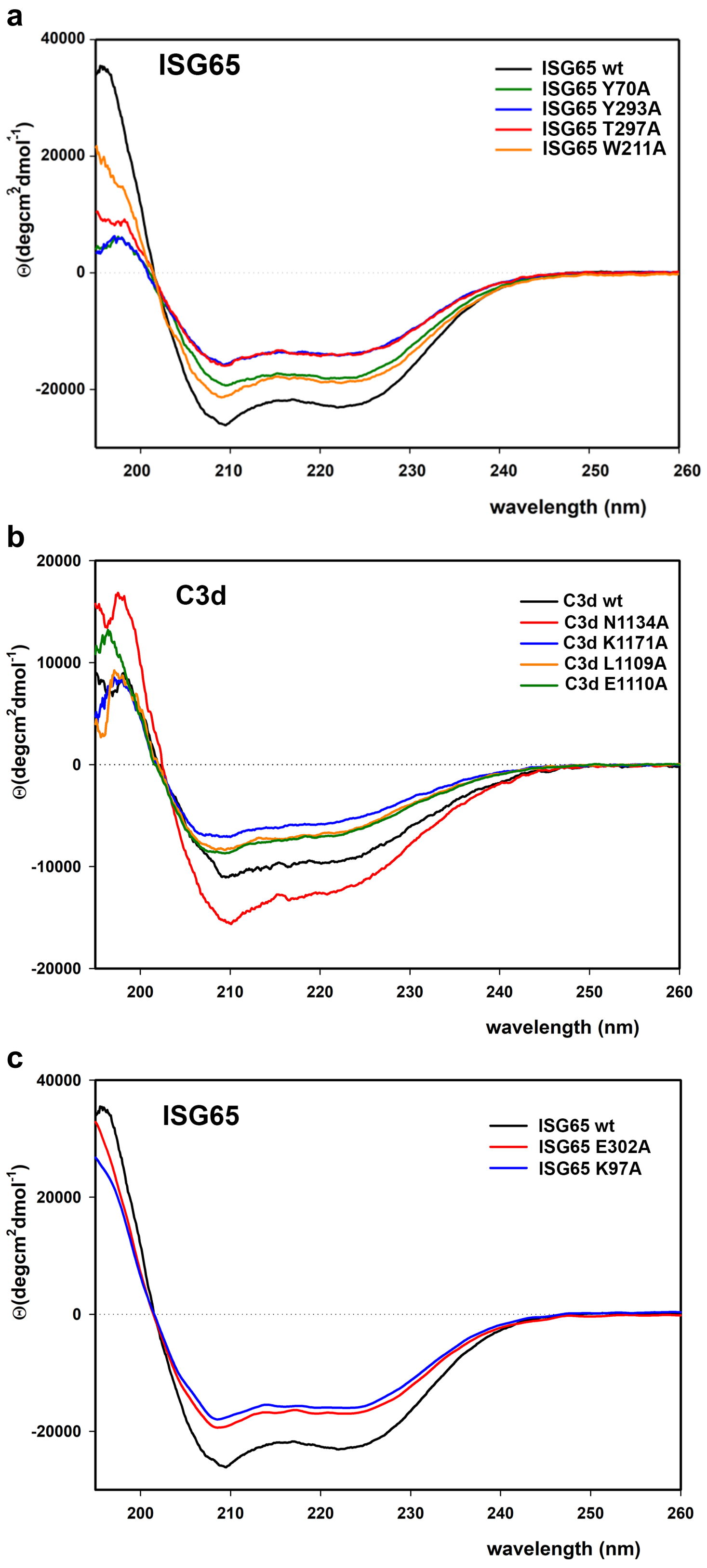

Supplementary Fig. 12: Secondary structure analysis of ISG65 and C3d mutants. Circular dichroism spectra for both wt and mutants of **a** ISG65, TED interface **b** C3d, ISG65 interface **c** ISG65 ANA interface. All proteins show minima at 208 and 222 nm characteristic for folded, alpha-helical proteins.

Supplementary Table 3: Binding affinities between ISG65 and complement factors measured by surface plasmon resonance.

All data interaction parameters were calculated using the BIAcore T200 evaluation software. For each interaction, the method of K_D calculation is indicated. NB - no binding.

Ligand	Analyte	Mutation	k_{on1} ($M^{-1}s^{-1}$)	k_{on2} ($M^{-1}s^{-1}$)	k_{off1} (s^{-1})	k_{off2} (s^{-1})	K_D (nM)	C3d binding (%)	Method of K_D calculation
**Binding impaired mutants**									
C3d-Avi	ISG65	Wt	1003000	0.90	0.04	0.002	27.9	100	2-state
		Y70A	118000	0.008	0.14	0.58	78.2	36	2-state
		W211A	320000	0.003	0.16	0.39	66.8	42	2-state
		Y293A	-	-	-	-	NB	NB	-
		T297A	171000	0.001	0.05	0.002	181.8	15	2-state
ISG65-Avi	C3d							**ISG65 binding (%)**	
		Wt	5241000	-	0.03785	-	11	100	Steady state
		L1109A	-	-	-	-	88	8	Steady state
		E1110A	-	-	-	-	16	69	Steady state
		R1134A	1893000	-	0.05419	-	37	30	Steady state
ISG65	C3a	Wt	-	-	-	-	18 100	100	Steady state
		E302A	-	-	-	-	78 000	23	Steady state
		K97A	-	-	-	-	80 000	23	Steady state
**C3 fragments**									
ISG65-Avi	C3MA		941500	-	0.01698	-	18.0	-	Langmuir
	C3b		262200	-	0.02120	-	80.9	-	Langmuir
	C3		81840	0.002427	0.05433	0.0005864	129.2	-	2-state
	C3c		-	-	-	-	NB	-	-

Supplementary Table 4: Reporting summary for SAS data acquisition, sample details, data analysis, modelling fitting and software used.

**(a) Sample details**		
	**ISG65**	**ISG65:C3d**
Organism	*Trypansoma brucei gambiense*	*Trypansoma brucei gambiense: Homo sapiens*
Source (Catalogue No. or reference)	*E. coli* T7 Shuffle<br>NEB (C3026J)<br>C9ZJ67 (18-363)	*E. coli* T7 Shuffle<br>NEB (C3026J)<br>C9ZJ67 (18-363): V9HWA9 (996-1287)
Extinction coefficient ε (wavelength and units)	1.228	1.305
Partial specific volume $\bar{\upsilon}$ (cm^3 g^{-1})	0.743	0.743
Mean solute and solvent scattering length densities and mean scattering contrast $\Delta\bar{\rho}$ (cm^{-2})	2.81	2.81
Molecular mass *M* from chemical composition (Da)	40800	76000
For SEC-SAS, loading volume/concentration, (mg ml^{-1}) injection volume (µl), flow rate (ml min^{-1})	6.2 mg ml^{-1}, 50 µl, 0.08 ml min^{-1}	15.0 mg ml^{-1}, 50 µl, 0.08 ml min^{-1}
Concentration (range/values) measured and method		
Solvent composition and source	20 mM HEPES, 150 mM NaCl, 3% (v/v) glycerol, pH 7.5	20 mM HEPES, 150 mM NaCl, 3% (v/v) glycerol, pH 7.5

**(b) SAS data collection parameters**	
Source, instrument and description or reference	BM29 beam line at the ESRF (Grenoble, France) with Pilatus3 2M detector in vacuum
Wavelength (Å)	0.99
Beam geometry (size, sample-to-detector distance)	200 x 200 µm , 2.867 m
q-measurement range (Å^{-1})	0.0025 – 0.6 Å^{-1}
Absolute scaling method	n/a (no absolute determination of I(0))
Basis for normalization to constant counts	The data were normalized to the intensity of the transmitted beam and radially averaged
Method for monitoring radiation damage, X-ray dose	n/a
Exposure time, number of exposures	1200 successive 1 second frames of SEC elution
Sample configuration including path length and flow rate where relevant	Quartz glass capillary, 1 mm diameter<br>0.08 ml min^{-1}
Sample temperature (ºC)	20

(c) Software employed for SAS data reduction, analysis and interpretation

SAS data reduction: Primus[10] and Chromxis[11] from ATSAS 3.2.1

Calculation of ε from sequence: ProtParam[12]

Calculation of $\Delta\bar{\rho}$ and $\bar{\upsilon}$ values from chemical composition: Primus from ATSAS 3.2.1

Basic analyses: Guinier, *P*(*r*), V_P: Primus from ATSAS 3.2.1 and BioXTAS RAW[13]

Shape/bead modelling: DAMMIF[9] via ATSAS online

Atomic structure modelling (ensemble): EOM 2.0[14,15] via ATSAS online

Modelling of missing sequence from PDB files: AlphaFold2[7] via ColabFold[16]

Molecular graphics: PyMOL v2.4.2 MacOS[6]

**(d) Structural parameters**		
Guinier Analysis	**ISG65**	**ISG65:C3d**
I(0) (arbitrary)	33.46 +/- 0.09	76.88 +/- 0.1
R_g (Å)	35.8 +/- 0.2	37.0 +/- 9.97e-2
q-range ($Å^{-1}$)	0.009-0.036	0.009-0.036
Quality-of-fit parameter (r^2)	0.982	0.989
M from *I*(0) (ratio to expected value)	n/a	n/a
P(*r*) analysis	ISG65	ISG65: C3d
I(0) (arbitrary)	32.66 +/- 0.13	75.17 +/- 0.19
R_g (Å)	35.6 +/- 0.2	36.8 +/- 0.1
d_{max} (Å)	124.5	128
q-range ($Å^{-1}$)	0.018 to 0.234	0.020 to 0.224
Quality-of-fit parameter (chi^2)	1.1368	0.8715
M from Bayesian inference (Da)	36900	74325
Porod Volume ($Å^3$)	70475	103461
**(e) Shape modelling results**		
DAMMIF (default parameters, 20 calculations)	**-**	**ISG65:C3d**
q-range for fitting ($Å^{-1}$)		0.02-0.223
Symmetry/anisotropy assumptions		P1, none
Ambiguity measure(s) (*AMBIMETER score*)		2.338
Quality-of-fit parameters envelope/model (chi^2)		0.91/6.65
Adjustable parameters in the model fit		n/a
Model volume ($Å^3$)		143000
Model resolution (Å)		46 +/- 3
For multiple phase shape models, R_g values and relative phase volumes		n/a
**(f) Atomistic modelling**		
EOM 2.0 (default parameters, 10 000 models)	**ISG65**	**-**
q-range for fitting ($Å^{-1}$)	0.0092 to 0.49	
Symmetry assumptions	P1	
Constant subtraction	0.072	
Any measures of model precision	n/a	
Chi^2 value (ensemble)	1.147	
Rflex random/ensemble	90.5%/79.3%	
Adjustable parameters in the model fit	n/a	
Relevant output parameters (ensemble R_g/D_{max})	35.1/115.57	
Number of representative structures	6	
Regions of presumed flexibility (residues)	318-363	
**(g) Data and model deposition IDs**		
	**ISG65**	**ISG65:C3d**
	SASDP99	SASDPA9

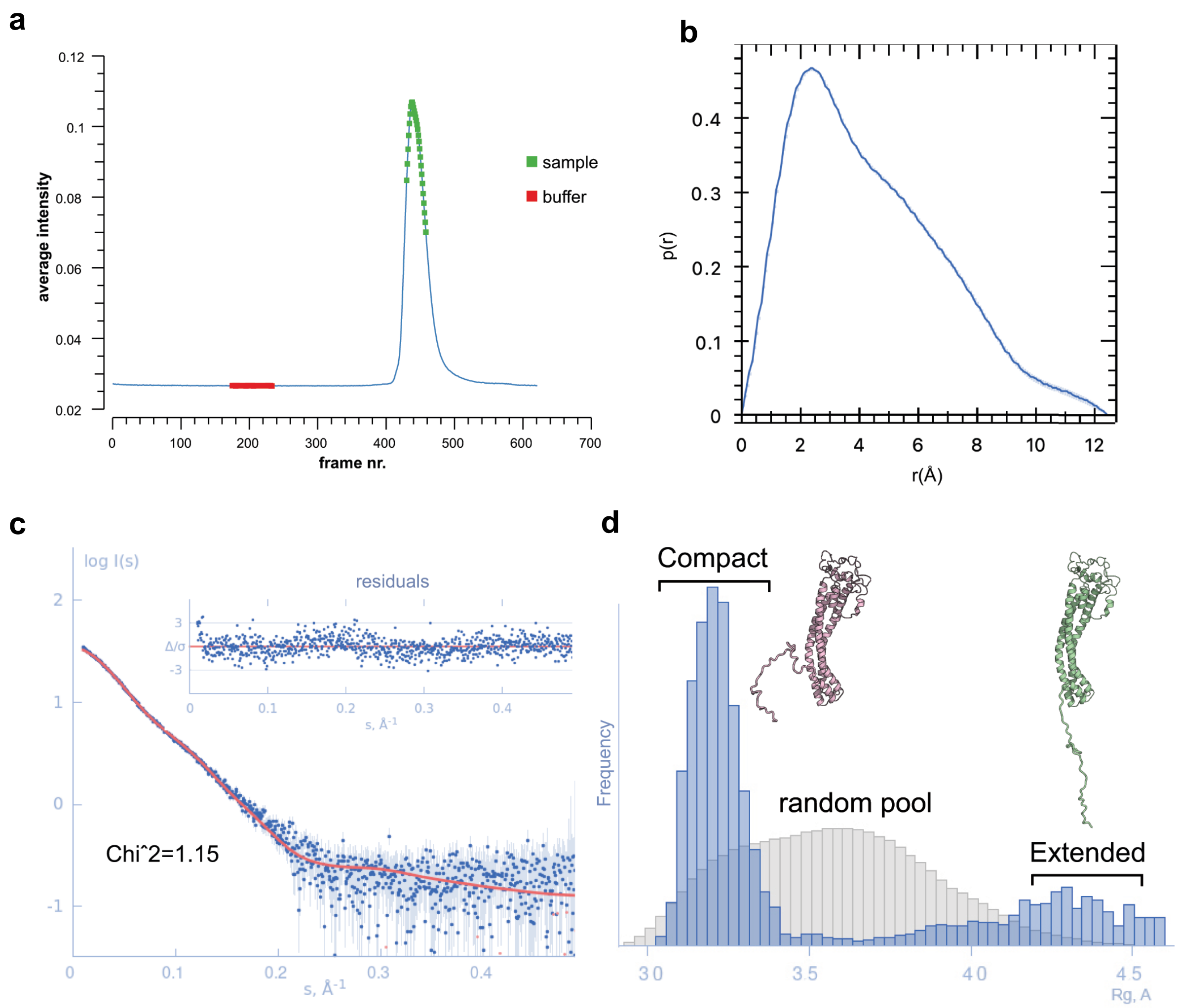

e

Ensemble of models			
	R_g (Å)	D_{max} (Å)	Fraction
Model 1	31.81	100.73	0.17
Model 2	32.71	100.73	0.17
Model 3	35.04	114.85	0.17
Model 4	33.50	103.5	0.17
Model 5	45.84	172.89	0.17
Model 6	31.69	100.73	0.17

Supplementary Fig. 13: SAXS data collection and flexibility analysis. Scattering data was collected in SEC-SAXS mode and processed using the ATSAS 3.0 software package[10]. The conformational states of the disordered C-terminal linker (aa 317-363) of ISG65 were calculated using EOM 2.0[14,15]. **a** SEC-SAXS trace of ISG65**.** Selected frames are marked (buffer, red; protein, green). **b** Pair-distance distribution function of ISG65. The maximum dimension was determined to be 124.5 Å. **c** Theoretical scattering curve of the selected ensemble (red line) superimposed on the experimental scattering data (blue dots). Error bars are shown as light blue, vertical lines. The residual plot is shown as inset. I, Intensity; s, scattering vector **d** R_g distribution of models in the

random pool (grey) and selected subsets (blue) in compact or extended conformation that best fit the experimental data. The hight of the bars corresponds to the number of models in each subset. 6 models representative for the most populated subsets were created (see table). One model of each subset is shown next to its corresponding pool (pink, compact; green, extended). Overall, the selected ensemble is less flexible than the random pool, with compact states being more frequent than extended states. **e** Modelling summary of the chosen ensemble. Models representative for compact and extended states are highlighted in yellow.

Supplementary References

1 Perez-Riverol, Y. *et al.* The PRIDE database resources in 2022: a hub for mass spectrometry-based proteomics evidences. *Nucleic Acids Res* **50**, D543-D552, doi:10.1093/nar/gkab1038 (2022).

2 Field, M. C. & Carrington, M. The trypanosome flagellar pocket. *Nat Rev Microbiol* **7**, 775-786, doi:10.1038/nrmicro2221 (2009).

3 Menny, A. *et al.* CryoEM reveals how the complement membrane attack complex ruptures lipid bilayers. *Nat Commun* **9**, 5316, doi:10.1038/s41467-018-07653-5 (2018).

4 Rooijakkers, S. H. *et al.* Structural and functional implications of the alternative complement pathway C3 convertase stabilized by a staphylococcal inhibitor. *Nat Immunol* **10**, 721-727, doi:10.1038/ni.1756 (2009).

5 Strohalm, M., Kavan, D., Novak, P., Volny, M. & Havlicek, V. mMass 3: a cross-platform software environment for precise analysis of mass spectrometric data. *Anal Chem* **82**, 4648-4651, doi:10.1021/ac100818g (2010).

6 The PyMOL Molecular Graphics System, Version 2.5 Schrödinger, LLC.

7 Jumper, J. *et al.* Highly accurate protein structure prediction with AlphaFold. *Nature* **596**, 583-589, doi:10.1038/s41586-021-03819-2 (2021).

8 Liebschner, D. *et al.* Macromolecular structure determination using X-rays, neutrons and electrons: recent developments in Phenix. *Acta Crystallogr D Struct Biol* **75**, 861-877, doi:10.1107/S2059798319011471 (2019).

9 Franke, D. & Svergun, D. I. DAMMIF, a program for rapid ab-initio shape determination in small-angle scattering. *J Appl Crystallogr* **42**, 342-346, doi:10.1107/S0021889809000338 (2009).

10 Manalastas-Cantos, K. *et al.* ATSAS 3.0: expanded functionality and new tools for small-angle scattering data analysis. *J Appl Crystallogr* **54**, 343-355, doi:10.1107/S1600576720013412 (2021).

11 Panjkovich, A. & Svergun, D. I. CHROMIXS: automatic and interactive analysis of chromatography-coupled small-angle X-ray scattering data. *Bioinformatics* **34**, 1944-1946, doi:10.1093/bioinformatics/btx846 (2018).

12 Wilkins, M. R. *et al.* Protein identification and analysis tools in the ExPASy server. *Methods Mol Biol* **112**, 531-552, doi:10.1385/1-59259-584-7:531 (1999).

13 Hopkins, J. B., Gillilan, R. E. & Skou, S. BioXTAS RAW: improvements to a free open-source program for small-angle X-ray scattering data reduction and analysis. *J Appl Crystallogr* **50**, 1545-1553, doi:10.1107/S1600576717011438 (2017).

14 Tria, G., Mertens, H. D., Kachala, M. & Svergun, D. I. Advanced ensemble modelling of flexible macromolecules using X-ray solution scattering. *IUCrJ* **2**, 207-217, doi:10.1107/S205225251500202X (2015).

15 Bernado, P., Mylonas, E., Petoukhov, M. V., Blackledge, M. & Svergun, D. I. Structural characterization of flexible proteins using small-angle X-ray scattering. *J Am Chem Soc* **129**, 5656-5664, doi:10.1021/ja069124n (2007).

16 Mirdita, M. *et al.* ColabFold: making protein folding accessible to all. *Nat Methods* **19**, 679-682, doi:10.1038/s41592-022-01488-1 (2022).
