# Supplementary material for: Cryo-EM structures of Trypanosoma brucei gambiense ISG65 with human complement C3 and C3b and their roles in alternative pathway restriction
Source: Nat Commun. 2023 Apr 27;14:2403. doi: 10.1038/s41467-023-37988-7 (PMC10140031; doi:10.1038/s41467-023-37988-7)
Supplement: Supplementary file 3 — Description of Additional Supplementary Files [file 41467_2023_37988_MOESM3_ESM.docx]

File Name: Supplementary Data 1

Description: Complete flow cytometry traces for *T.b. gambiense* cells, incubated with AF594 labelled

human transferrin or AF594 labelled human in the presence or absence of ISG65 and ISG75.

File Name: Supplementary Data 2
Description: HDX-MS analysis summary, peptide mapping, deuterium uptake heat maps and plots for the ISG65:C3d complex.
