# Supplementary material for: Cryo-EM structures of Trypanosoma brucei gambiense ISG65 with human complement C3 and C3b and their roles in alternative pathway restriction
Source: Nat Commun. 2023 Apr 27;14:2403. doi: 10.1038/s41467-023-37988-7 (PMC10140031; doi:10.1038/s41467-023-37988-7)
Supplement: Supplementary file 4 — Supplementary Data 1 [file 41467_2023_37988_MOESM4_ESM.pdf]

Complete flow cytometry traces. *T.b. gambiense* cells were incubated with AF594 labelled human transferrin or AF594 labelled human C3b for 0 min at 4°C (surface binding conditions; see Methods part), for 2 min or 5 min at 37°C, respectively, in the presence or absence of ISG65 and ISG75. Alternatively, non-labelled C3b was added at 4-fold molar excess. Cells were then fixed and subjected to flow cytometry using an excitation wavelength of 560 nm in combination with a PE-Texas Red filter. Non-treated control cells served to determine the gate for AF594 positive cells. Each page shows traces for 1 sample: an FSC-A/SSC-A plot, an FSC-A/FSC-H plot and an PE-Texas Red-A/FSC-A plot with applied gates indicated. Histogram plots of cell count versus fluorescence intensity (Count/PE-Texas Red-A) and a table summarizing the distribution of events for all gates are shown at the bottom.

Sample layout:

**Sample / 3 replicates each**	**Supplements for uptake assay**	**t /min**	**Pages**
Cells only-0min	-	0	2-4
Trans-0min	$Transferrin_{AF594}$	0	5-7
C3b-0min	$C3b_{AF594}$	0	8-10
C3b-ISG65-0min	$C3b_{AF594}$+ISG65	0	11-13
C3b-ISG75-0min	$C3b_{AF594}$+ISG75	0	14-16
Cells only-2min	-	2	17-19
Trans-2min	$Transferrin_{AF594}$	2	20-22
C3b-2min	$C3b_{AF594}$	2	23-25
C3b-ISG65-2min	$C3b_{AF594}$+ISG65	2	26-28
C3b-ISG75-2min	$C3b_{AF594}$+ISG75	2	29-31
Cells only-5min	-	5	32-34
Trans-5min	$Transferrin_{AF594}$	5	35-37
C3b-5min	$C3b_{AF594}$	5	38-40
C3b-ISG65-5min	$C3b_{AF594}$+ISG65	5	41-43
C3b-ISG75-5min	$C3b_{AF594}$+ISG75	5	44-46
C3bc3b-0min	$C3b_{AF594}$+C3b	0	47-49
C3bc3b-ISG65-0min	$C3b_{AF594}$+C3b +ISG65	0	50-52
C3bc3b-ISG75-0min	$C3b_{AF594}$+C3b +ISG75	0	53-55
C3bc3b-2min	$C3b_{AF594}$+C3b	2	56-58
C3bc3b-ISG65-2min	$C3b_{AF594}$+C3b +ISG65	2	59-61
C3bc3b-ISG75-2min	$C3b_{AF594}$+C3b +ISG75	2	62-64
C3bc3b-5min	$C3b_{AF594}$+C3b	5	65-67
C3bc3b-ISG65-5min	$C3b_{AF594}$+C3b +ISG65	5	68-70
C3bc3b-ISG75-5min	$C3b_{AF594}$+C3b +ISG75	5	71-73

BD FACSDiva 8.0.1

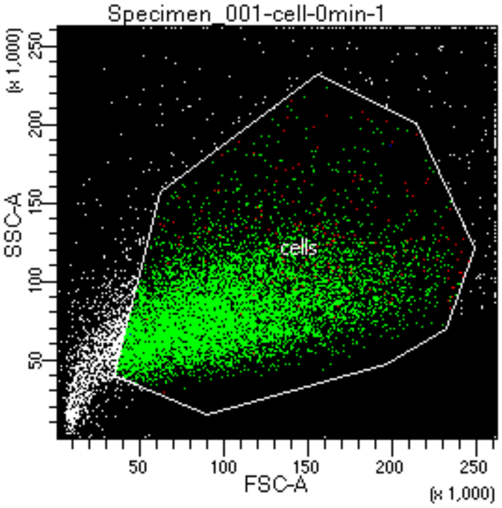

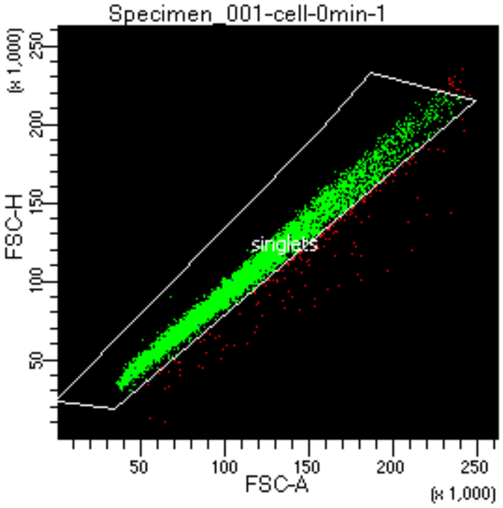

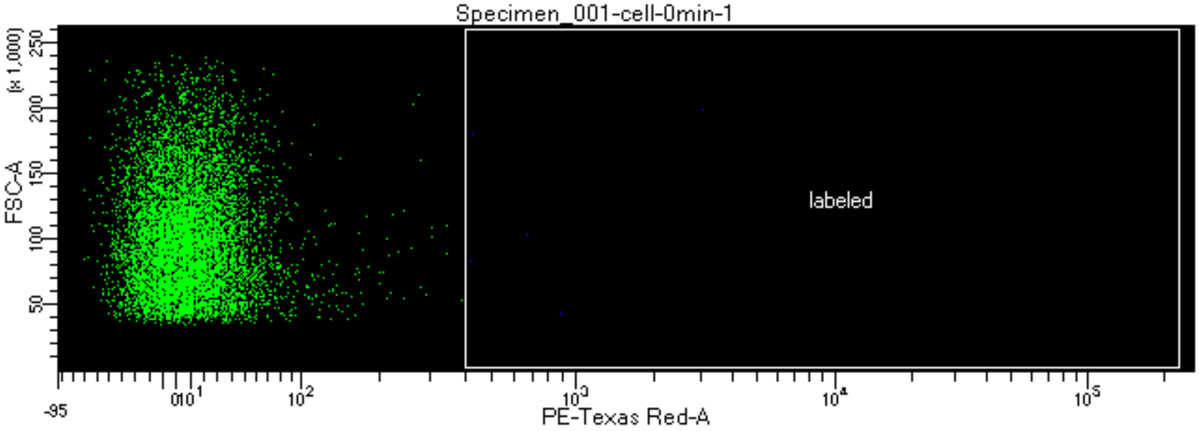

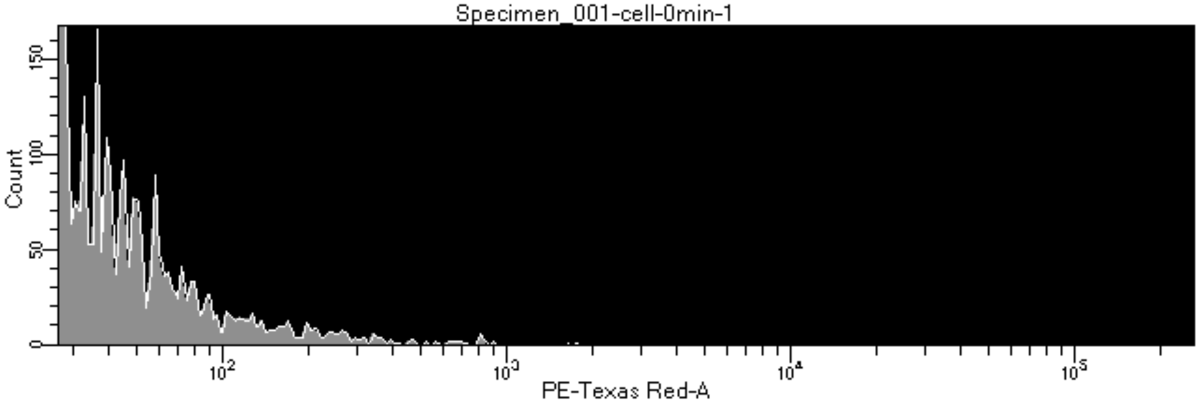

Tube: cell-0min-1

Population	#Events	%Parent	%Total
All Events	10,000	####	100.0
cells	8,351	83.5	83.5
singlets	8,161	97.7	81.6
labele	5	0.1	0.0

BD FACSDiva 8.0.1

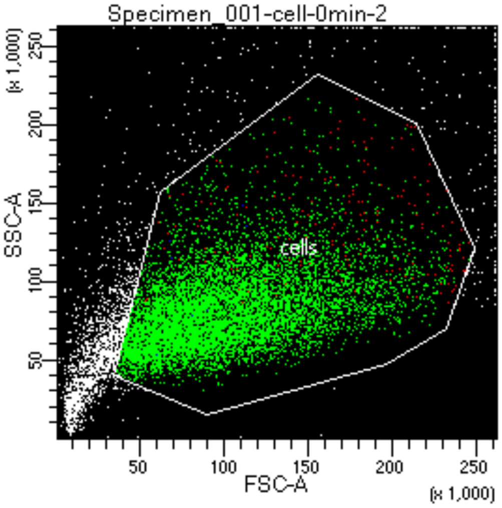

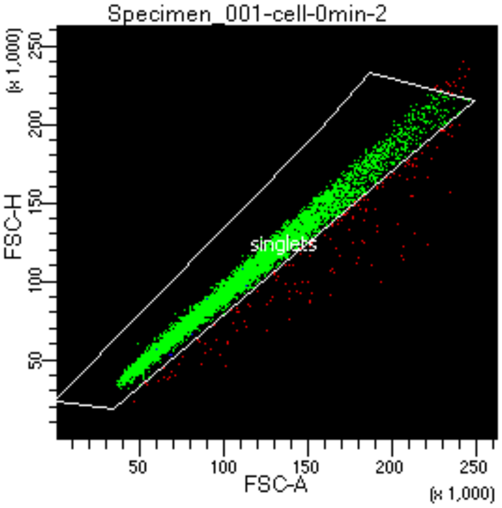

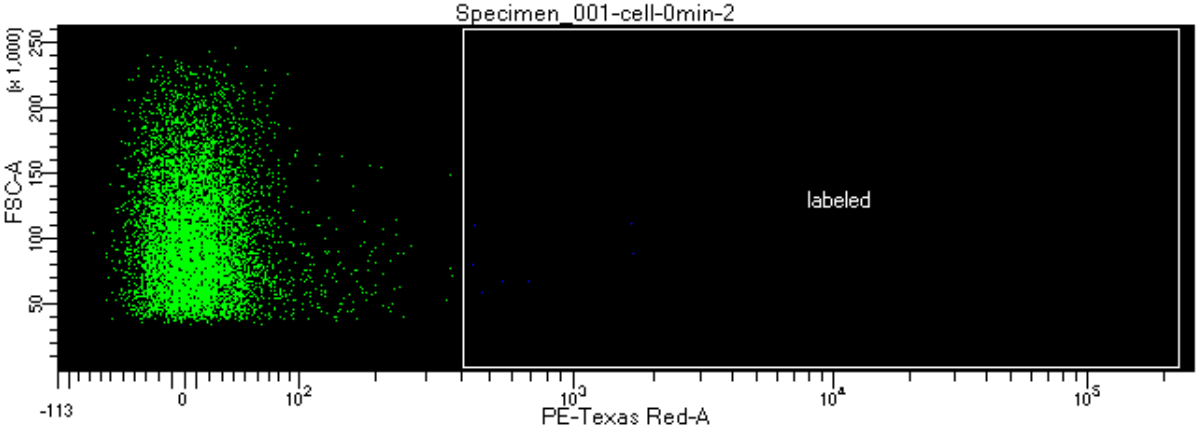

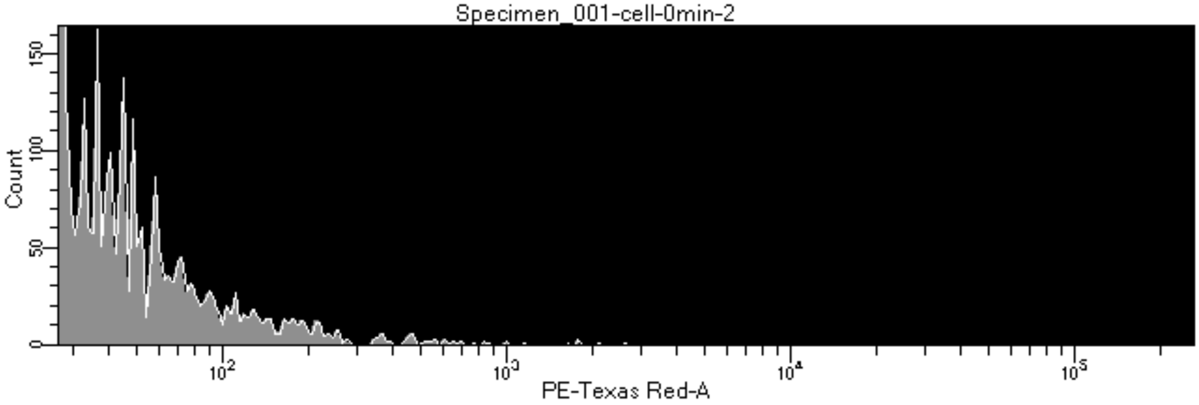

Tube: cell-0min-2

Population	#Events	%Parent	%Total
All Events	10,000	####	100.0
cells	8,072	80.7	80.7
singlets	7,889	97.7	78.9
label€	7	0.1	0.1

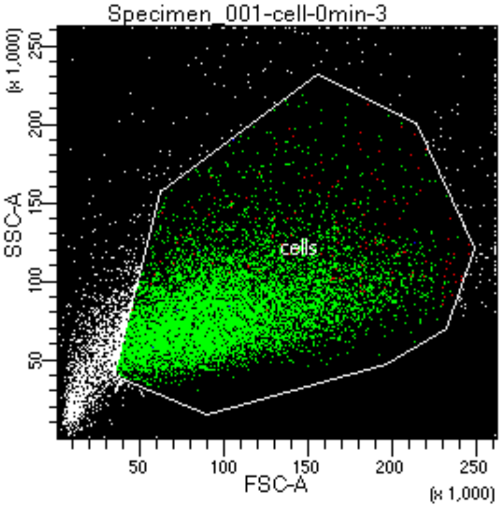

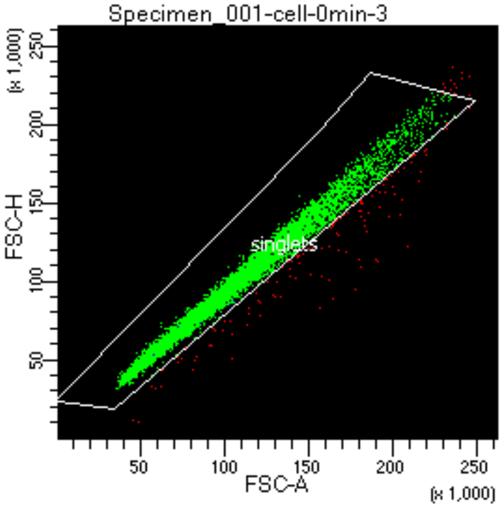

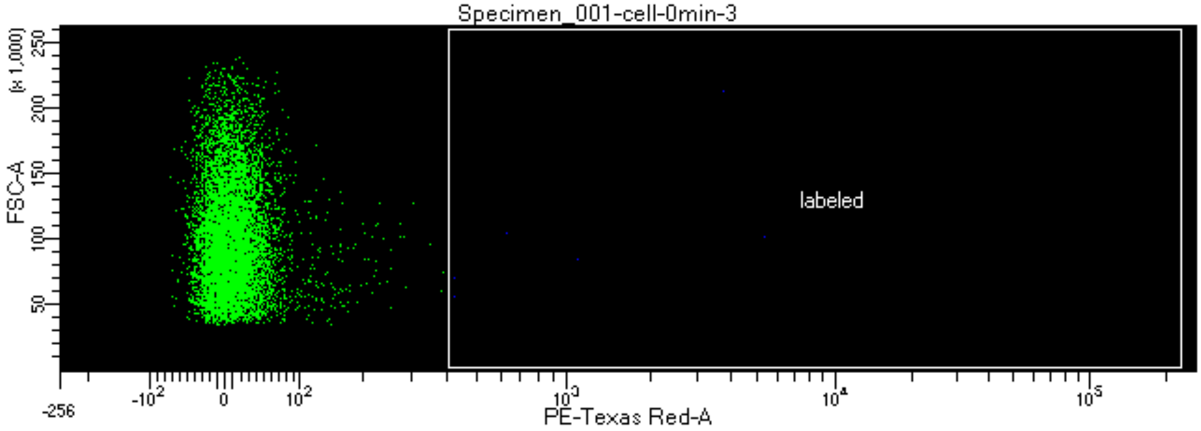

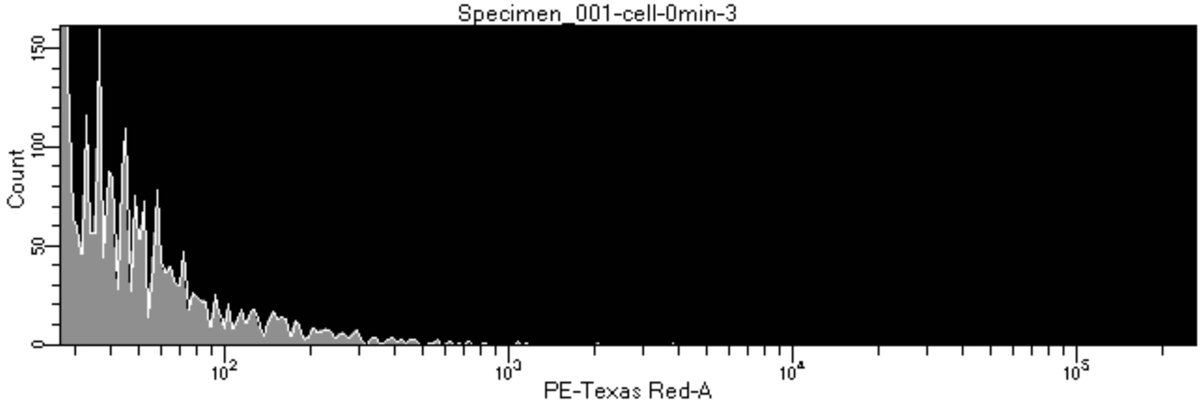

Tube: cell-0min-3

Population	#Events	%Parent	%Total
All Events	10,000	####	100.0
cells	8,047	80.5	80.5
singlets	7,902	98.2	79.0
label(	6	0.1	0.1

BD FACSDiva 8.0.1

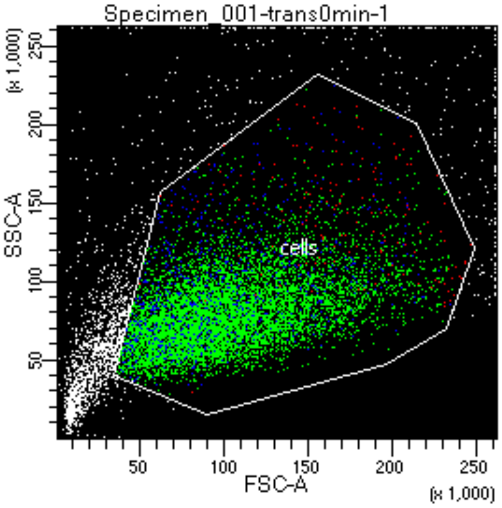

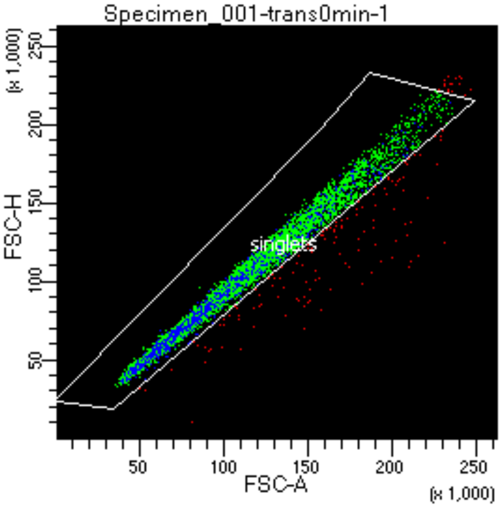

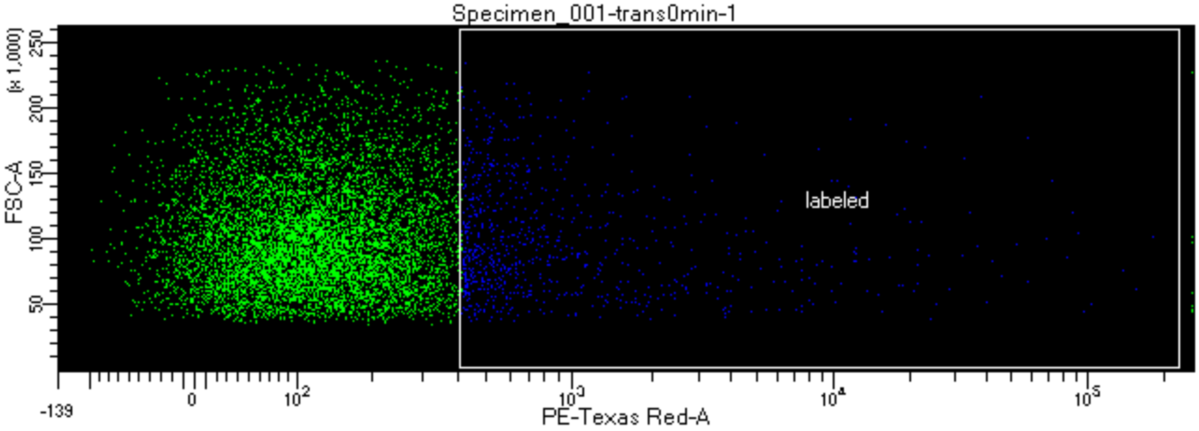

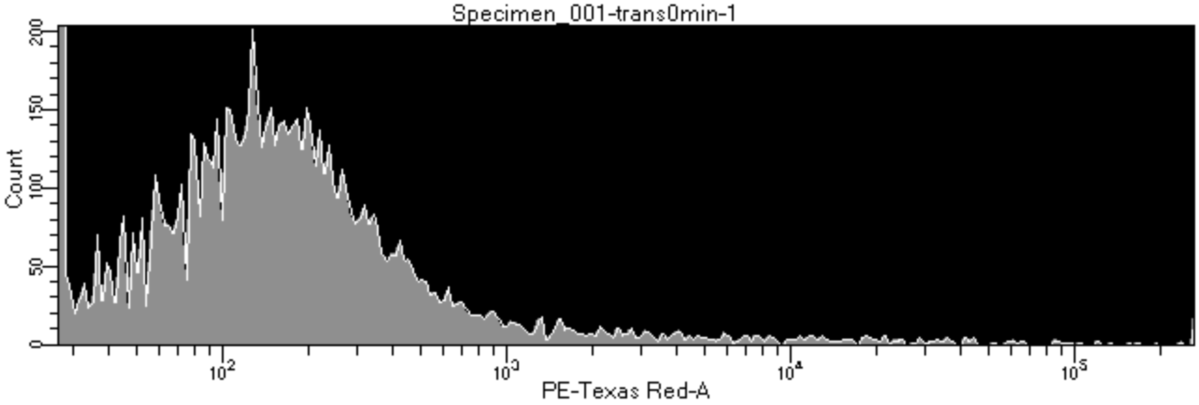

Tube: trans0min-1

Population	#Events	%Parent	%Total
All Events	10,000	####	100.0
cells	8,191	81.9	81.9
singlets	8,028	98.0	80.3
labele	799	10.0	8.0

BD FACSDiva 8.0.1

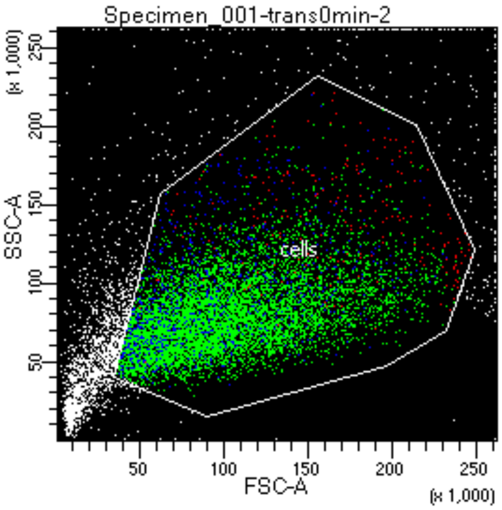

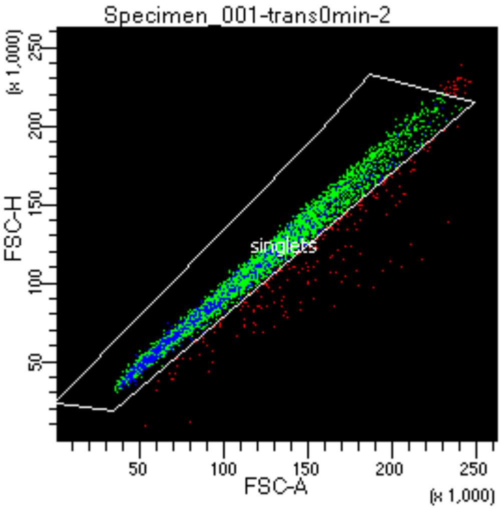

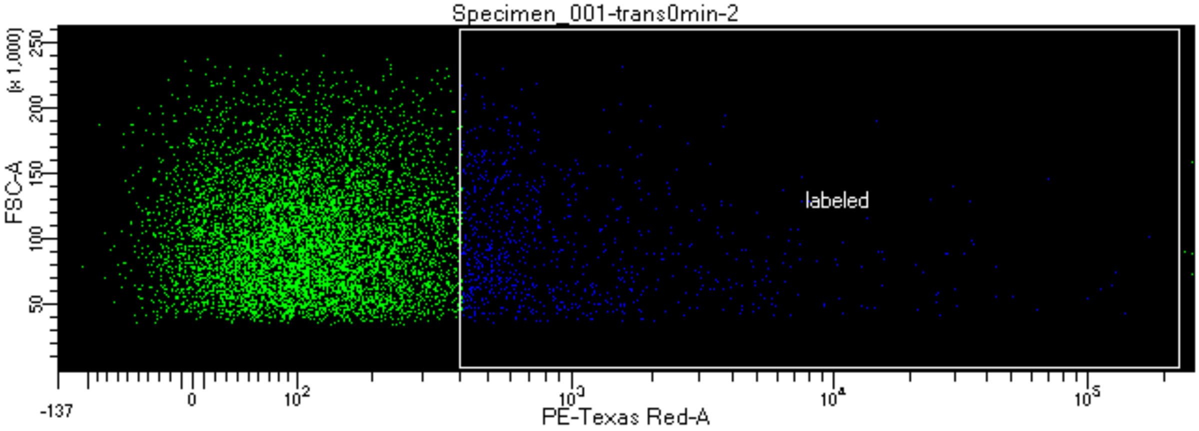

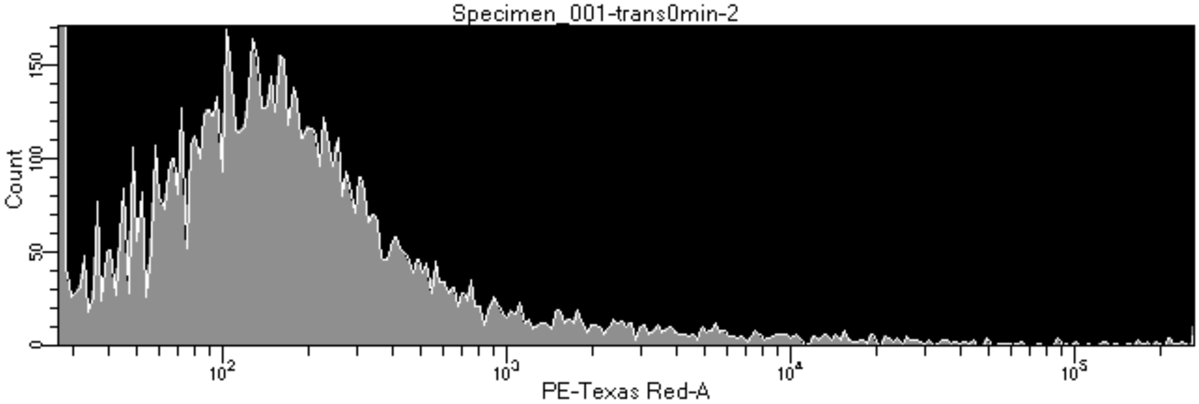

Tube: trans0min-2

Population	#Events	%Parent	%Total
All Events	10,000	####	100.0
cells	7,970	79.7	79.7
singlets	7,742	97.1	77.4
label	826	10.7	8.3

BD FACSDiva 8.0.1

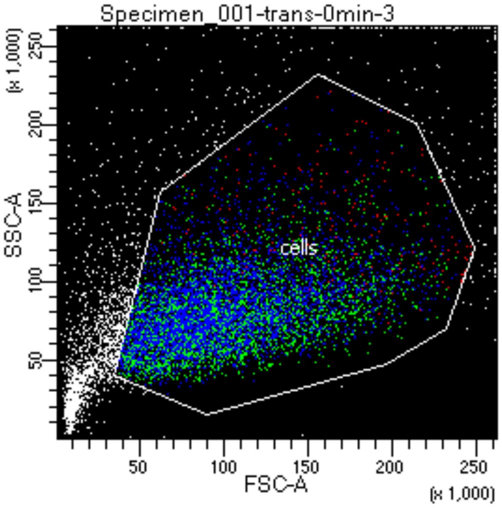

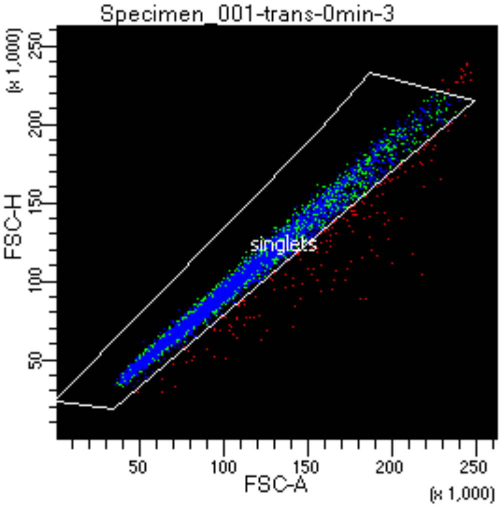

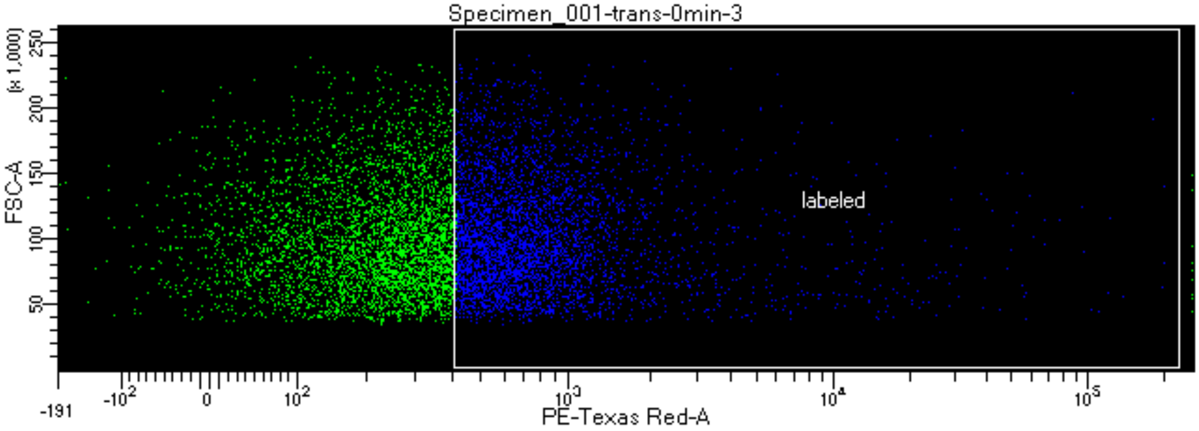

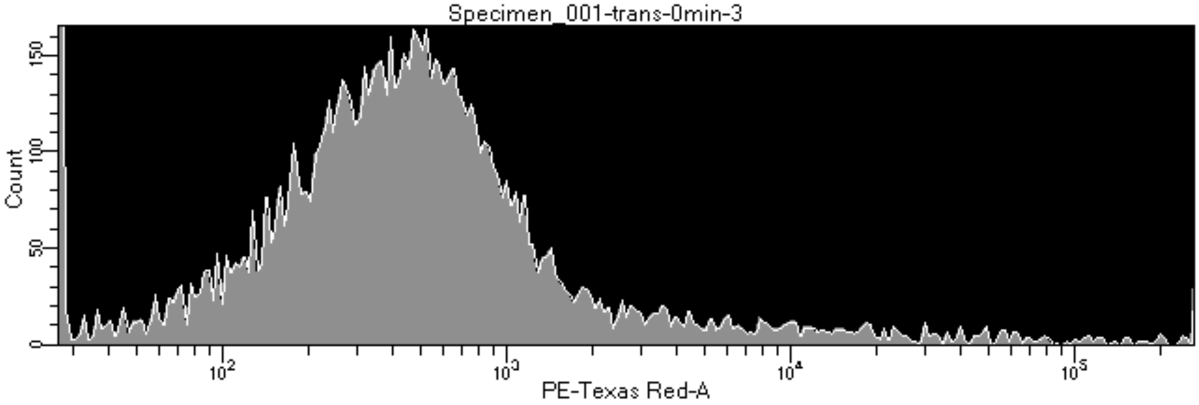

Tube: trans-0min-3

Population	#Events	%Parent	%Total
All Events	10,000	####	100.0
cells	8,141	81.4	81.4
singlets	7,953	97.7	79.5
labele	3,869	48.6	38.7

BD FACSDiva 8.0.1

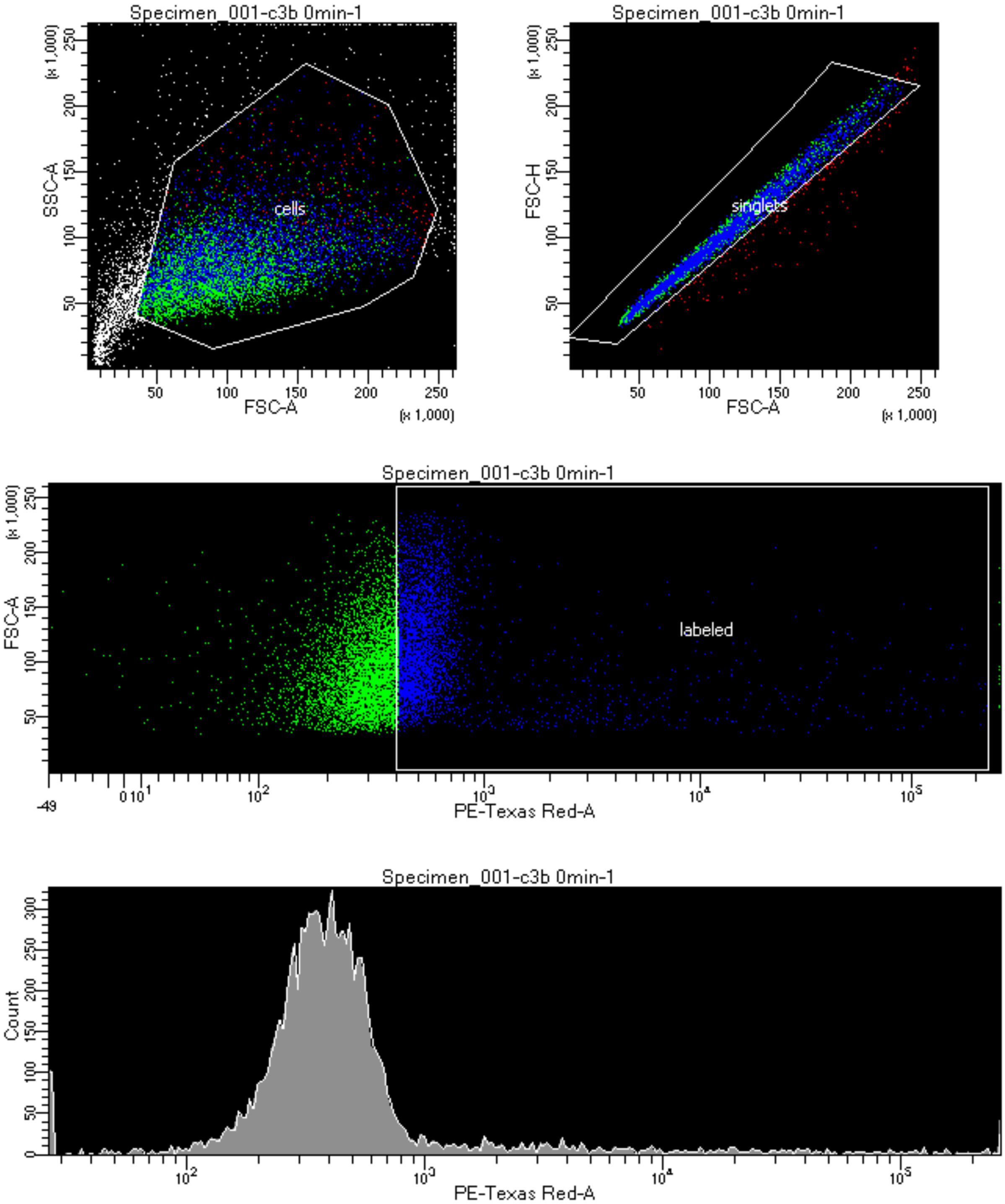

Tube: c3b 0min-1

Population	#Events	%Parent	%Total
All Events	10,000	####	100.0
cells	8,275	82.8	82.8
singlets	8,085	97.7	80.8
labele	3,392	42.0	33.9

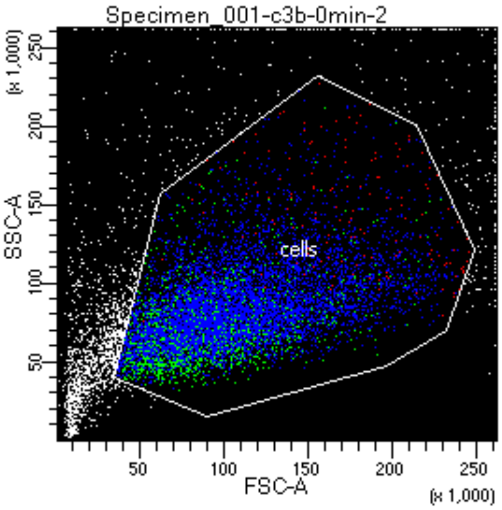

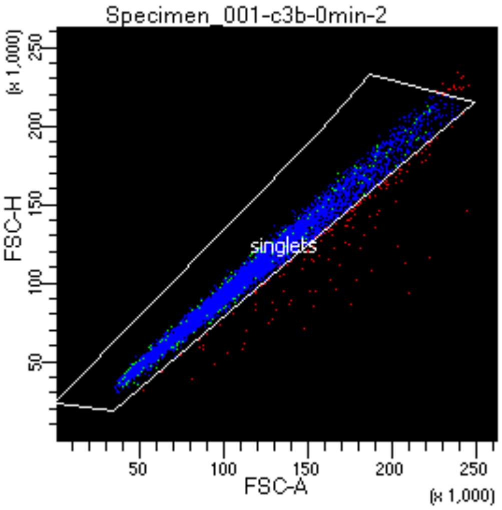

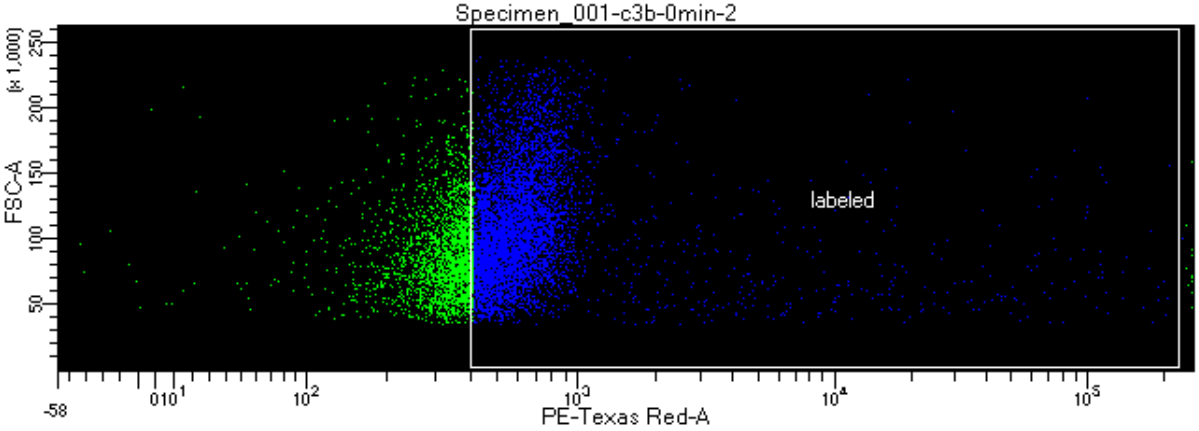

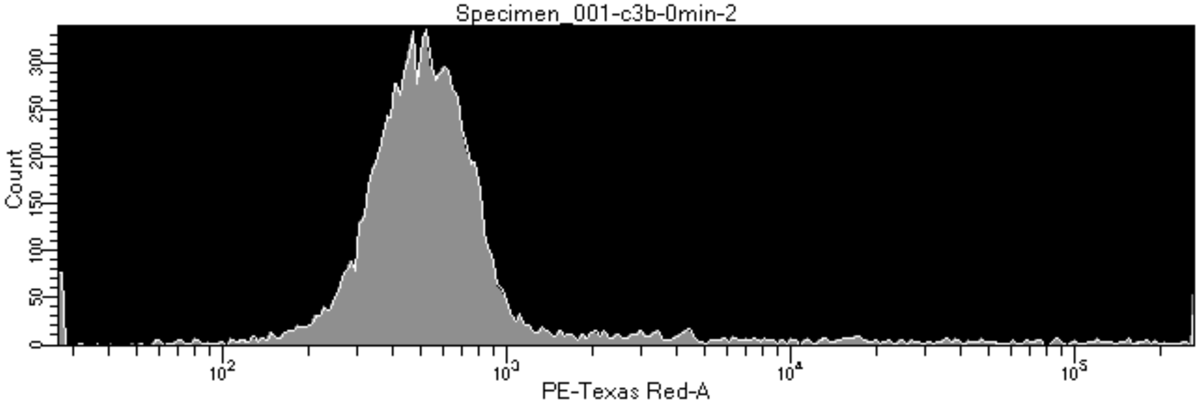

Tube: c3b-0min-2

Population	#Events	%Parent	%Total
All Events	10,000	####	100.0
cells	8,363	83.6	83.6
singlets	8,193	98.0	81.9
label‹	5,762	70.3	57.6

BD FACSDiva 8.0.1

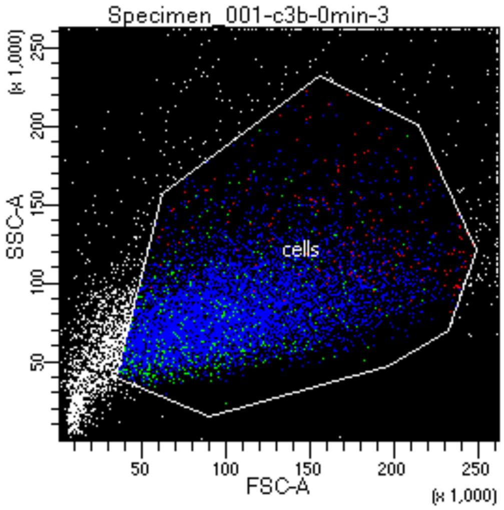

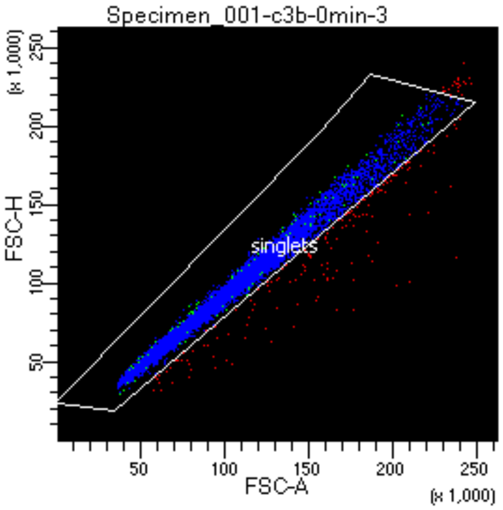

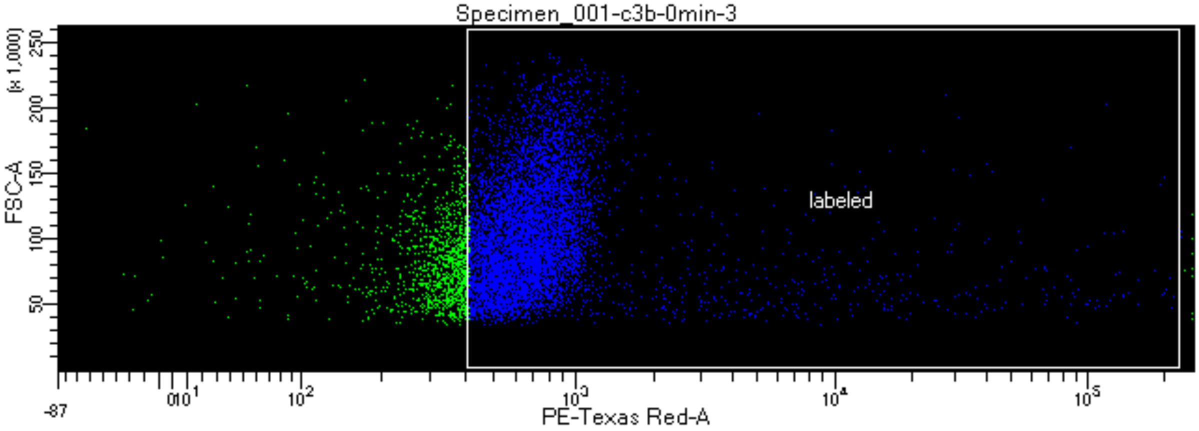

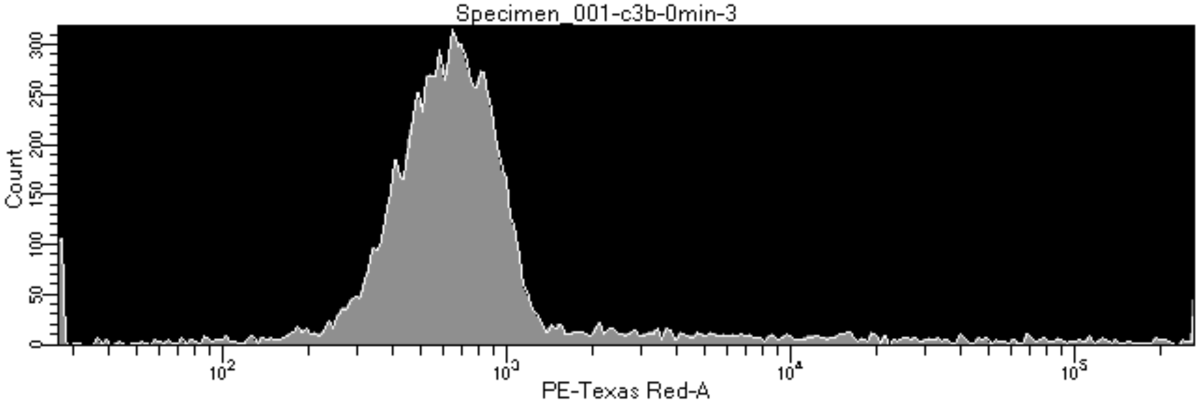

Tube: c3b-0min-3

Population	#Events	%Parent	%Total
All Events	10,000	####	100.0
cells	8,083	80.8	80.8
singlets	7,893	97.6	78.9
label‹	6,663	84.4	66.6

BD FACSDiva 8.0.1

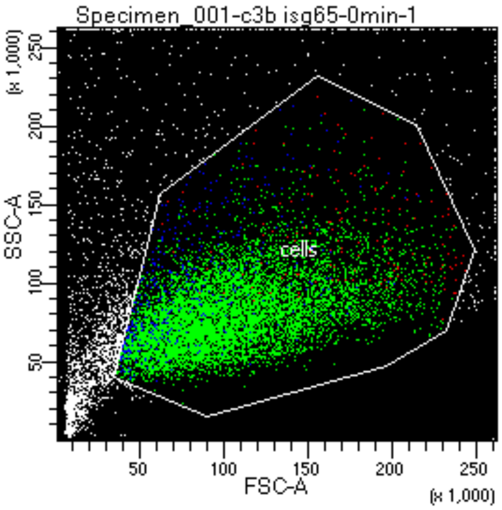

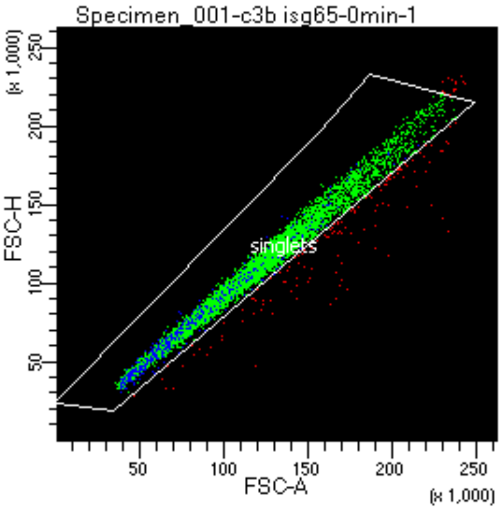

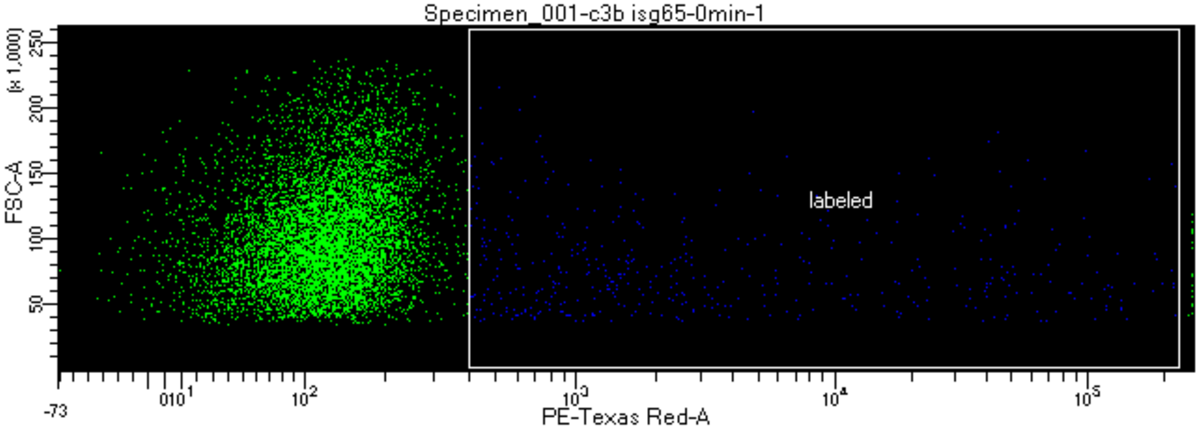

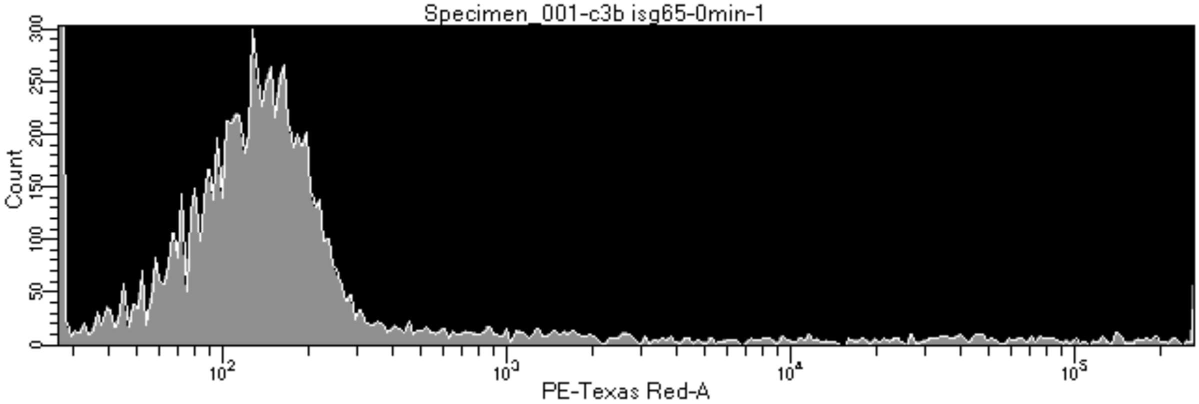

Tube: c3b isg65-0min-1

Population	#Events	%Parent	%Total
All Events	10,000	####	100.0
cells	7,919	79.2	79.2
singlets	7,758	98.0	77.6
label(	429	5.5	4.3

BD FACSDiva 8.0.1

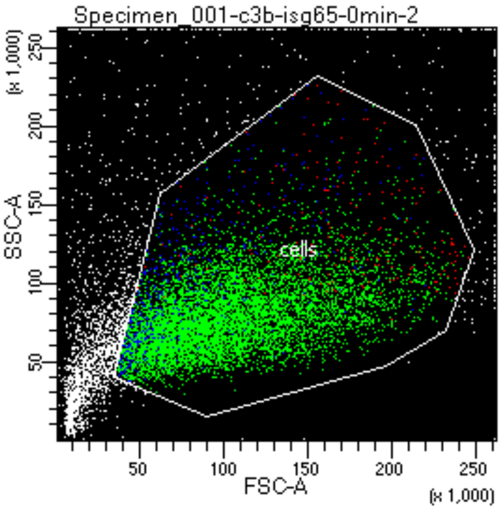

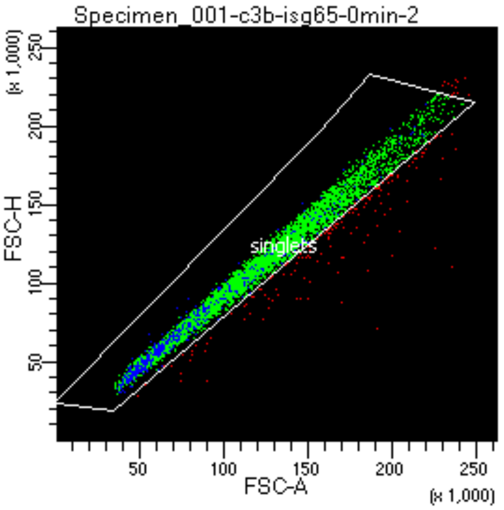

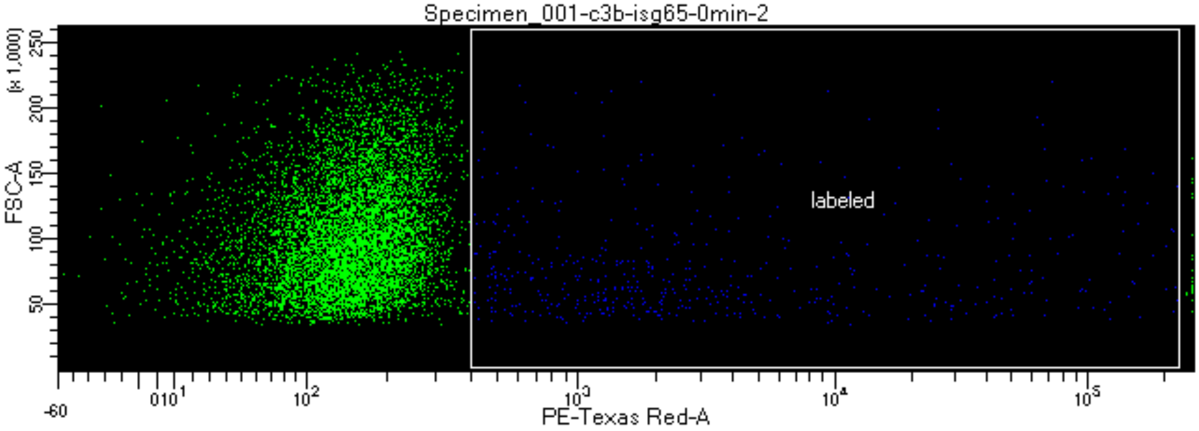

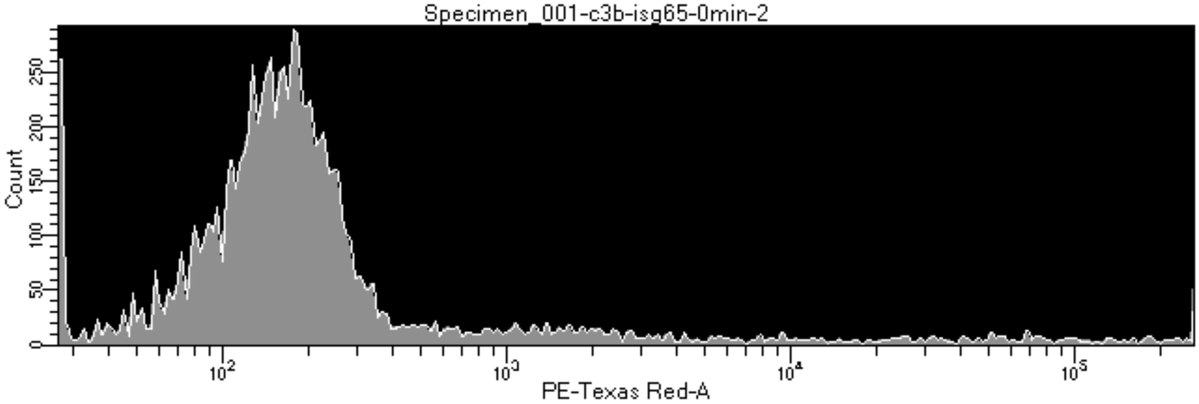

Tube: c3b-isg65-0min-2

Population	#Events	%Parent	%Total
All Events	10,000	####	100.0
cells	7,756	77.6	77.6
singlets	7,560	97.5	75.6
labele	486	6.4	4.9

BD FACSDiva 8.0.1

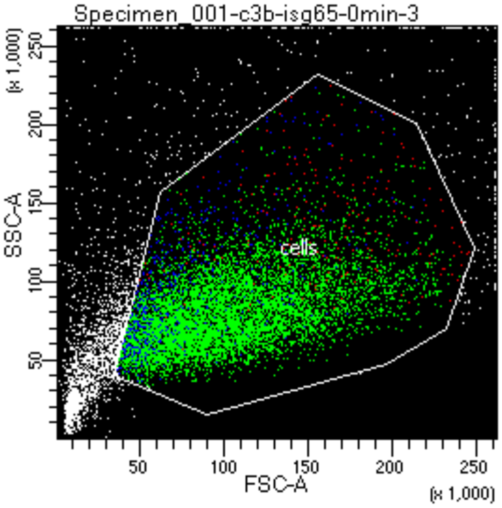

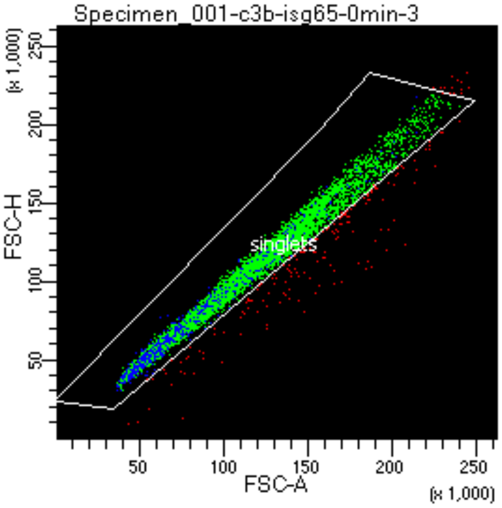

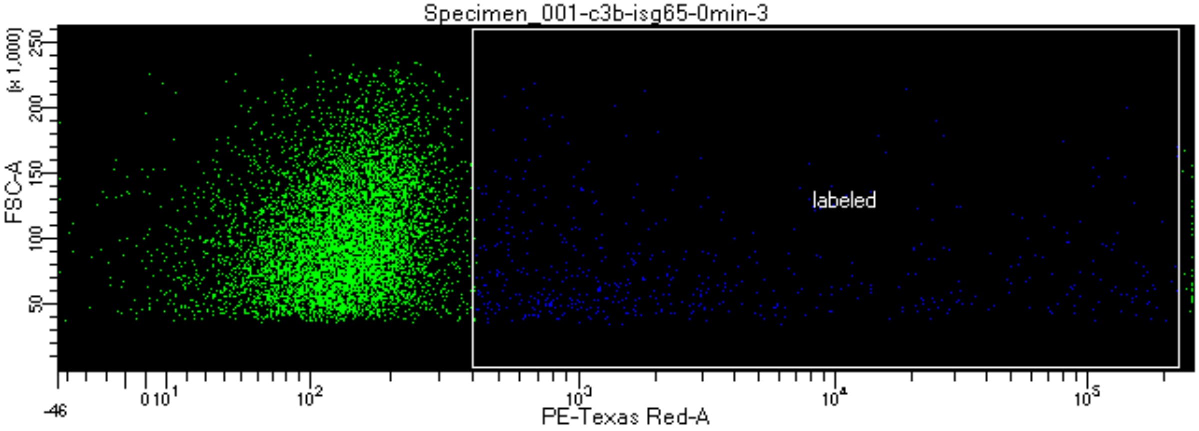

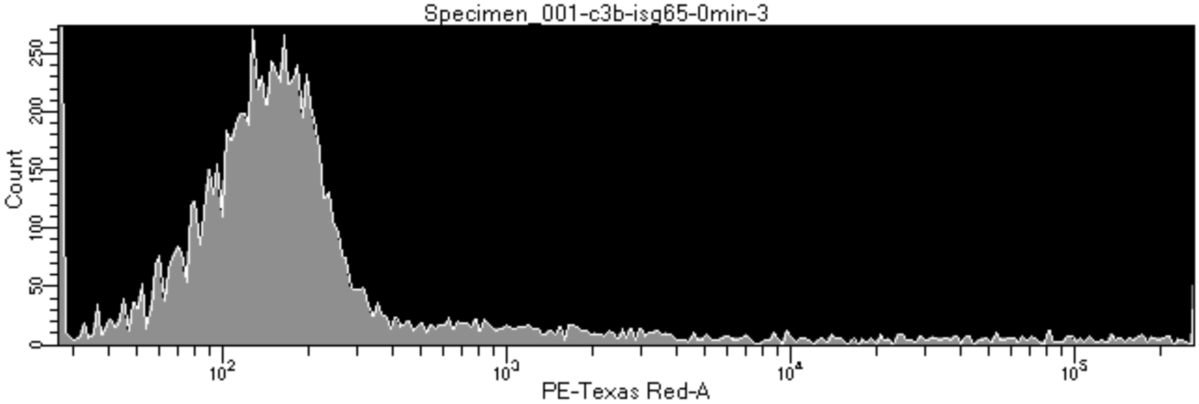

Tube: c3b-isg65-0min-3

Population	#Events	%Parent	%Total
All Events	10,000	####	100.0
cells	7,919	79.2	79.2
singlets	7,711	97.4	77.1
label‹	534	6.9	5.3

BD FACSDiva 8.0.1

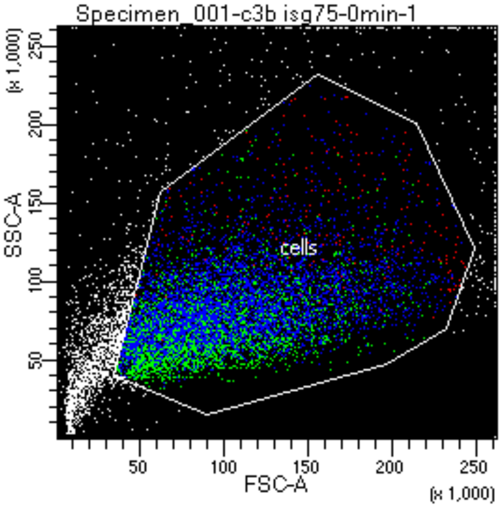

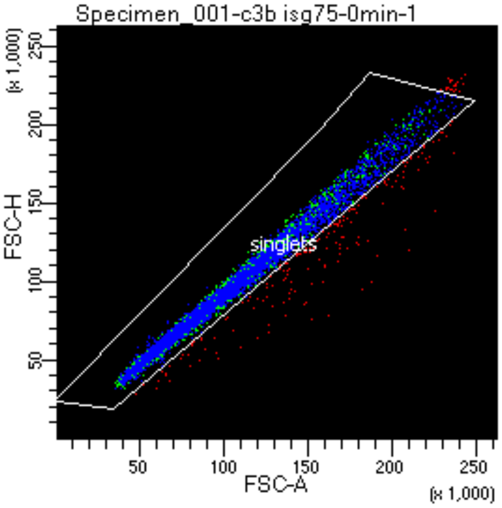

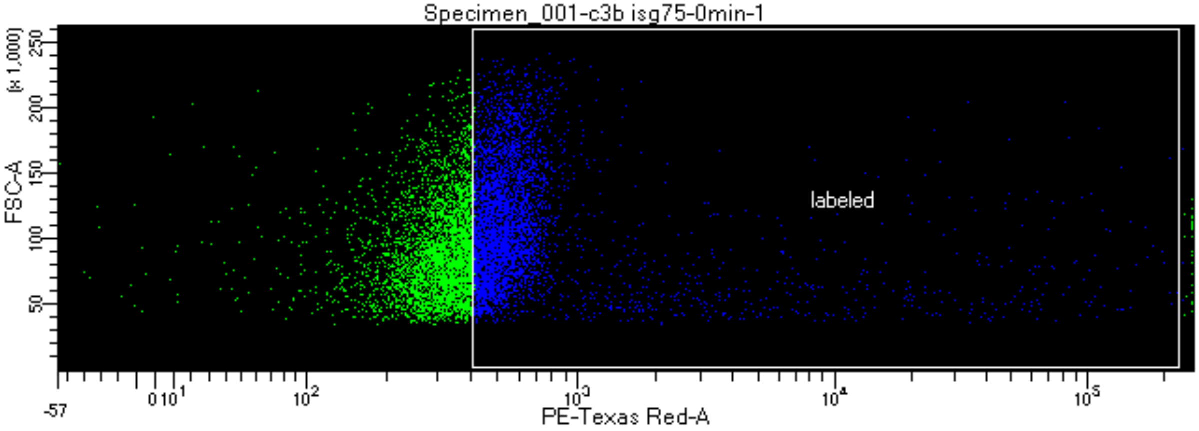

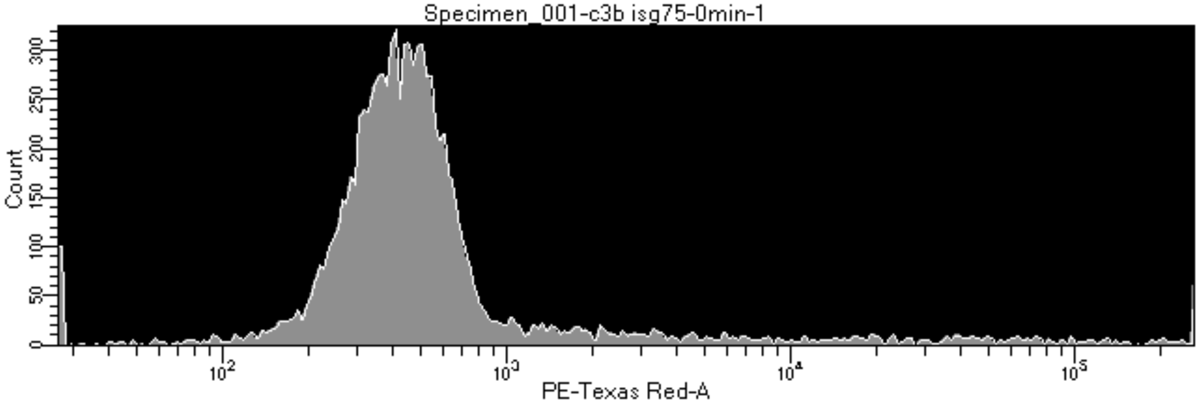

Tube: c3b isg75-0min-1

Population	#Events	%Parent	%Total
All Events	10,000	####	100.0
cells	8,067	80.7	80.7
singlets	7,852	97.3	78.5
label(	4,095	52.2	40.9

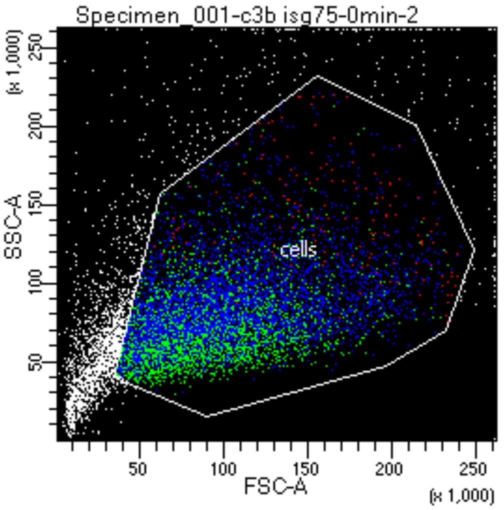

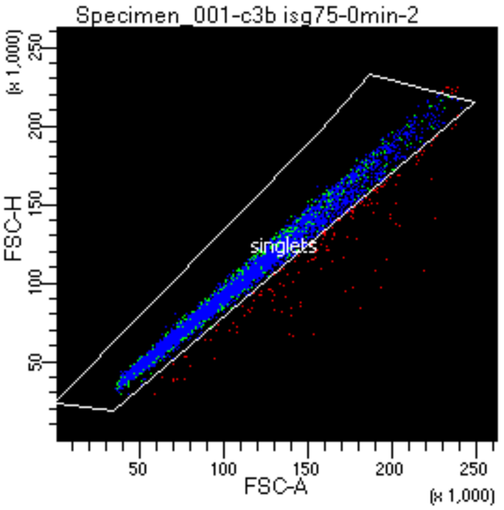

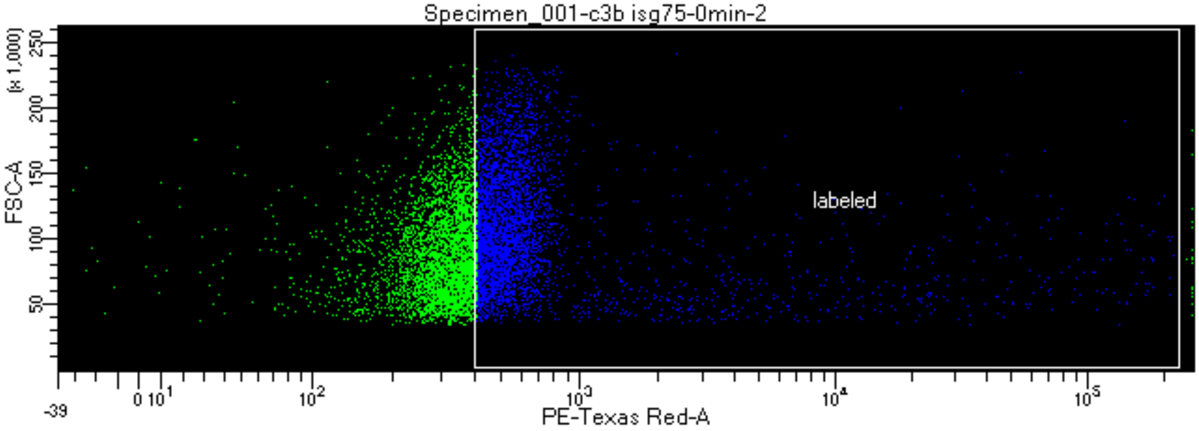

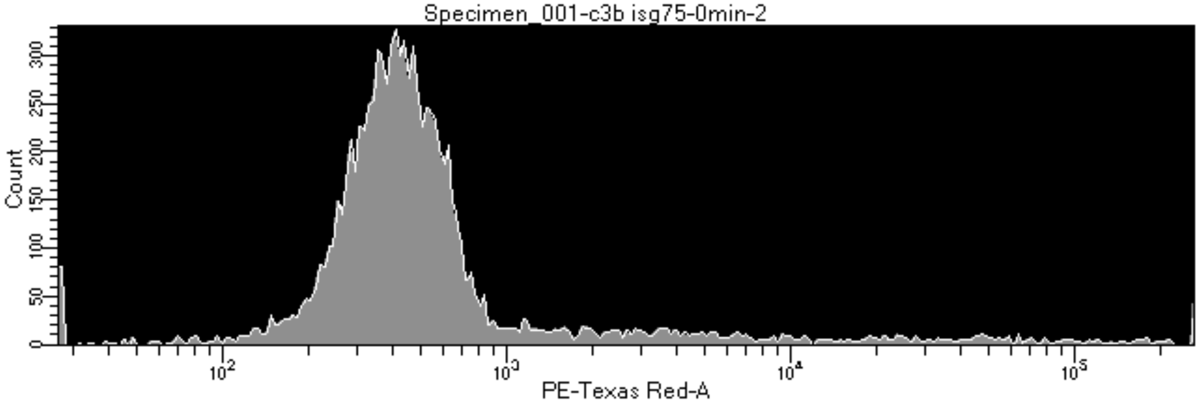

Tube: c3b isg75-0min-2

Population	#Events	%Parent	%Total
All Events	10,000	####	100.0
cells	8,034	80.3	80.3
singlets	7,860	97.8	78.6
label	3,958	50.4	39.6

BD FACSDiva 8.0.1

Specimen_001-c3b-isg75-0 min-3

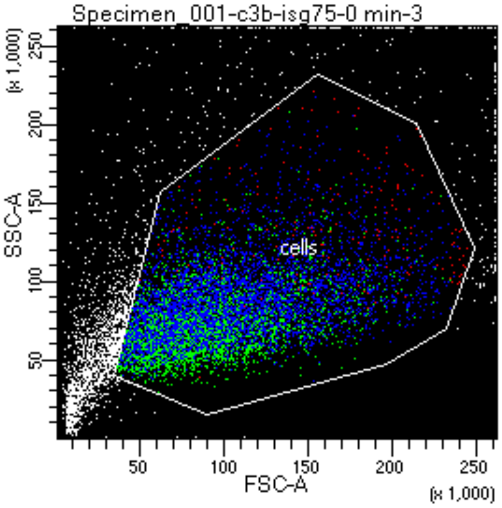

Specimen_001-c3b-isg75-0 min-3

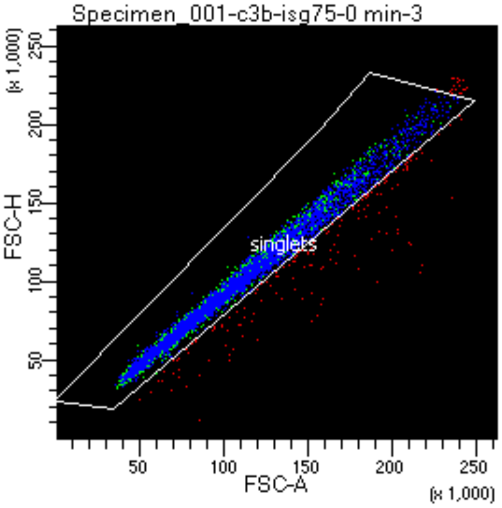

Specimen_001-c3b-isg75-0 min-3

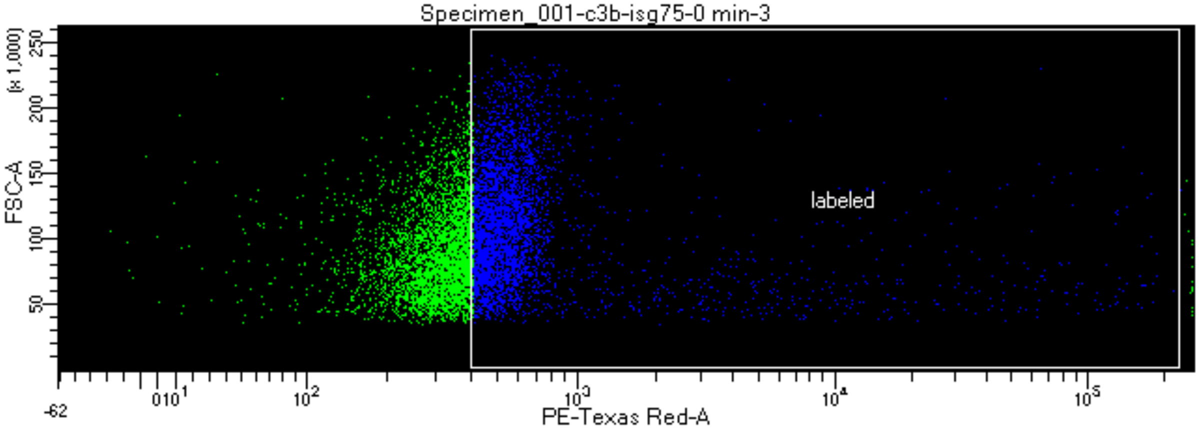

Specimen_001-c3b-isg75-0 min-3

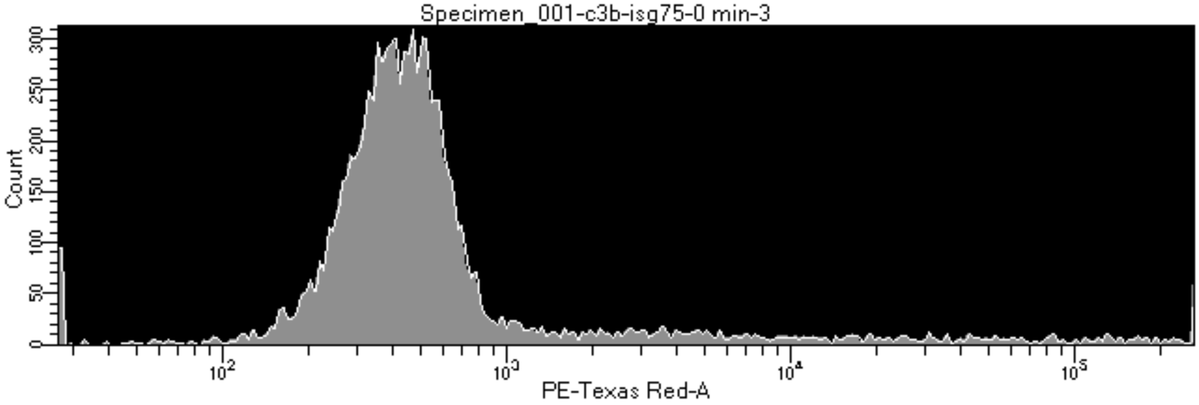

Tube: c3b-isg75-0 min-3

Population	#Events	%Parent	%Total
All Events	10,000	####	100.0
cells	8,017	80.2	80.2
singlets	7,812	97.4	78.1
label(	4,002	51.2	40.0

BD FACSDiva 8.0.1

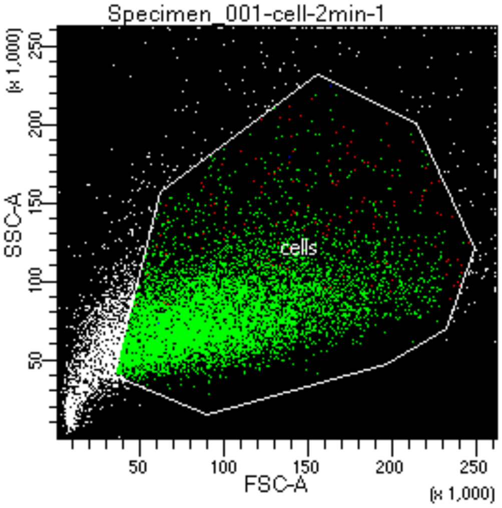

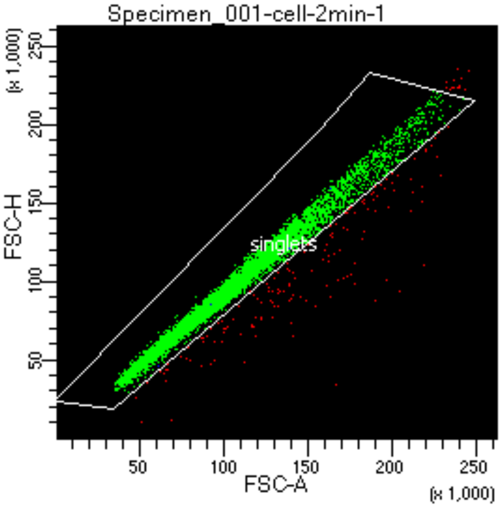

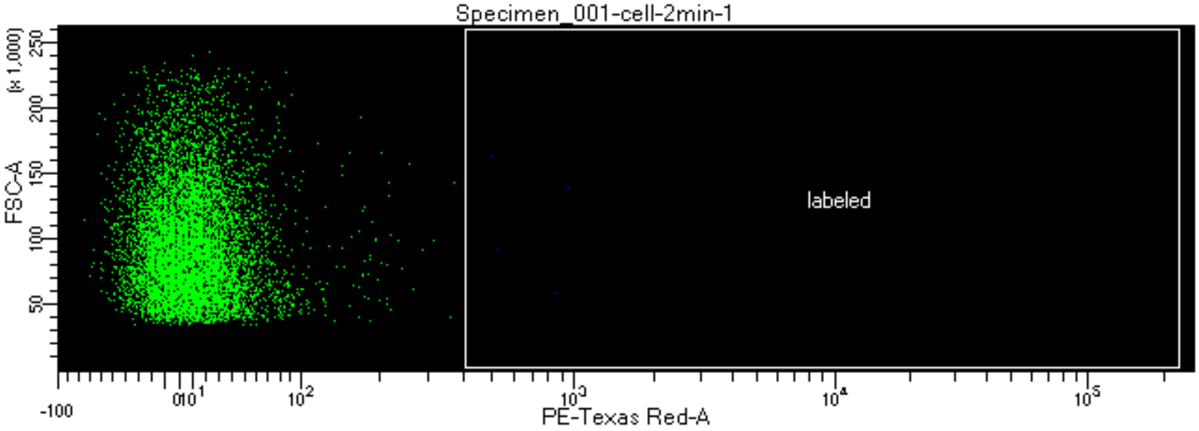

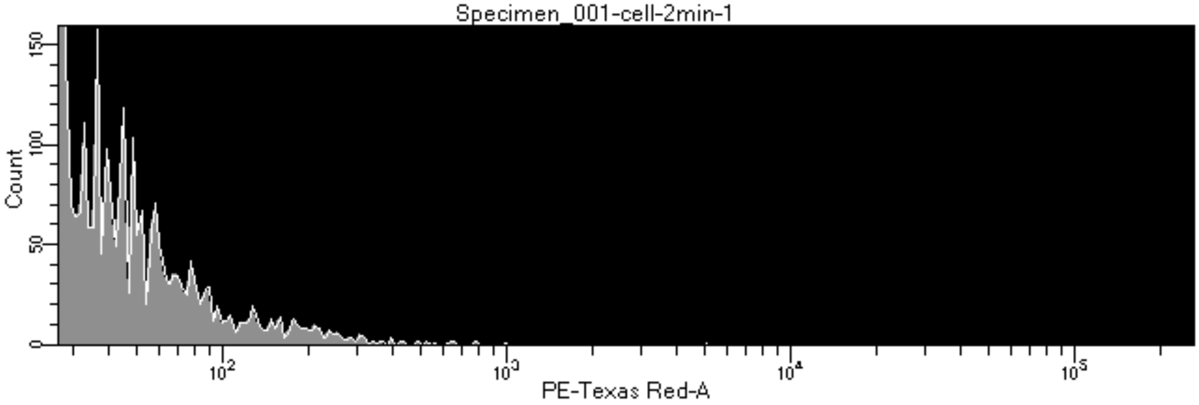

Tube: cell-2min-1

Population	#Events	%Parent	%Total
All Events	10,000	####	100.0
cells	7,767	77.7	77.7
singlets	7,597	97.8	76.0
labele	4	0.1	0.0

 Printed on: Mon Aug 22, 2022 11:50:24 CEST

BD FACSDiva 8.0.1

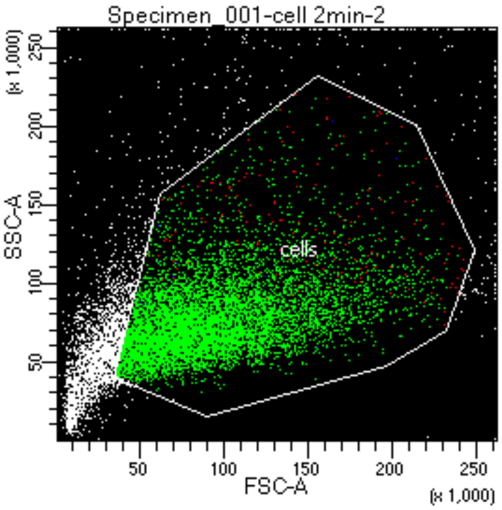

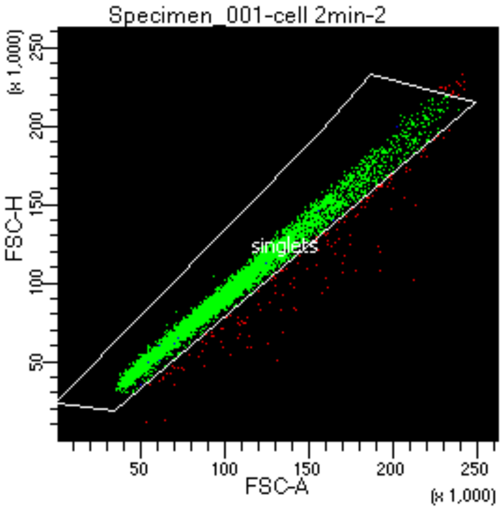

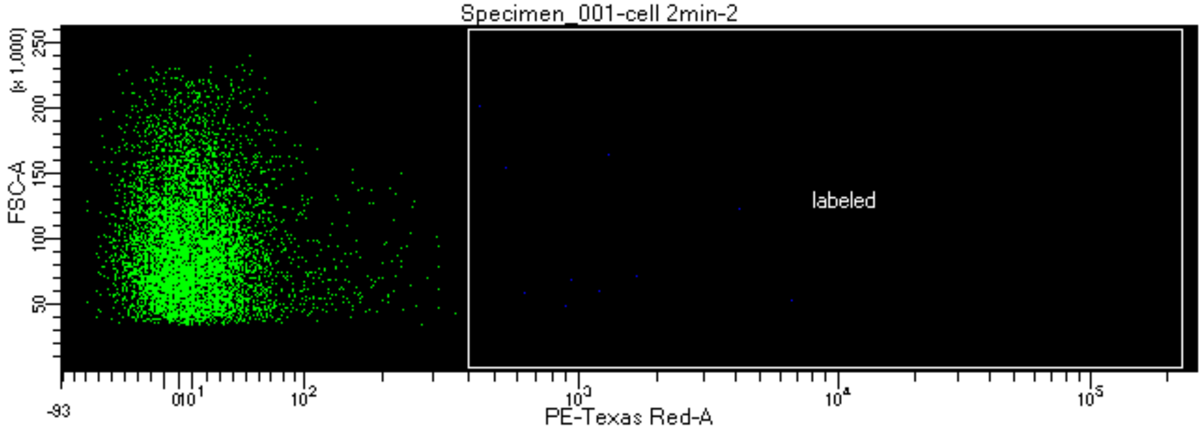

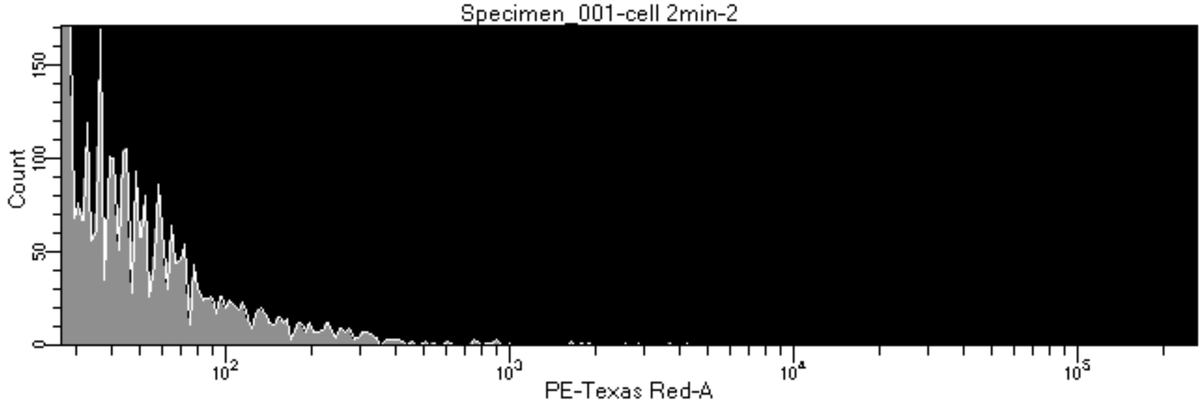

Tube: cell 2min-2

Population	#Events	%Parent	%Total
All Events	10,000	####	100.0
cells	7,286	72.9	72.9
singlets	7,107	97.5	71.1
labele	10	0.1	0.1

BD FACSDiva 8.0.1

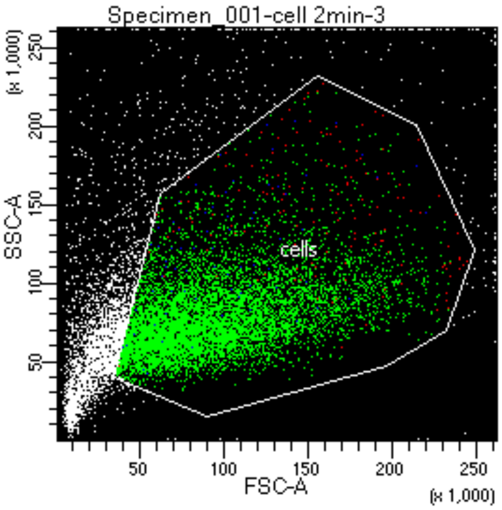

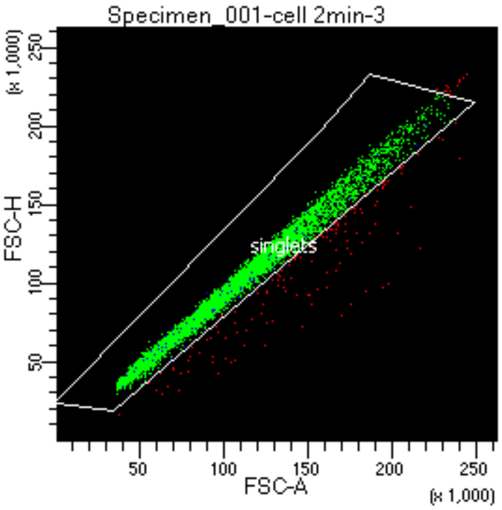

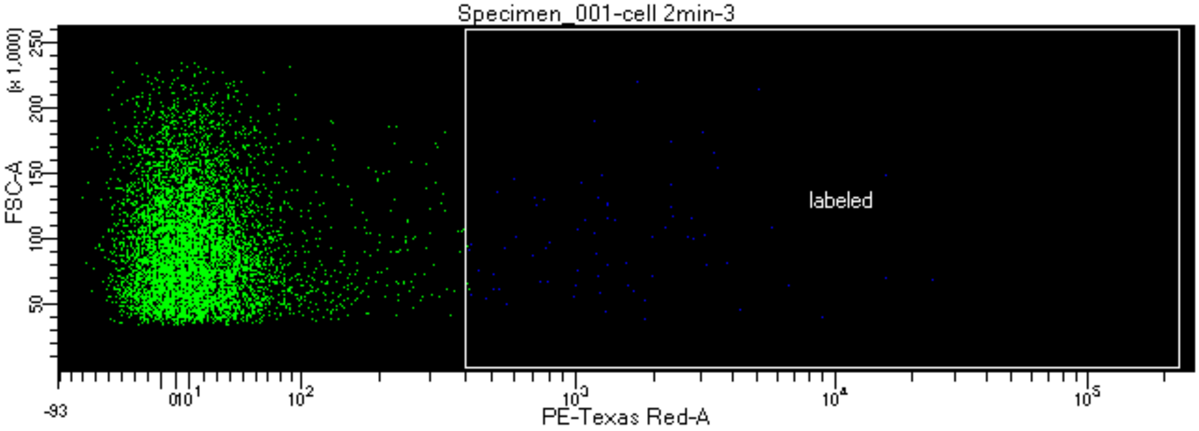

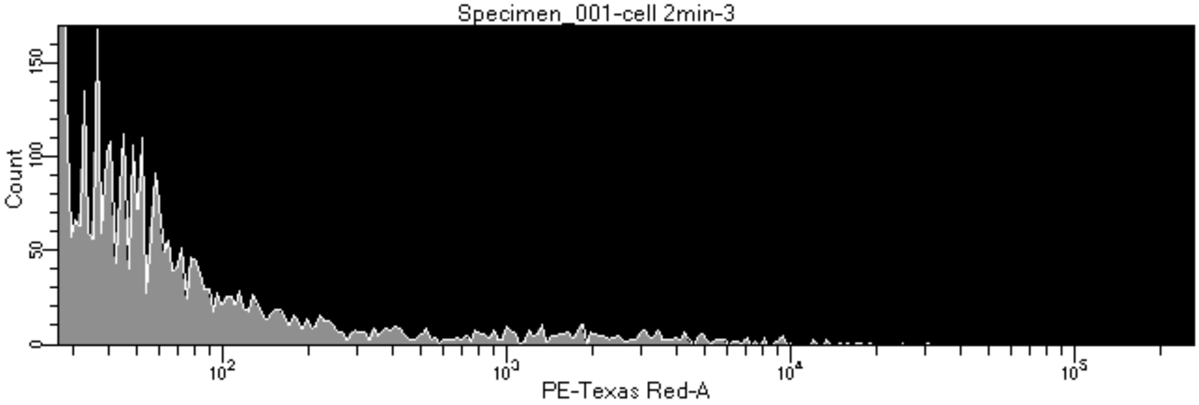

Tube: cell 2min-3

Population	#Events	%Parent	%Total
All Events	10,000	####	100.0
cells	6,942	69.4	69.4
singlets	6,784	97.7	67.8
label(	70	1.0	0.7

BD FACSDiva 8.0.1

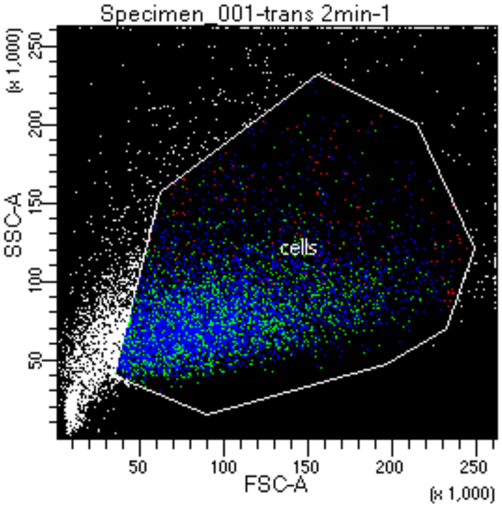

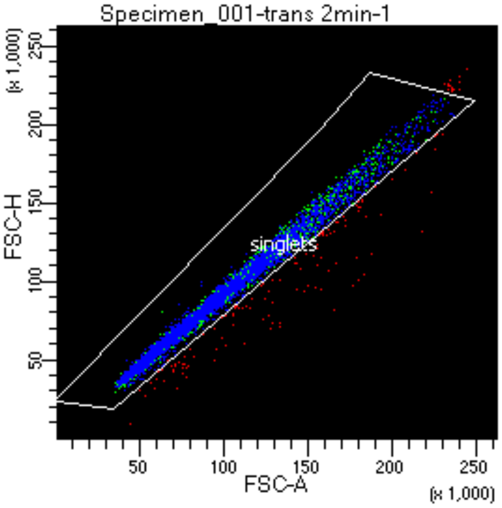

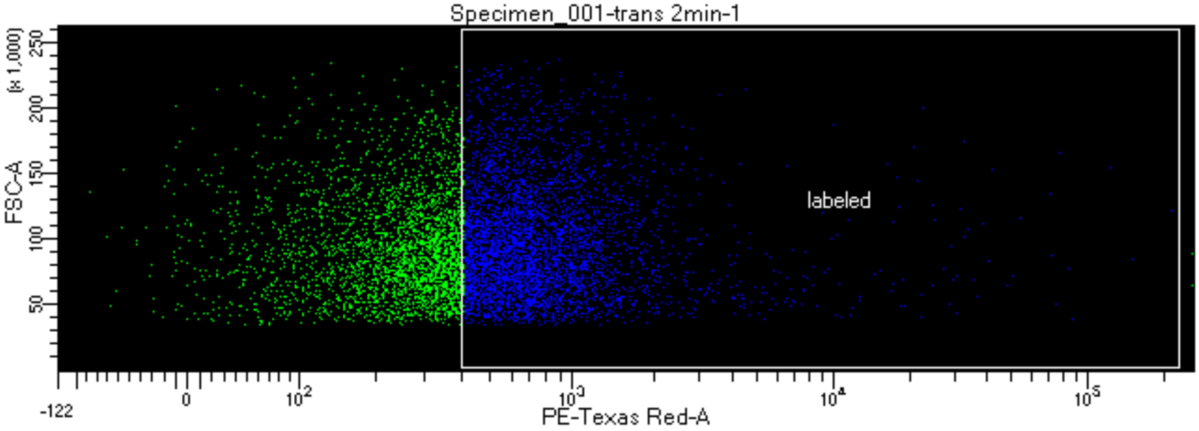

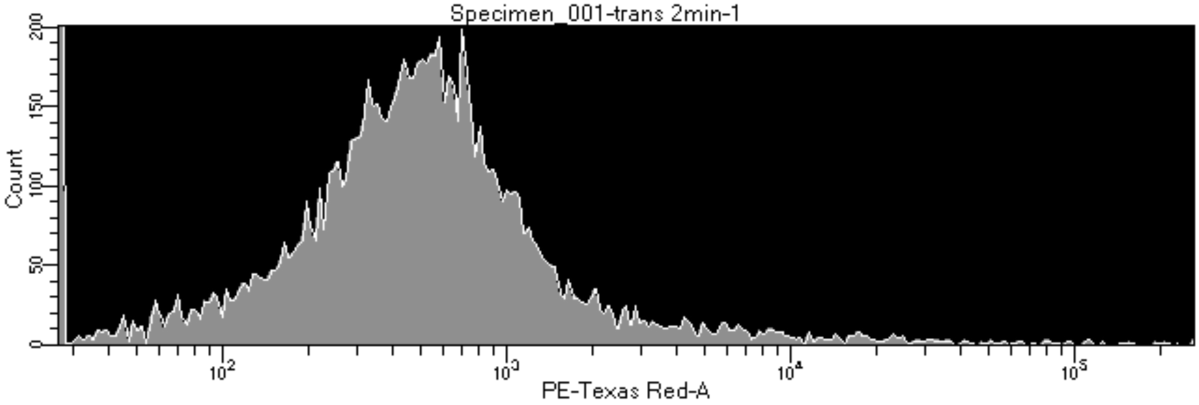

Tube: trans 2min-1

Population	#Events	%Parent	%Total
All Events	10,000	####	100.0
cells	7,213	72.1	72.1
singlets	7,066	98.0	70.7
label‹	4,139	58.6	41.4

BD FACSDiva 8.0.1

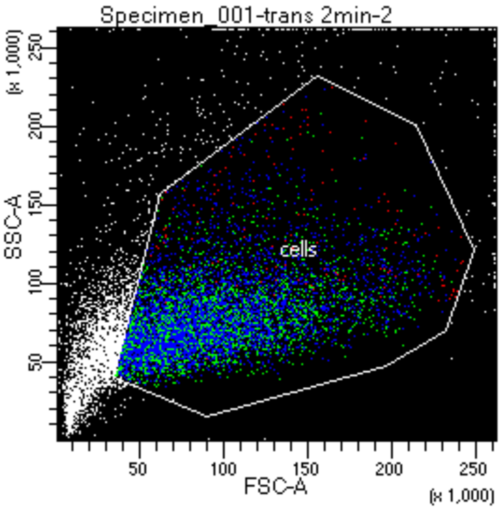

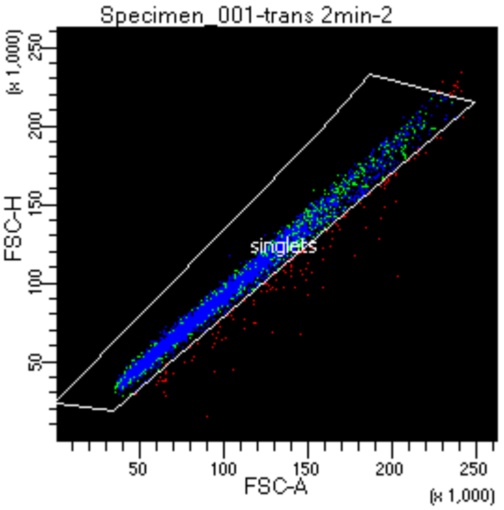

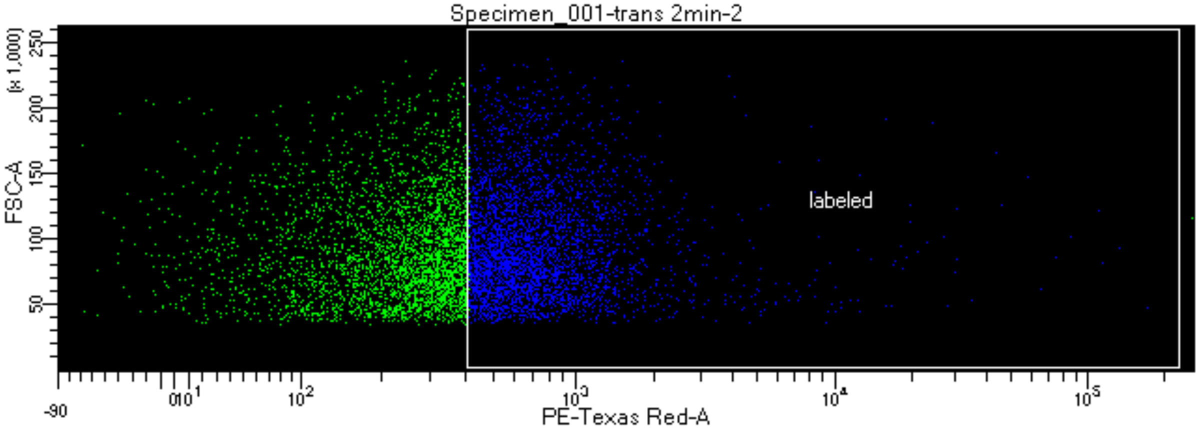

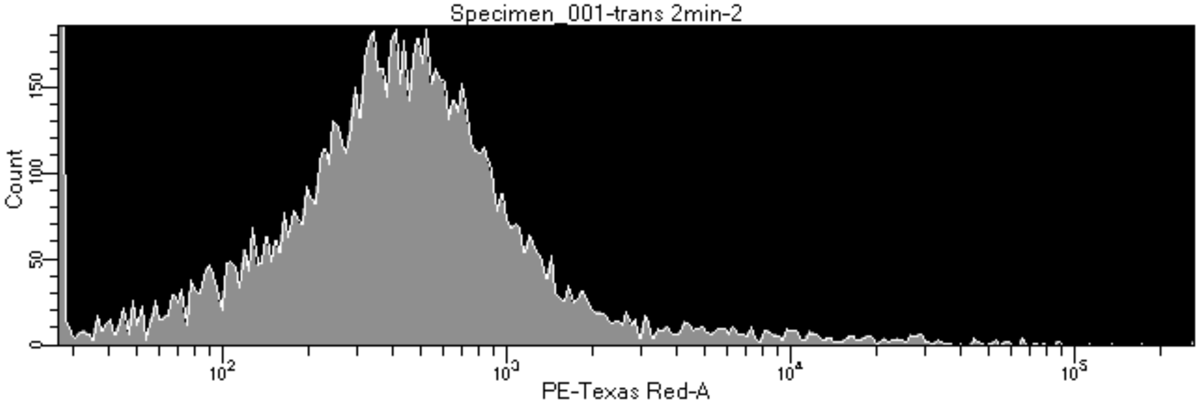

Tube: trans 2min-2

Population	#Events	%Parent	%Total
All Events	10,000	####	100.0
cells	6,948	69.5	69.5
singlets	6,803	97.9	68.0
label‹	3,502	51.5	35.0

BD FACSDiva 8.0.1

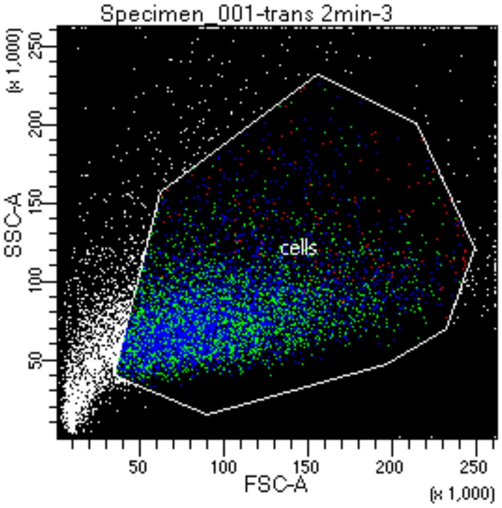

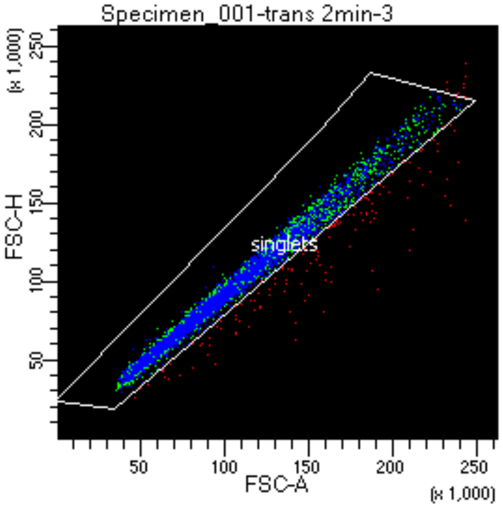

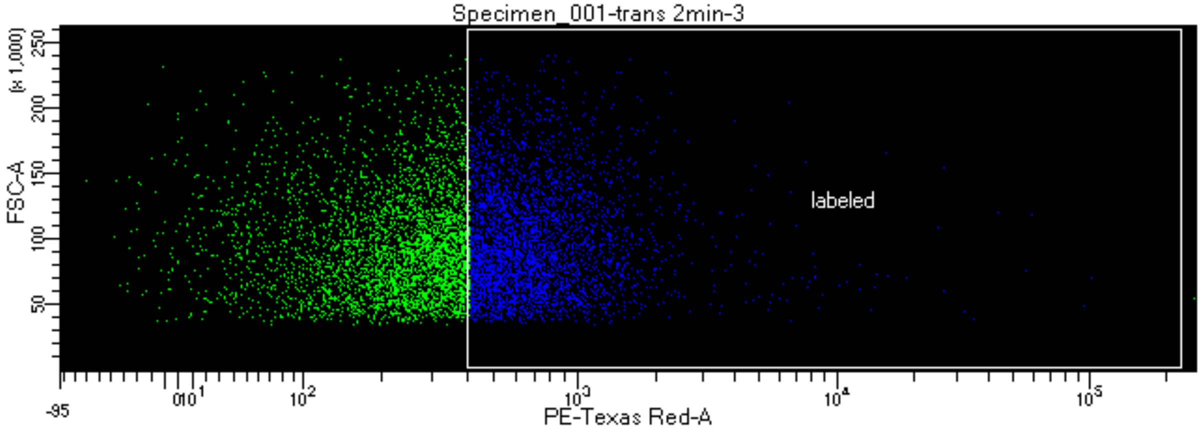

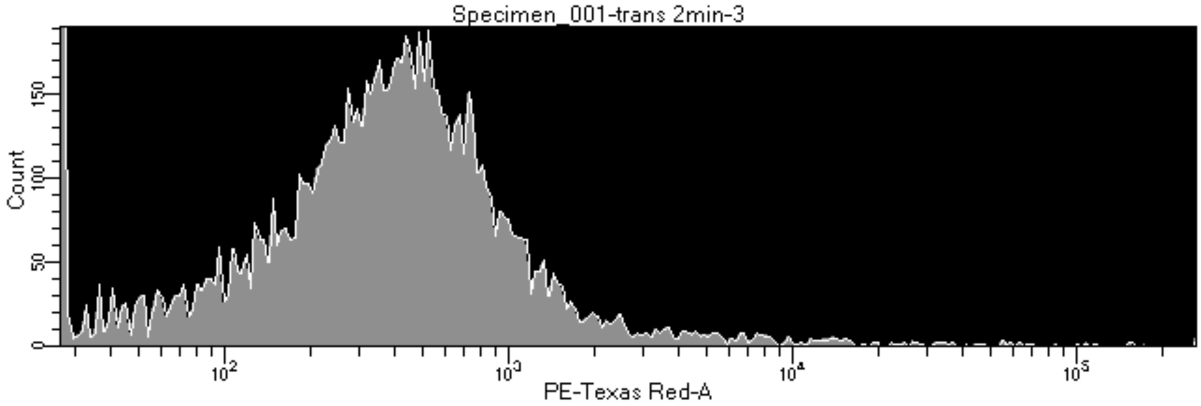

Tube: trans 2min-3

Population	#Events	%Parent	%Total
All Events	10,000	####	100.0
cells	7,259	72.6	72.6
singlets	7,097	97.8	71.0
label‹	3,450	48.6	34.5

BD FACSDiva 8.0.1

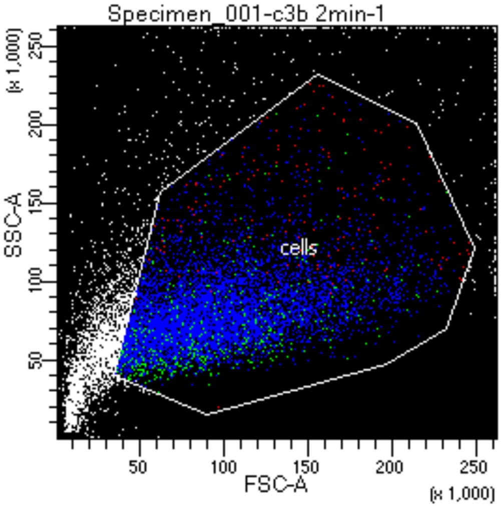

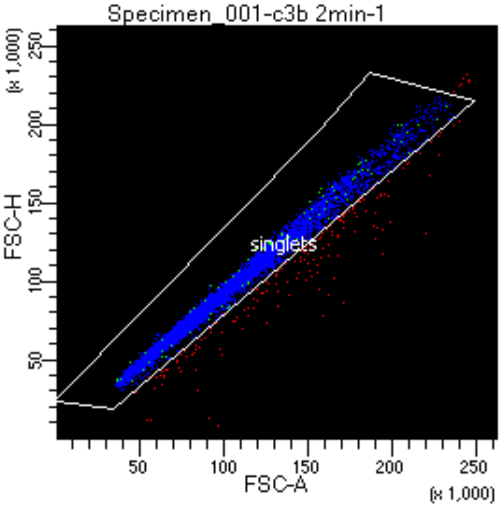

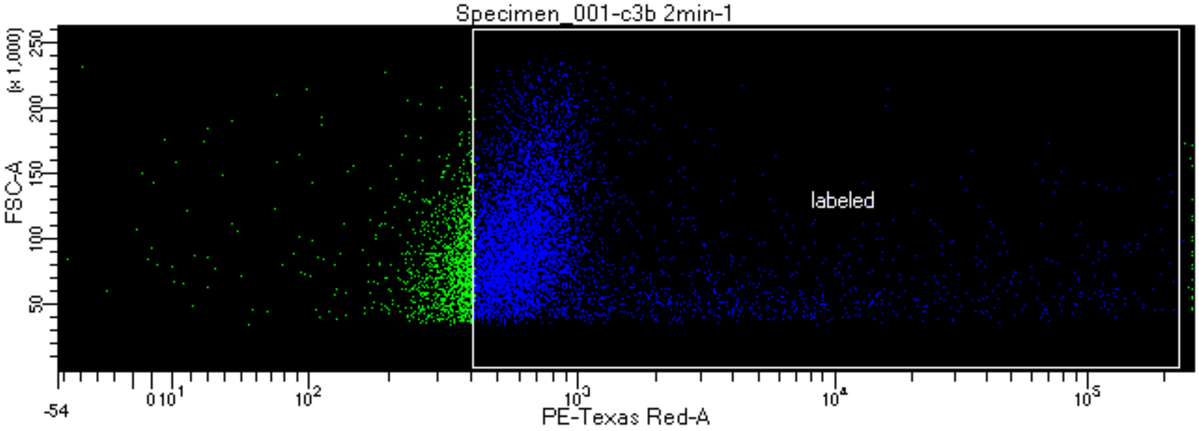

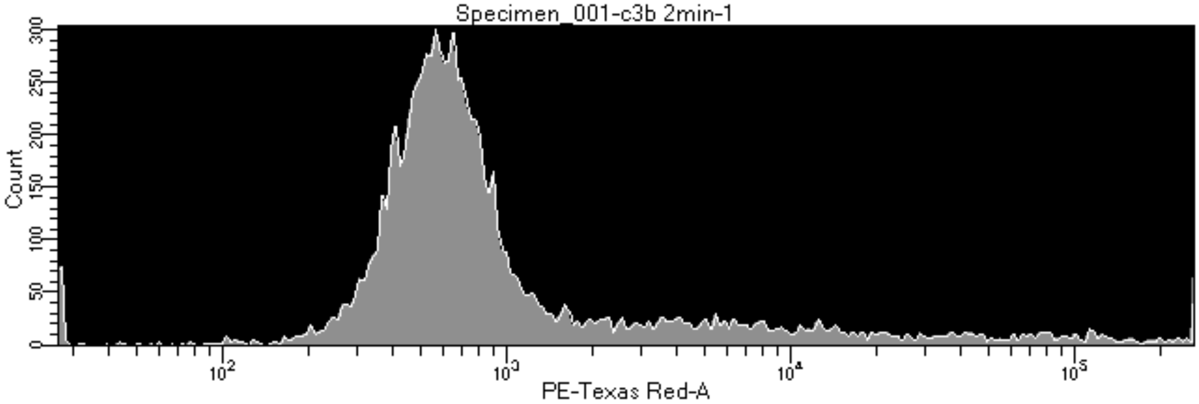

Tube: c3b 2min-1

Population	#Events	%Parent	%Total
All Events	10,000	####	100.0
cells	7,298	73.0	73.0
singlets	7,123	97.6	71.2
labele	5,895	82.8	59.0

BD FACSDiva 8.0.1

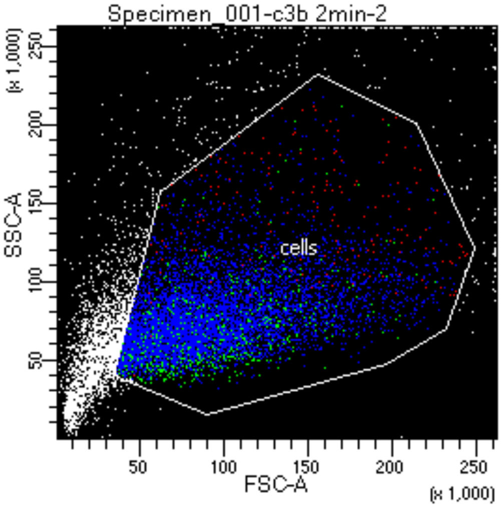

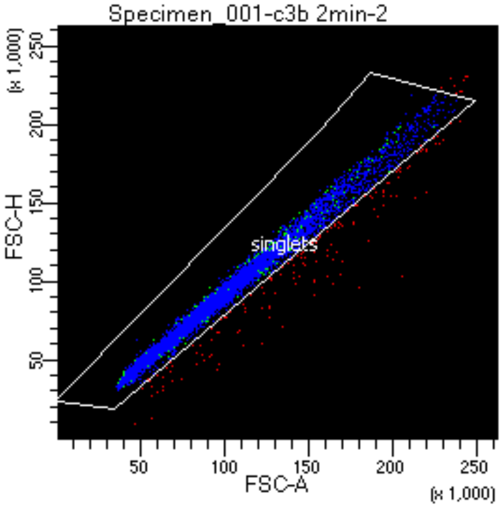

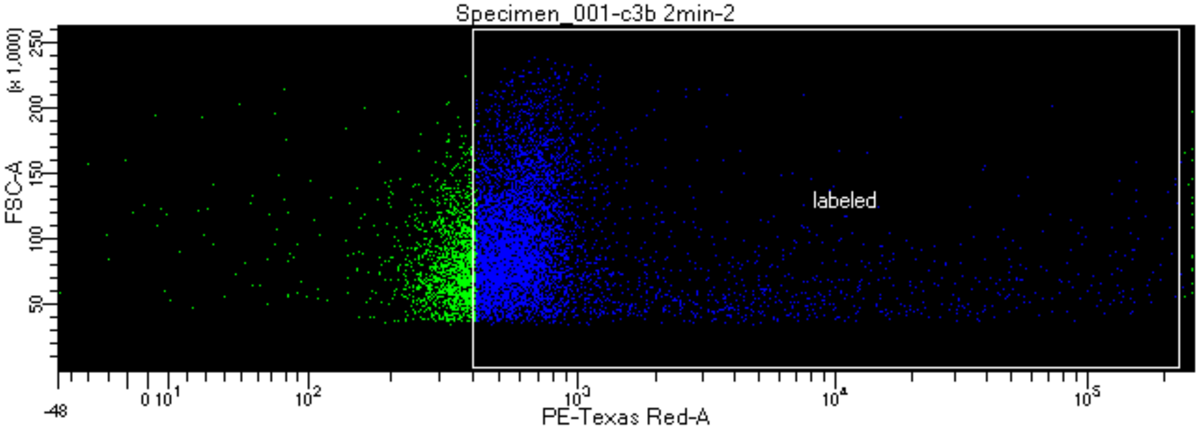

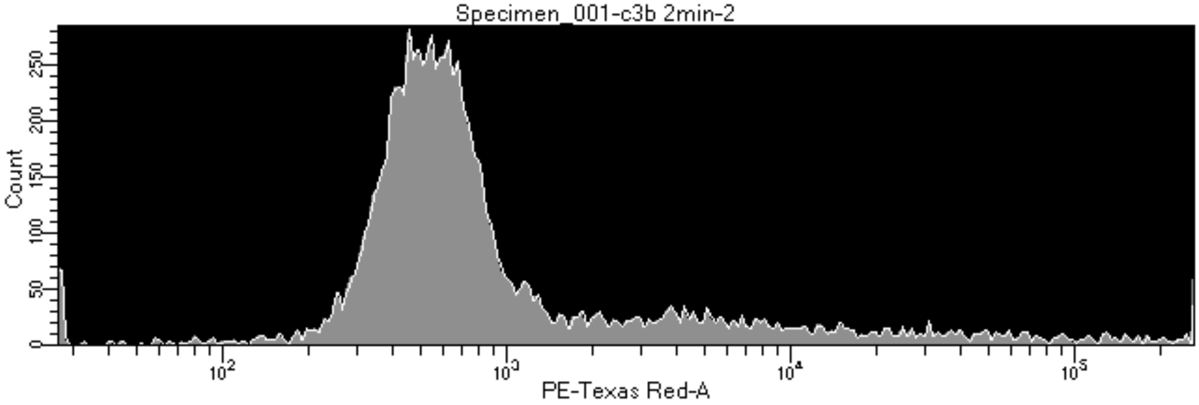

Tube: c3b 2min-2

Population	#Events	%Parent	%Total
All Events	10,000	####	100.0
cells	7,138	71.4	71.4
singlets	6,958	97.5	69.6
label(	5,364	77.1	53.6

BD FACSDiva 8.0.1

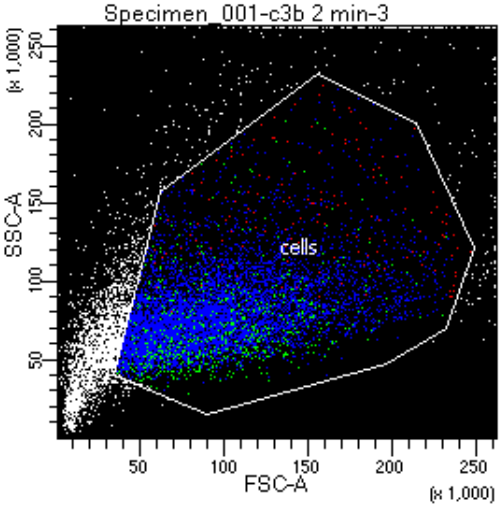

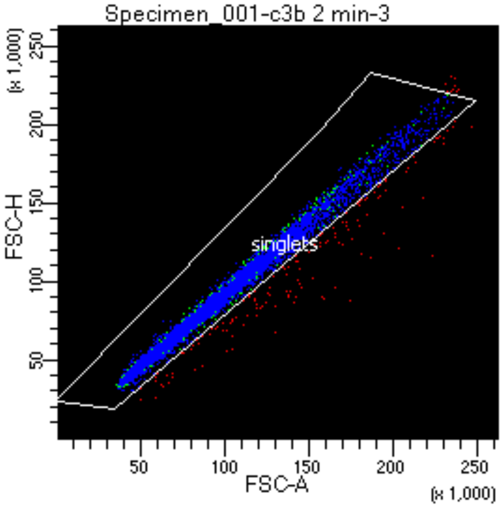

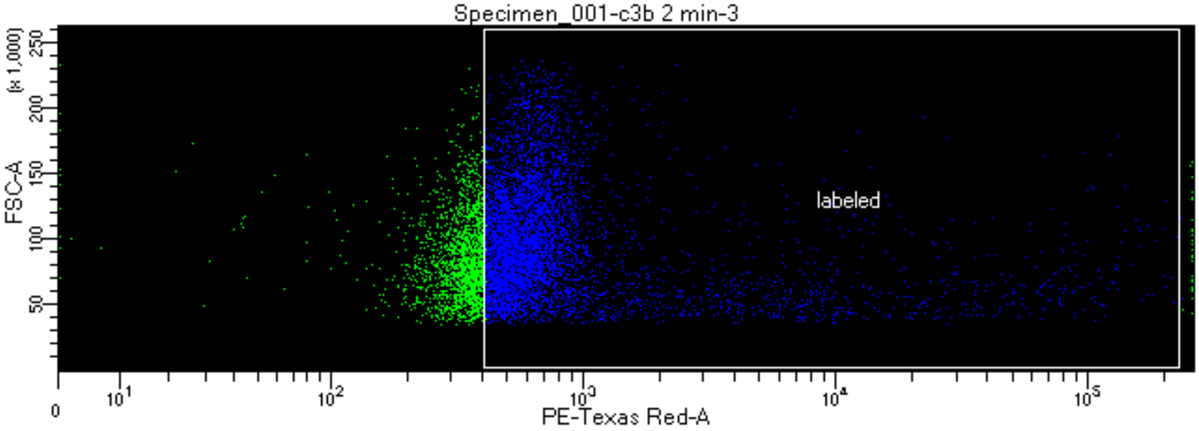

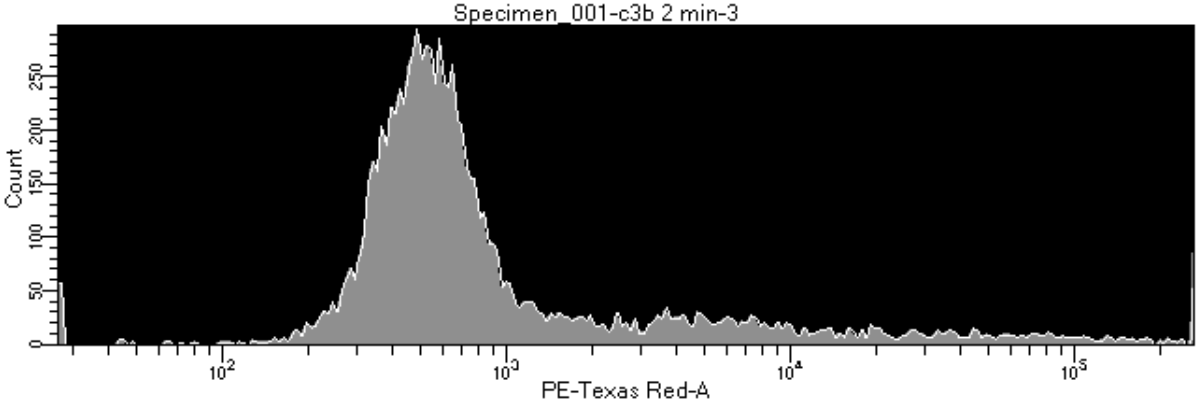

Tube: c3b 2 min-3

Population	#Events	%Parent	%Total
All Events	10,000	####	100.0
cells	7,283	72.8	72.8
singlets	7,120	97.8	71.2
labele	5,301	74.5	53.0

BD FACSDiva 8.0.1

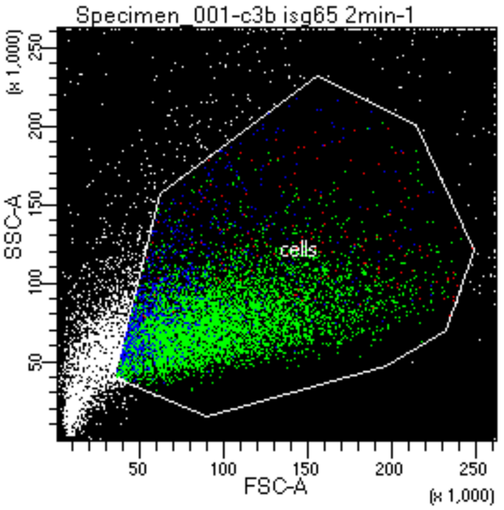

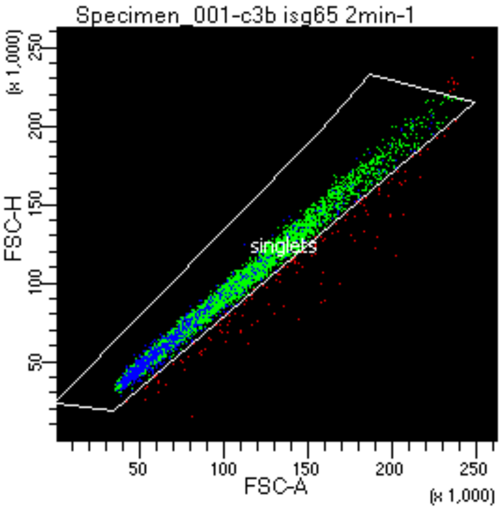

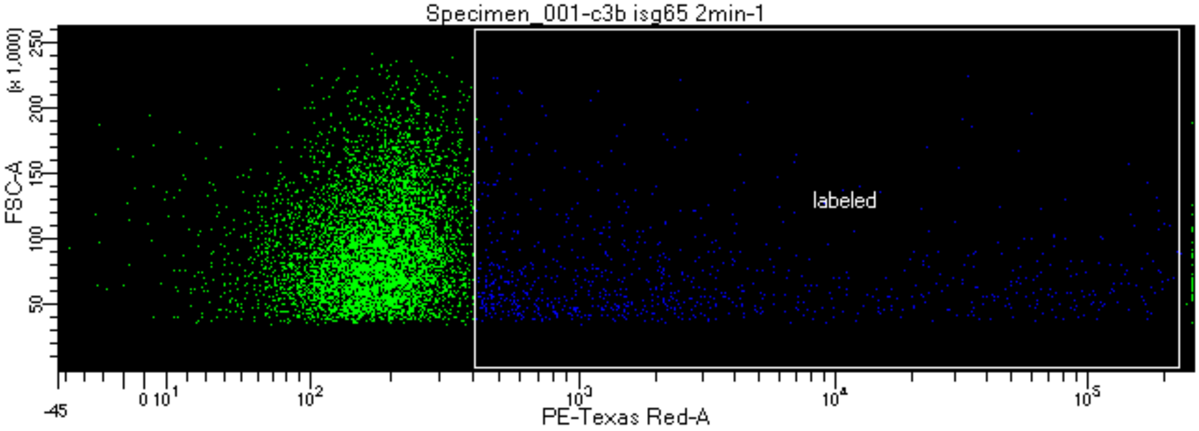

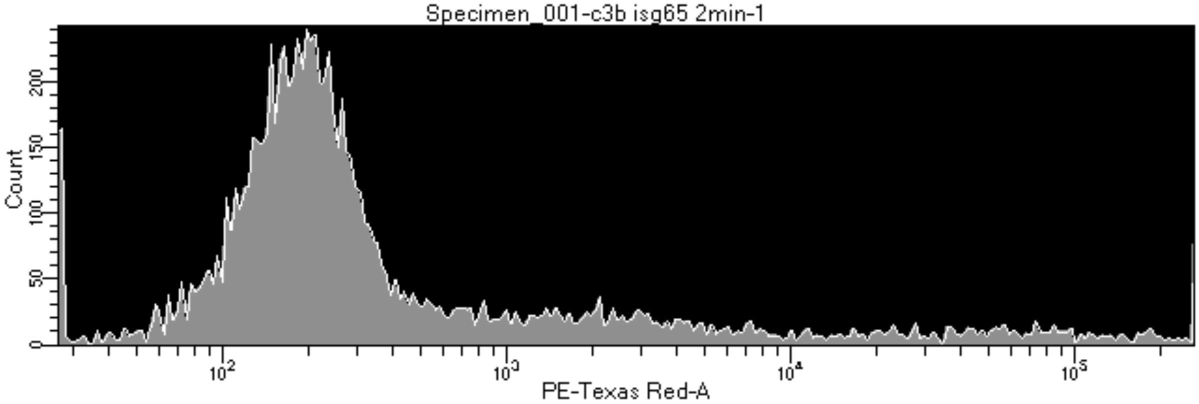

Tube: c3b isg65 2min-1

Population	#Events	%Parent	%Total
All Events	10,000	####	100.0
cells	6,734	67.3	67.3
singlets	6,578	97.7	65.8
labele	848	12.9	8.5

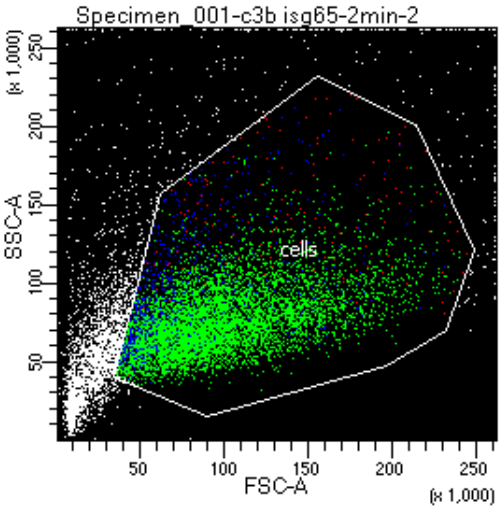

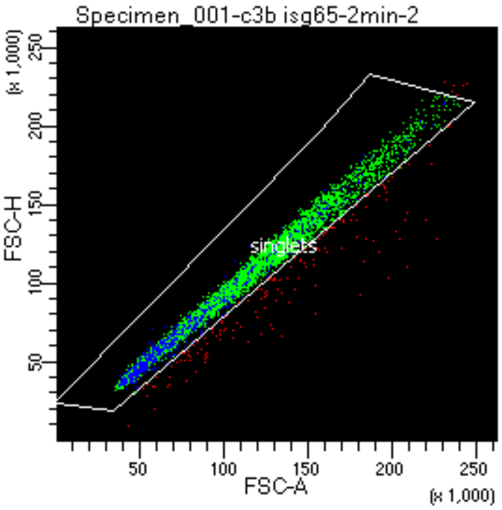

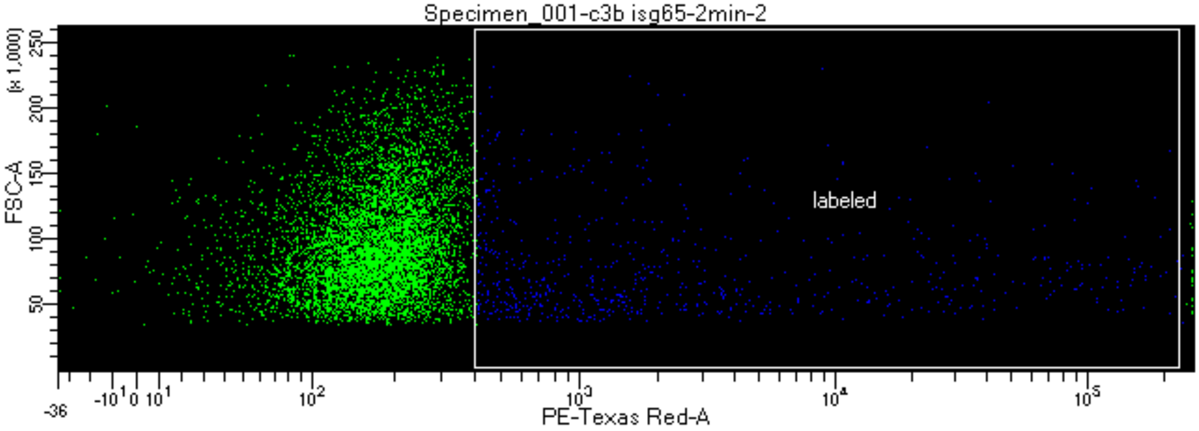

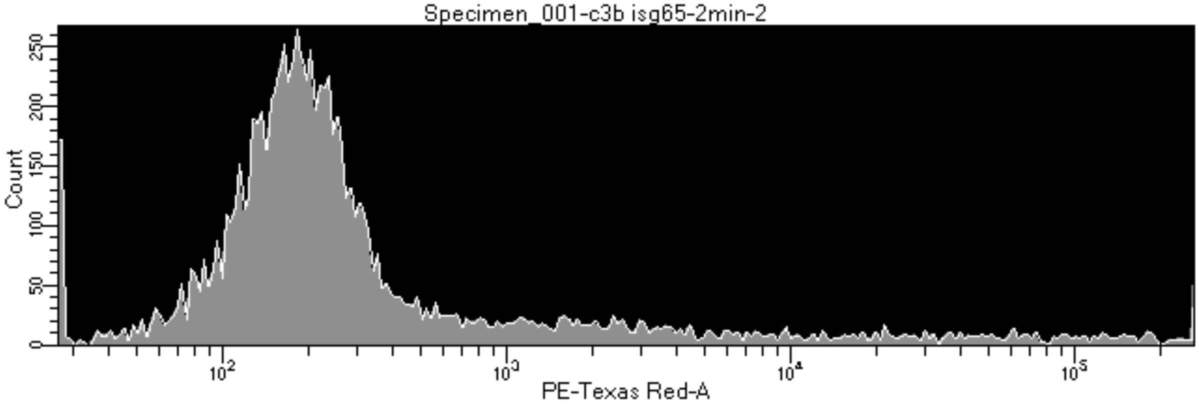

Tube: c3b isg65-2min-2

Population	#Events	%Parent	%Total
All Events	10,000	####	100.0
cells	7,072	70.7	70.7
singlets	6,911	97.7	69.1
label‹	661	9.6	6.6

BD FACSDiva 8.0.1

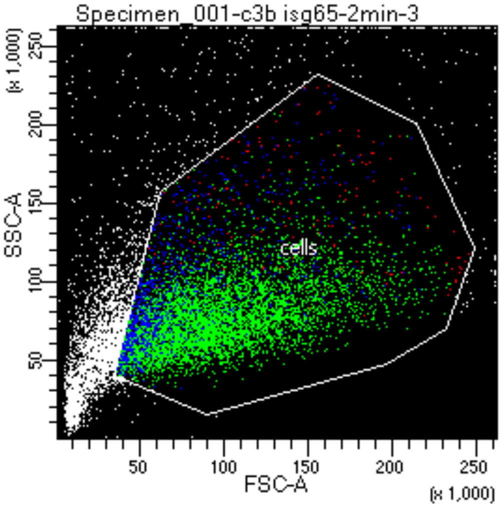

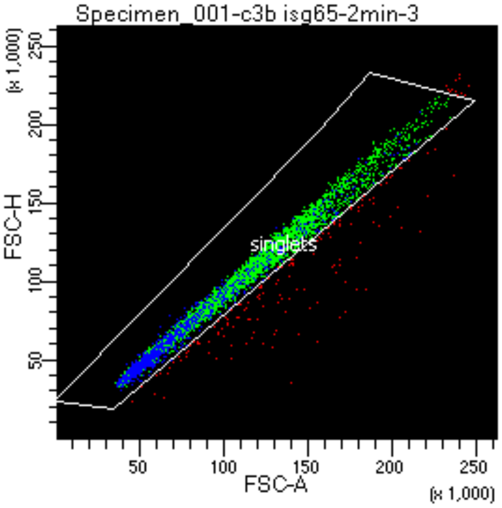

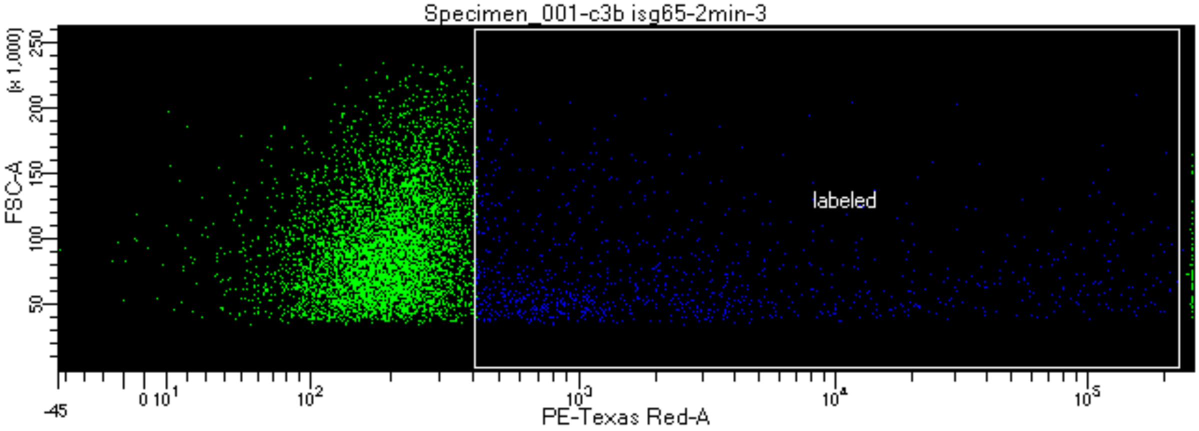

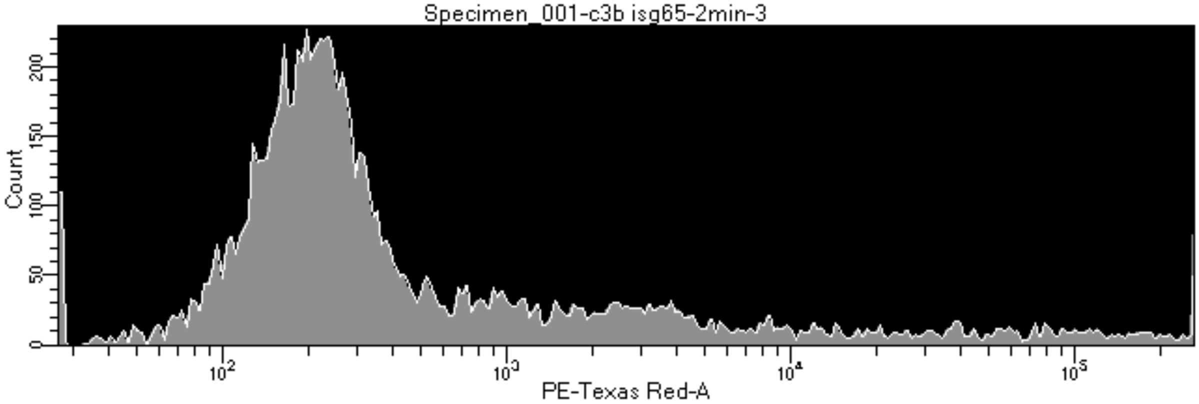

Tube: c3b isg65-2min-3

Population	#Events	%Parent	%Total
All Events	10,000	####	100.0
cells	6,685	66.8	66.8
singlets	6,517	97.5	65.2
labele	1,006	15.4	10.1

BD FACSDiva 8.0.1

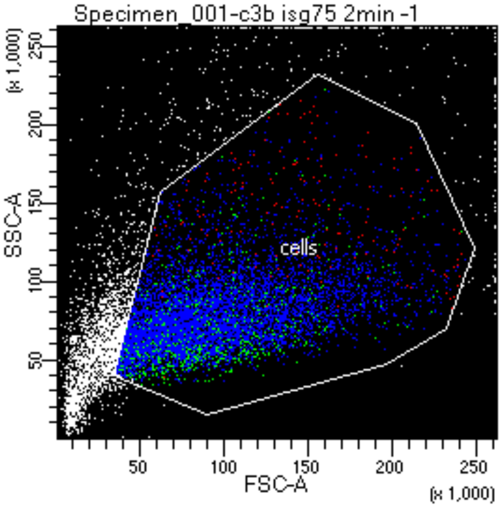

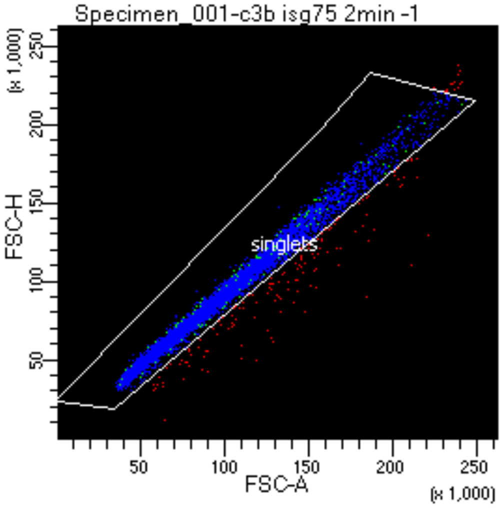

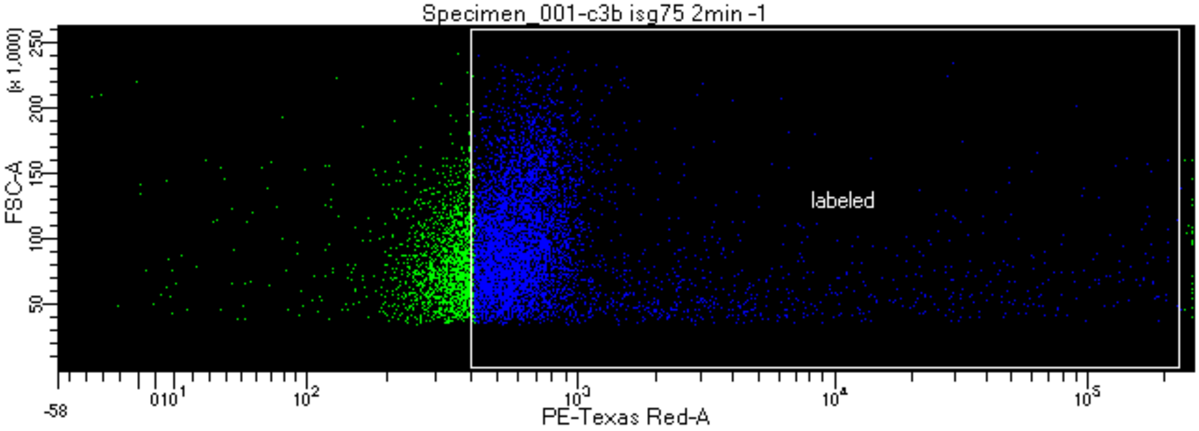

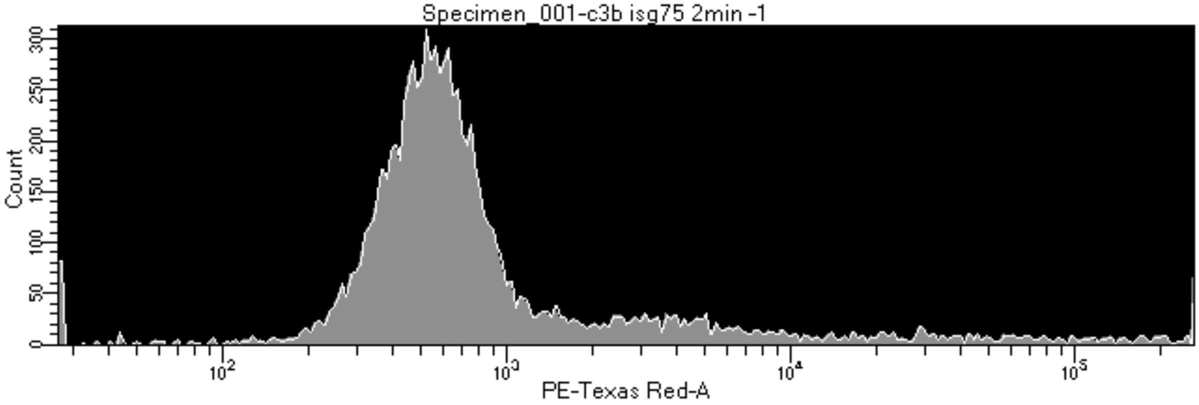

Tube: c3b isg75 2min -1

Population	#Events	%Parent	%Total
All Events	10,000	####	100.0
cells	7,263	72.6	72.6
singlets	7,093	97.7	70.9
label‹	5,436	76.6	54.4

BD FACSDiva 8.0.1

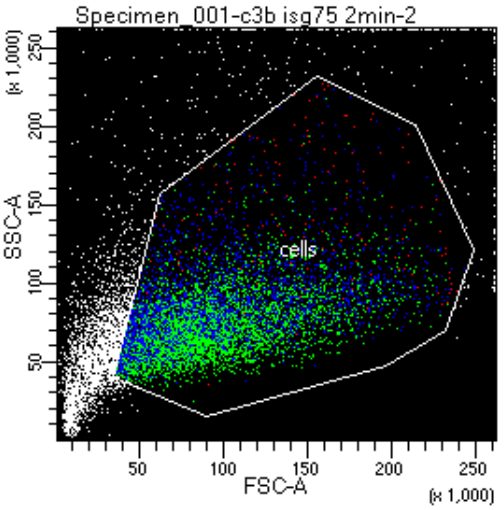

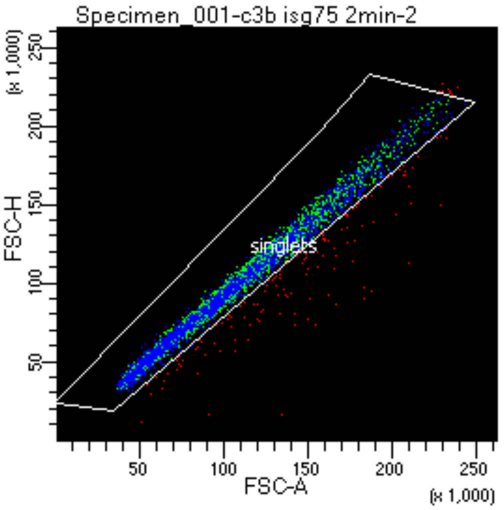

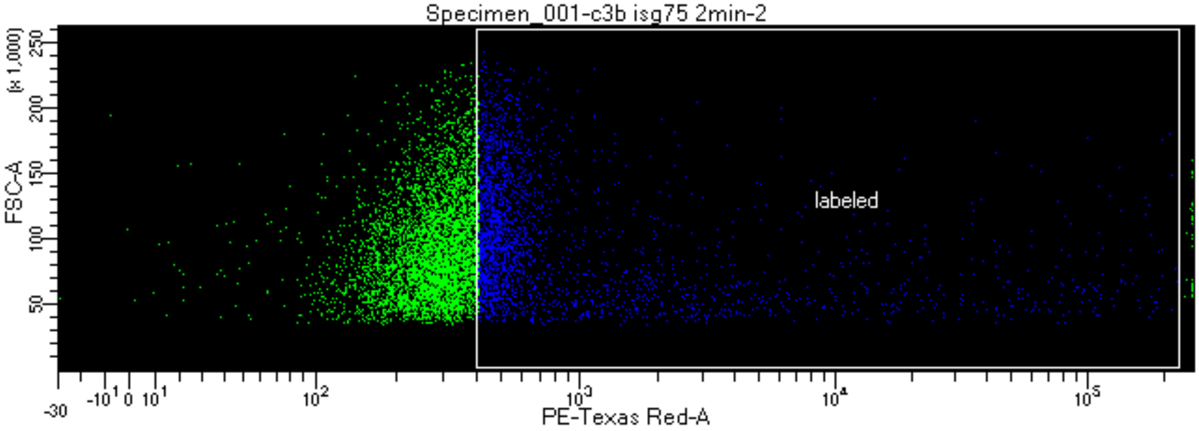

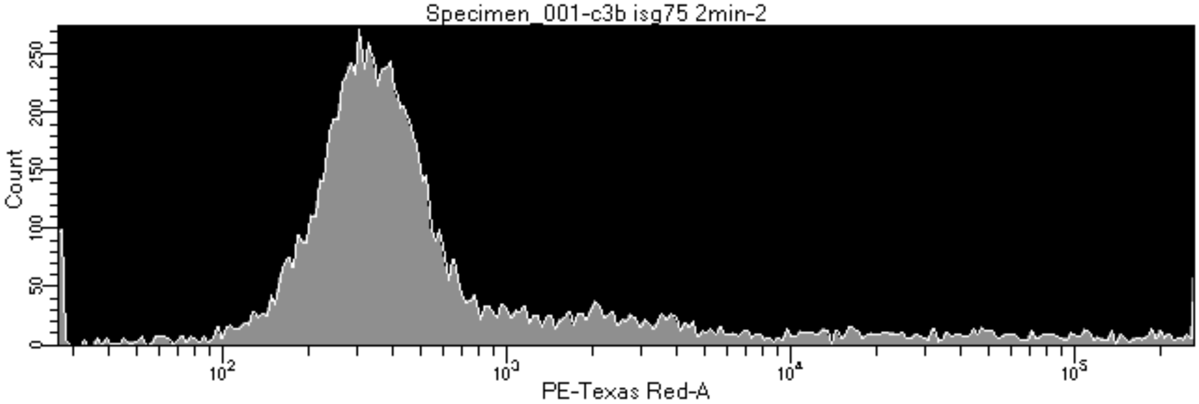

Tube: c3b isg75 2min-2

Population	#Events	%Parent	%Total
All Events	10,000	####	100.0
cells	6,964	69.6	69.6
singlets	6,816	97.9	68.2
labele	2,280	33.5	22.8

BD FACSDiva 8.0.1

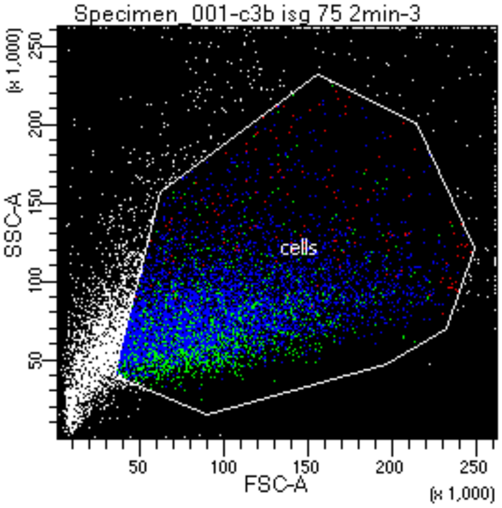

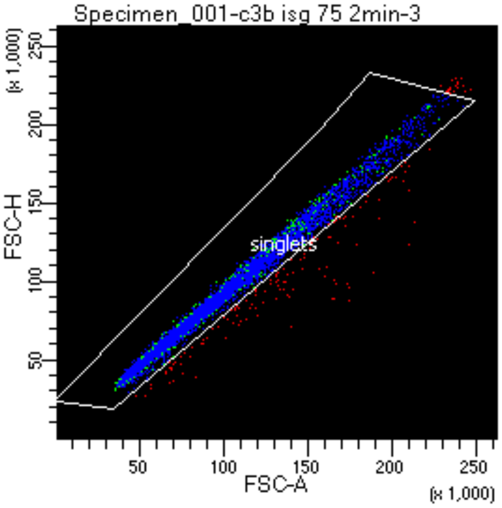

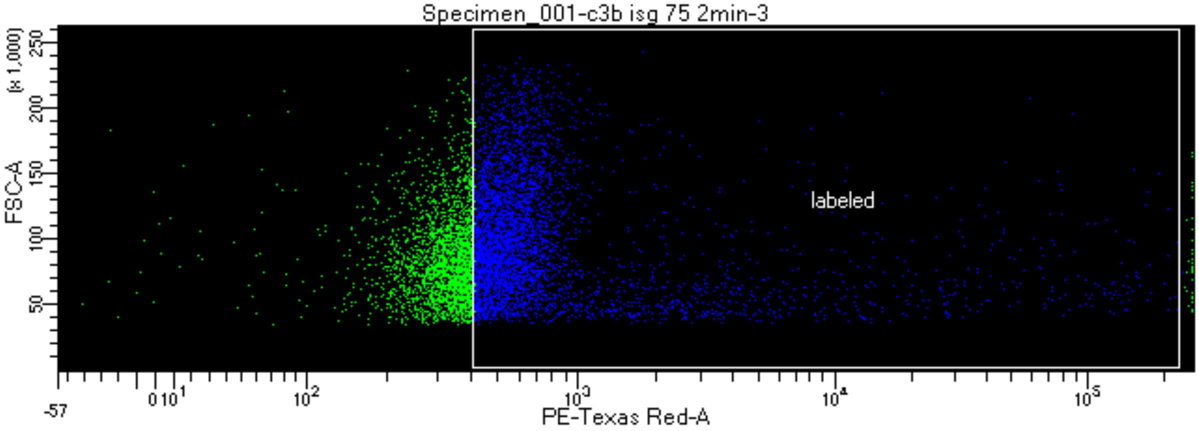

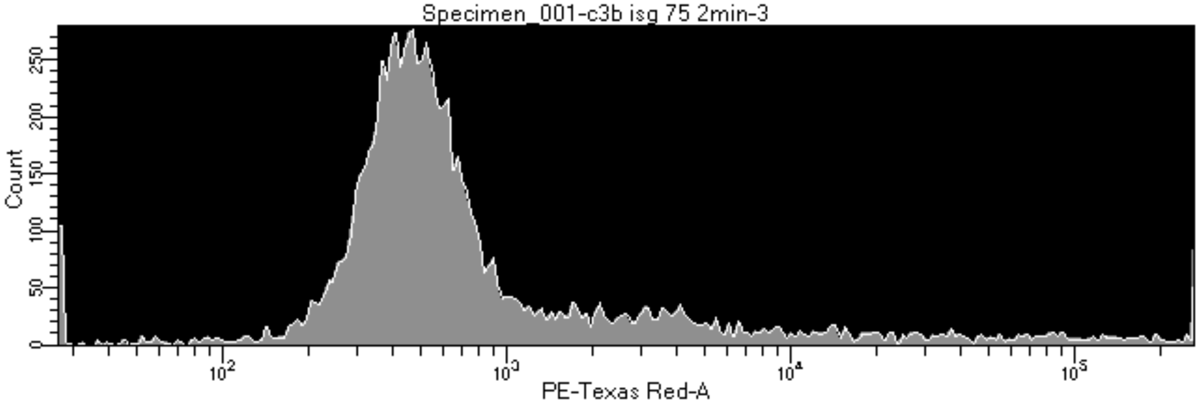

Tube: c3b isg 75 2min-3

Population	#Events	%Parent	%Total
All Events	10,000	####	100.0
cells	6,989	69.9	69.9
singlets	6,829	97.7	68.3
label[illegible]	4,337	63.5	43.4

BD FACSDiva 8.0.1

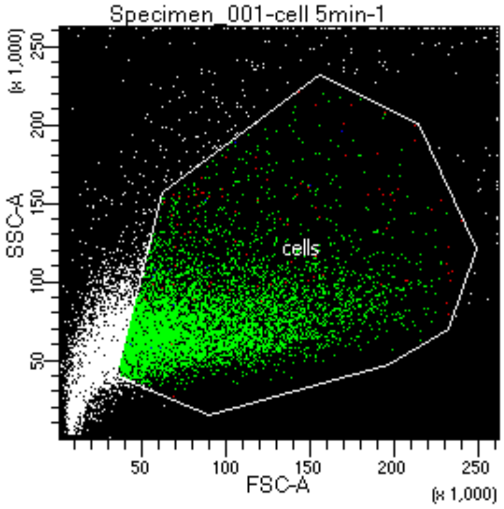

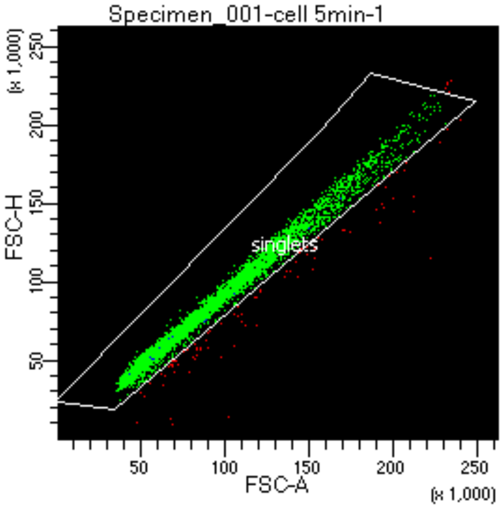

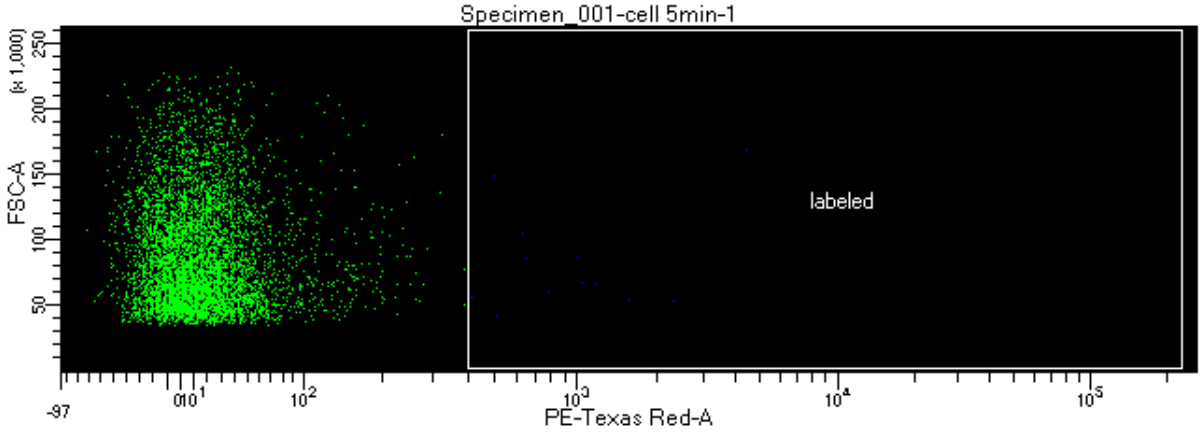

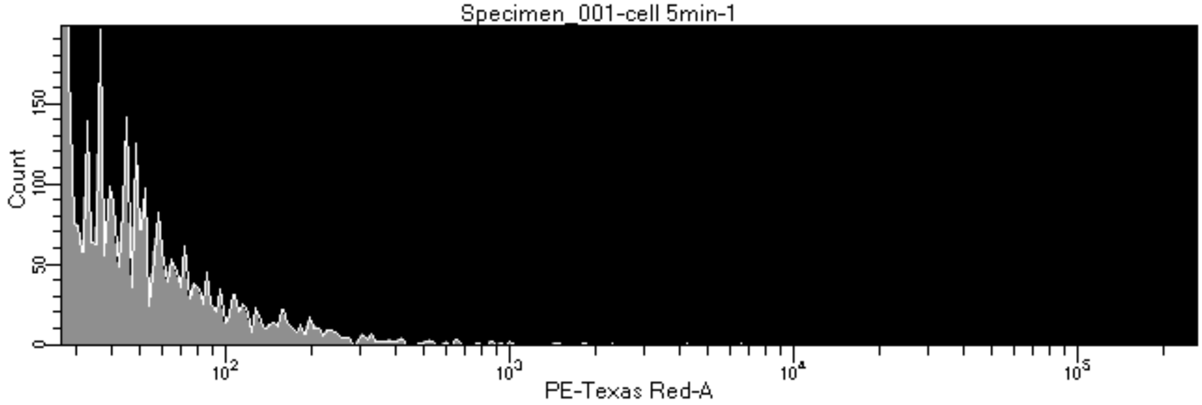

Tube: cell 5min-1

Population	#Events	%Parent	%Total
All Events	10,000	####	100.0
cells	5,577	55.8	55.8
singlets	5,474	98.2	54.7
labele	12	0.2	0.1

BD FACSDiva 8.0.1

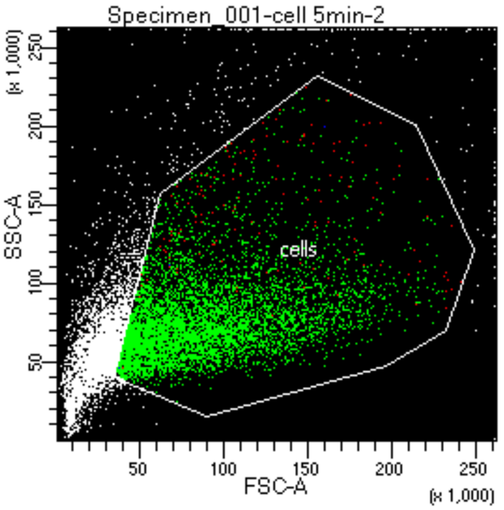

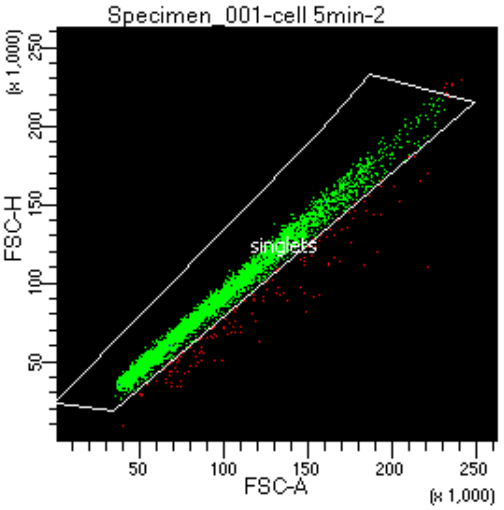

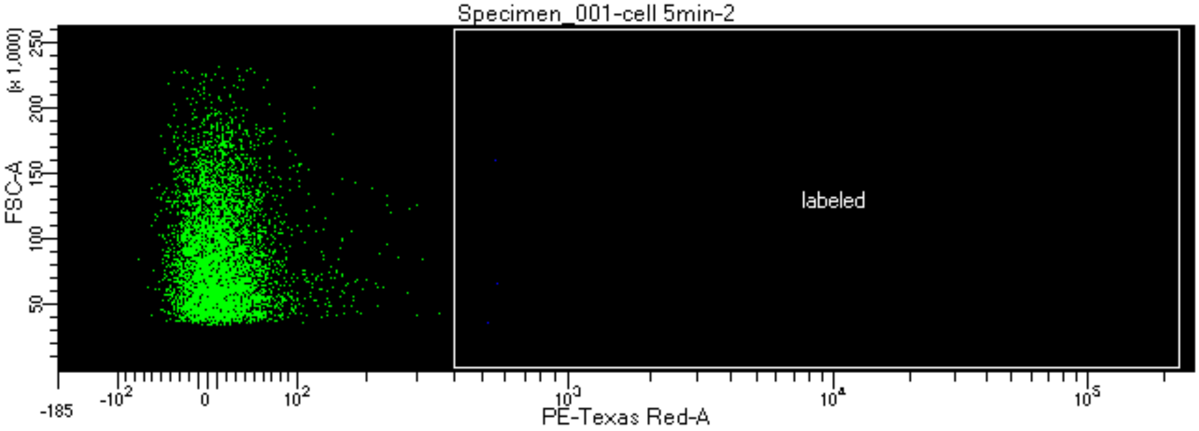

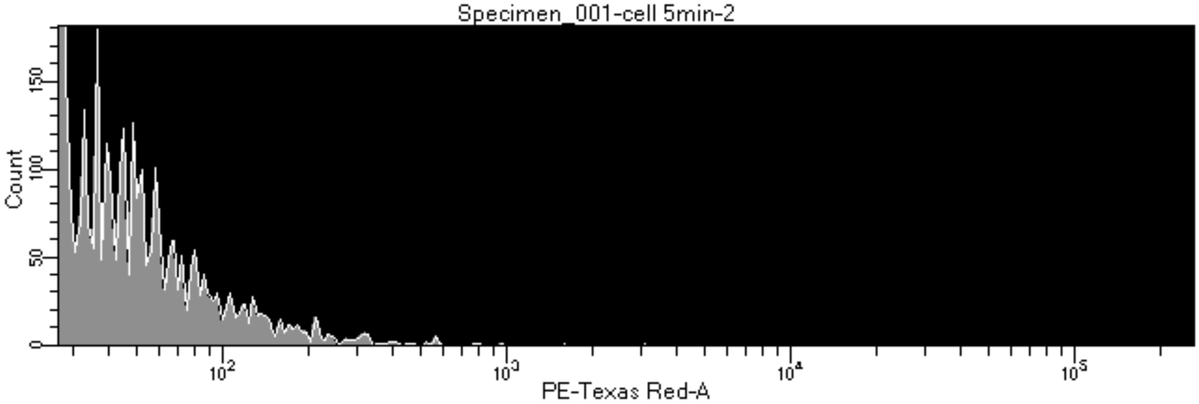

Tube: cell 5min-2

Population	#Events	%Parent	%Total
All Events	10,000	####	100.0
cells	5,716	57.2	57.2
singlets	5,567	97.4	55.7
labele	3	0.1	0.0

BD FACSDiva 8.0.1

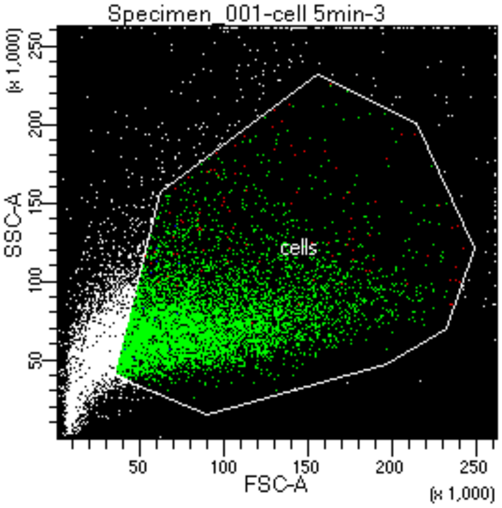

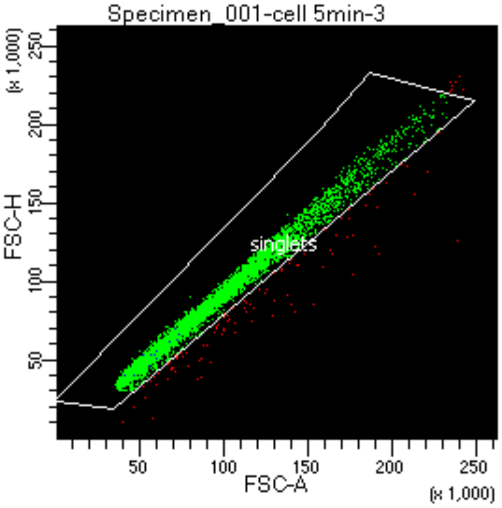

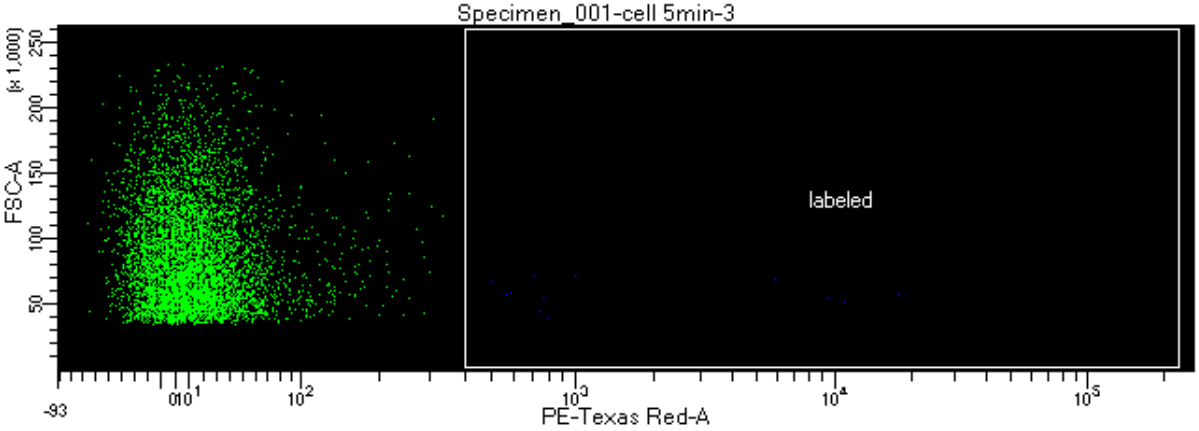

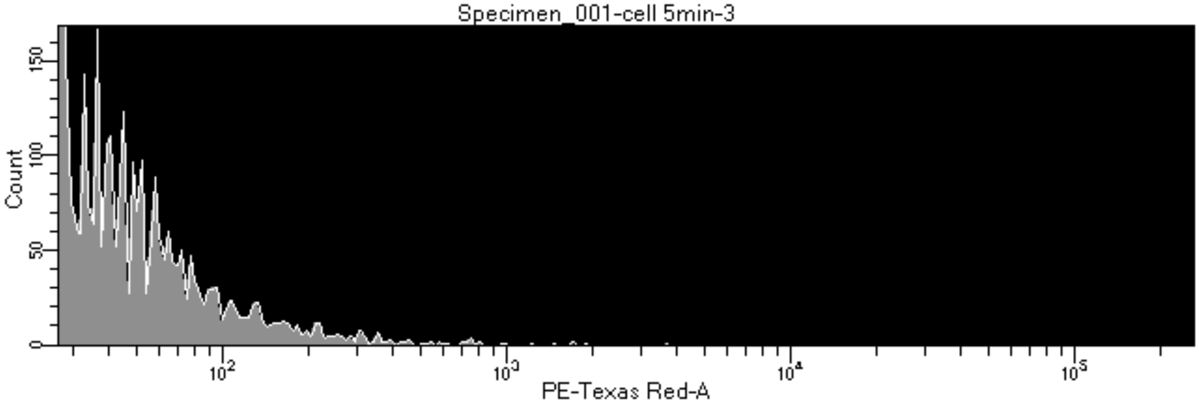

Tube: cell 5min-3

Population	#Events	%Parent	%Total
All Events	10,000	####	100.0
cells	5,786	57.9	57.9
singlets	5,670	98.0	56.7
label(	12	0.2	0.1

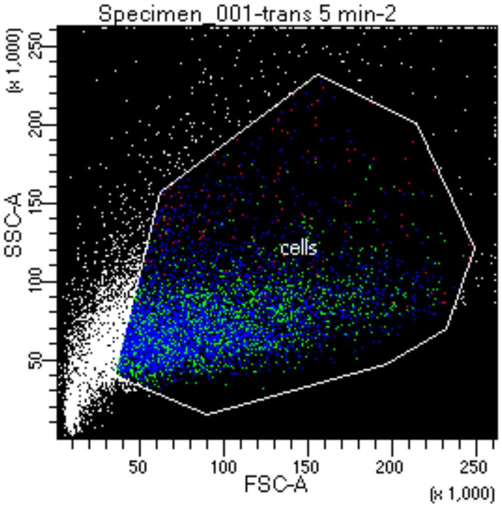

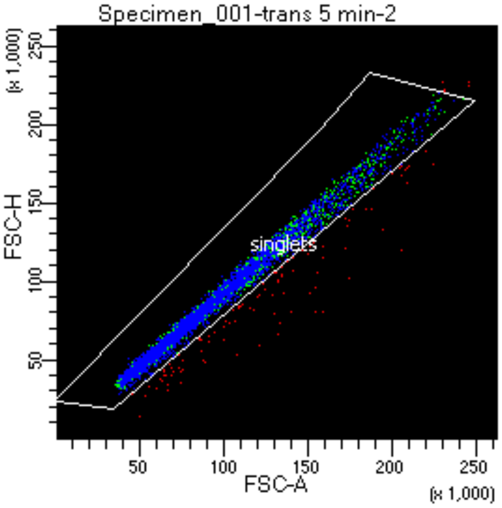

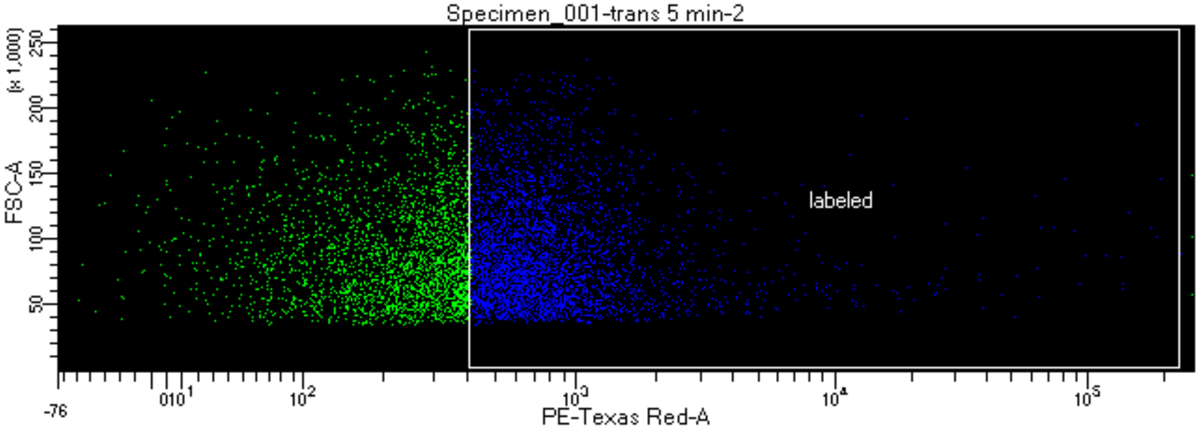

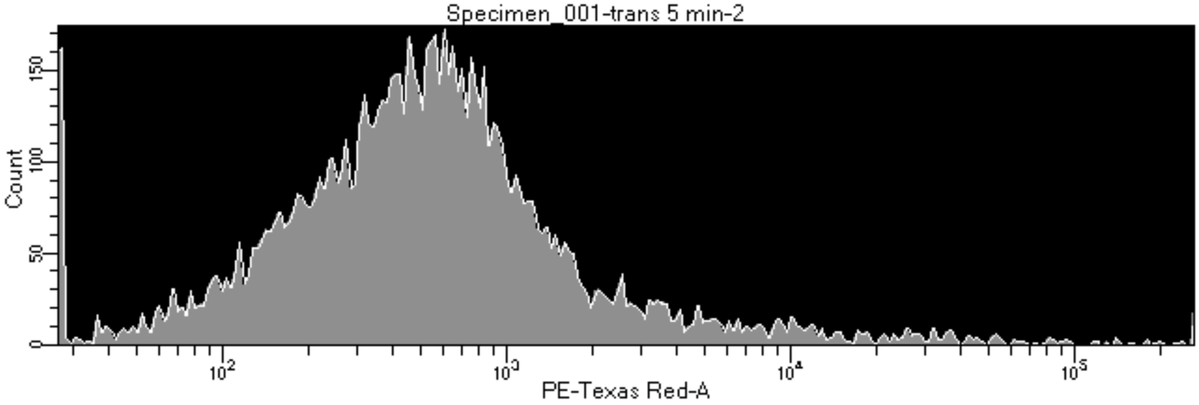

Tube: trans 5 min-2

Population	#Events	%Parent	%Total
All Events	10,000	####	100.0
cells	6,134	61.3	61.3
singlets	6,014	98.0	60.1
label‹	3,525	58.6	35.2

BD FACSDiva 8.0.1

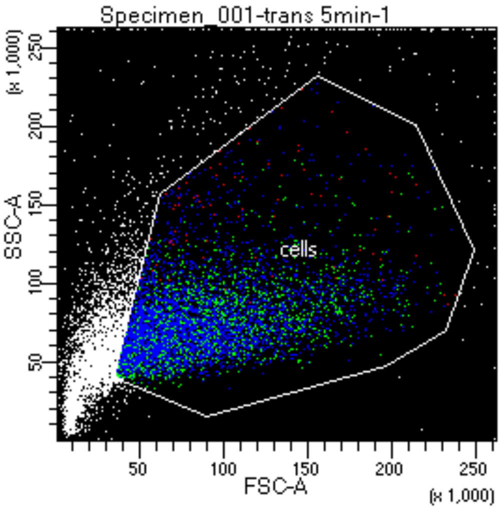

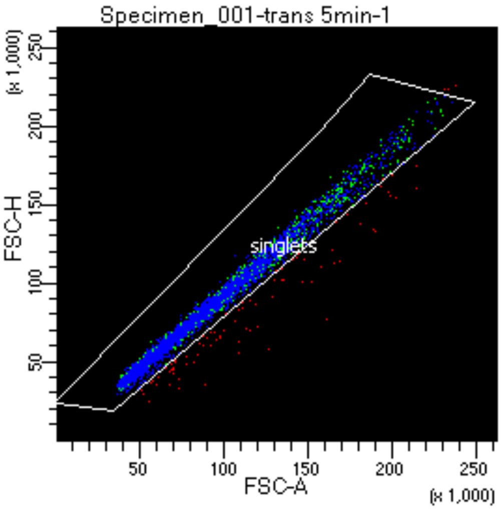

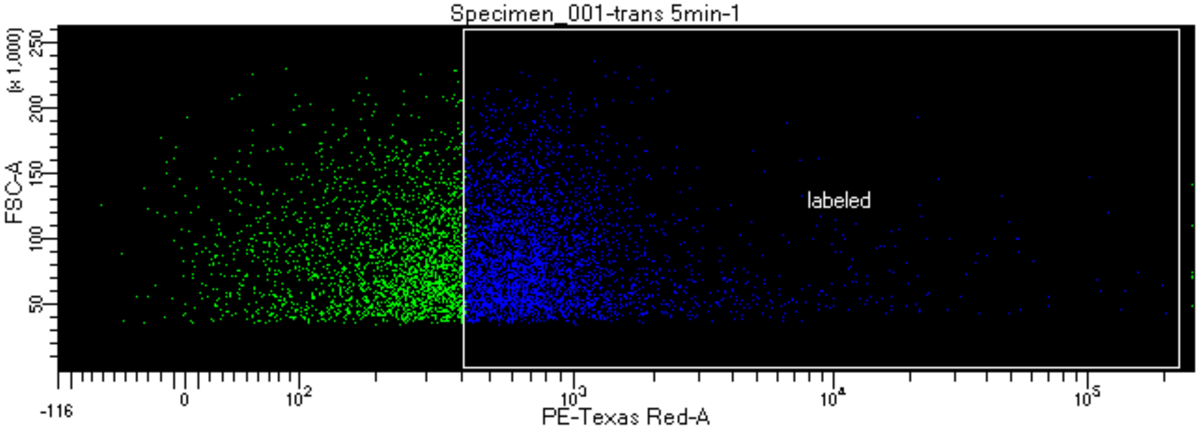

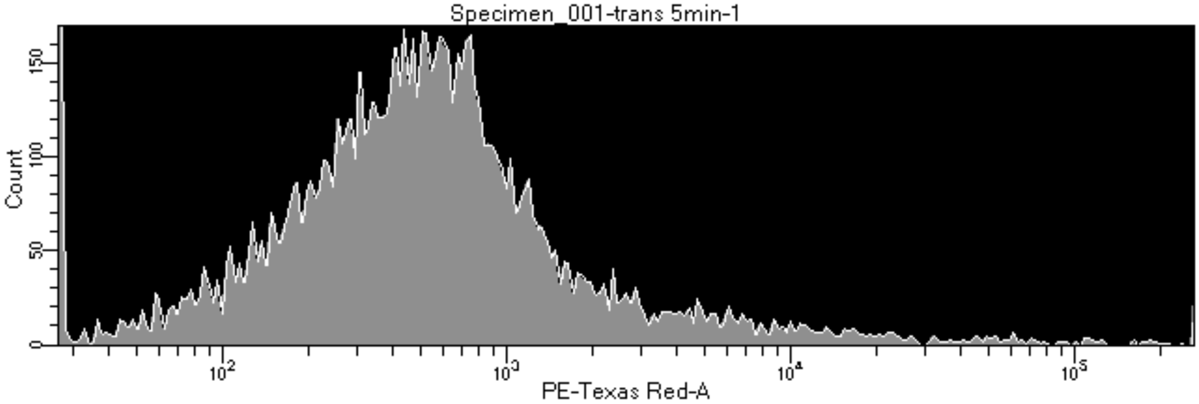

Tube: trans 5min-1

Population	#Events	%Parent	%Total
All Events	10,000	####	100.0
cells	5,854	58.5	58.5
singlets	5,765	98.5	57.6
labele	3,396	58.9	34.0

BD FACSDiva 8.0.1

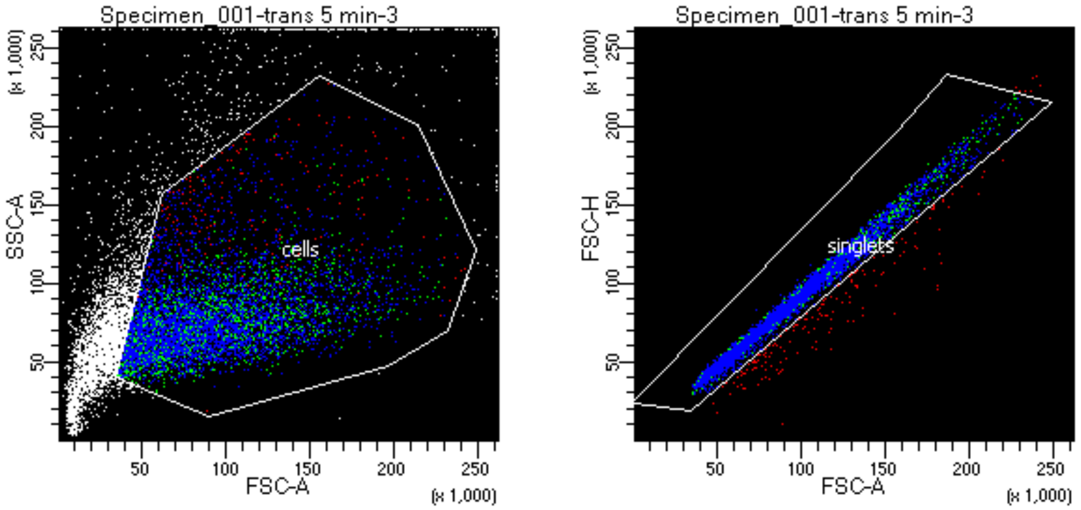

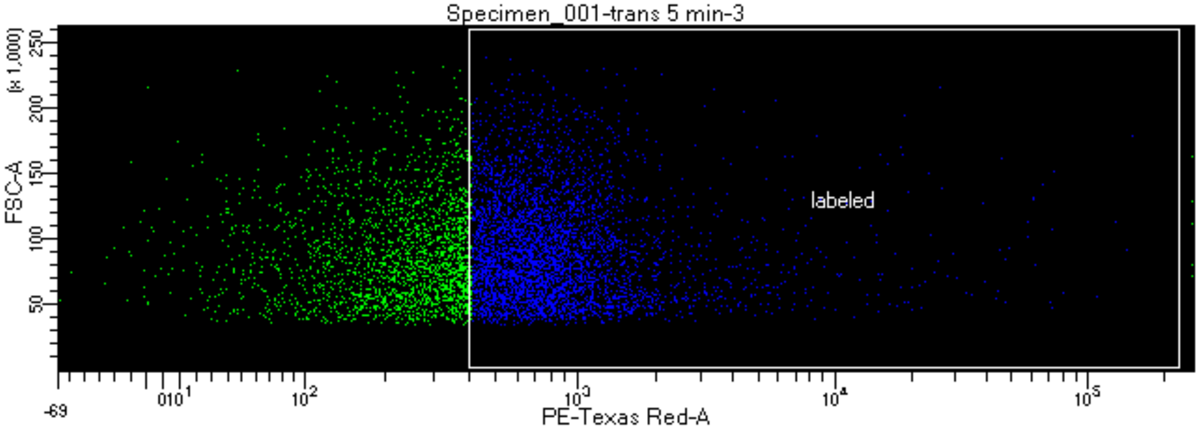

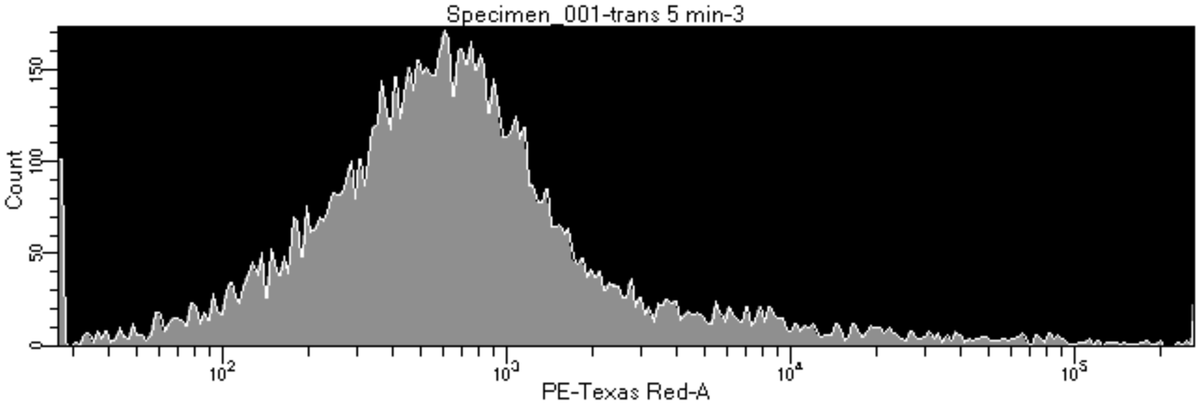

Tube: trans 5 min-3

Population	#Events	%Parent	%Total
All Events	10,000	####	100.0
cells	6,218	62.2	62.2
singlets	6,043	97.2	60.4
label(	3,898	64.5	39.0

BD FACSDiva 8.0.1

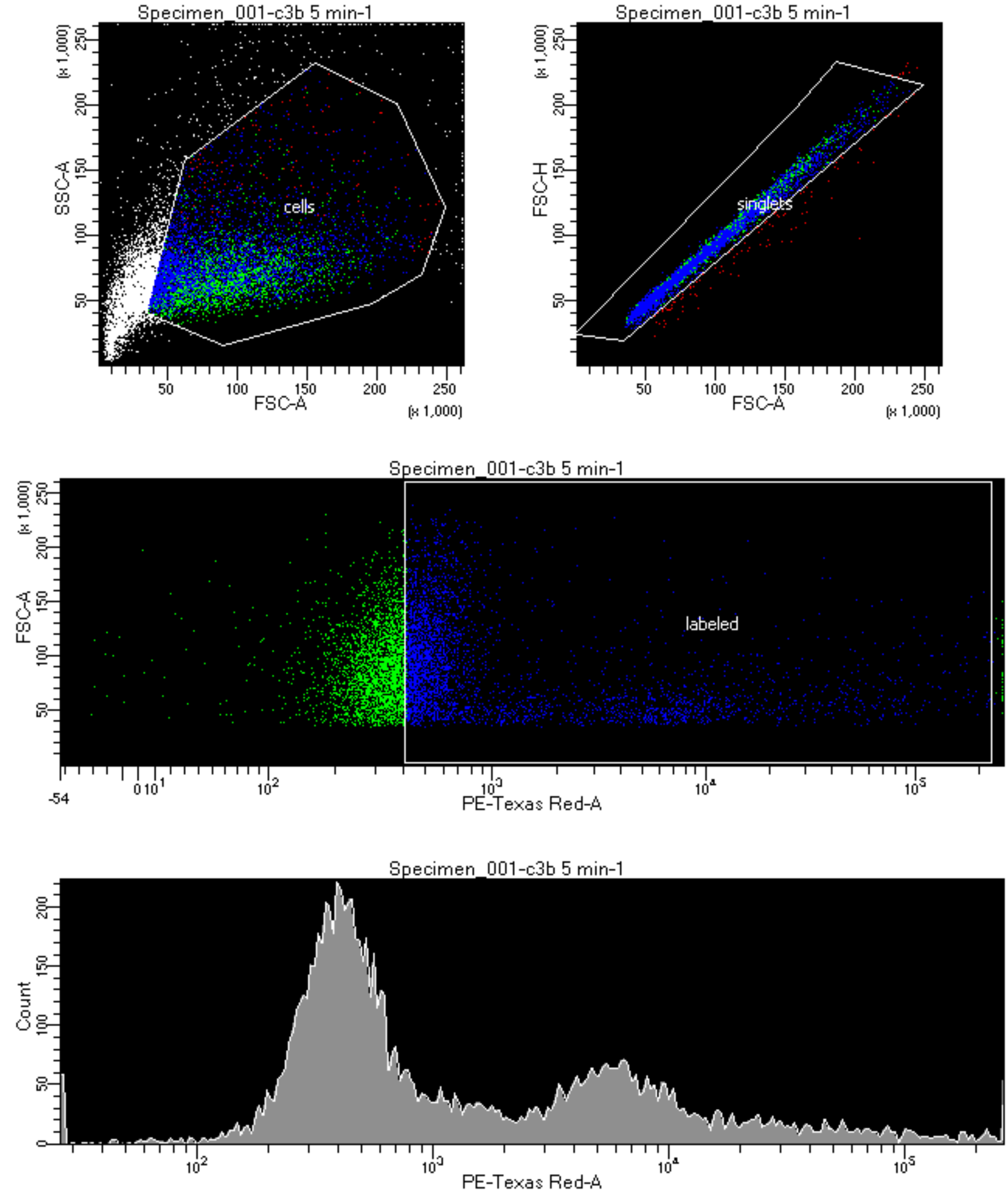

Tube: c3b 5 min-1

Population	#Events	%Parent	%Total
All Events	10,000	####	100.0
cells	5,865	58.7	58.7
singlets	5,741	97.9	57.4
labele	3,207	55.9	32.1

BD FACSDiva 8.0.1

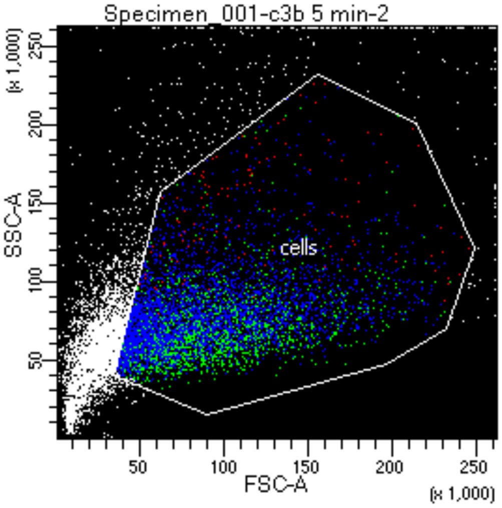

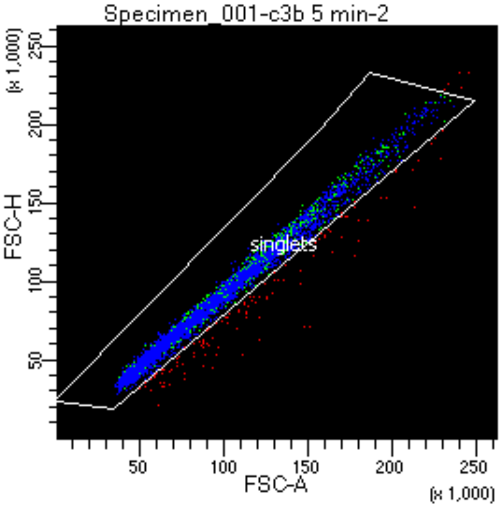

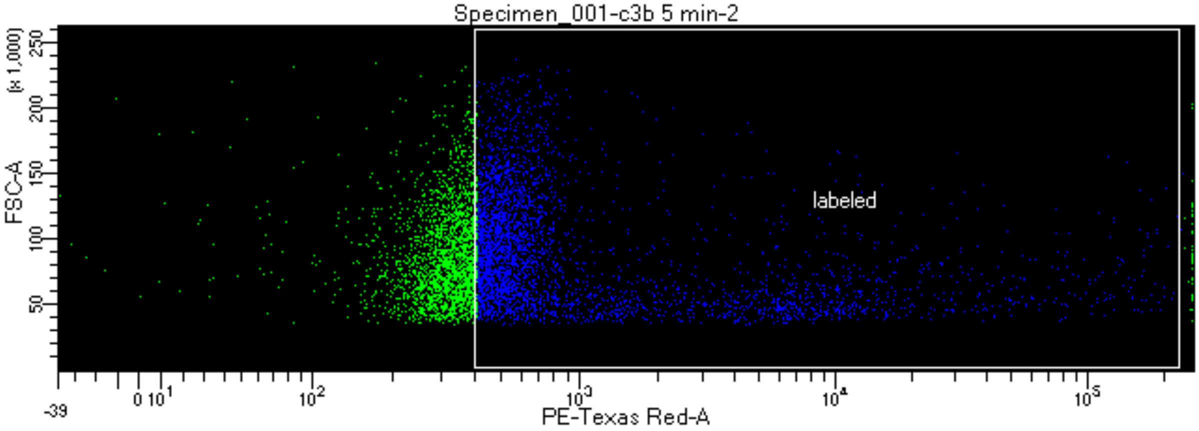

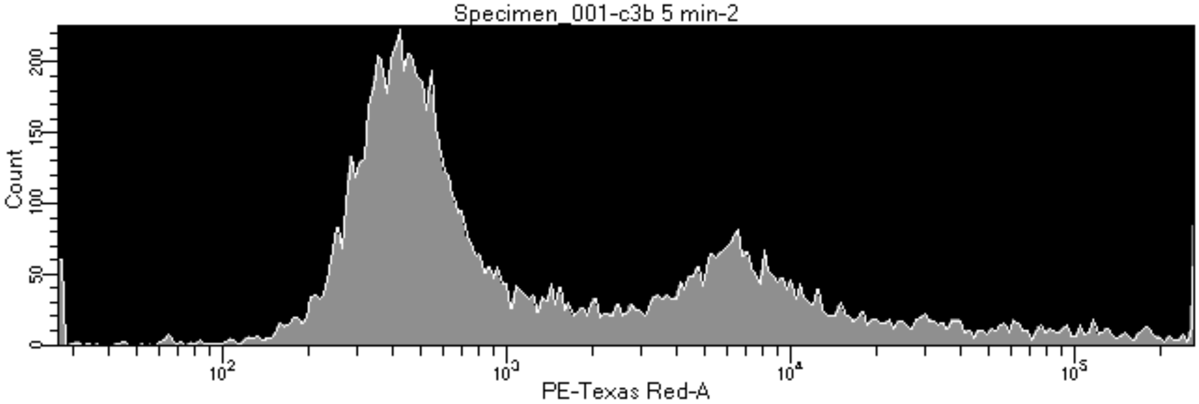

Tube: c3b 5 min-2

Population	#Events	%Parent	%Total
All Events	10,000	####	100.0
cells	5,874	58.7	58.7
singlets	5,744	97.8	57.4
label€	3,451	60.1	34.5

BD FACSDiva 8.0.1

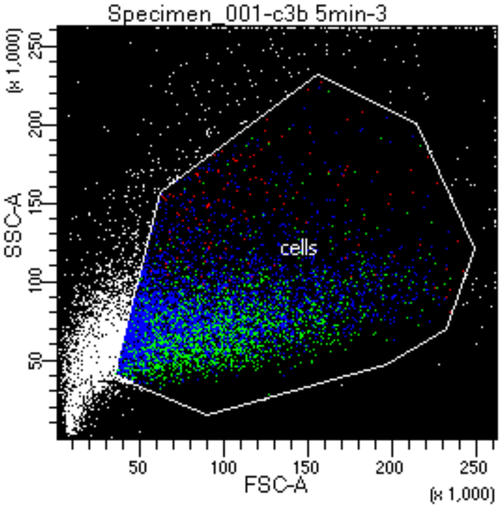

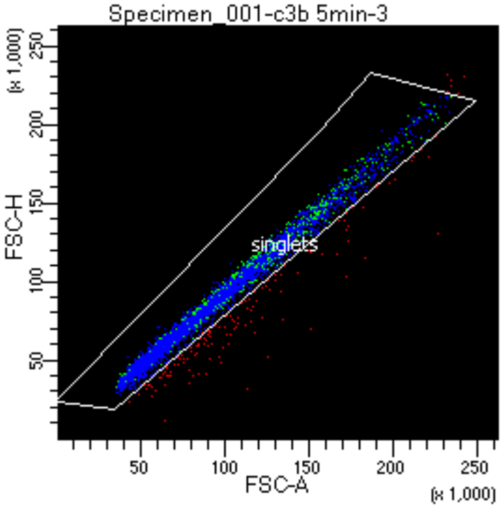

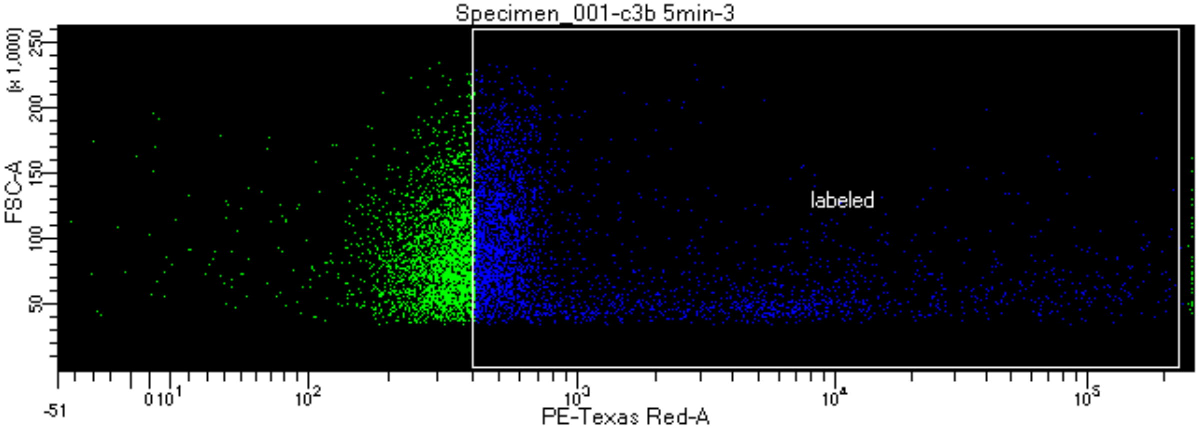

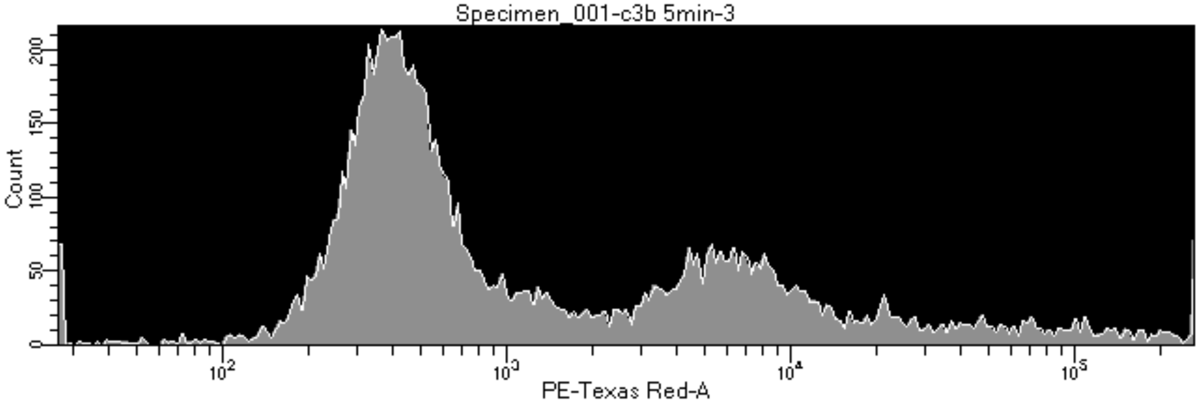

Tube: c3b 5min-3

Population	#Events	%Parent	%Total
All Events	10,000	####	100.0
cells	6,026	60.3	60.3
singlets	5,882	97.6	58.8
labele	3,175	54.0	31.8

BD FACSDiva 8.0.1

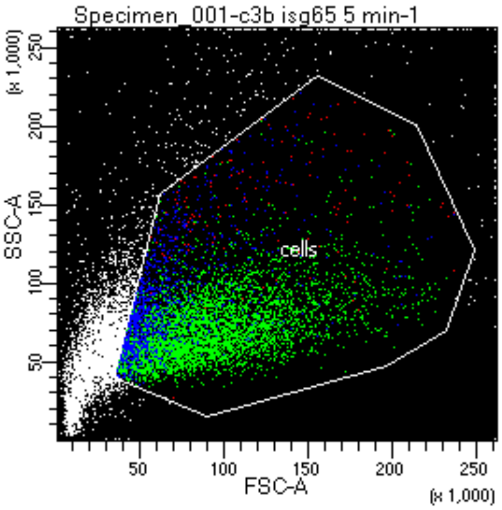

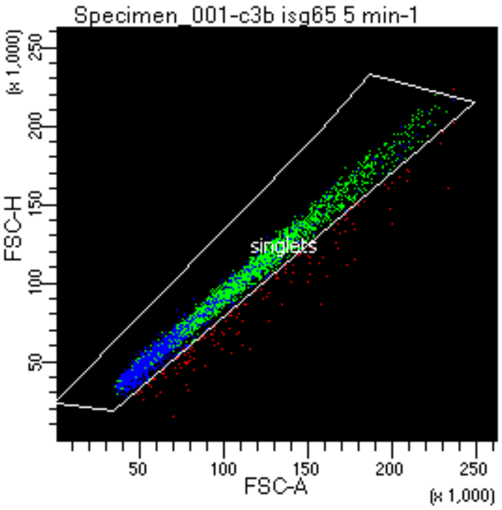

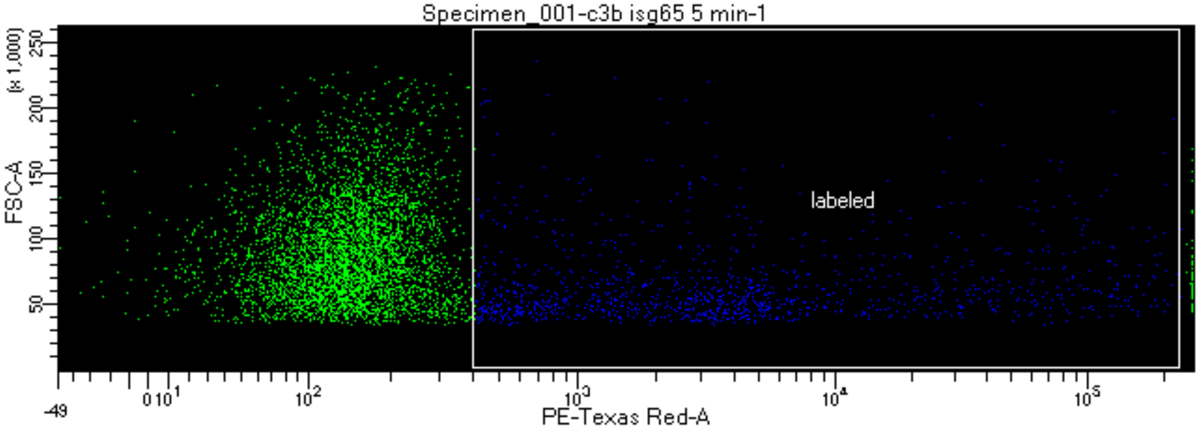

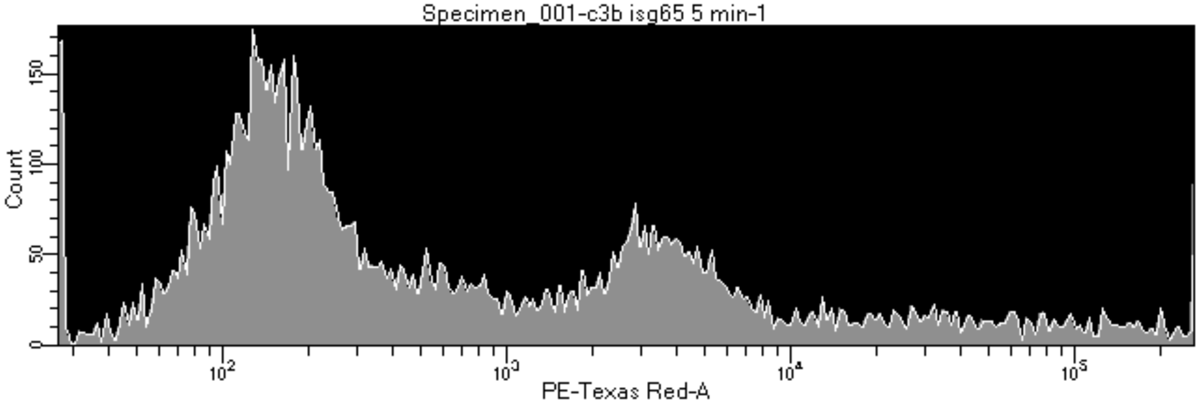

Tube: c3b isg65 5 min-1

Population	#Events	%Parent	%Total
All Events	10,000	####	100.0
cells	5,508	55.1	55.1
singlets	5,364	97.4	53.6
labele	1,136	21.2	11.4

BD FACSDiva 8.0.1

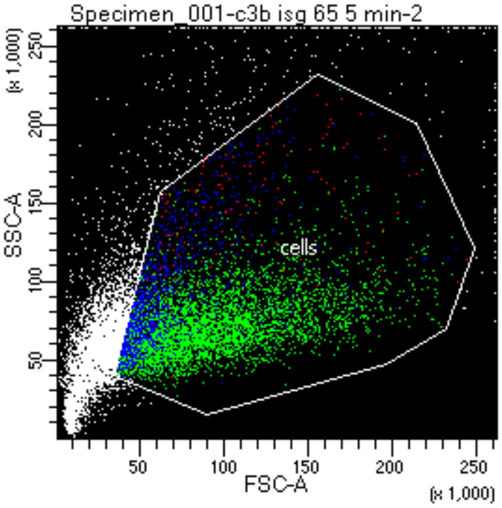

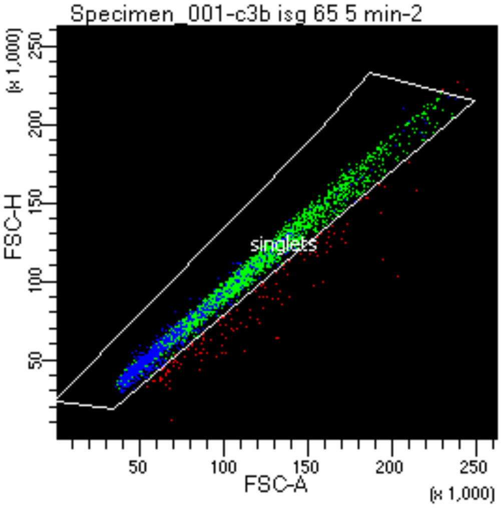

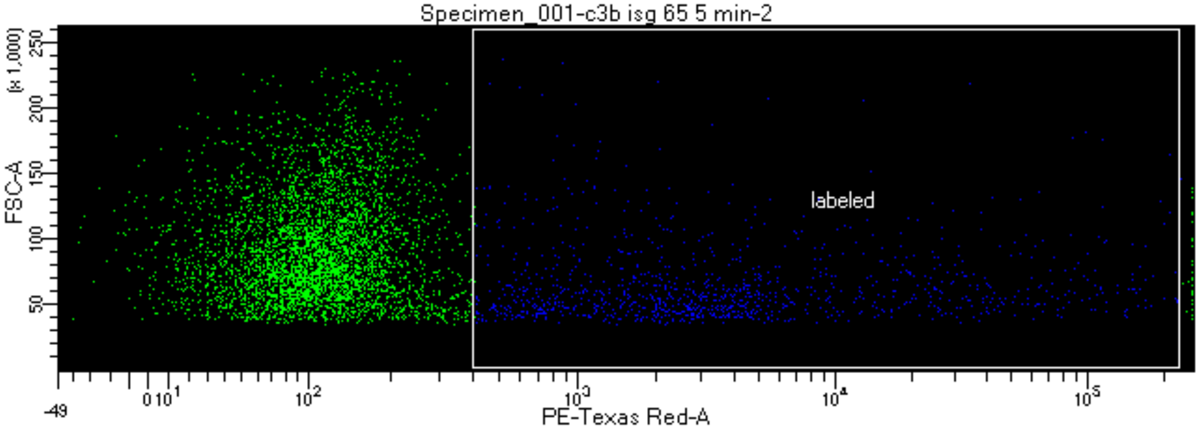

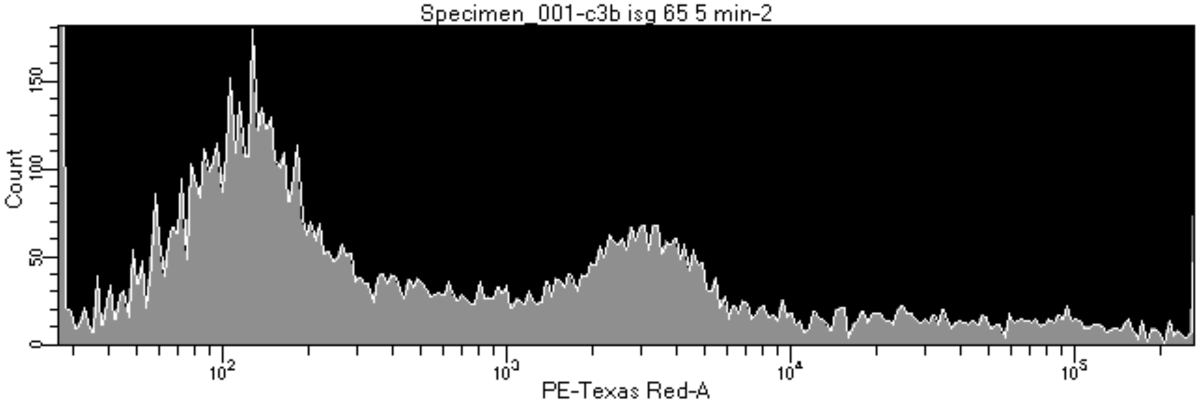

Tube: c3b isg 65 5 min-2

Population	#Events	%Parent	%Total
All Events	10,000	####	100.0
cells	5,297	53.0	53.0
singlets	5,164	97.5	51.6
labele	1,036	20.1	10.4

BD FACSDiva 8.0.1

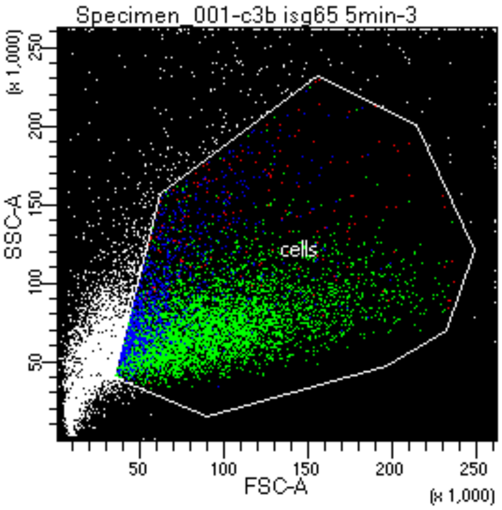

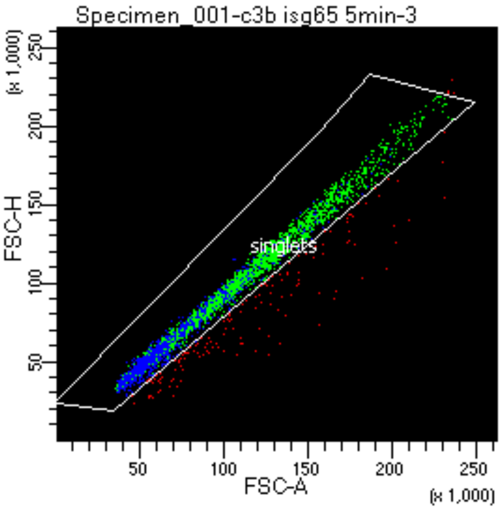

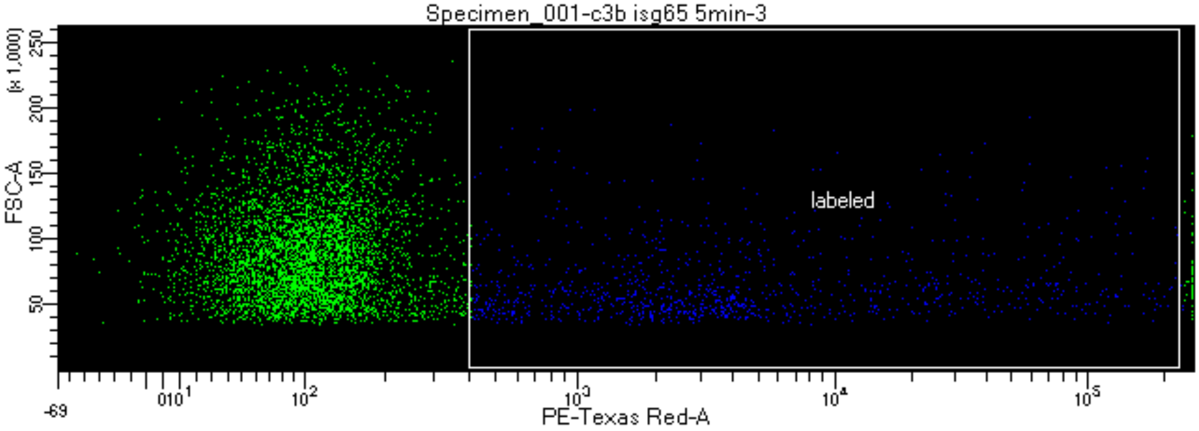

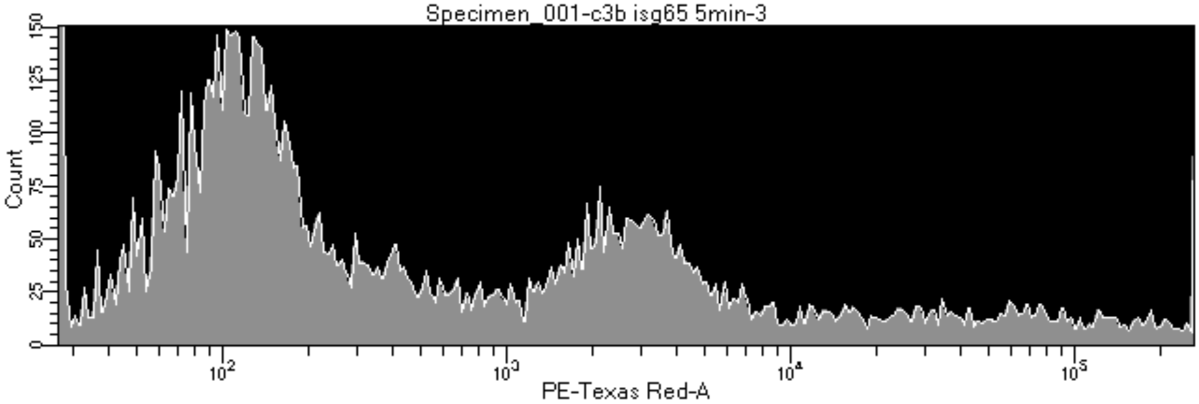

Tube: c3b isg65 5min-3

Population	#Events	%Parent	%Total
All Events	10,000	####	100.0
cells	5,493	54.9	54.9
singlets	5,332	97.1	53.3
label€	983	18.4	9.8

BD FACSDiva 8.0.1

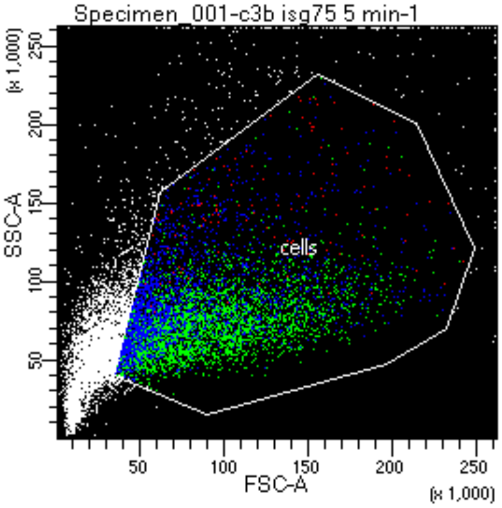

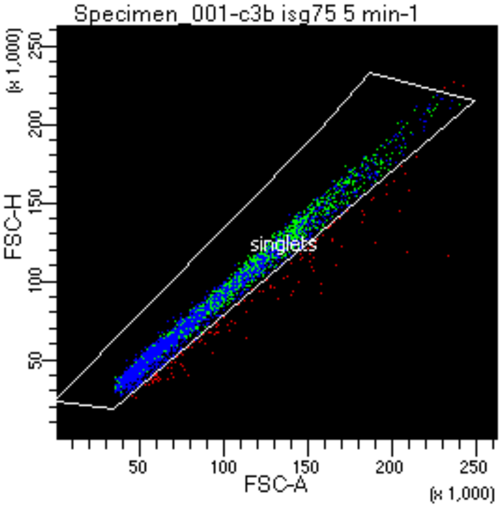

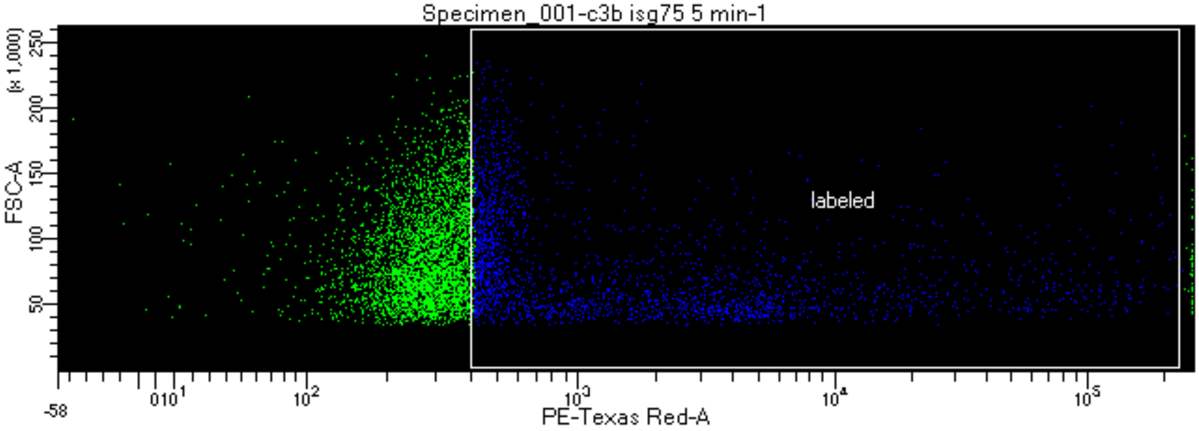

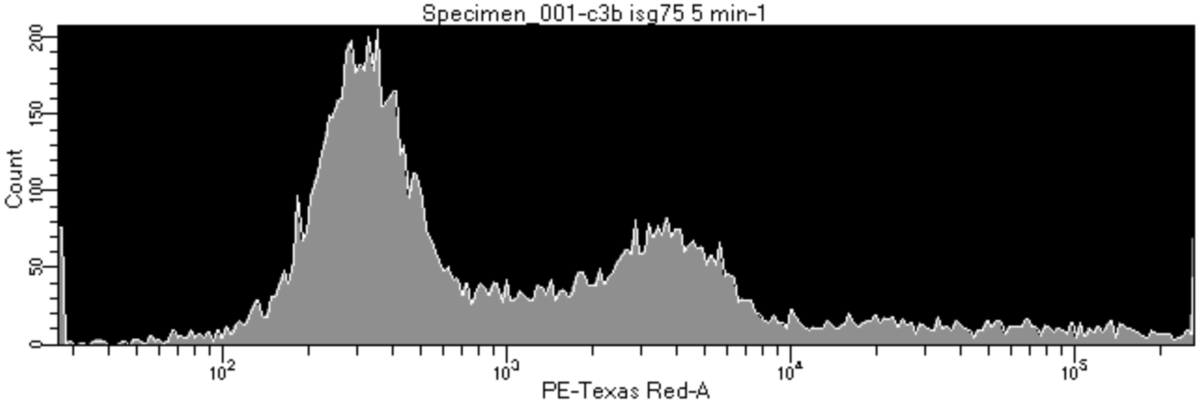

Tube: c3b isg75 5 min-1

Population	#Events	%Parent	%Total
All Events	10,000	####	100.0
cells	5,510	55.1	55.1
singlets	5,383	97.7	53.8
label€	2,013	37.4	20.1

BD FACSDiva 8.0.1

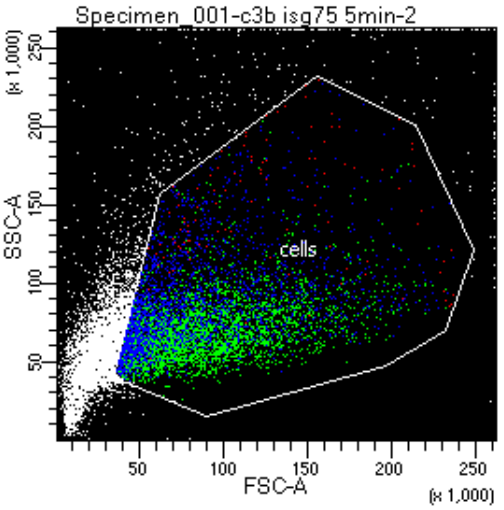

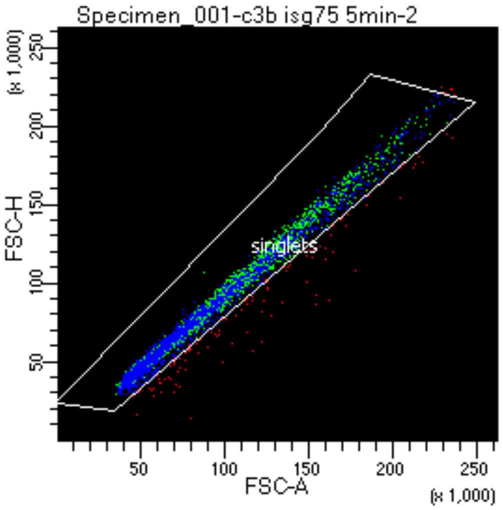

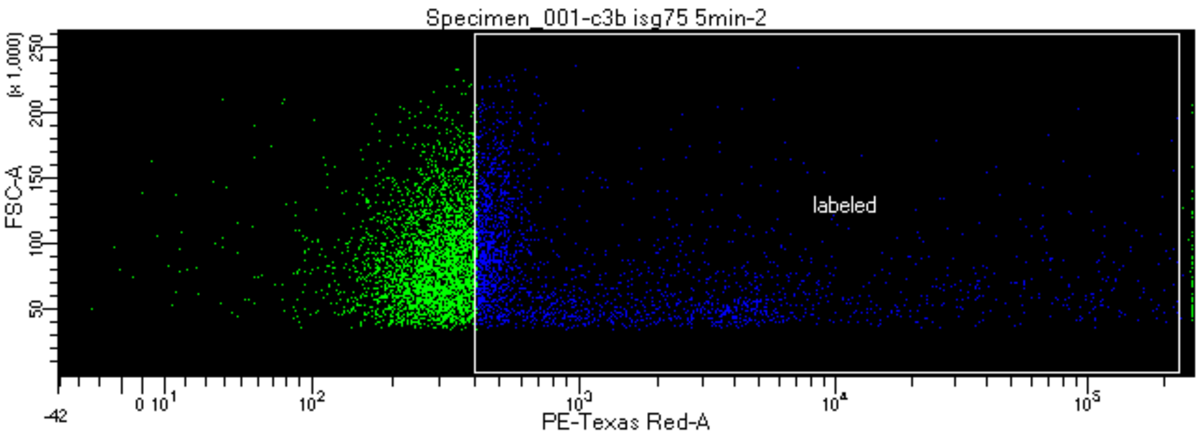

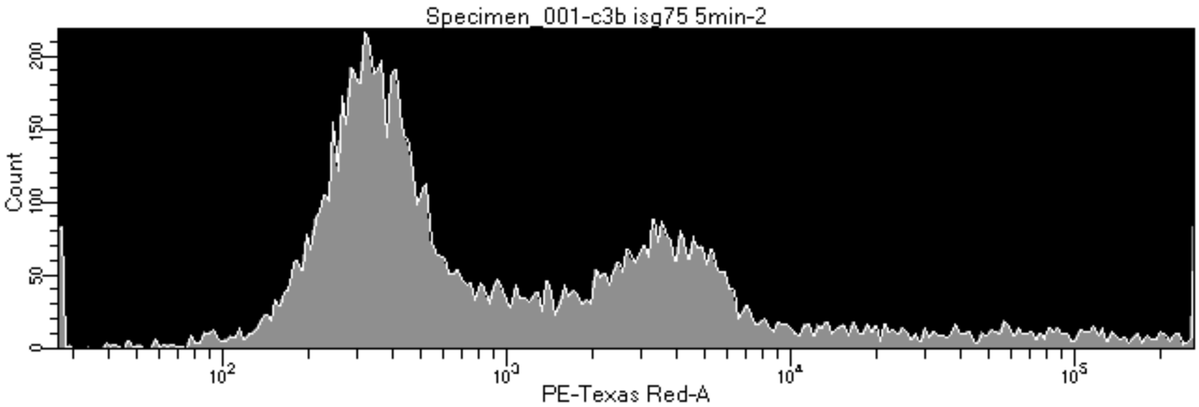

Tube: c3b isg75 5min-2

Population	#Events	%Parent	%Total
All Events	10,000	####	100.0
cells	5,495	54.9	54.9
singlets	5,372	97.8	53.7
labele	2,110	39.3	21.1

BD FACSDiva 8.0.1

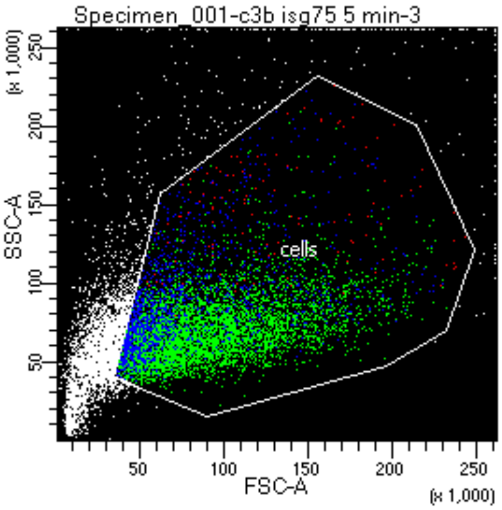

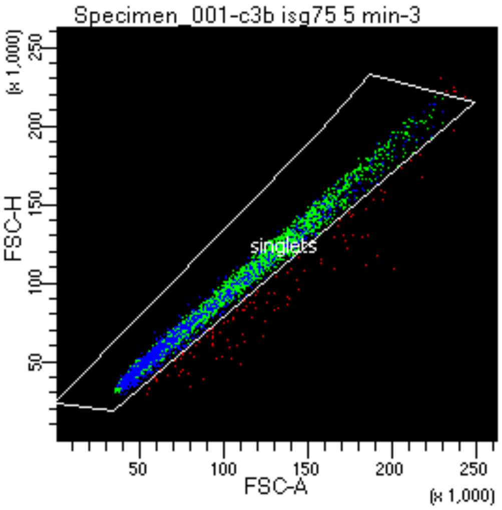

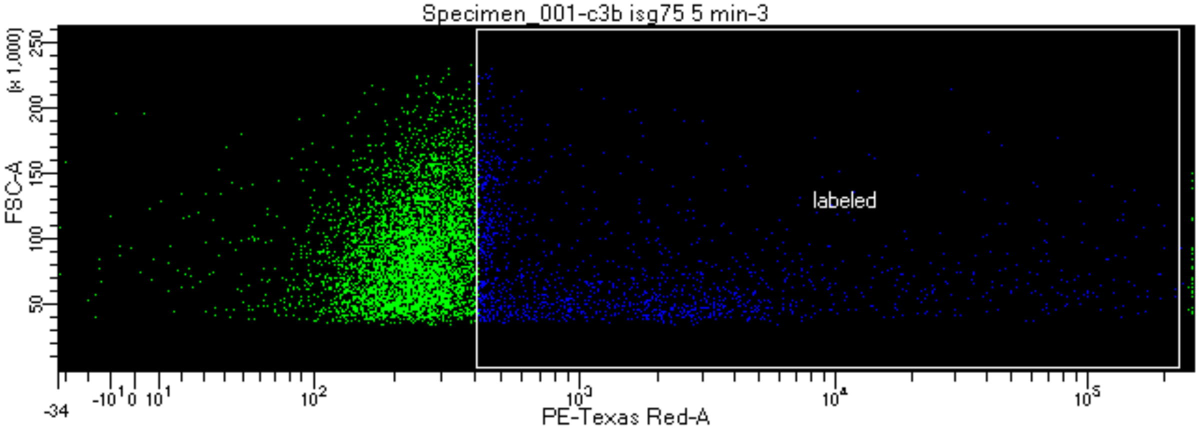

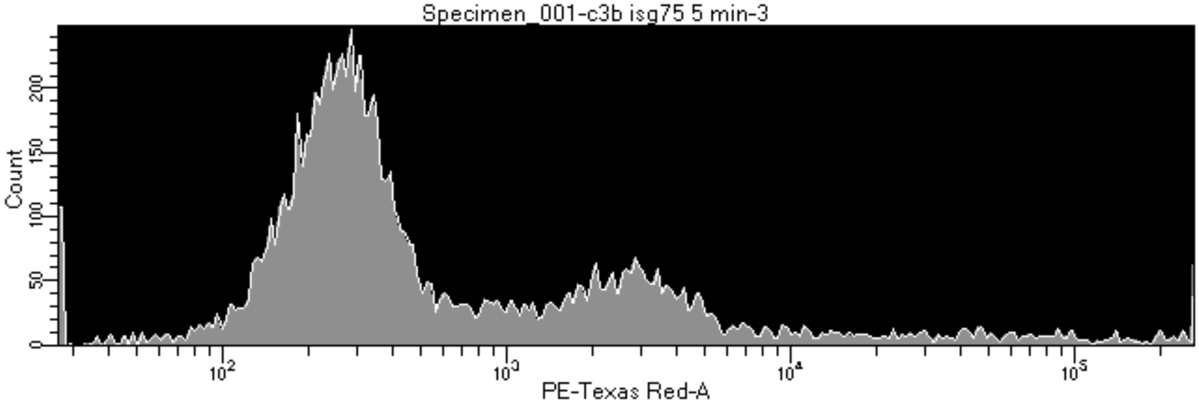

Tube: c3b isg75 5 min-3

Population	#Events	%Parent	%Total
All Events	10,000	####	100.0
cells	6,234	62.3	62.3
singlets	6,113	98.1	61.1
label(	1,370	22.4	13.7

BD FACSDiva 8.0.1

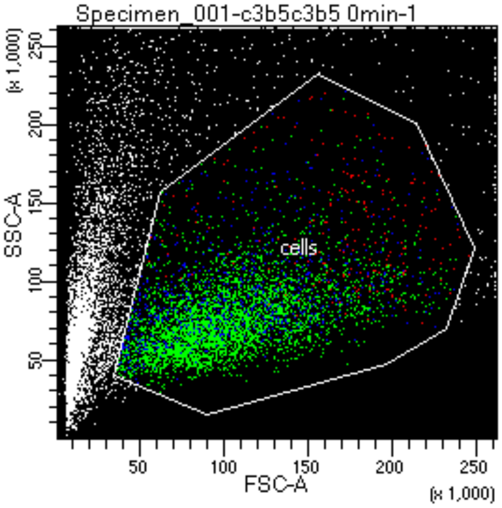

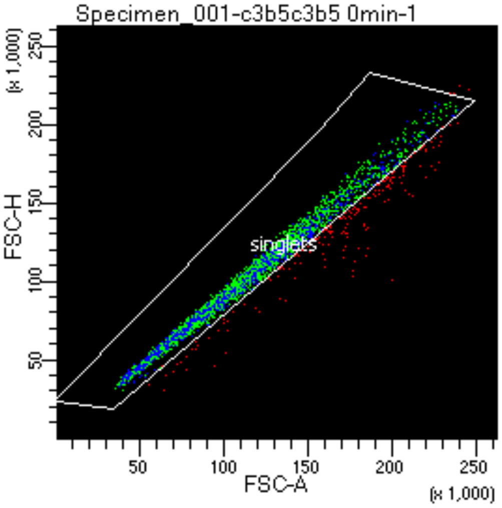

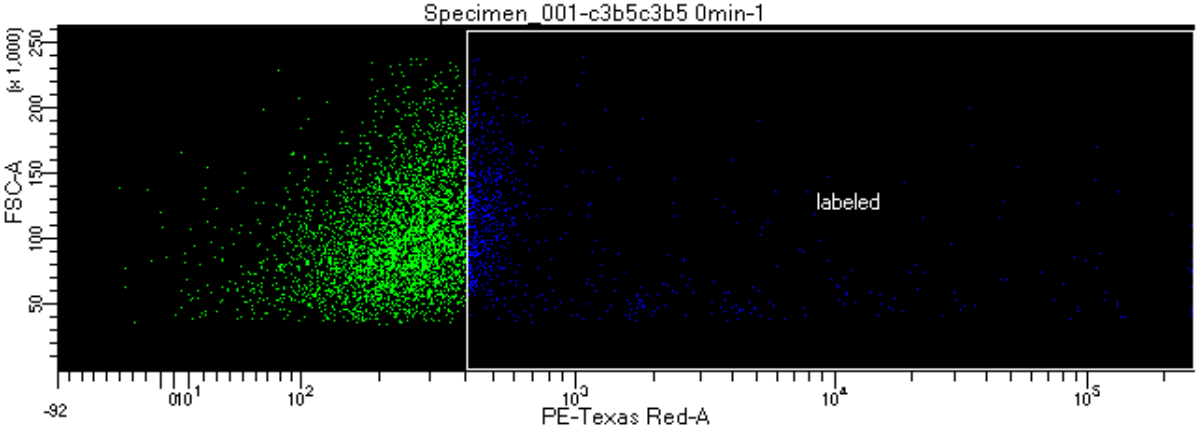

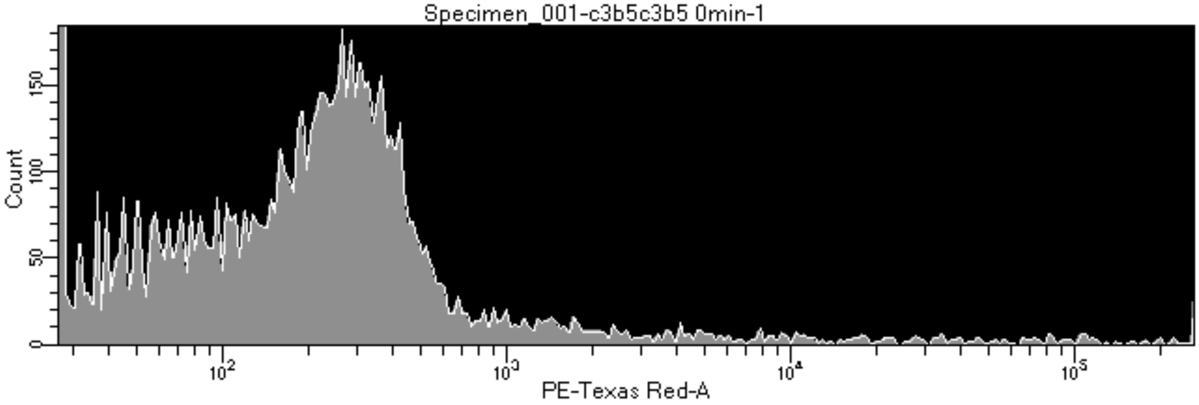

Tube: c3b5c3b5 0min-1

Population	#Events	%Parent	%Total
All Events	10,000	####	100.0
cells	4,481	44.8	44.8
singlets	4,266	95.2	42.7
labeled	723	16.9	7.2

BD FACSDiva 8.0.1

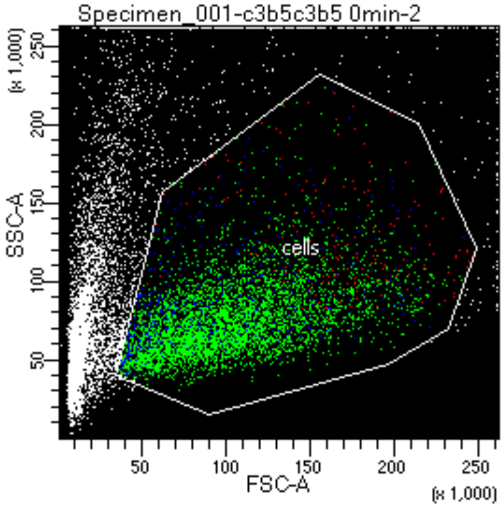

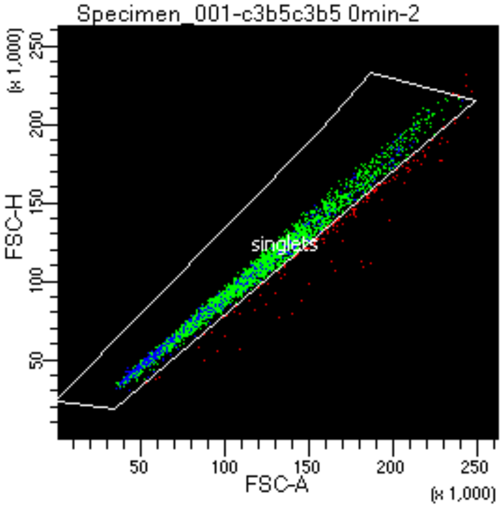

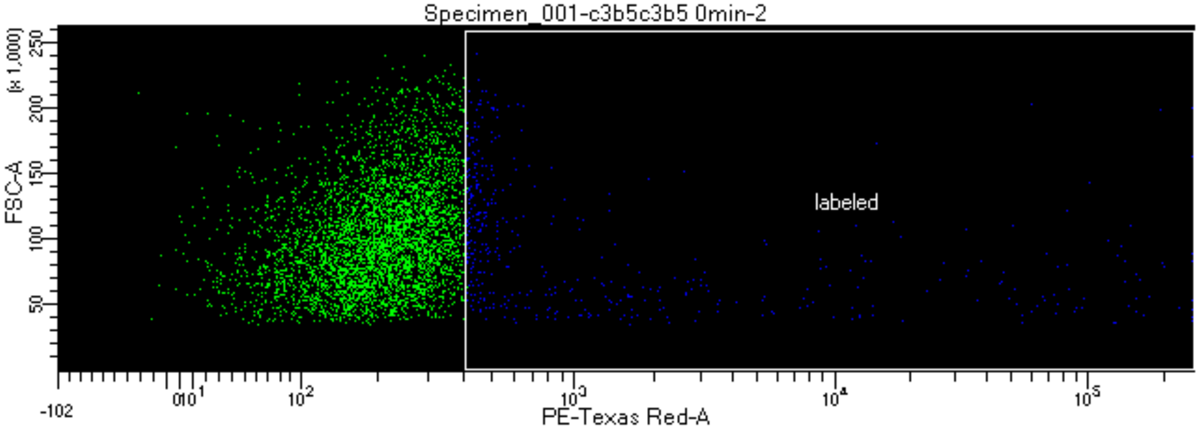

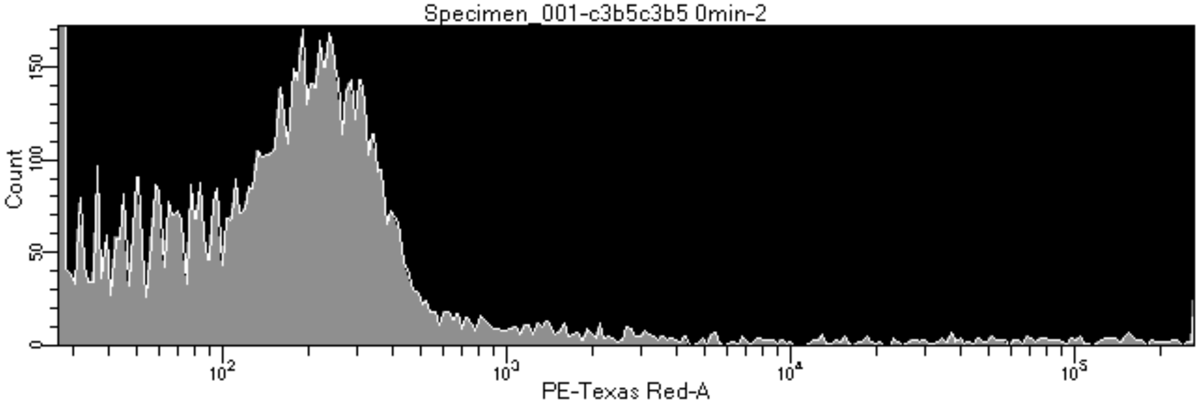

Tube: c3b5c3b5 0min-2

Population	#Events	%Parent	%Total
All Events	10,000	####	100.0
cells	4,678	46.8	46.8
singlets	4,511	96.4	45.1
labeled	424	9.4	4.2

BD FACSDiva 8.0.1

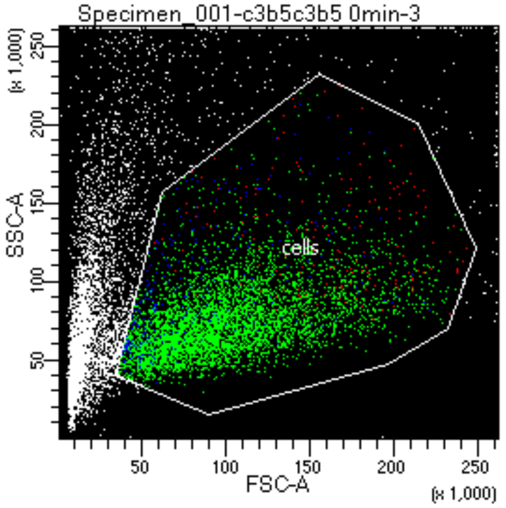

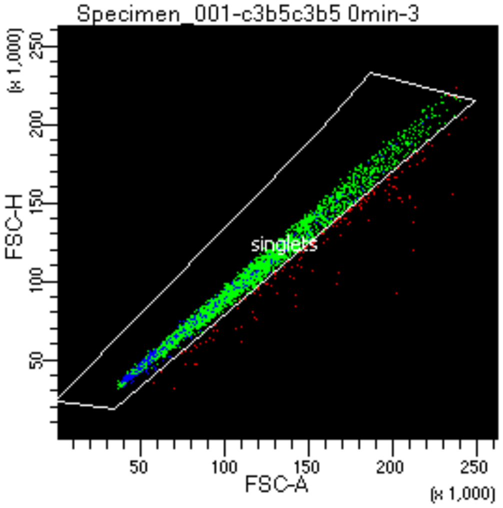

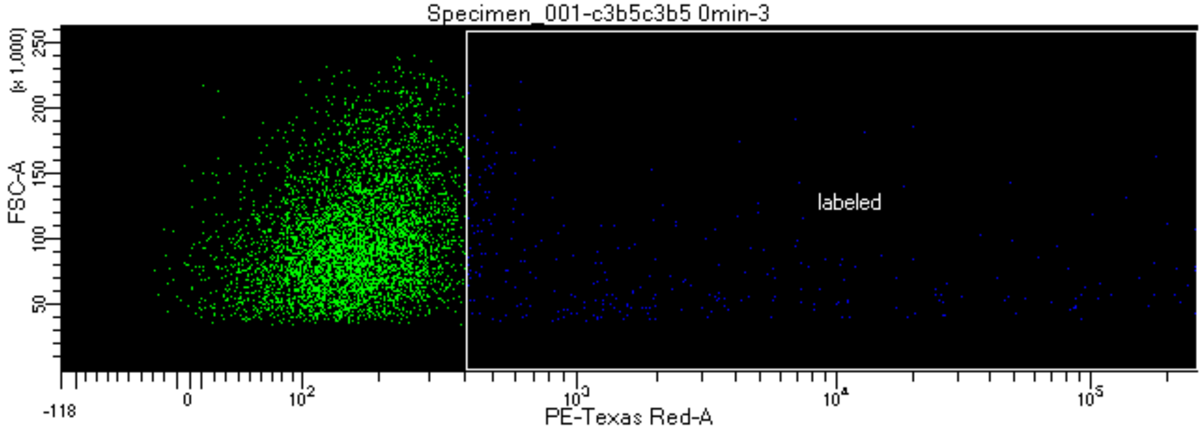

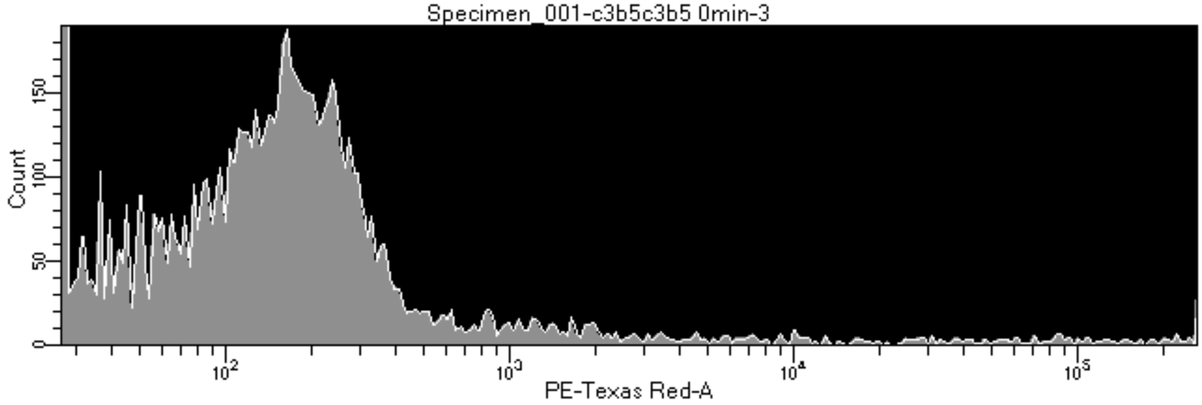

Tube: c3b5c3b5 0min-3

Population	#Events	%Parent	%Total
All Events	10,000	####	100.0
cells	4,990	49.9	49.9
singlets	4,816	96.5	48.2
labeled	282	5.9	2.8

BD FACSDiva 8.0.1

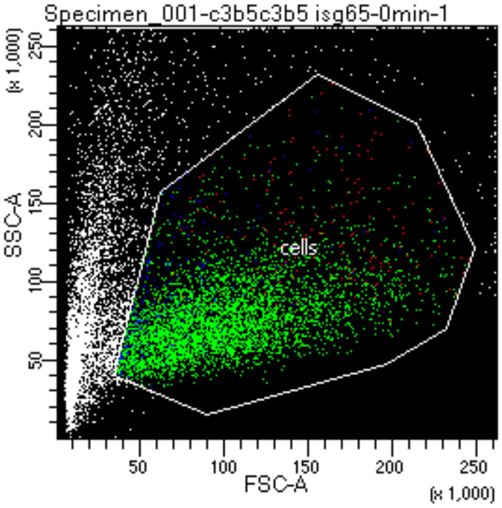

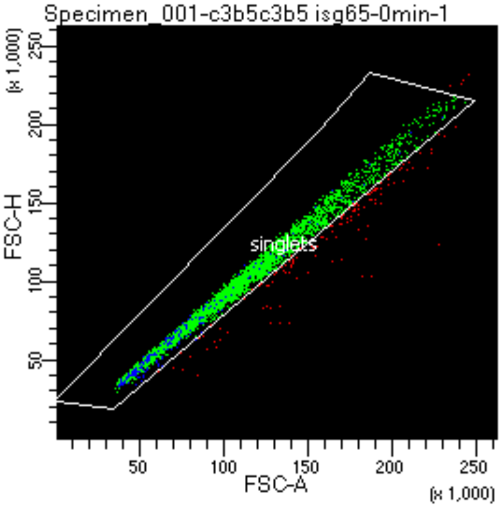

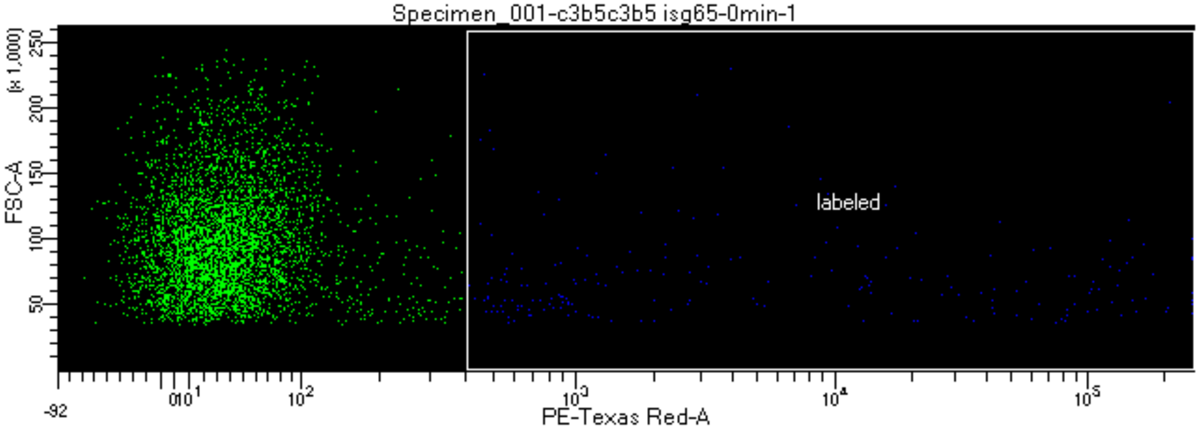

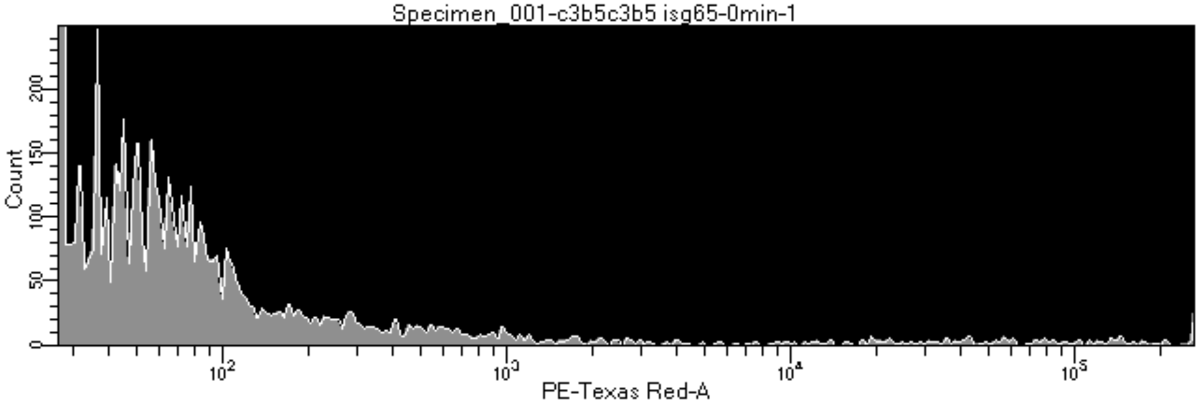

Tube: c3b5c3b5 isg65-0min-1

Population	#Events	%Parent	%Total
All Events	10,000	####	100.0
cells	4,444	44.4	44.4
singlets	4,282	96.4	42.8
labeled	197	4.6	2.0

BD FACSDiva 8.0.1

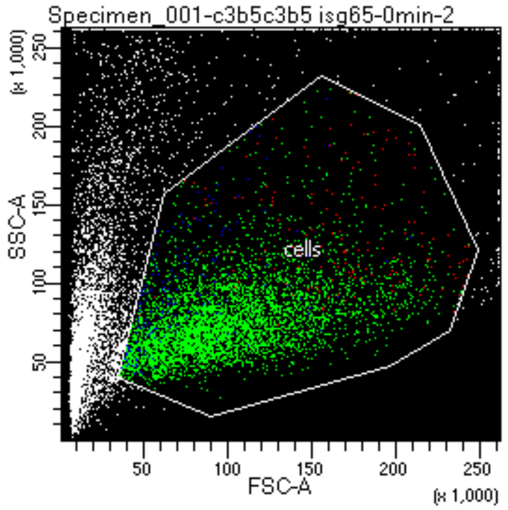

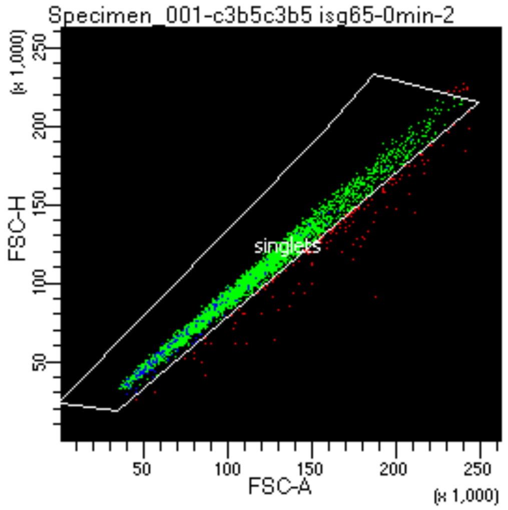

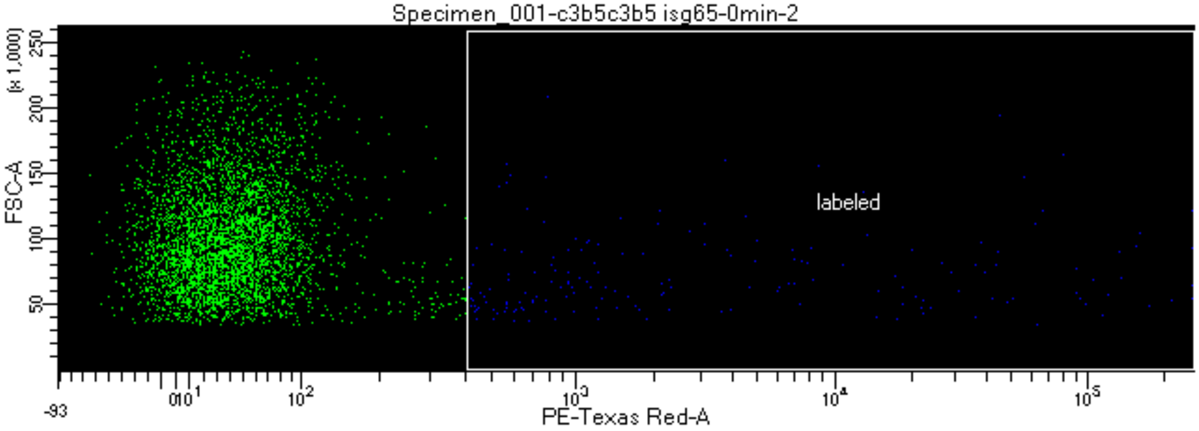

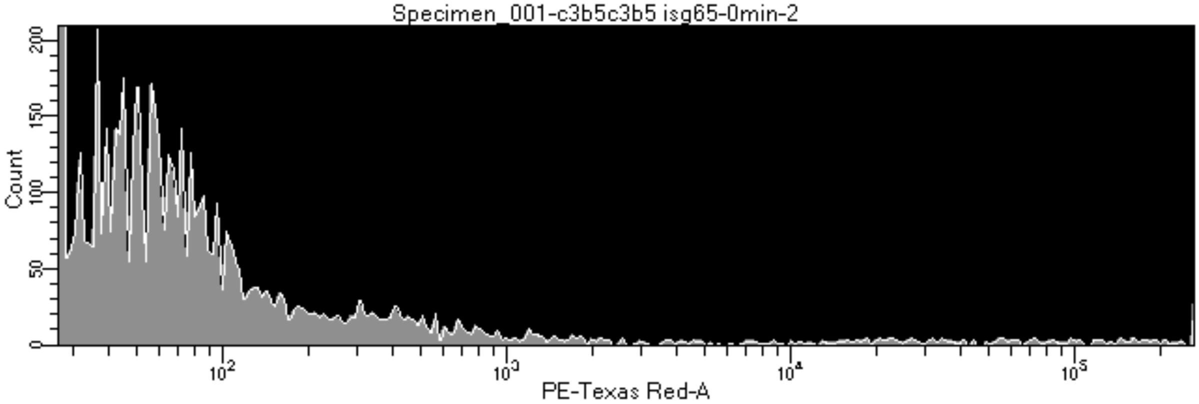

Tube: c3b5c3b5 isg65-0min-2

Population	#Events	%Parent	%Total
All Events	10,000	####	100.0
cells	4,436	44.4	44.4
singlets	4,275	96.4	42.8
labeled	171	4.0	1.7

BD FACSDiva 8.0.1

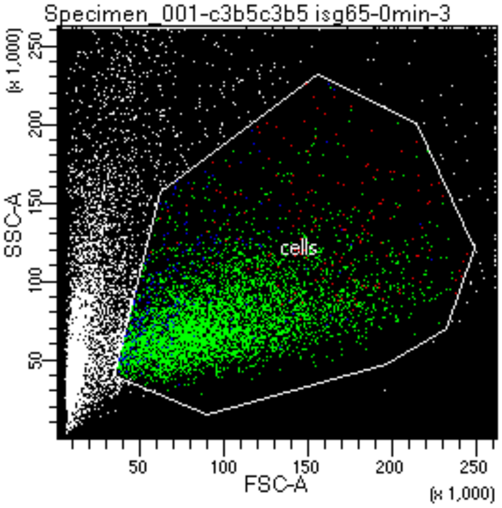

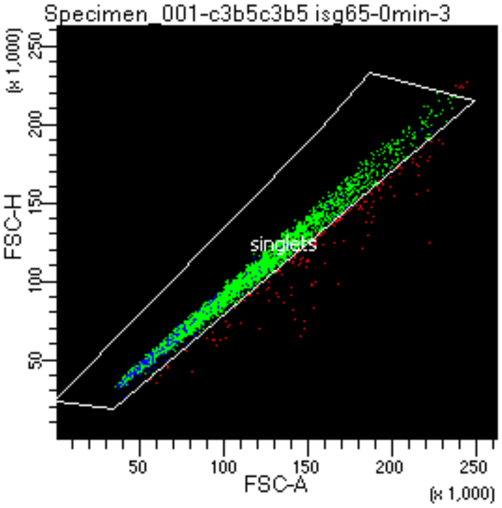

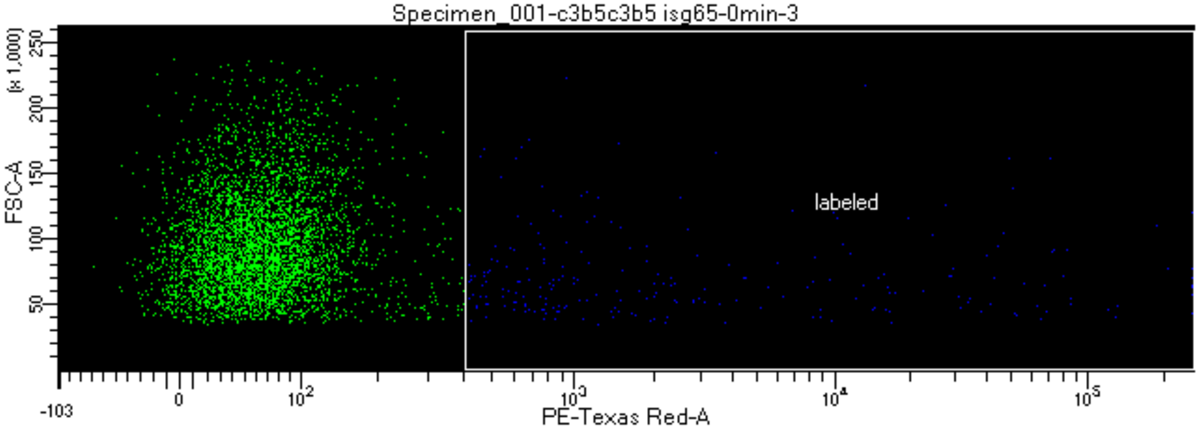

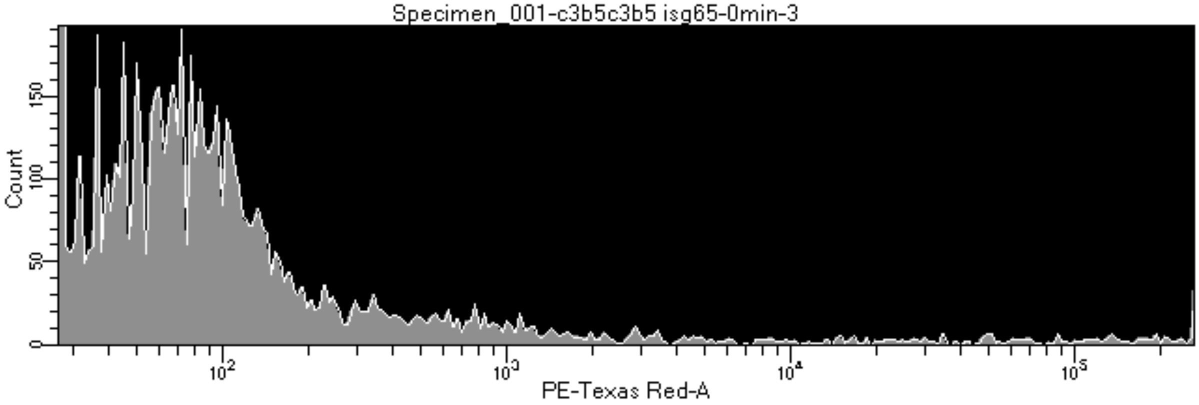

Tube: c3b5c3b5 isg65-0min-3

Population	#Events	%Parent	%Total
All Events	10,000	####	100.0
cells	4,578	45.8	45.8
singlets	4,411	96.4	44.1
labeled	222	5.0	2.2

BD FACSDiva 8.0.1

Specimen_001-c3b5c3b5 isg75-0min-1

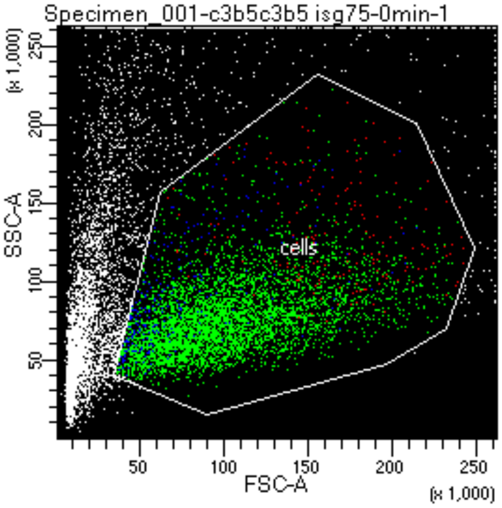

Specimen_001-c3b5c3b5 isg75-0min-1

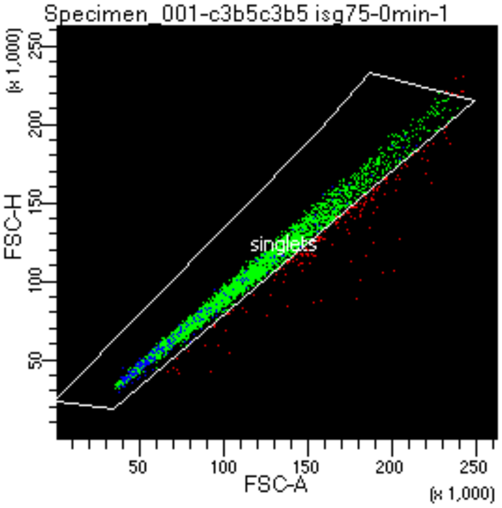

Specimen_001-c3b5c3b5 isg75-0min-1

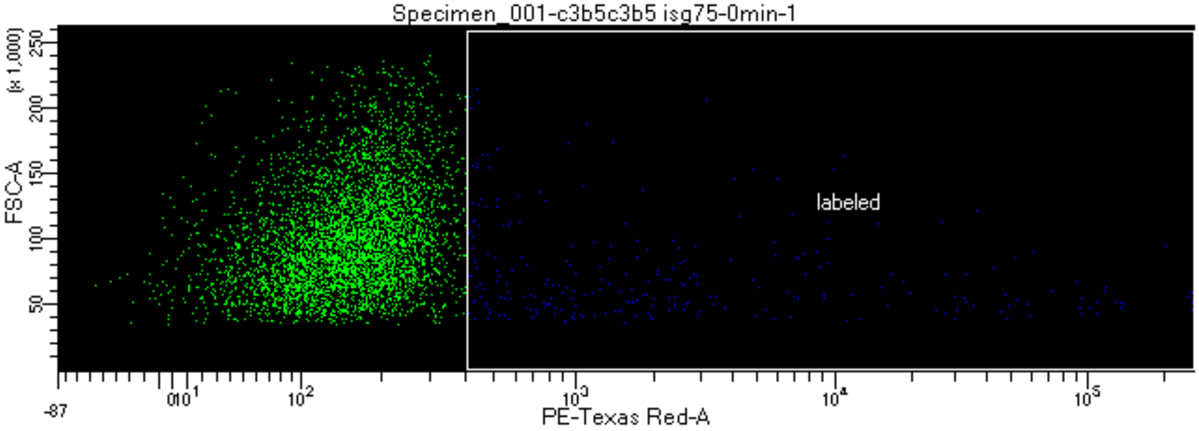

Specimen_001-c3b5c3b5 isg75-0min-1

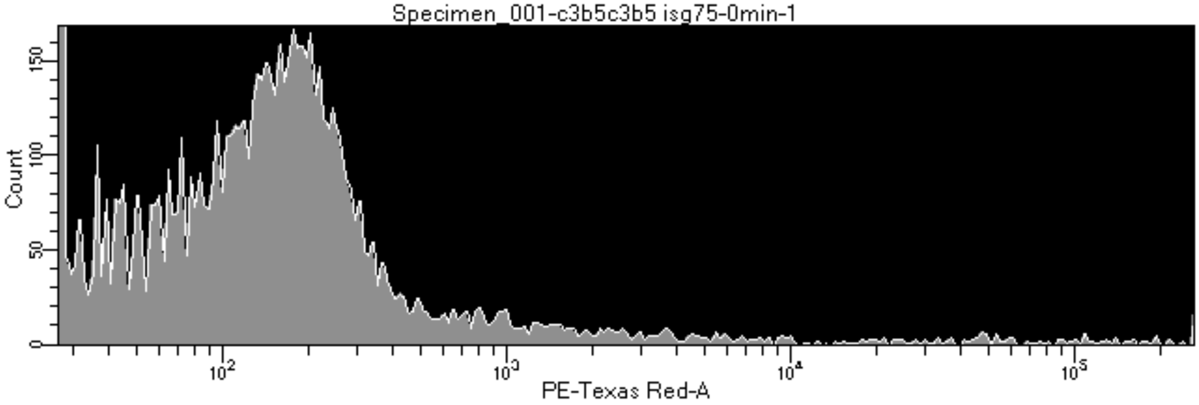

Tube: c3b5c3b5 isg75-0min-1

Population	#Events	%Parent	%Total
All Events	10,000	####	100.0
cells	4,748	47.5	47.5
singlets	4,568	96.2	45.7
labeled	255	5.6	2.6

BD FACSDiva 8.0.1

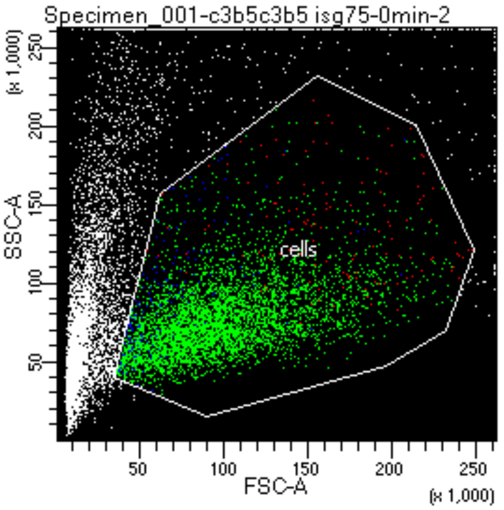

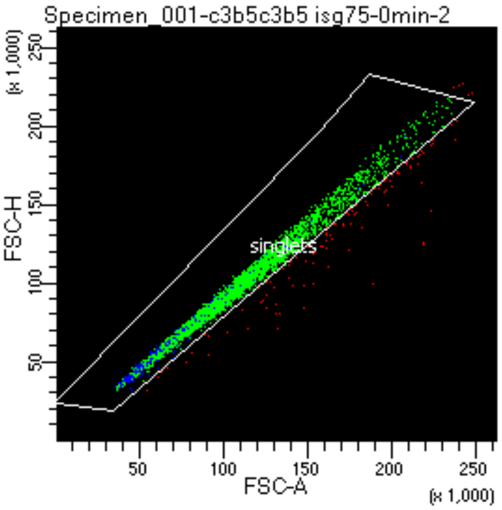

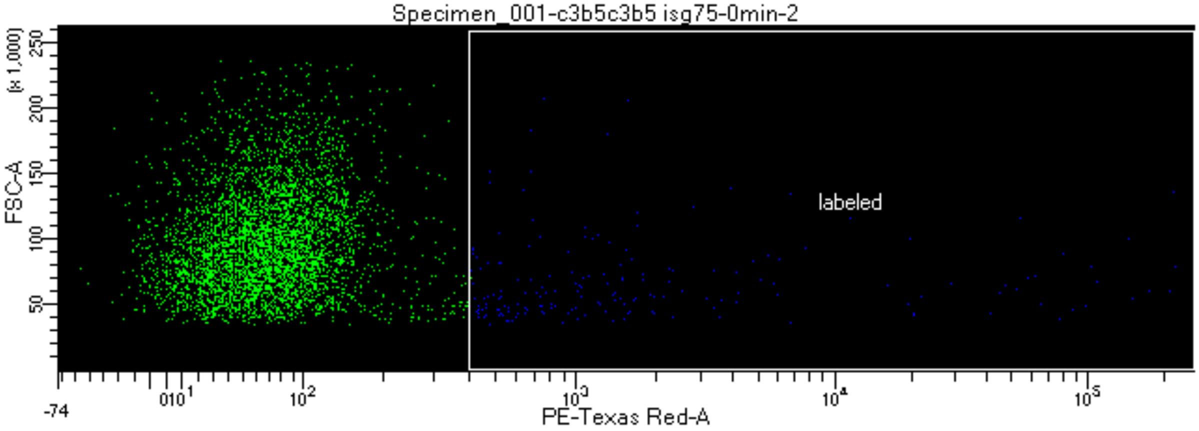

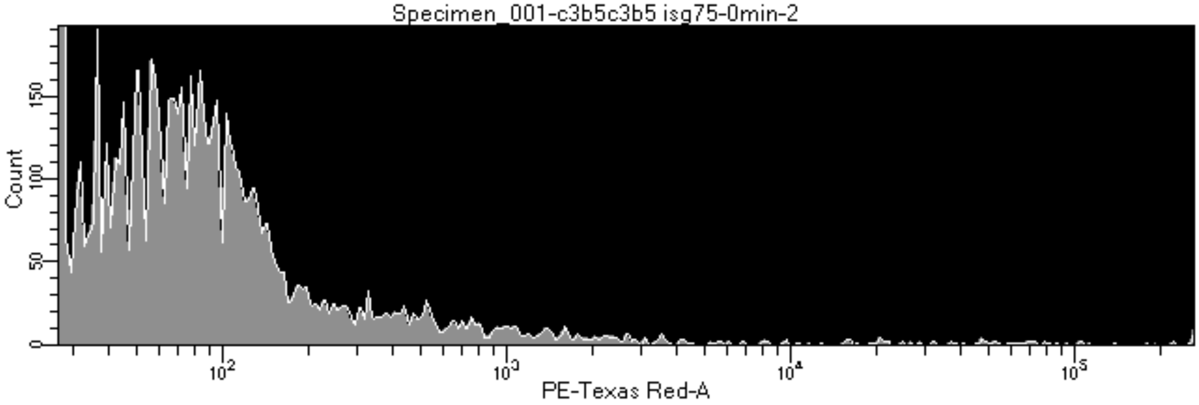

Tube: c3b5c3b5 isg75-0min-2

Population	#Events	%Parent	%Total
All Events	10,000	####	100.0
cells	4,489	44.9	44.9
singlets	4,358	97.1	43.6
labeled	175	4.0	1.8

BD FACSDiva 8.0.1

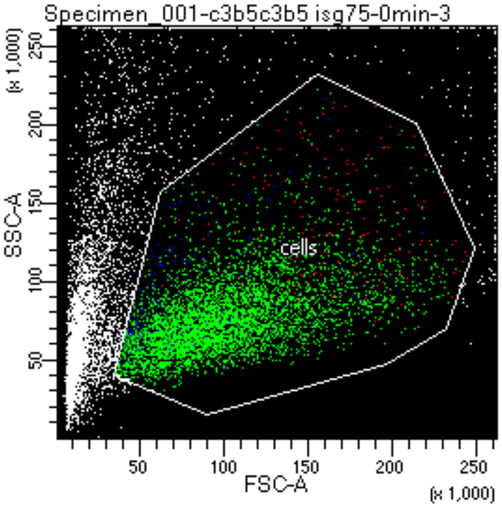

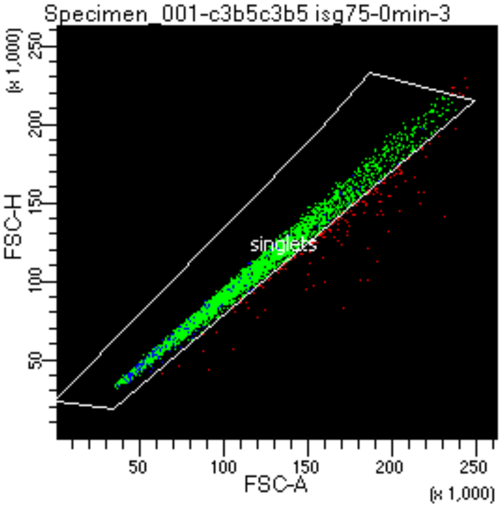

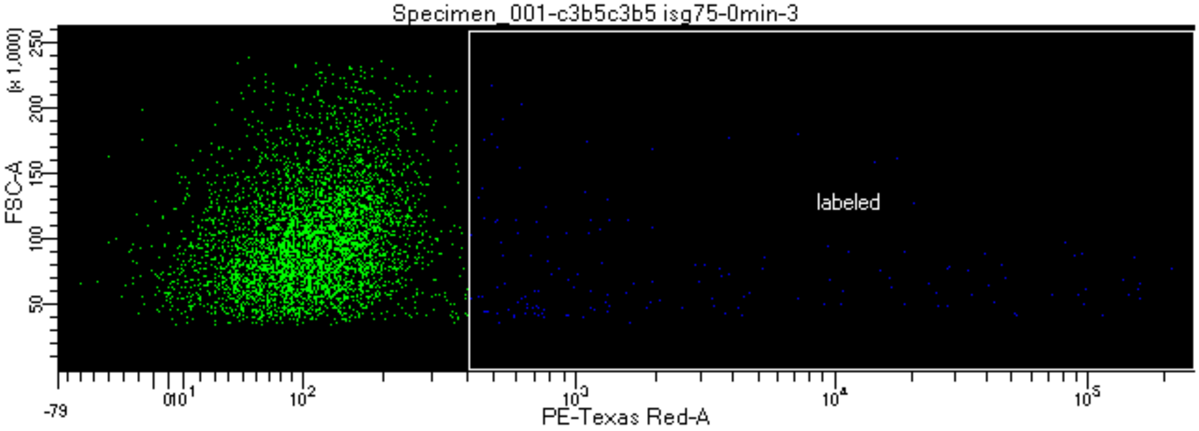

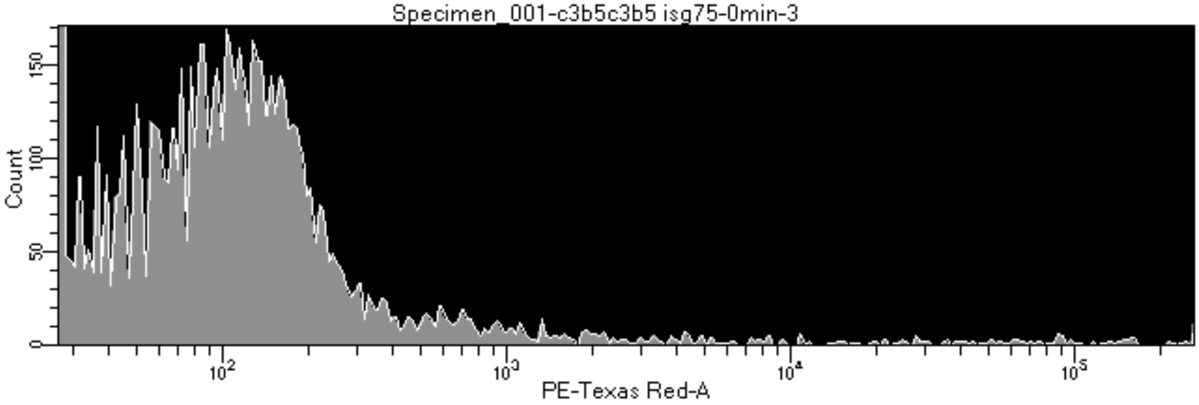

Tube: c3b5c3b5 isg75-0min-3

Population	#Events	%Parent	%Total
All Events	10,000	####	100.0
cells	4,806	48.1	48.1
singlets	4,639	96.5	46.4
labeled	148	3.2	1.5

BD FACSDiva 8.0.1

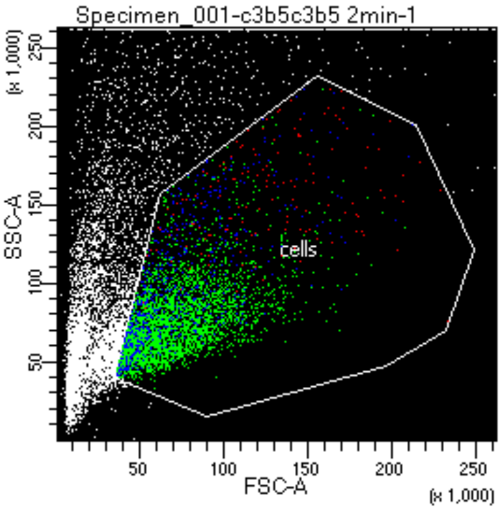

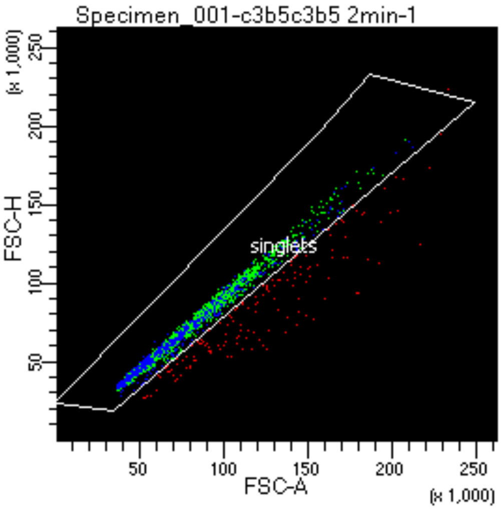

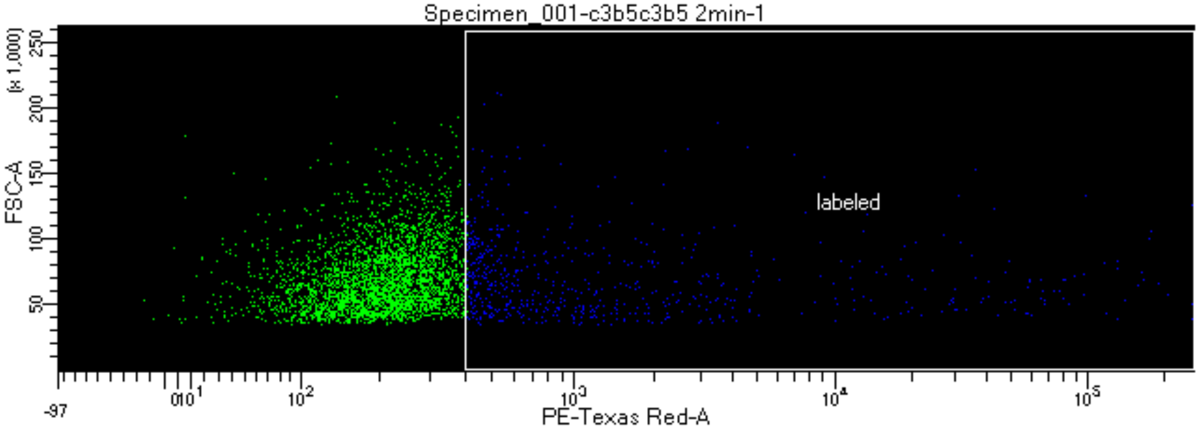

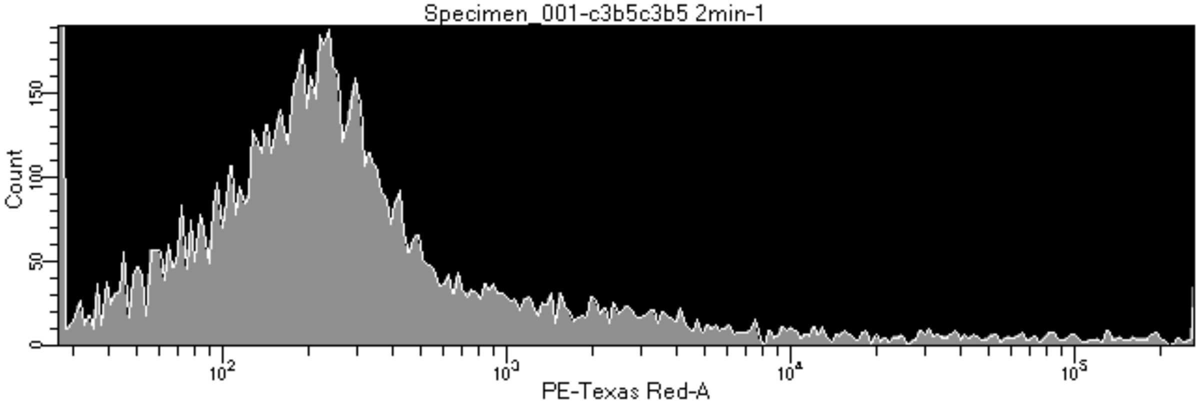

Tube: c3b5c3b5 2min-1

Population	#Events	%Parent	%Total
All Events	10,000	####	100.0
cells	3,904	39.0	39.0
singlets	3,754	96.2	37.5
labeled	618	16.5	6.2

BD FACSDiva 8.0.1

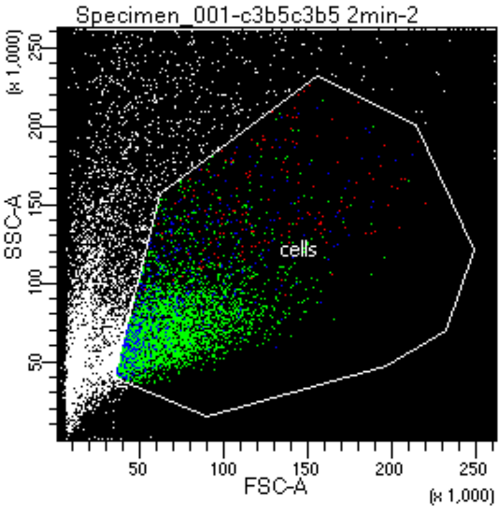

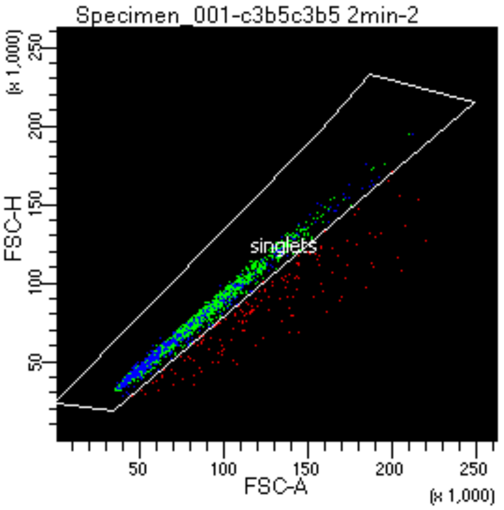

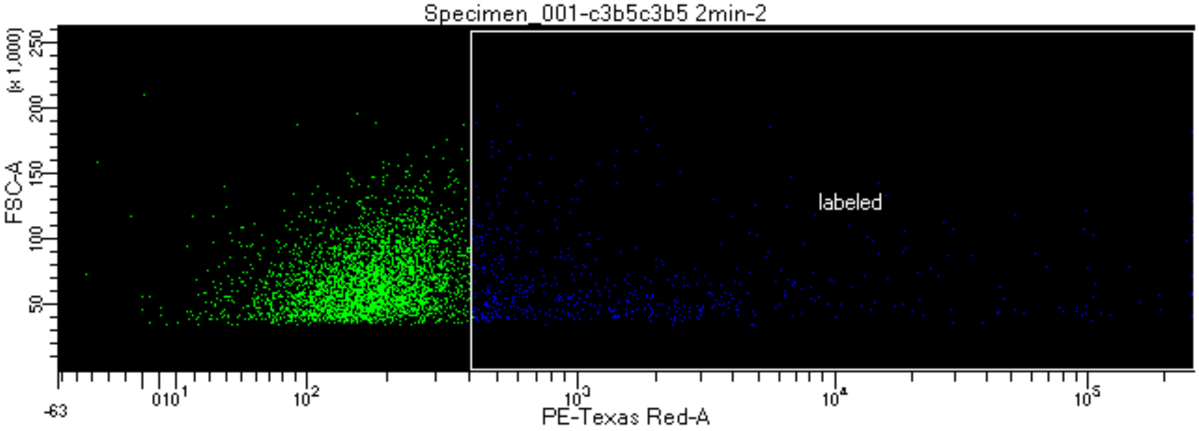

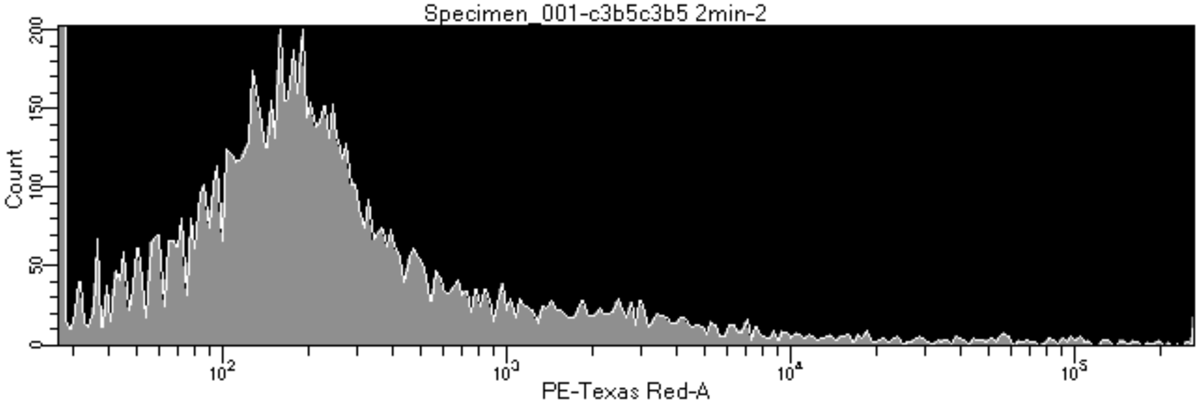

Tube: c3b5c3b5 2min-2

Population	#Events	%Parent	%Total
All Events	10,000	####	100.0
cells	3,884	38.8	38.8
singlets	3,726	95.9	37.3
labeled	521	14.0	5.2

BD FACSDiva 8.0.1

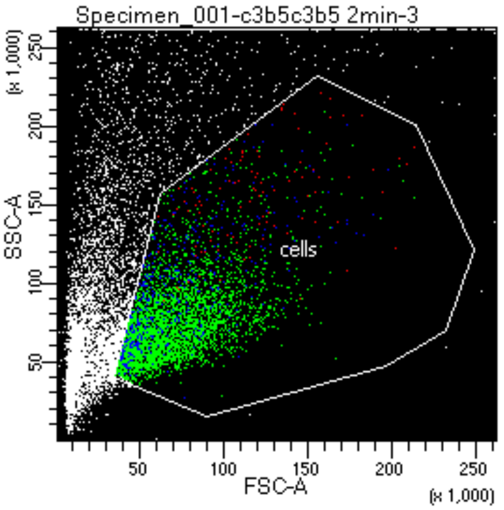

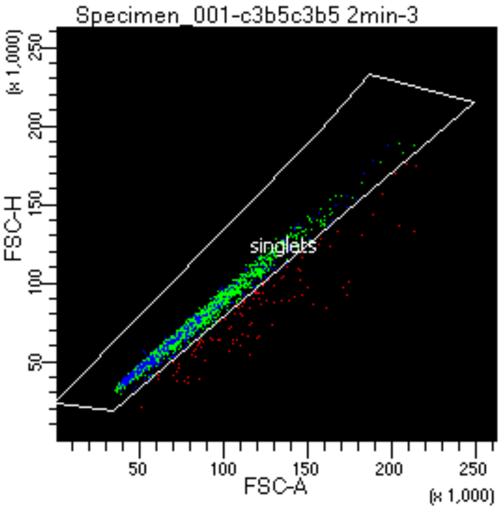

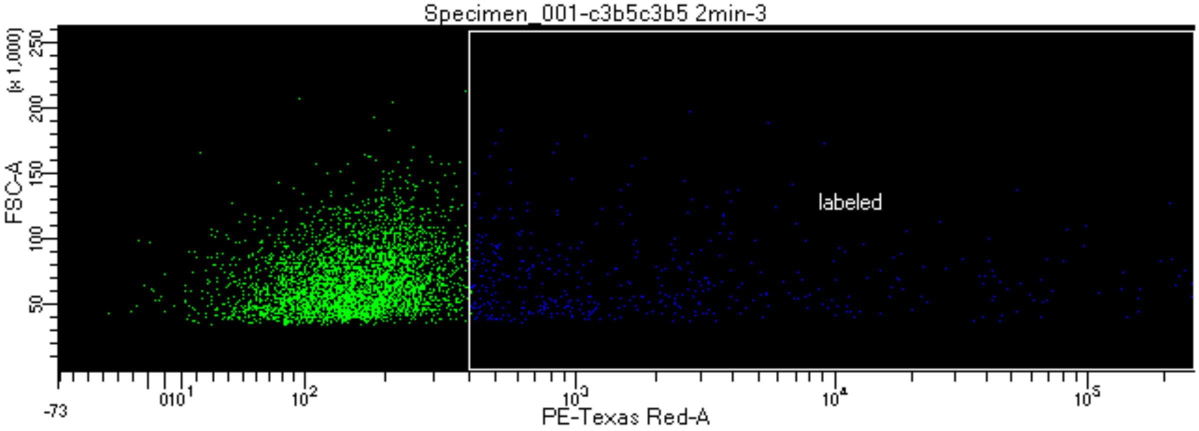

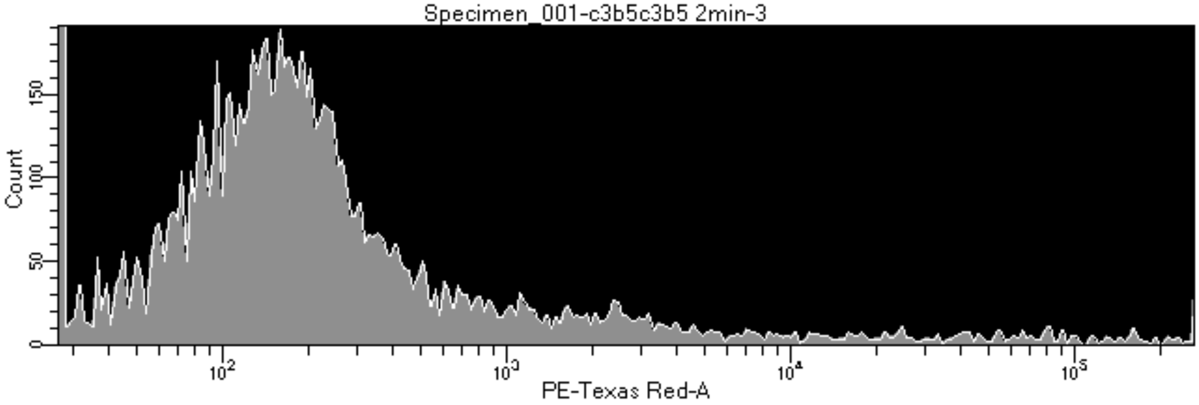

Tube: c3b5c3b5 2min-3

Population	#Events	%Parent	%Total
All Events	10,000	####	100.0
cells	4,112	41.1	41.1
singlets	4,003	97.3	40.0
labeled	431	10.8	4.3

BD FACSDiva 8.0.1

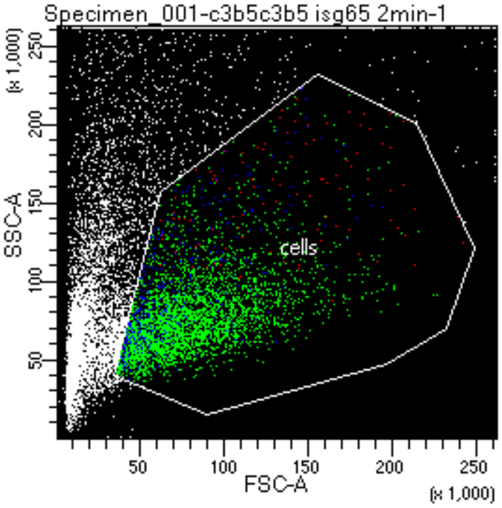

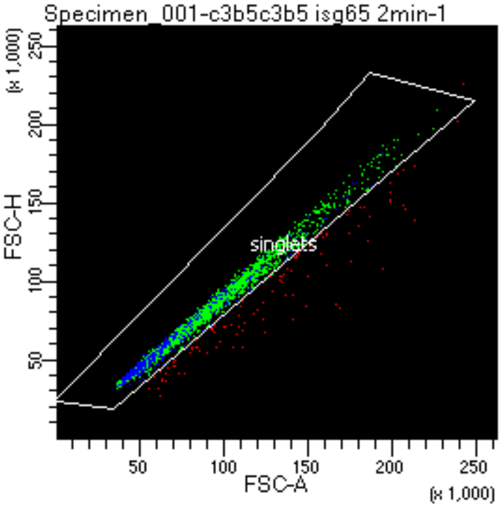

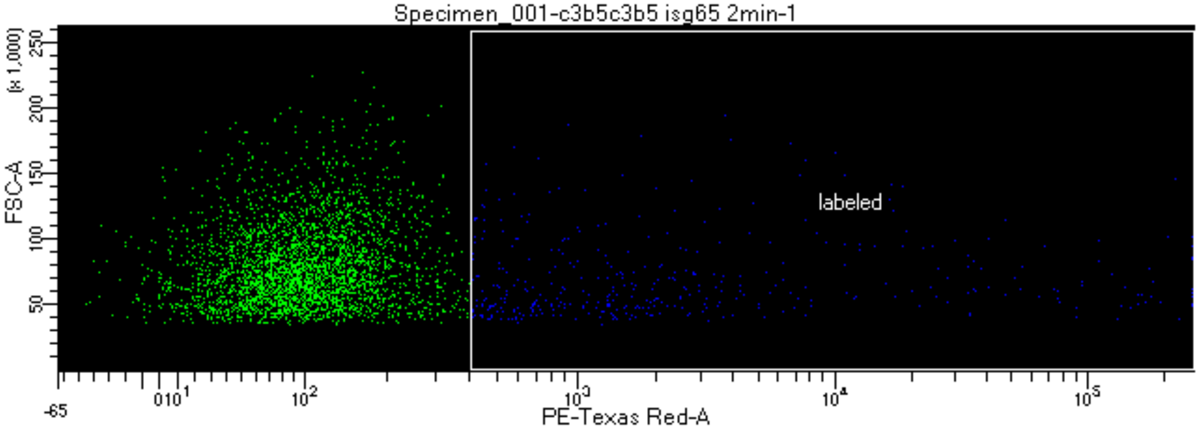

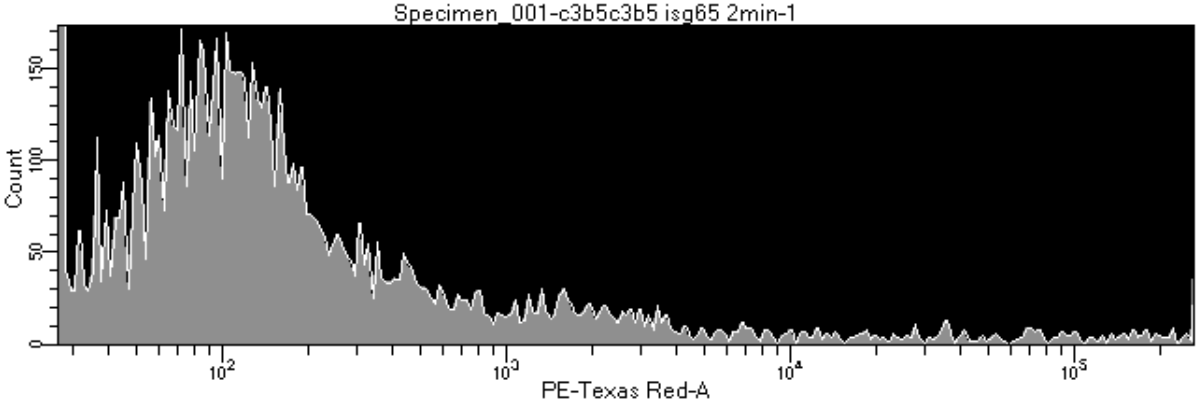

Tube: c3b5c3b5 isg65 2min-1

Population	#Events	%Parent	%Total
All Events	10,000	####	100.0
cells	3,656	36.6	36.6
singlets	3,547	97.0	35.5
labeled	377	10.6	3.8

BD FACSDiva 8.0.1

Specimen_001-c3b5c3b5 isg65 2min-2

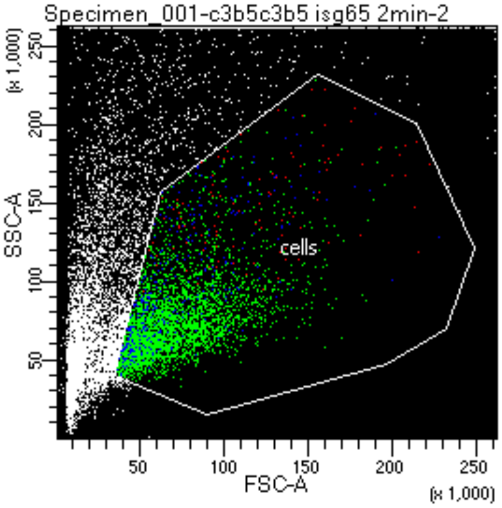

Specimen_001-c3b5c3b5 isg65 2min-2

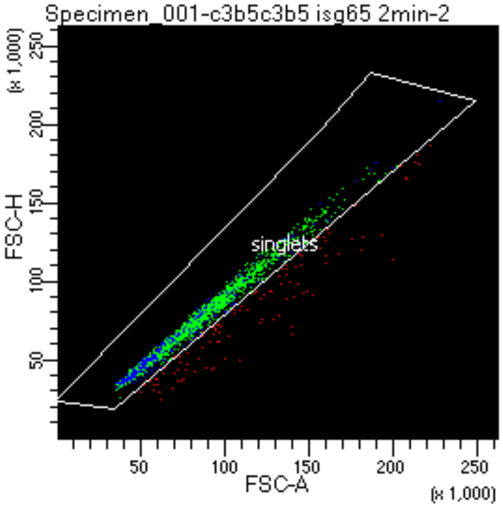

Specimen_001-c3b5c3b5 isg65 2min-2

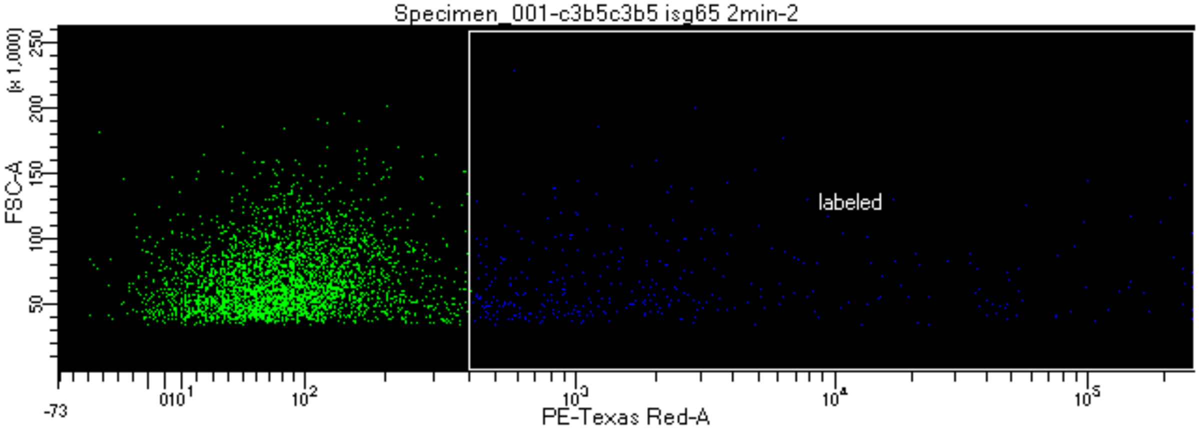

Specimen_001-c3b5c3b5 isg65 2min-2

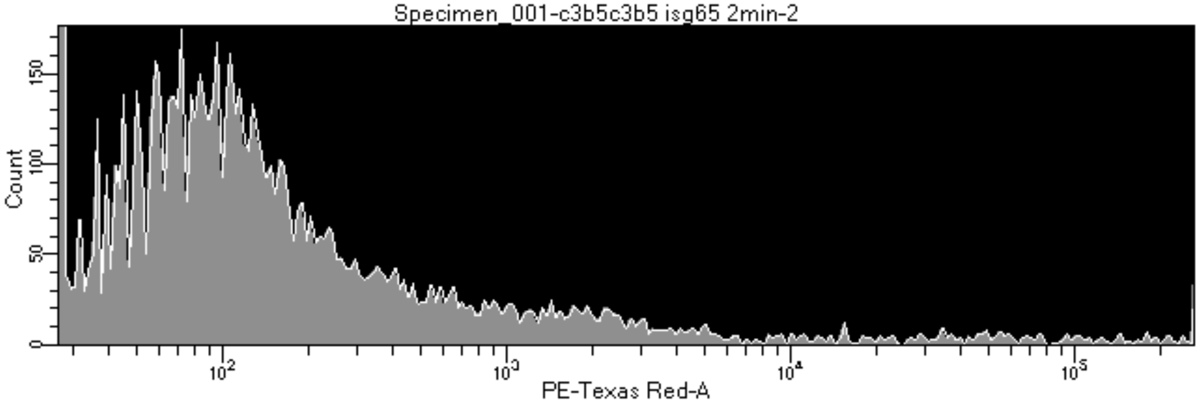

Tube: c3b5c3b5 isg65 2min-2

Population	#Events	%Parent	%Total
All Events	10,000	####	100.0
cells	3,735	37.4	37.4
singlets	3,621	96.9	36.2
labeled	347	9.6	3.5

BD FACSDiva 8.0.1

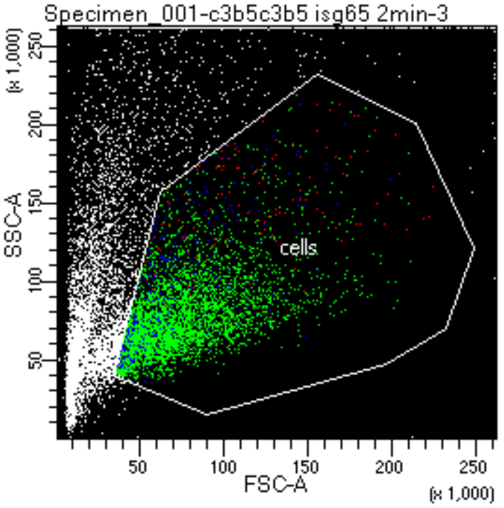

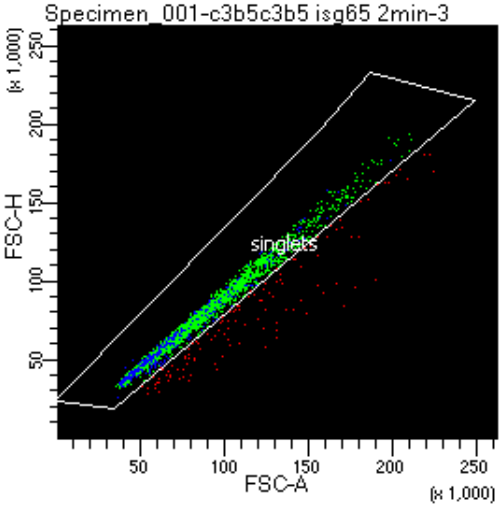

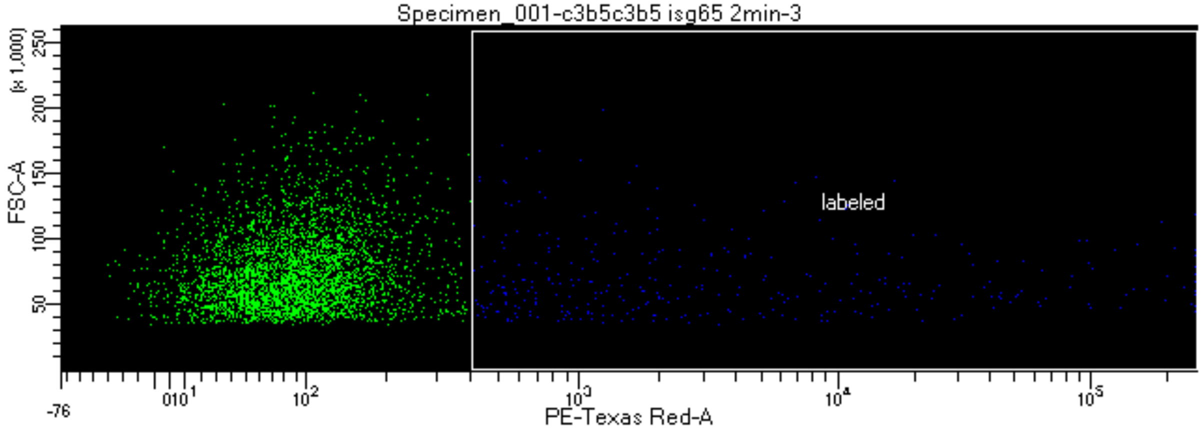

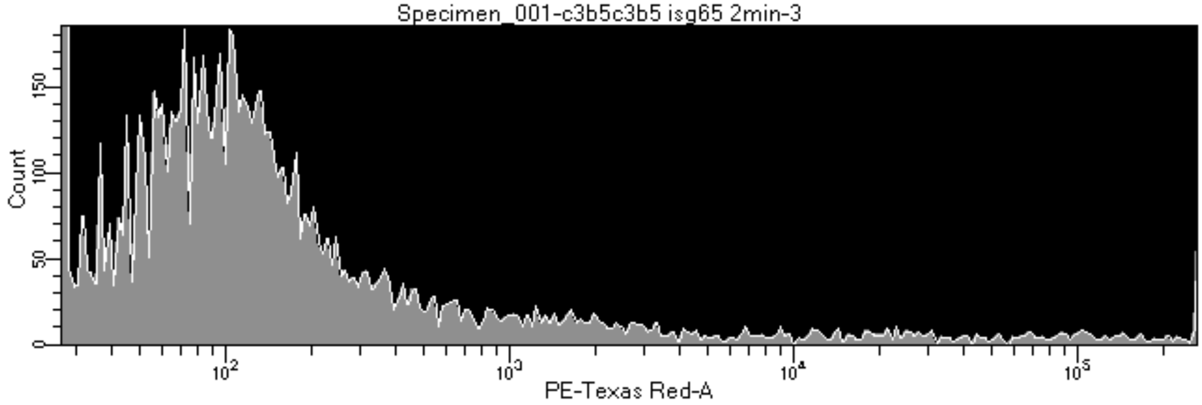

Tube: c3b5c3b5 isg65 2min-3

Population	#Events	%Parent	%Total
All Events	10,000	####	100.0
cells	4,095	40.9	40.9
singlets	3,968	96.9	39.7
labeled	323	8.1	3.2

BD FACSDiva 8.0.1

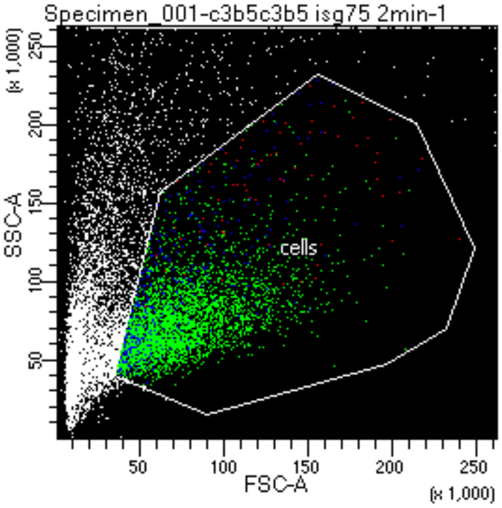

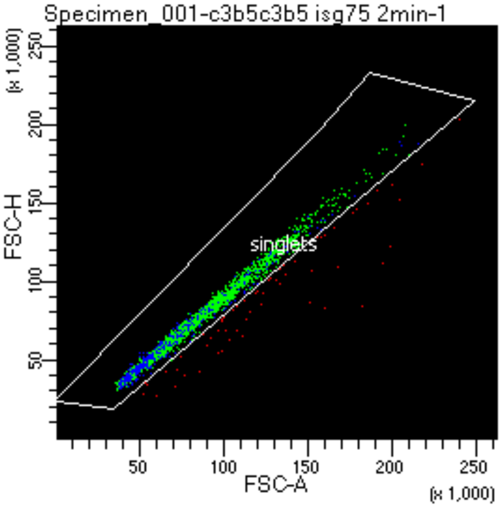

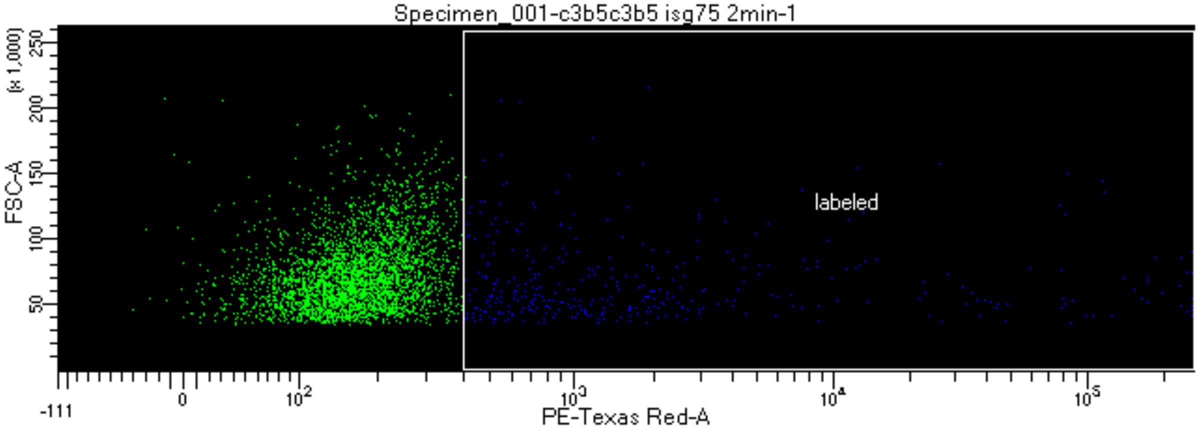

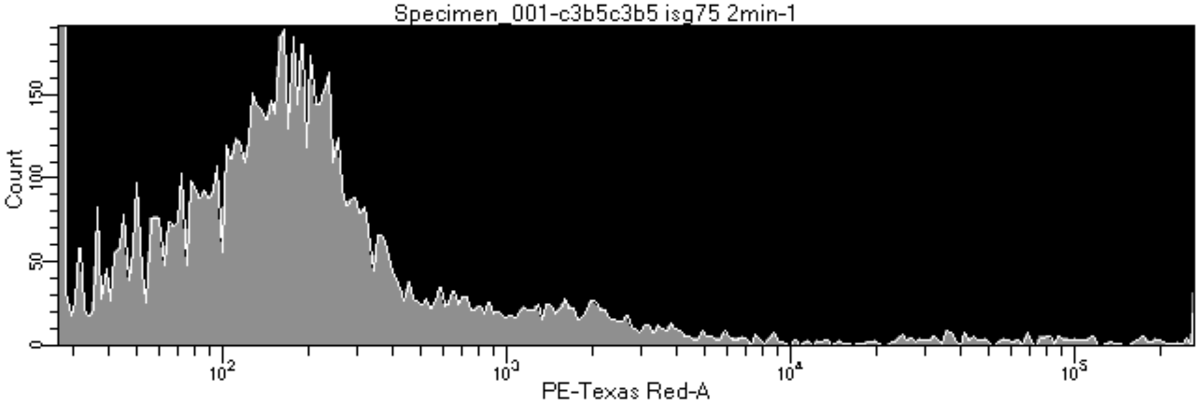

Tube: c3b5c3b5 isg75 2min-1

Population	#Events	%Parent	%Total
All Events	10,000	####	100.0
cells	3,847	38.5	38.5
singlets	3,777	98.2	37.8
labeled	370	9.8	3.7

BD FACSDiva 8.0.1

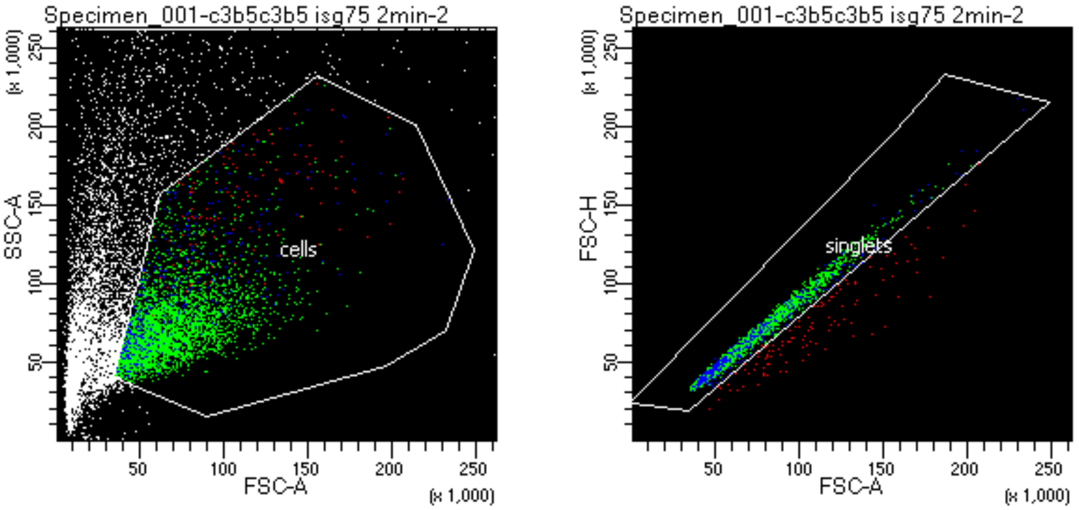

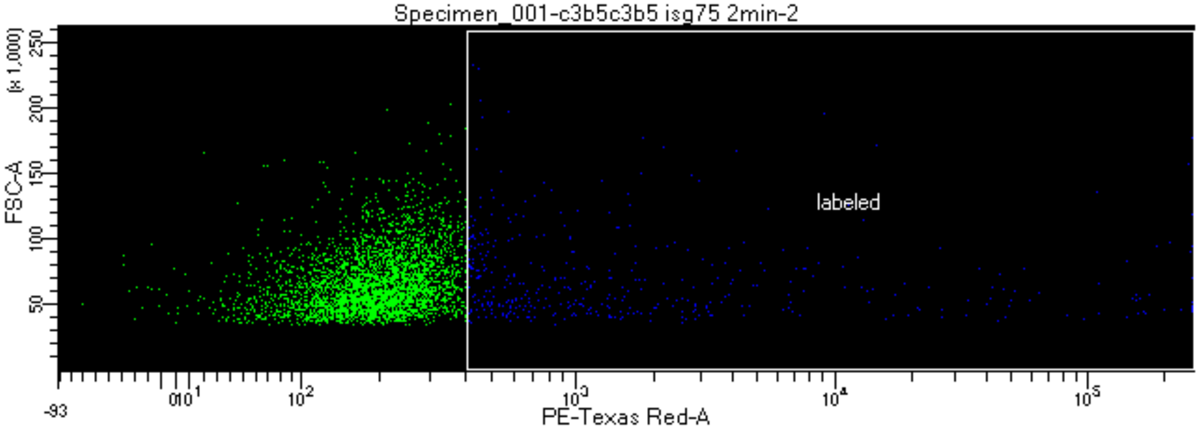

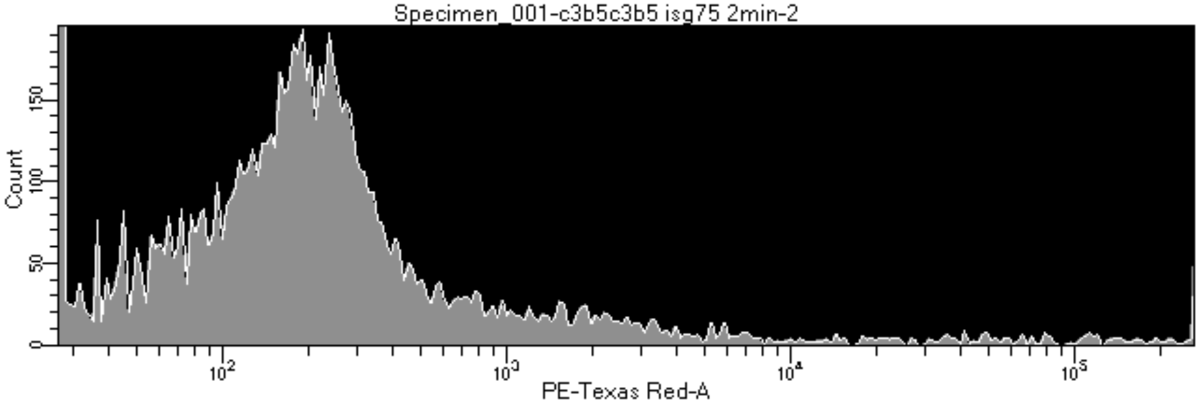

Tube: c3b5c3b5 isg75 2min-2

Population	#Events	%Parent	%Total
All Events	10,000	####	100.0
cells	3,927	39.3	39.3
singlets	3,801	96.8	38.0
labeled	398	10.5	4.0

BD FACSDiva 8.0.1

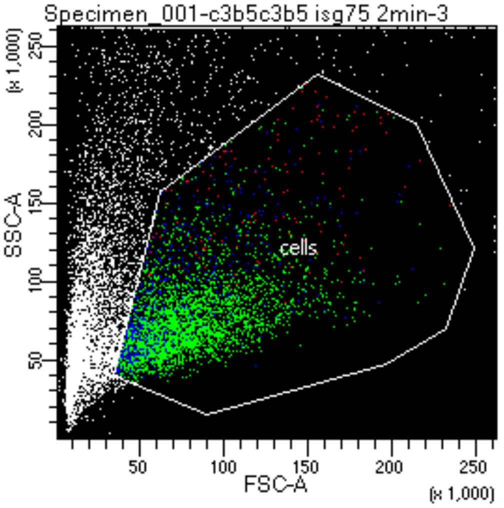

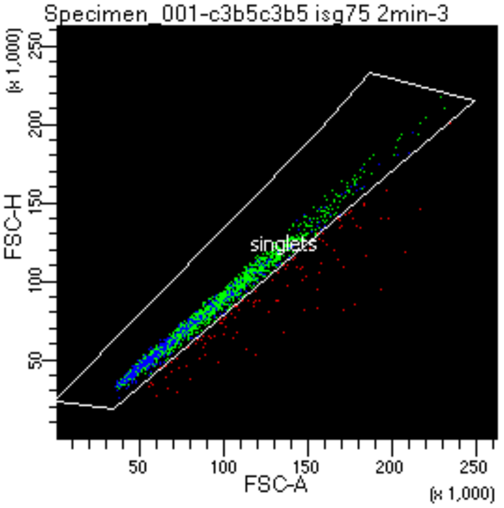

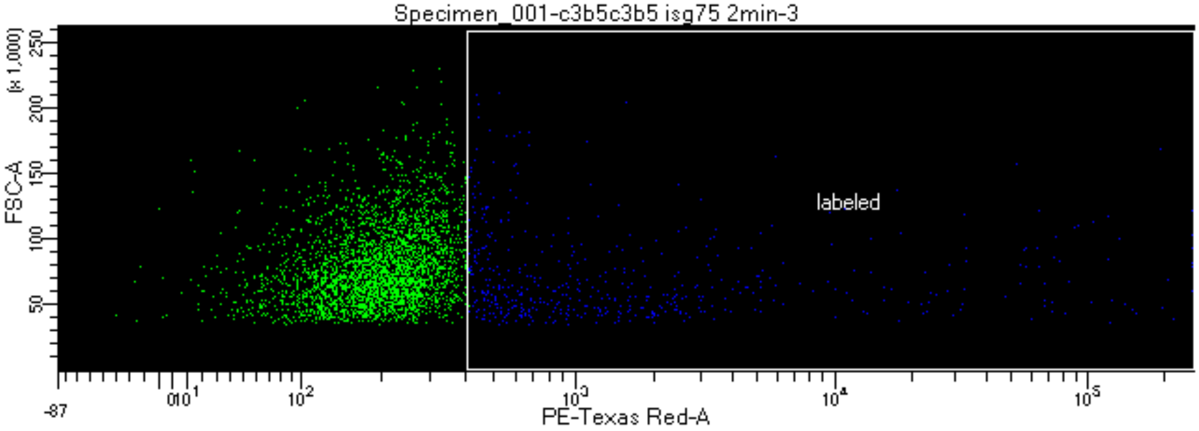

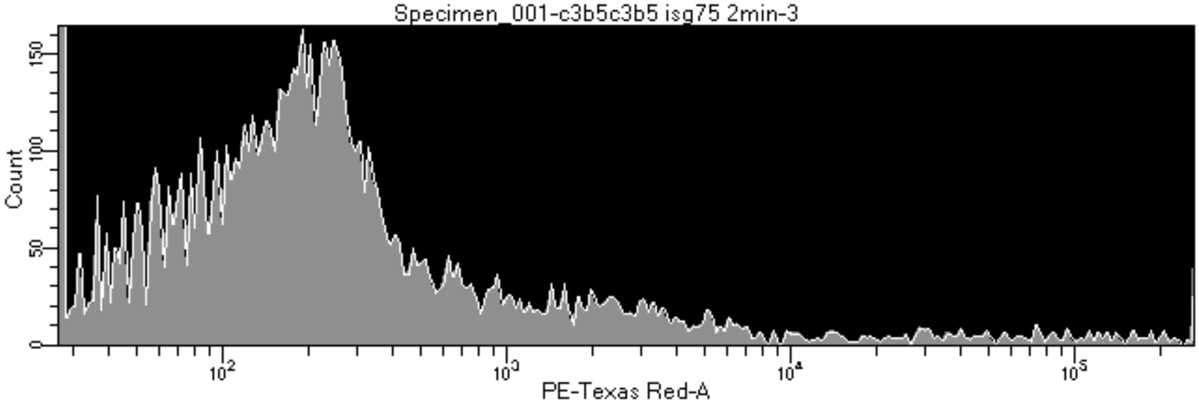

Tube: c3b5c3b5 isg75 2min-3

Population	#Events	%Parent	%Total
All Events	10,000	####	100.0
cells	3,586	35.9	35.9
singlets	3,466	96.7	34.7
labeled	481	13.9	4.8

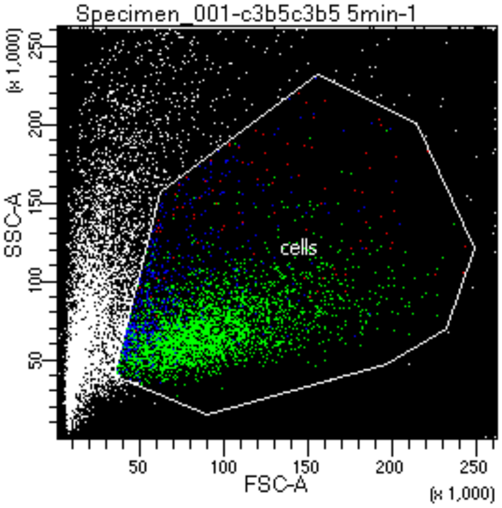

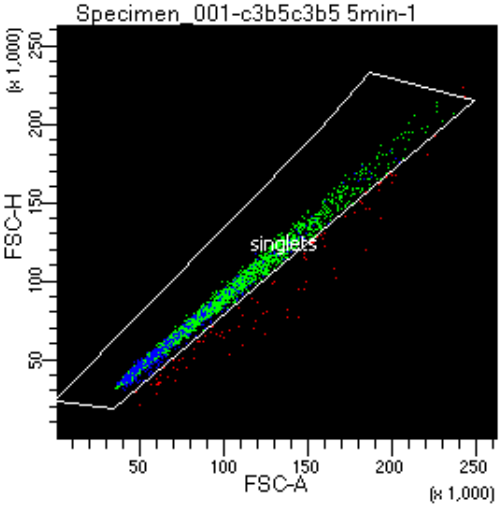

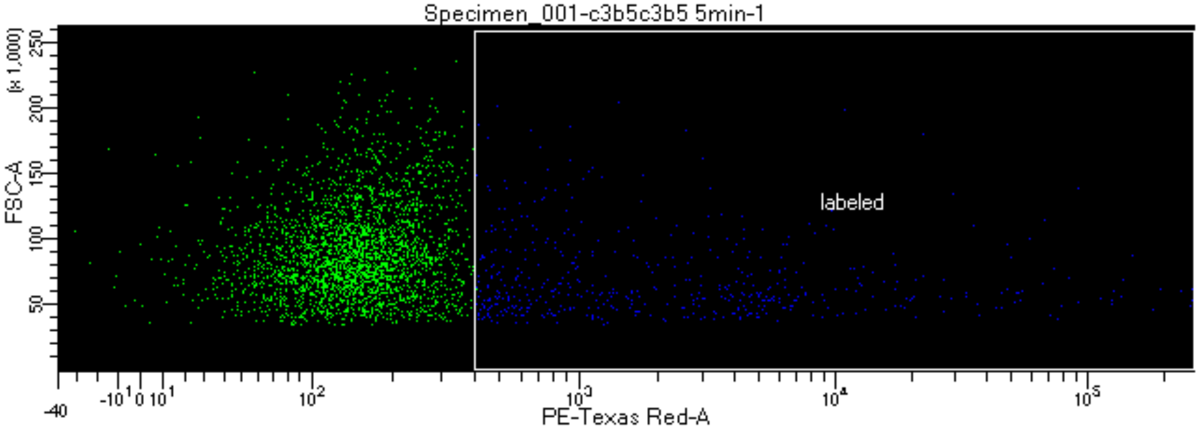

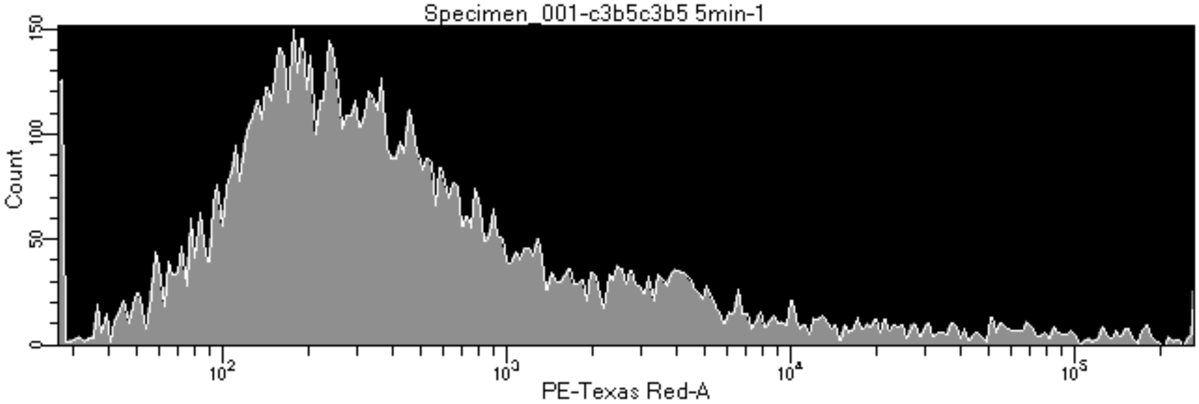

Tube: c3b5c3b5 5min-1

Population	#Events	%Parent	%Total
All Events	10,000	####	100.0
cells	3,242	32.4	32.4
singlets	3,153	97.3	31.5
labeled	495	15.7	5.0

BD FACSDiva 8.0.1

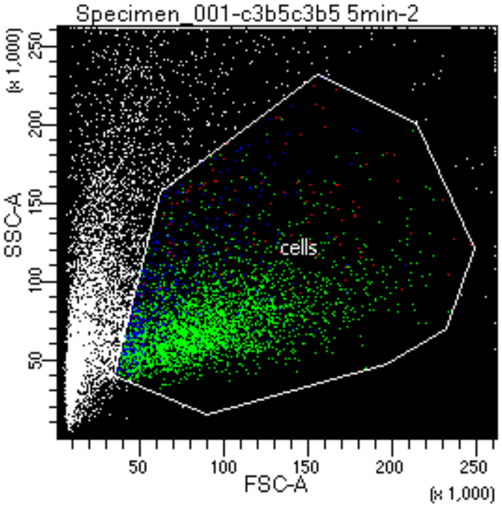

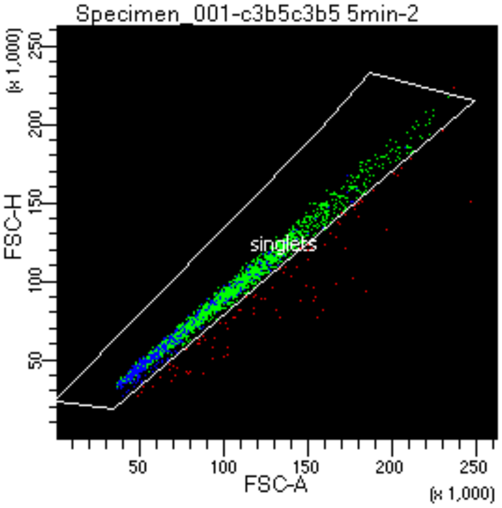

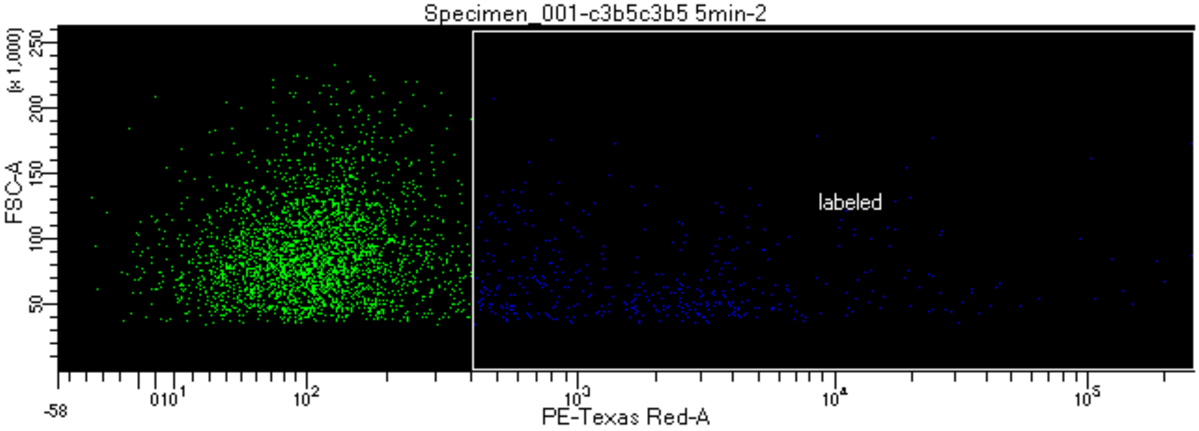

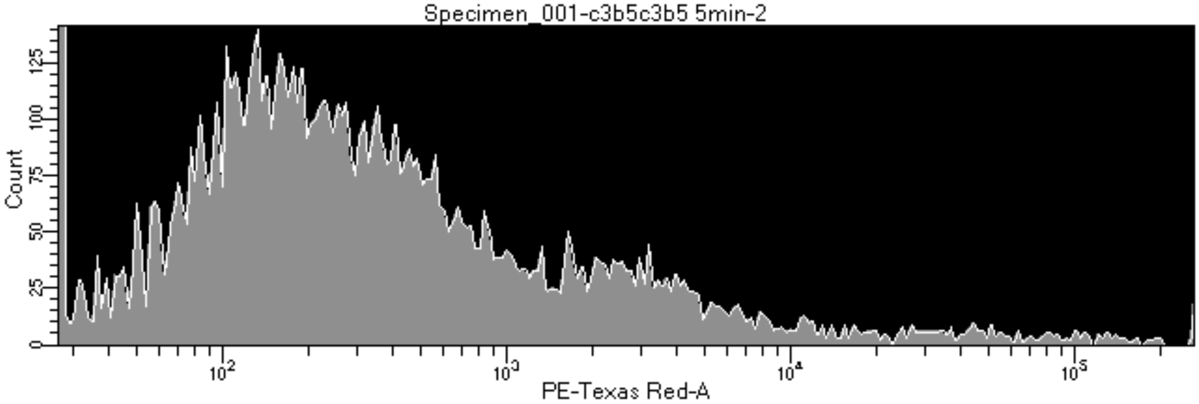

Tube: c3b5c3b5 5min-2

Population	#Events	%Parent	%Total
All Events	10,000	####	100.0
cells	3,388	33.9	33.9
singlets	3,287	97.0	32.9
labeled	419	12.7	4.2

BD FACSDiva 8.0.1

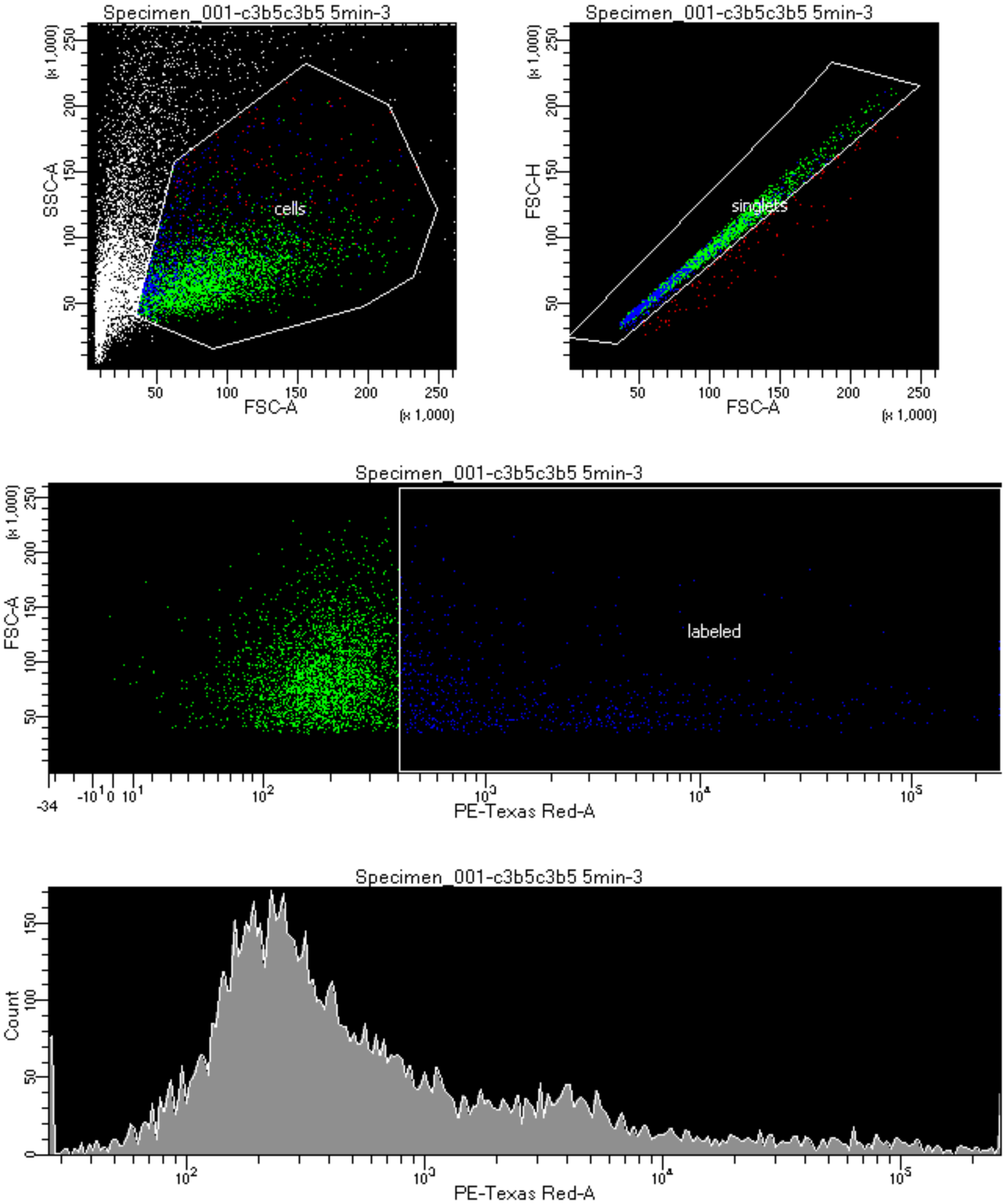

Tube: c3b5c3b5 5min-3

Population	#Events	%Parent	%Total
All Events	10,000	####	100.0
cells	3,592	35.9	35.9
singlets	3,476	96.8	34.8
labeled	597	17.2	6.0

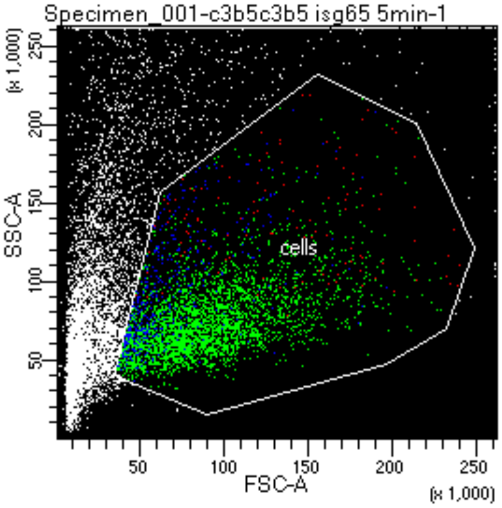

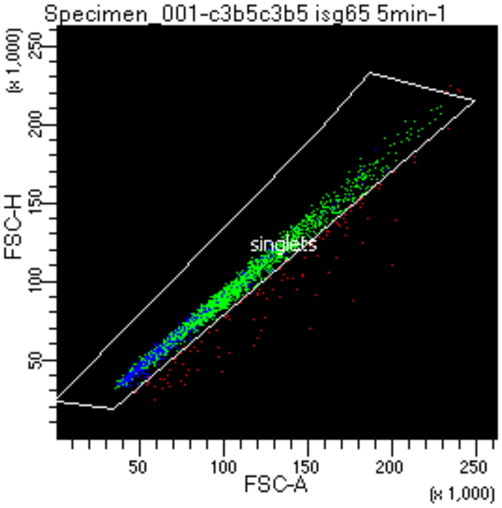

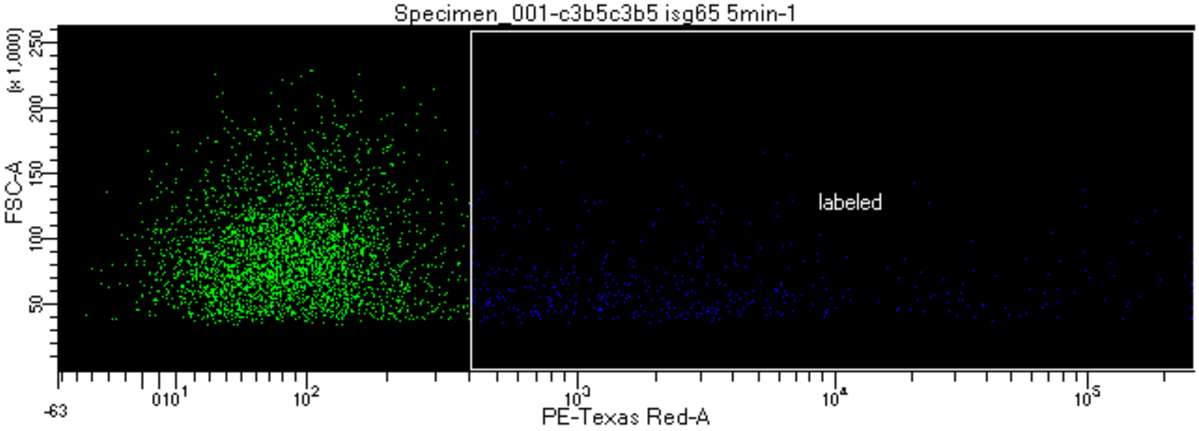

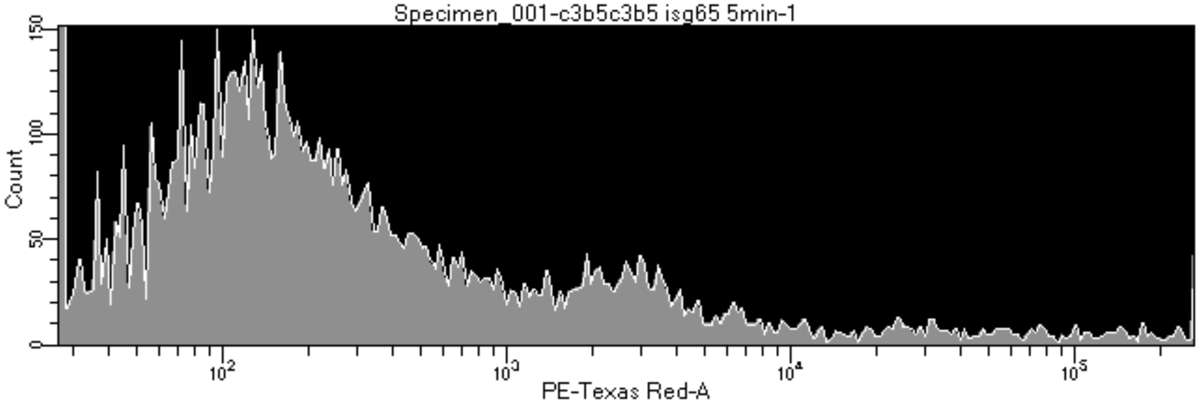

Tube: c3b5c3b5 isg65 5min-1

Population	#Events	%Parent	%Total
All Events	10,000	####	100.0
cells	3,592	35.9	35.9
singlets	3,476	96.8	34.8
labeled	428	12.3	4.3

BD FACSDiva 8.0.1

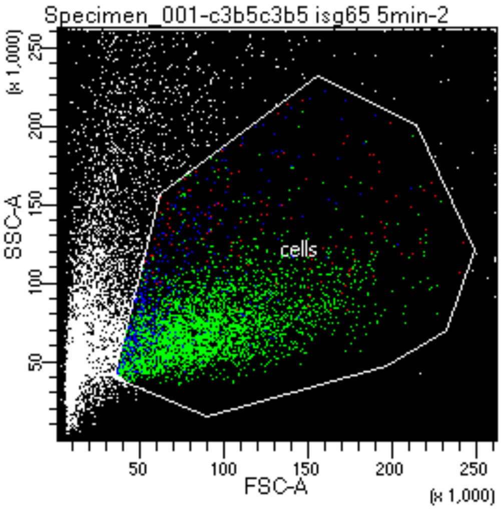

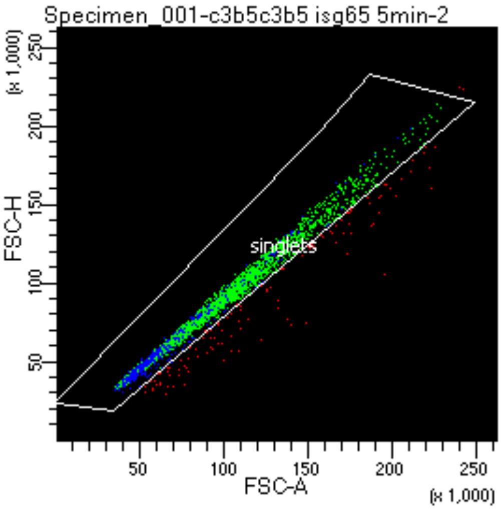

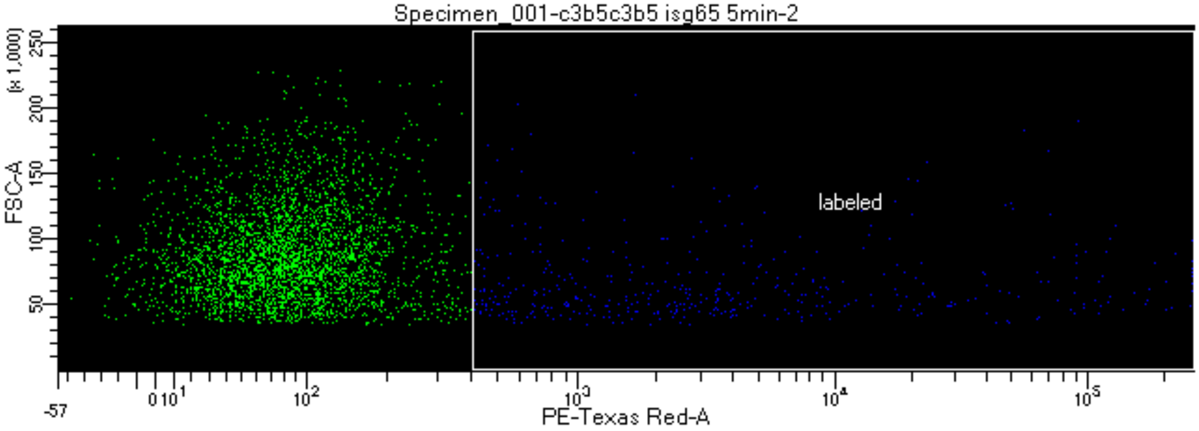

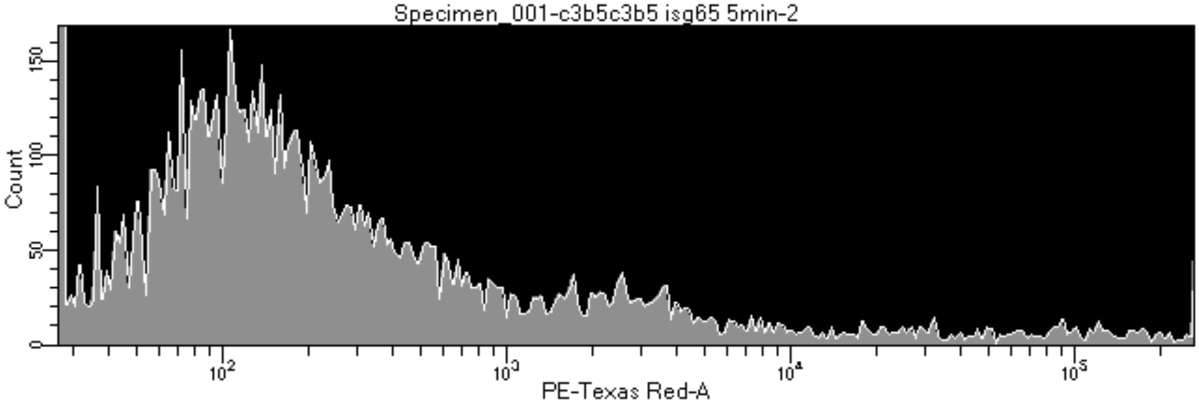

Tube: c3b5c3b5 isg65 5min-2

Population	#Events	%Parent	%Total
All Events	10,000	####	100.0
cells	3,804	38.0	38.0
singlets	3,680	96.7	36.8
labeled	418	11.4	4.2

Printed on: Tue Aug 30, 2022 11:01:05 CEST

BD FACSDiva 8.0.1

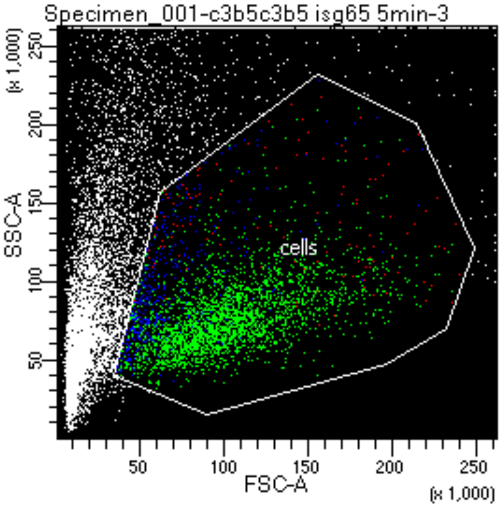

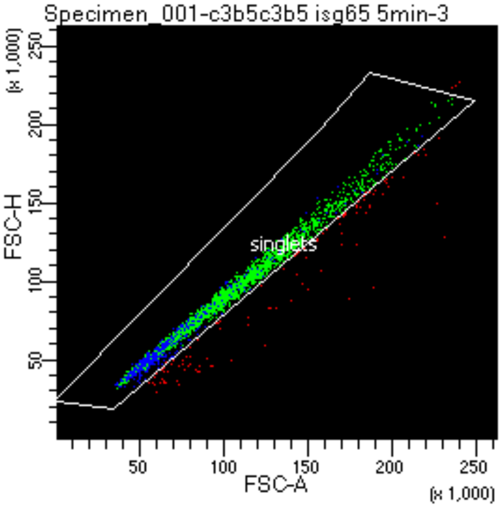

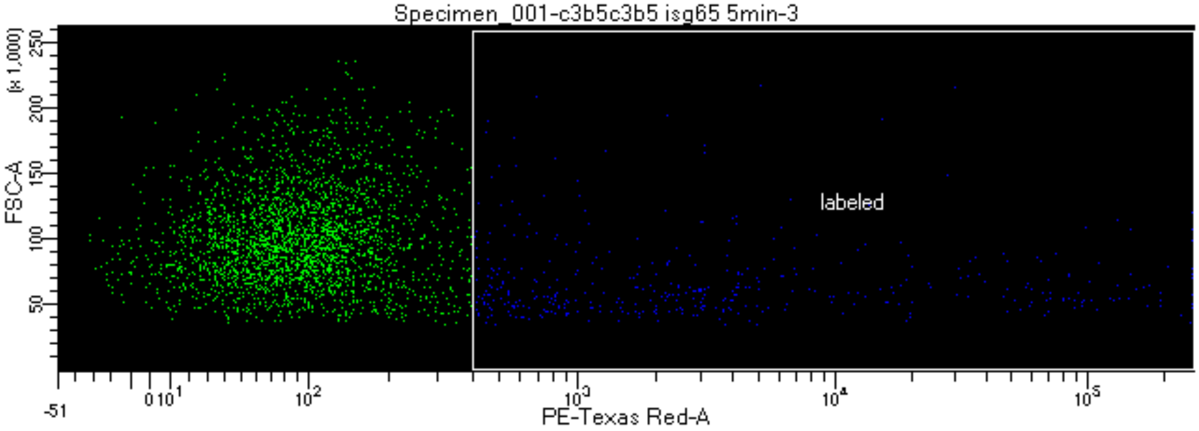

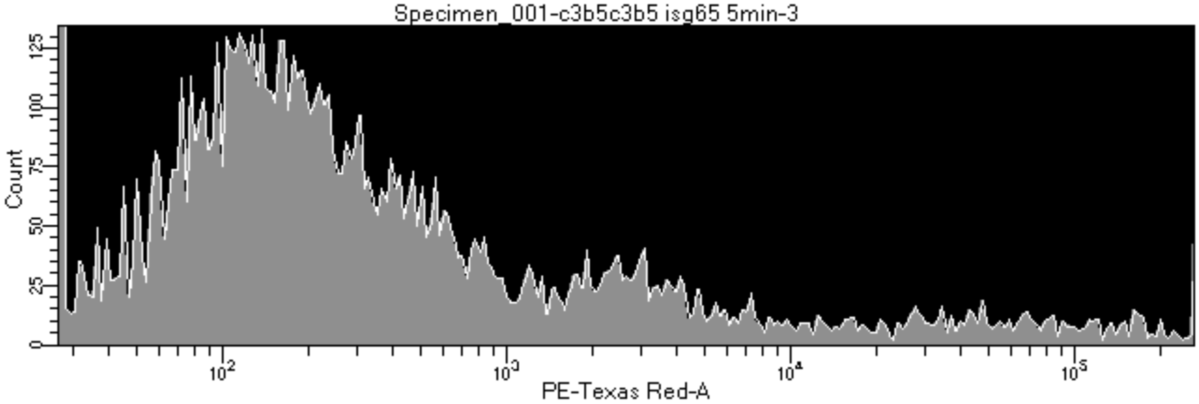

Tube: c3b5c3b5 isg65 5min-3

Population	#Events	%Parent	%Total
All Events	10,000	####	100.0
cells	3,011	30.1	30.1
singlets	2,887	95.9	28.9
labeled	397	13.8	4.0

BD FACSDiva 8.0.1

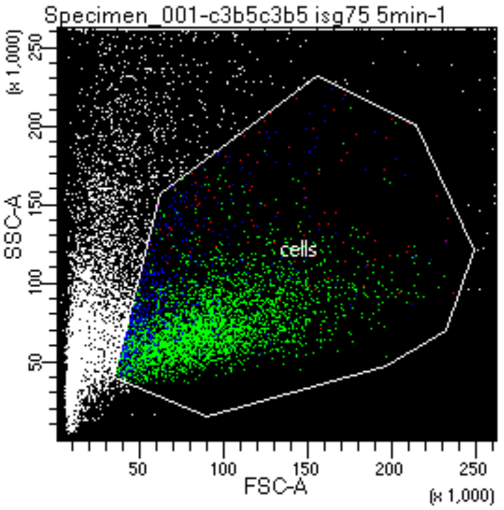

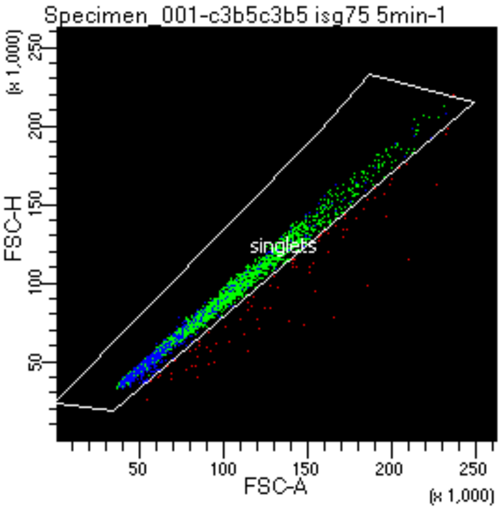

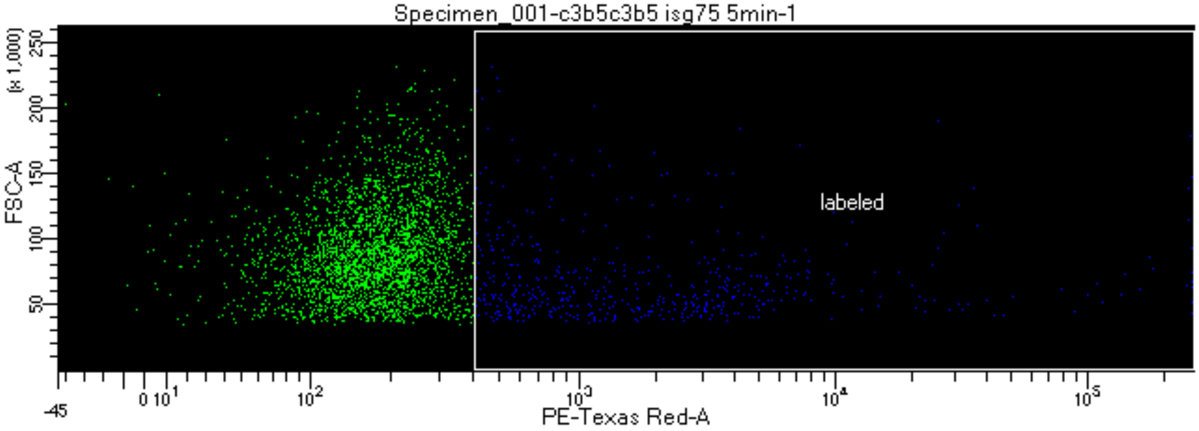

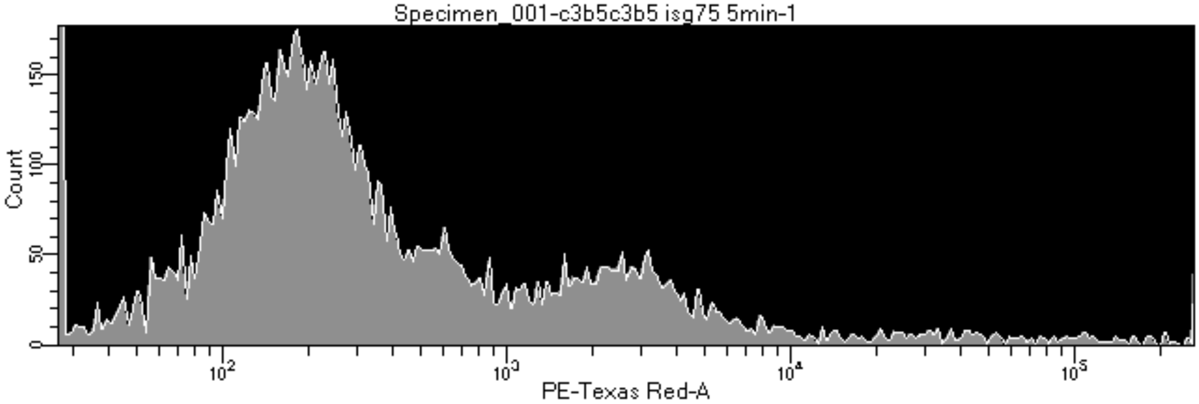

Tube: c3b5c3b5 isg75 5min-1

Population	#Events	%Parent	%Total
All Events	10,000	####	100.0
cells	3,417	34.2	34.2
singlets	3,321	97.2	33.2
labeled	501	15.1	5.0

BD FACSDiva 8.0.1

Specimen_001-c3b5c3b5 isg75 5min-2

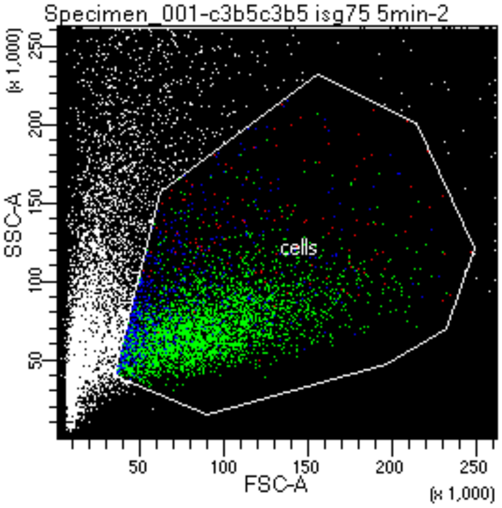

Specimen_001-c3b5c3b5 isg75 5min-2

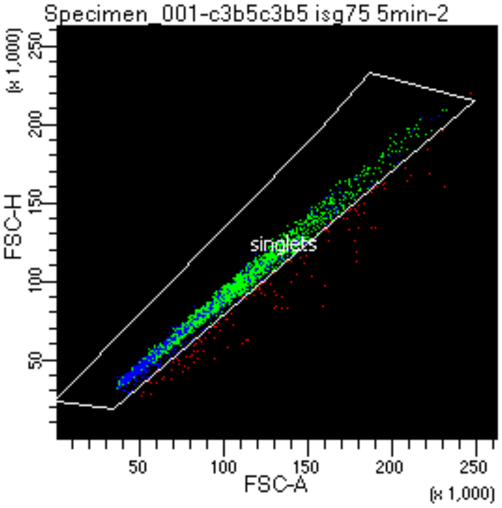

Specimen_001-c3b5c3b5 isg75 5min-2

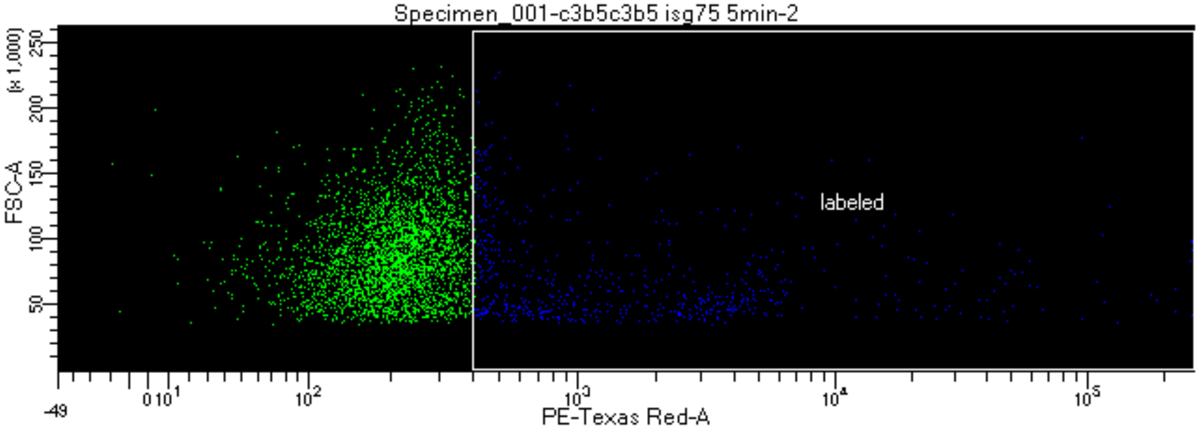

Specimen_001-c3b5c3b5 isg75 5min-2

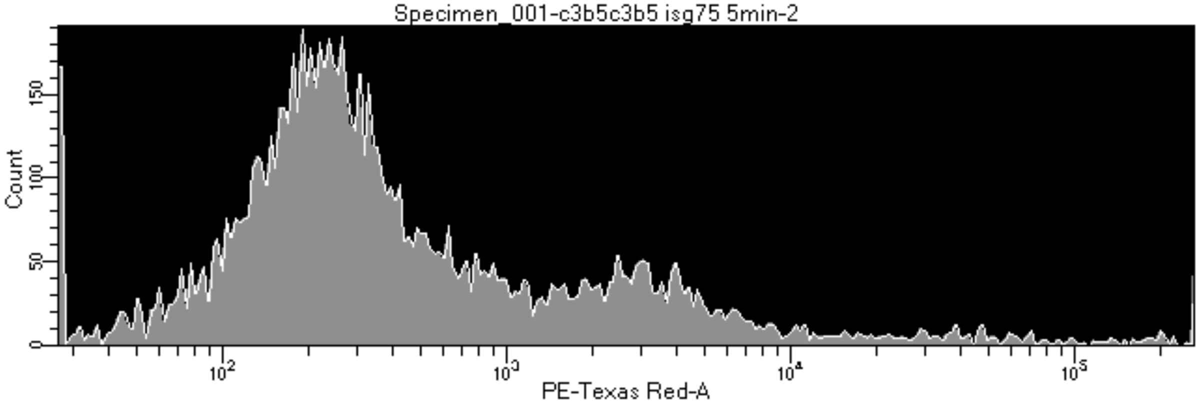

Tube: c3b5c3b5 isg75 5min-2

Population	#Events	%Parent	%Total
All Events	10,000	####	100.0
cells	3,642	36.4	36.4
singlets	3,511	96.4	35.1
labeled	591	16.8	5.9

BD FACSDiva 8.0.1

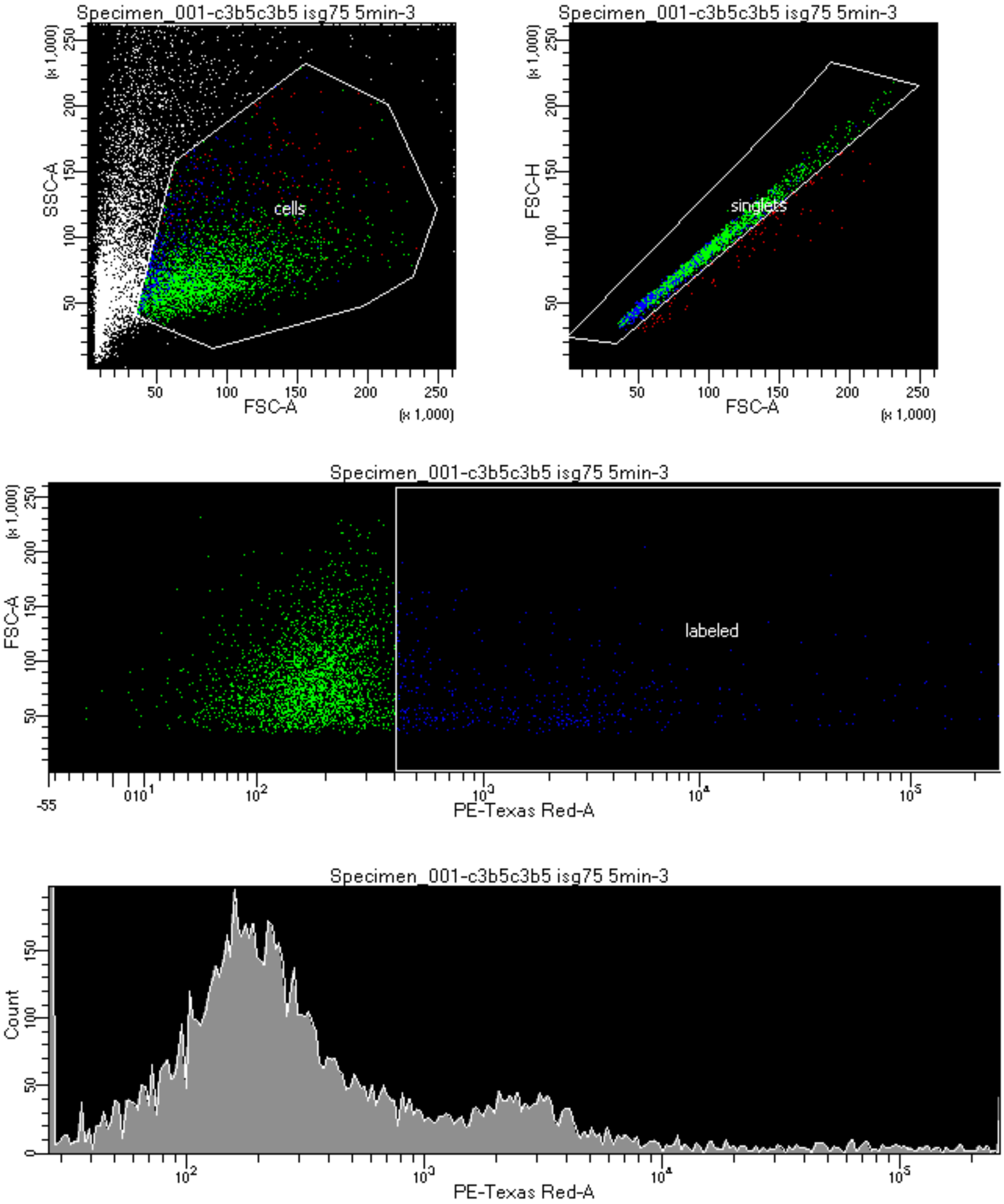

Tube: c3b5c3b5 isg75 5min-3

Population	#Events	%Parent	%Total
All Events	10,000	####	100.0
cells	3,092	30.9	30.9
singlets	2,982	96.4	29.8
labeled	401	13.4	4.0
