# Supplementary material for: Cryo-EM structures of Trypanosoma brucei gambiense ISG65 with human complement C3 and C3b and their roles in alternative pathway restriction
Source: Nat Commun. 2023 Apr 27;14:2403. doi: 10.1038/s41467-023-37988-7 (PMC10140031; doi:10.1038/s41467-023-37988-7)
Supplement: Supplementary file 5 — Supplementary Data 2 [file 41467_2023_37988_MOESM5_ESM.pdf]

Peptide mapping and HDX-MS analysis of the ISG65:C3d complex.
a Summary of the HDX-MS analysis. **b** Sequence coverage of ISG65 digested with nepenthesin in presence of 200 mM TCEP. In total, 254 peptides were detected. The sequence coverage is 97%. **c** Sequence coverage of C3d digested with nepenthesin in presence of 200 mM TCEP. In total, 270 peptides were detected. The sequence coverage is 98%. Blue boxes represent matched peptides. **d** Heat maps showing relative protection from deuterium uptake in ISG65 and C3d over 20 s, 120 s, 1200 s, 7200 s. The colour scale represents the level of protection in percent in ISG65:C3d complex compared to free ISG65 and free C3d. Red indicates high uptake protection. **e** Hydrogen/deuterium exchange plots for ISG65 (black) and ISG65:C3d (red) **f** Hydrogen/deuterium exchange plots for C3d (black) and ISG65:C3d (red). All time points were measured in triplicates. Figures were generated with *MSTools*[1]. The mass spectrometry proteomics data have been deposited to the ProteomeXchange Consortium via the PRIDE[2] partner repository with the dataset identifier PXD033606.
Source data are provided as a Source Data file.

a

Data Set	ISG65	C3d	ISG65:C3d
HDX reaction details	20 mM Hepes, 150 mM NaCl, pD (read) 7.1, 25 °C, 90% D_2O		
HDX time course (s)	20, 120, 1200, 7200		
HDX control samples	control	control	
Back-exchange (mean / IQR)	not measured		
# of Peptides	190	206	190
Sequence coverage	97%	97%	97%
Average peptide length / Redundancy	14.7 / 8.1	11.8 / 8.3	14.7 / 8.1
Replicates (biological or technical)	3 (technical)	3 (technical)	3 (technical)
Repeatability (average standard deviation)	0.112	0.044	0.066
Significant differences in HDX (delta HDX > X % D)			1.98%

b

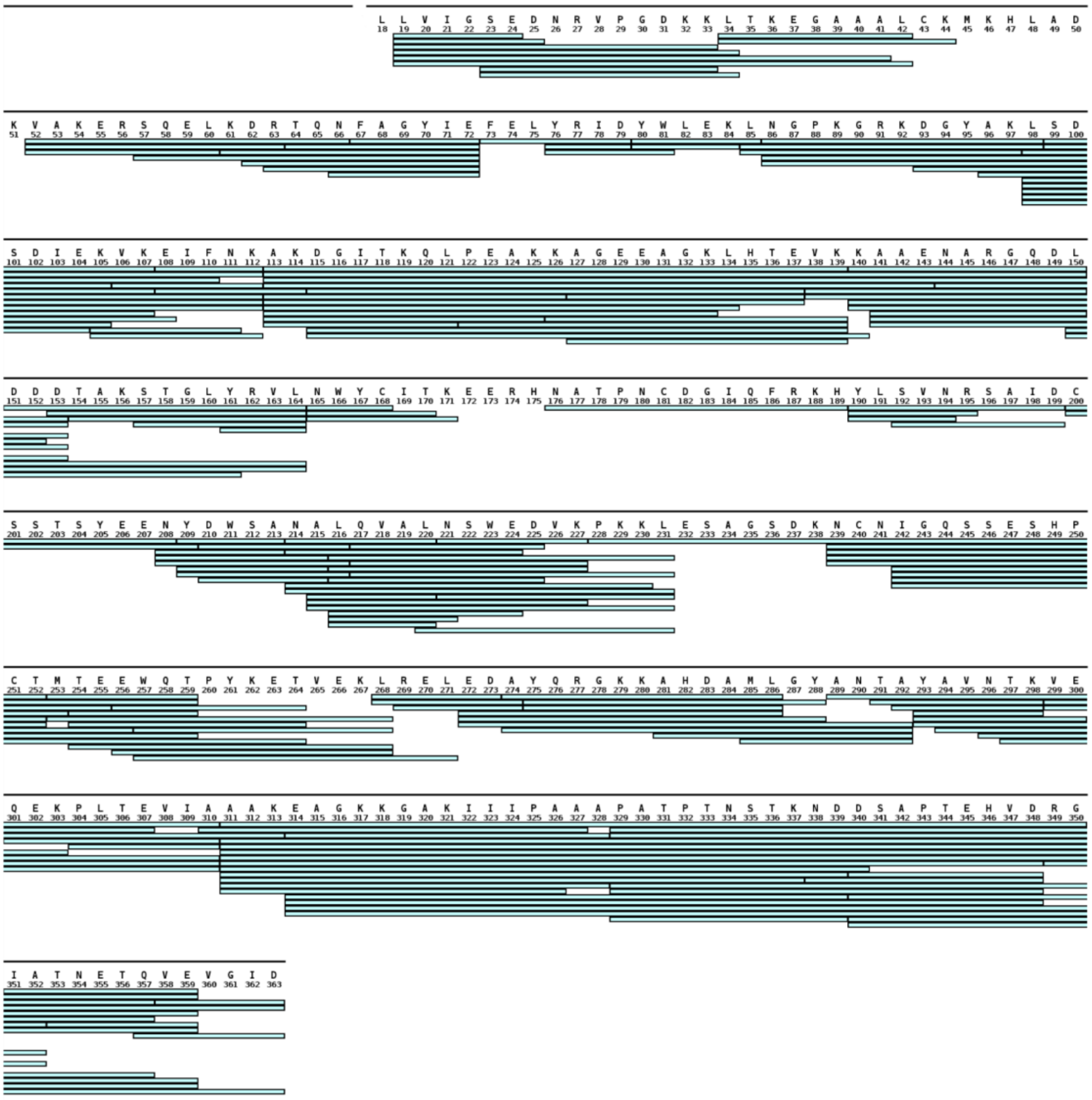
L L V I G S E D N R V P G D K K L T K E G A A A L C K M K H L A D
18 19 20 21 22 23 24 25 26 27 28 29 30 31 32 33 34 35 36 37 38 39 40 41 42 43 44 45 46 47 48 49 50
K V A K E R S Q E L K D R T Q N F A G Y I E F E L Y R I D Y W L E K L N G P K G R K D G Y A K L S D
51 52 53 54 55 56 57 58 59 60 61 62 63 64 65 66 67 68 69 70 71 72 73 74 75 76 77 78 79 80 81 82 83 84 85 86 87 88 89 90 91 92 93 94 95 96 97 98 99 100
S D I E K V K E I F N K A K D G I T K Q L P E A K K A G E E A G K L H T E V K K A A E N A R G Q D L
101 102 103 104 105 106 107 108 109 110 111 112 113 114 115 116 117 118 119 120 121 122 123 124 125 126 127 128 129 130 131 132 133 134 135 136 137 138 139 140 141 142 143 144 145 146 147 148 149 150
D D D T A K S T G L Y R V L N W Y C I T K E E R H N A T P N C D G I Q F R K H Y L S V N R S A I D C
151 152 153 154 155 156 157 158 159 160 161 162 163 164 165 166 167 168 169 170 171 172 173 174 175 176 177 178 179 180 181 182 183 184 185 186 187 188 189 190 191 192 193 194 195 196 197 198 199 200
S S T S Y E E N Y D W S A N A L Q V A L N S W E D V K P K K L E S A G S D K N C N I G Q S S E S H P
201 202 203 204 205 206 207 208 209 210 211 212 213 214 215 216 217 218 219 220 221 222 223 224 225 226 227 228 229 230 231 232 233 234 235 236 237 238 239 240 241 242 243 244 245 246 247 248 249 250
C T M T E E W Q T P Y K E T V E K L R E L E D A Y Q R G K K A H D A M L G Y A N T A Y A V N T K V E
251 252 253 254 255 256 257 258 259 260 261 262 263 264 265 266 267 268 269 270 271 272 273 274 275 276 277 278 279 280 281 282 283 284 285 286 287 288 289 290 291 292 293 294 295 296 297 298 299 300
Q E K P L T E V I A A A K E A G K K G A K I I I P A A A P A T P T N S T K N D D S A P T E H V D R G
301 302 303 304 305 306 307 308 309 310 311 312 313 314 315 316 317 318 319 320 321 322 323 324 325 326 327 328 329 330 331 332 333 334 335 336 337 338 339 340 341 342 343 344 345 346 347 348 349 350
I A T N E T Q V E V G I D
351 352 353 354 355 356 357 358 359 360 361 362 363

c

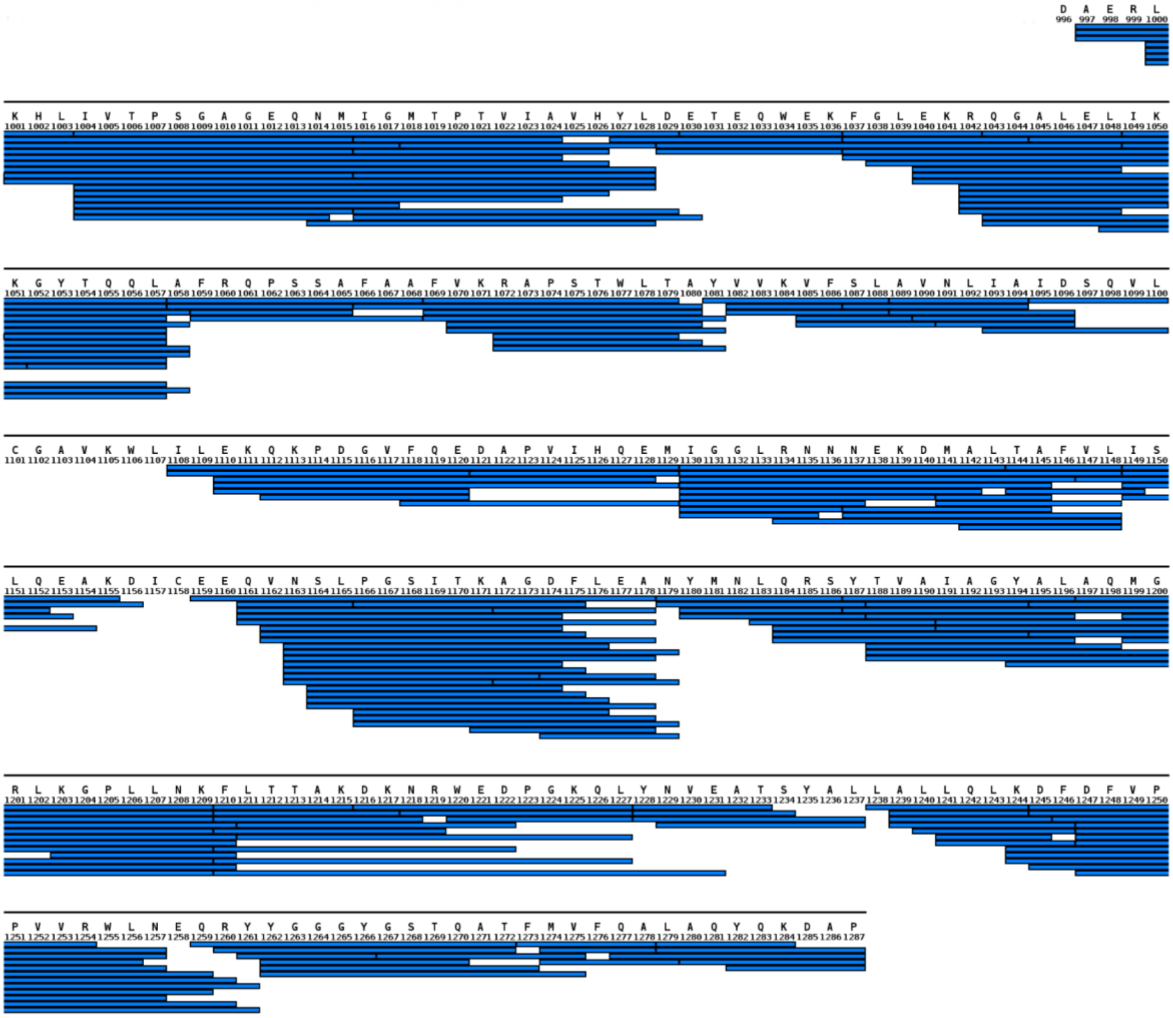

d

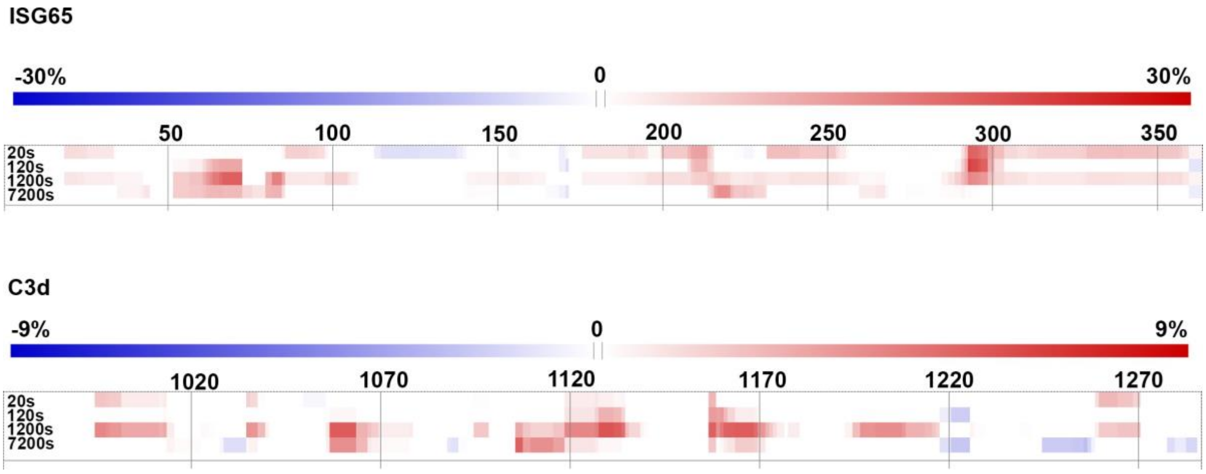
ISG65
-30%
0
30%
50
100
150
200
250
300
350
20s
120s
1200s
7200s
C3d
-9%
0
9%
1020
1070
1120
1170
1220
1270
20s
120s
1200s
7200s

e

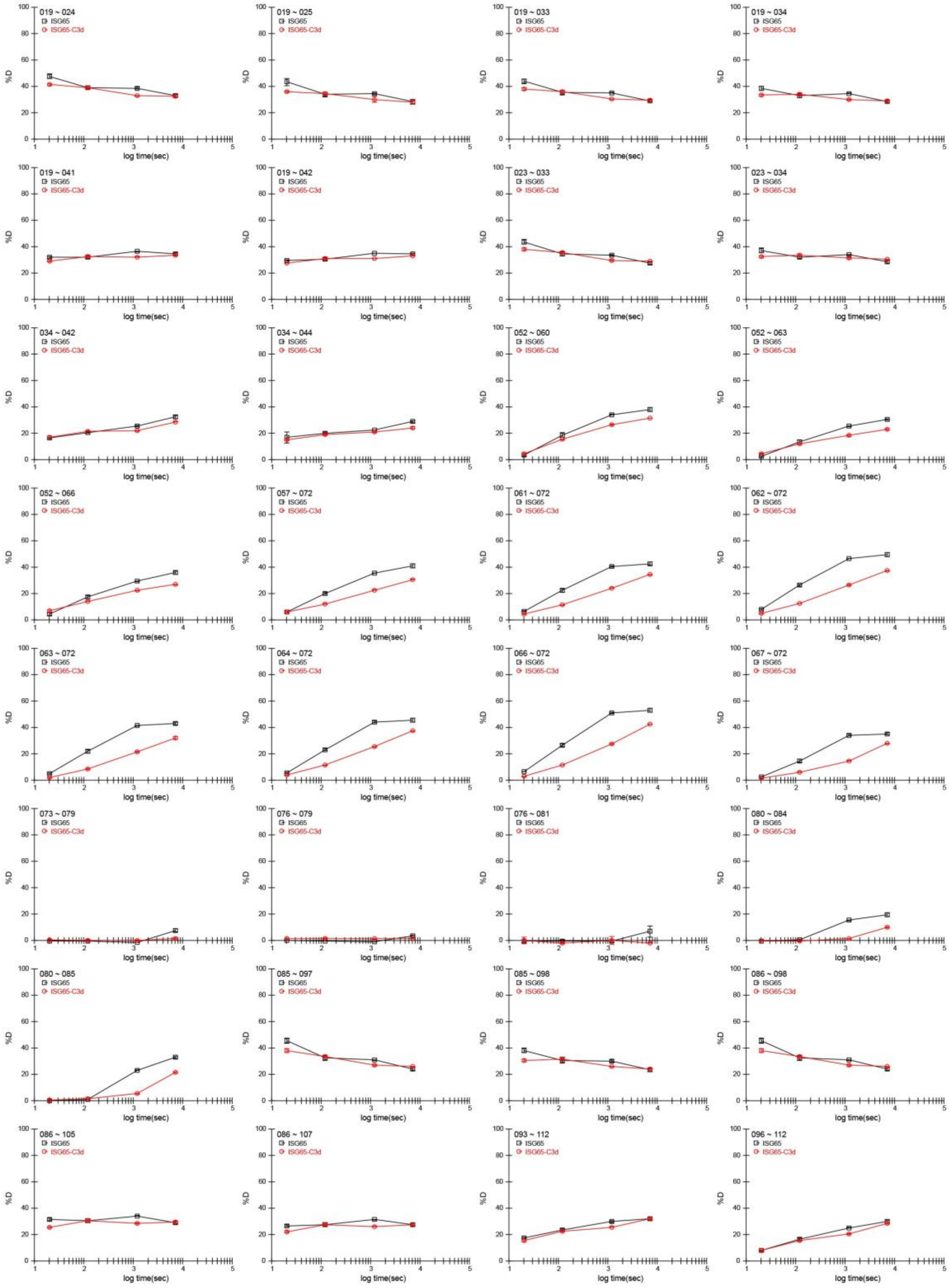
019 ~ 024
019 ~ 025
019 ~ 033
019 ~ 034
019 ~ 041
019 ~ 042
023 ~ 033
023 ~ 034
034 ~ 042
034 ~ 044
052 ~ 060
052 ~ 063
052 ~ 066
057 ~ 072
061 ~ 072
062 ~ 072
063 ~ 072
064 ~ 072
066 ~ 072
067 ~ 072
073 ~ 079
076 ~ 079
076 ~ 081
080 ~ 084
080 ~ 085
085 ~ 097
085 ~ 098
086 ~ 098
086 ~ 105
086 ~ 107
093 ~ 112
096 ~ 112
ISG65
ISG65-C3d
%D
log time(sec)

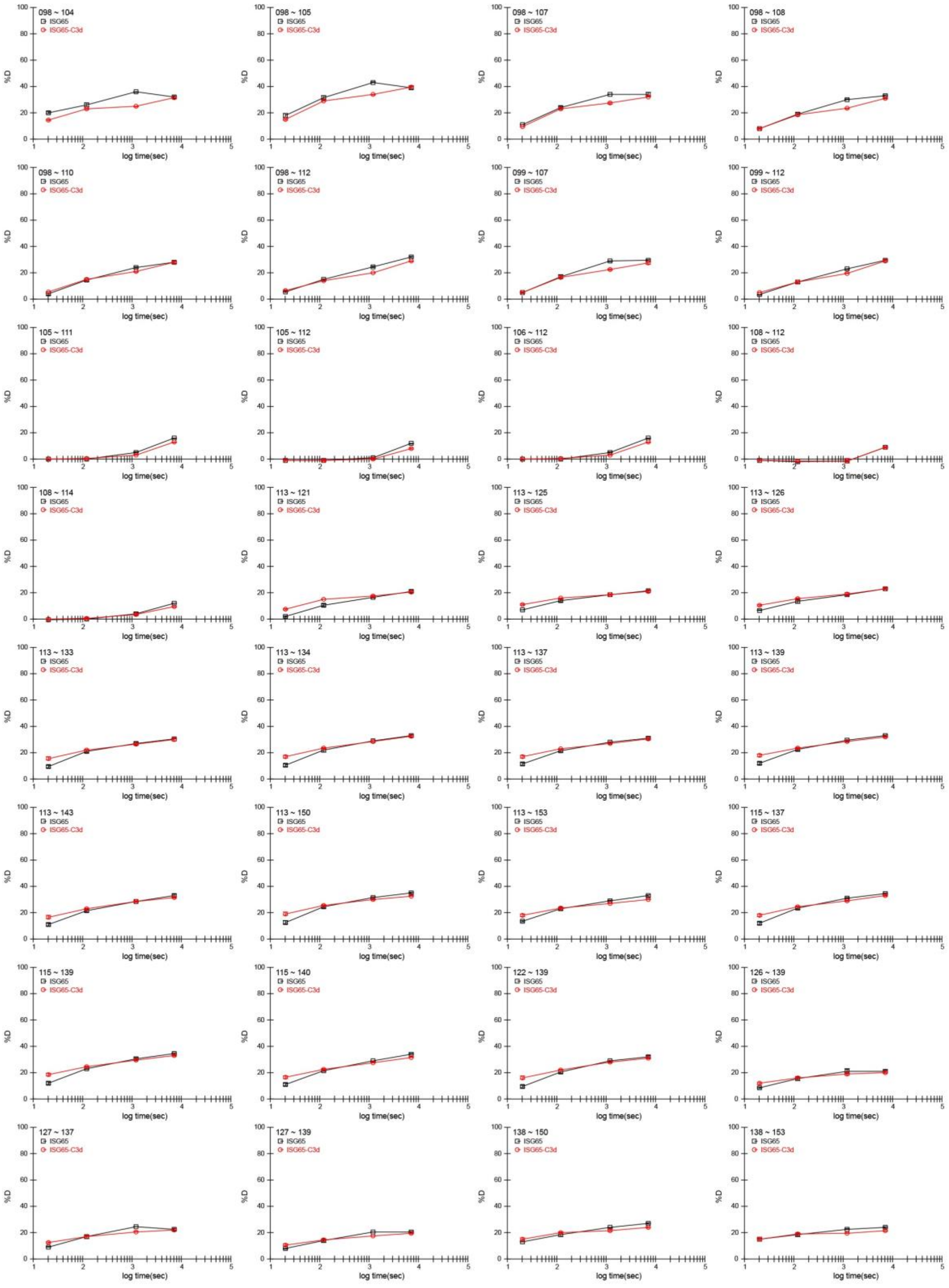
098 ~ 104
098 ~ 105
098 ~ 107
098 ~ 108
098 ~ 110
098 ~ 112
099 ~ 107
099 ~ 112
105 ~ 111
105 ~ 112
106 ~ 112
108 ~ 112
108 ~ 114
113 ~ 121
113 ~ 125
113 ~ 126
113 ~ 133
113 ~ 134
113 ~ 137
113 ~ 139
113 ~ 143
113 ~ 150
113 ~ 153
115 ~ 137
115 ~ 139
115 ~ 140
122 ~ 139
126 ~ 139
127 ~ 137
127 ~ 139
138 ~ 150
138 ~ 153
ISG65
ISG65-C3d
%D
log time(sec)

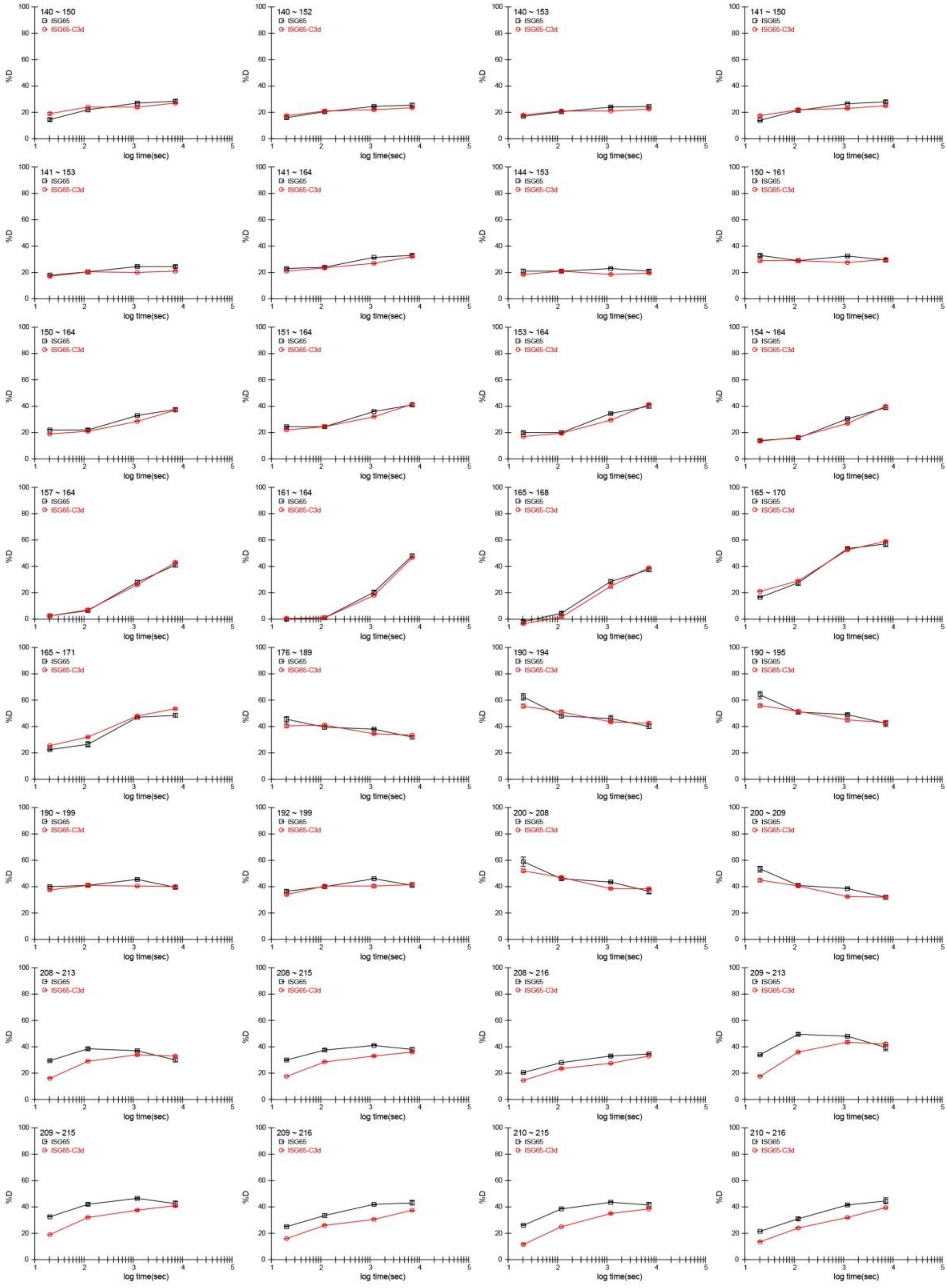
140 ~ 150
140 ~ 152
140 ~ 153
141 ~ 150
141 ~ 153
141 ~ 164
144 ~ 153
150 ~ 161
150 ~ 164
151 ~ 164
153 ~ 164
154 ~ 164
157 ~ 164
161 ~ 164
165 ~ 168
165 ~ 170
165 ~ 171
176 ~ 189
190 ~ 194
190 ~ 195
190 ~ 199
192 ~ 199
200 ~ 208
200 ~ 209
208 ~ 213
208 ~ 215
208 ~ 216
209 ~ 213
209 ~ 215
209 ~ 216
210 ~ 215
210 ~ 216
ISG65
ISG65-C3d
%D
log time(sec)

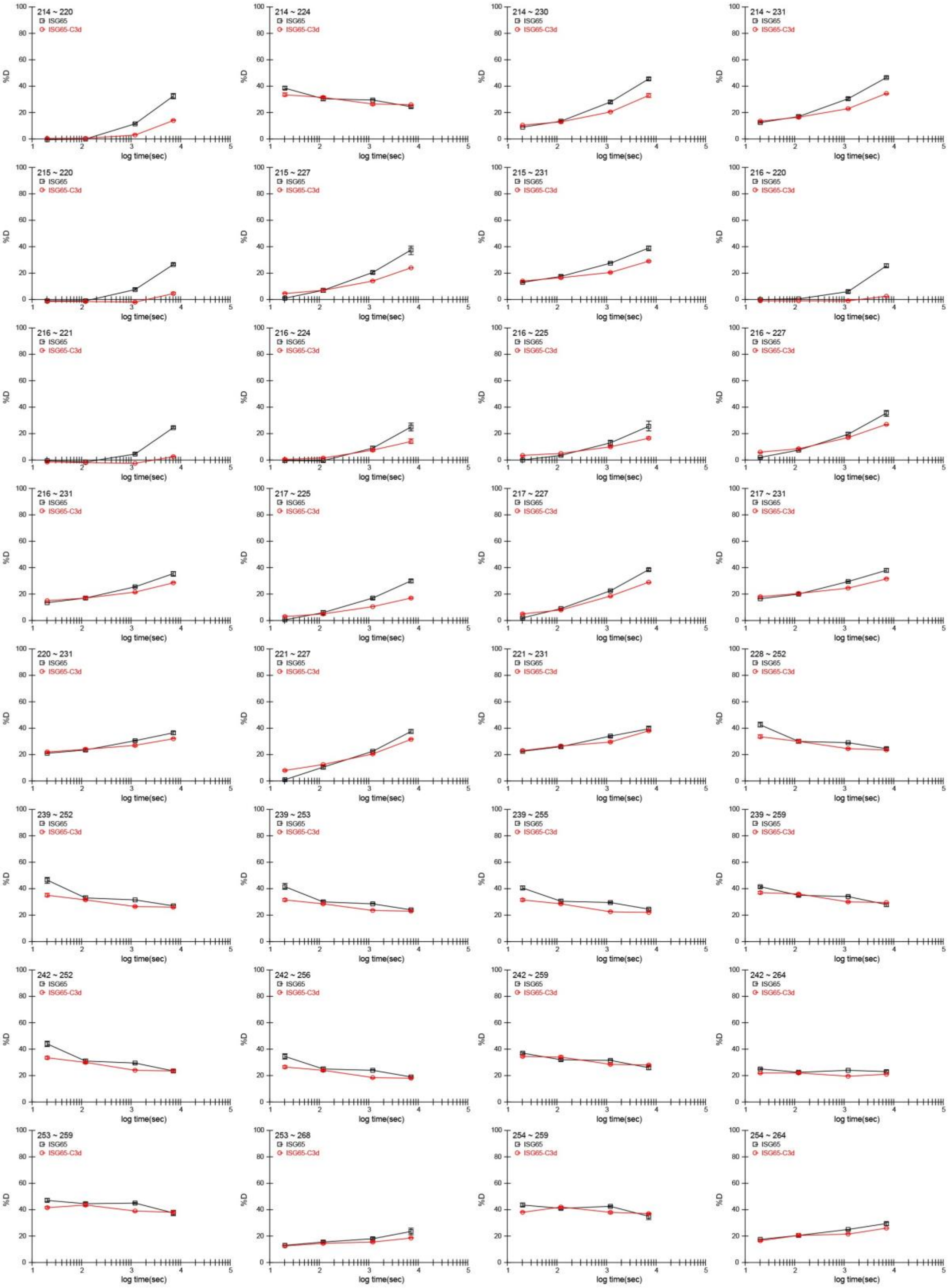
214 ~ 220
214 ~ 224
214 ~ 230
214 ~ 231
215 ~ 220
215 ~ 227
215 ~ 231
216 ~ 220
216 ~ 221
216 ~ 224
216 ~ 225
216 ~ 227
216 ~ 231
217 ~ 225
217 ~ 227
217 ~ 231
220 ~ 231
221 ~ 227
221 ~ 231
228 ~ 252
239 ~ 252
239 ~ 253
239 ~ 255
239 ~ 259
242 ~ 252
242 ~ 256
242 ~ 259
242 ~ 264
253 ~ 259
253 ~ 268
254 ~ 259
254 ~ 264
ISG65
ISG65-C3d
%D
log time(sec)

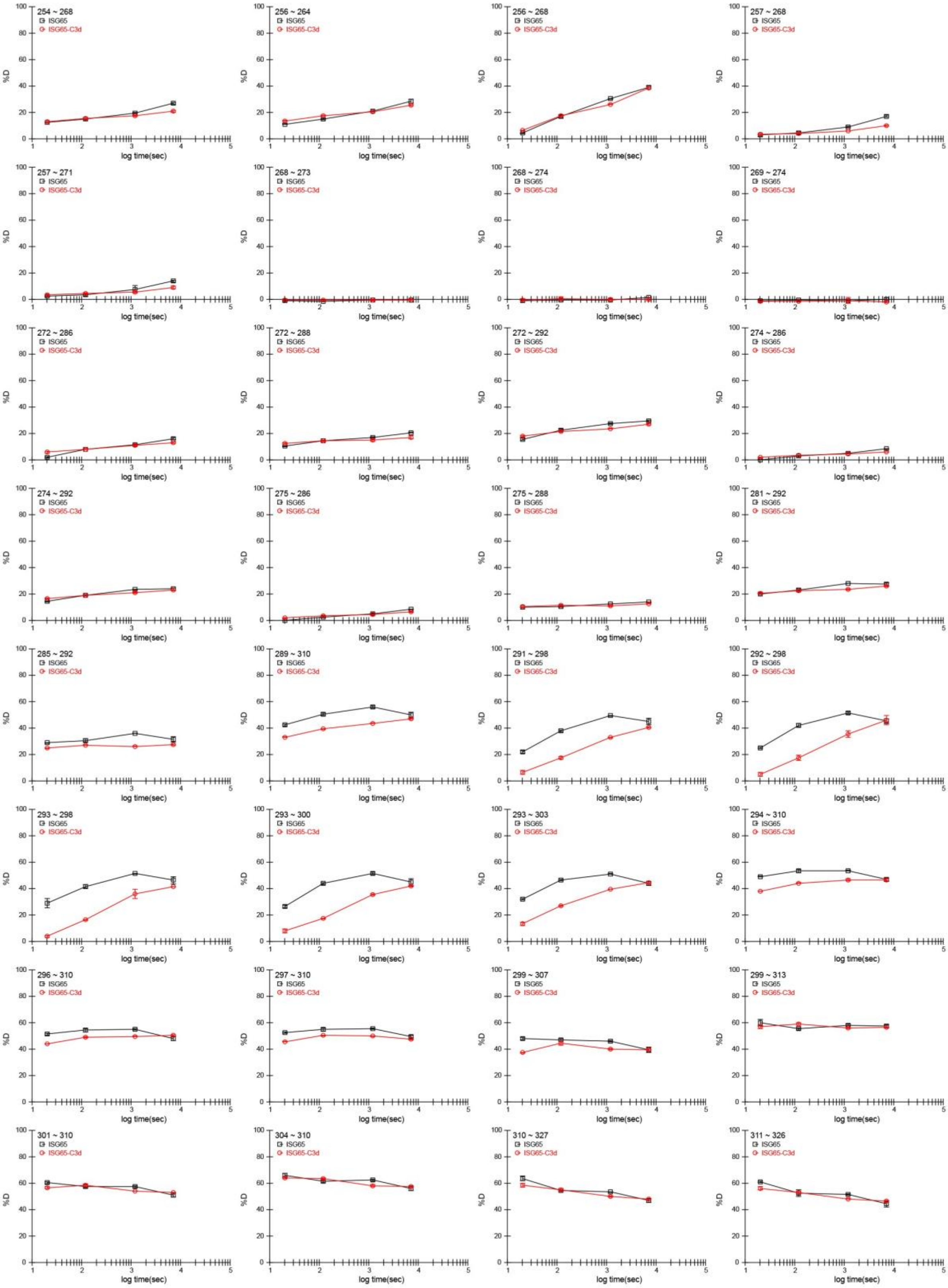
254 ~ 268
256 ~ 264
256 ~ 268
257 ~ 268
257 ~ 271
268 ~ 273
268 ~ 274
269 ~ 274
272 ~ 286
272 ~ 288
272 ~ 292
274 ~ 286
274 ~ 292
275 ~ 286
275 ~ 288
281 ~ 292
285 ~ 292
289 ~ 310
291 ~ 298
292 ~ 298
293 ~ 298
293 ~ 300
293 ~ 303
294 ~ 310
296 ~ 310
297 ~ 310
299 ~ 307
299 ~ 313
301 ~ 310
304 ~ 310
310 ~ 327
311 ~ 326
ISG65
ISG65-C3d
%D
log time(sec)

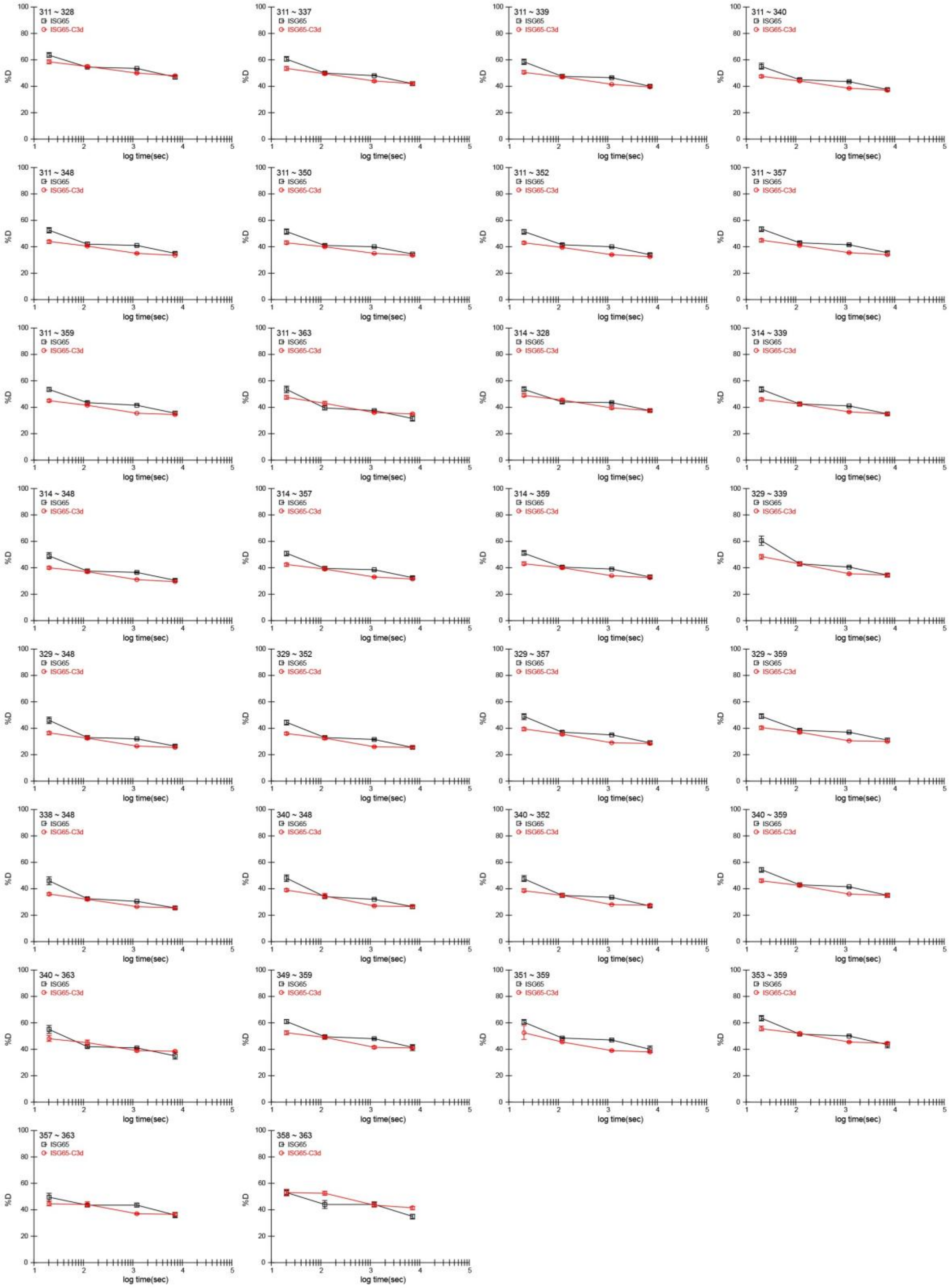
311 ~ 328
311 ~ 337
311 ~ 339
311 ~ 340
311 ~ 348
311 ~ 350
311 ~ 352
311 ~ 357
311 ~ 359
311 ~ 363
314 ~ 328
314 ~ 339
314 ~ 348
314 ~ 357
314 ~ 359
329 ~ 339
329 ~ 348
329 ~ 352
329 ~ 357
329 ~ 359
338 ~ 348
340 ~ 348
340 ~ 352
340 ~ 359
340 ~ 363
349 ~ 359
351 ~ 359
353 ~ 359
357 ~ 363
358 ~ 363
ISG65
ISG65-C3d
%D
log time(sec)

f

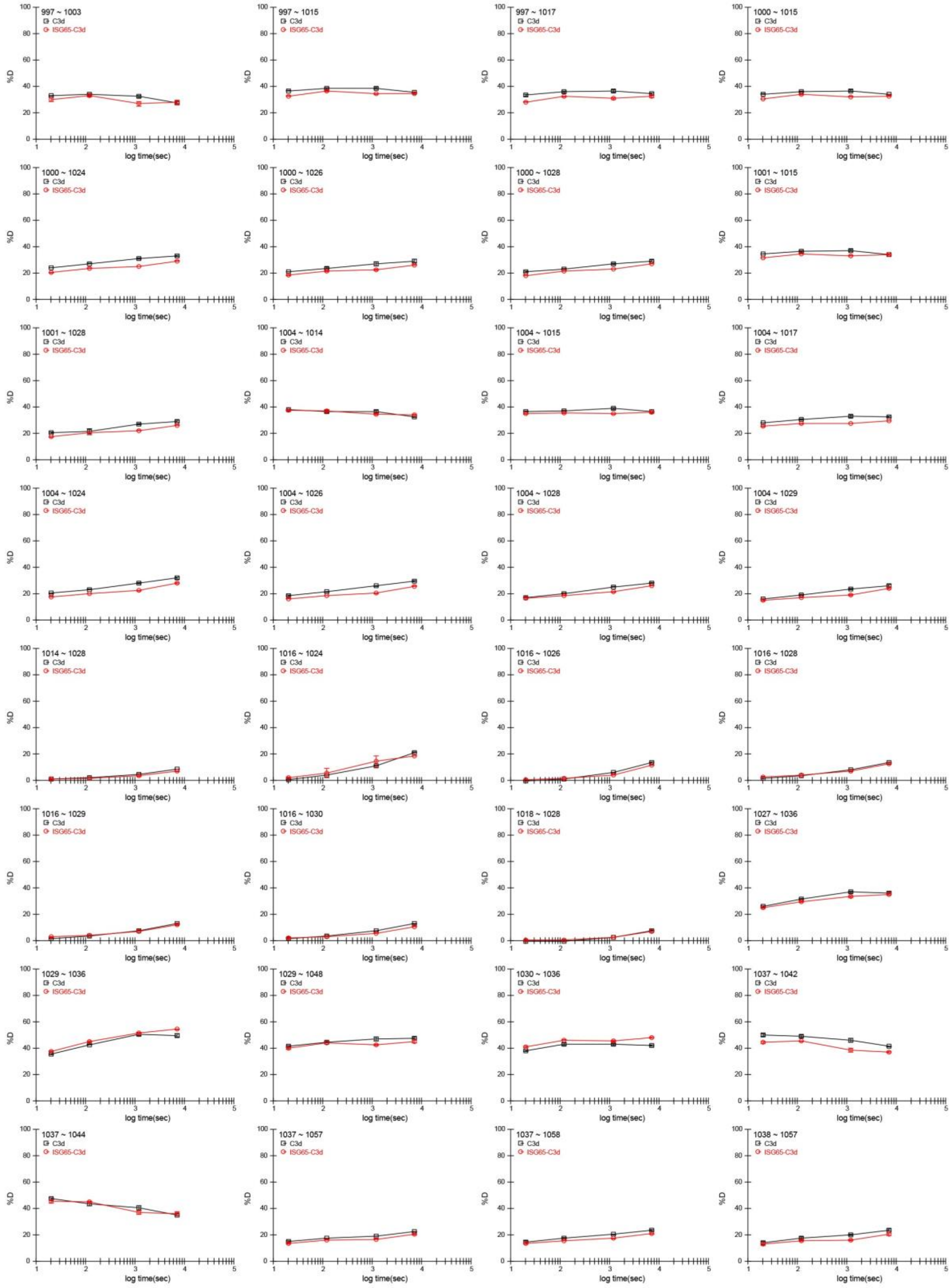

997 ~ 1003
997 ~ 1015
997 ~ 1017
1000 ~ 1015
1000 ~ 1024
1000 ~ 1026
1000 ~ 1028
1001 ~ 1015
1001 ~ 1028
1004 ~ 1014
1004 ~ 1015
1004 ~ 1017
1004 ~ 1024
1004 ~ 1026
1004 ~ 1028
1004 ~ 1029
1014 ~ 1028
1016 ~ 1024
1016 ~ 1026
1016 ~ 1028
1016 ~ 1029
1016 ~ 1030
1018 ~ 1028
1027 ~ 1036
1029 ~ 1036
1029 ~ 1048
1030 ~ 1036
1037 ~ 1042
1037 ~ 1044
1037 ~ 1057
1037 ~ 1058
1038 ~ 1057
C3d
ISG65-C3d
%D
log time(sec)

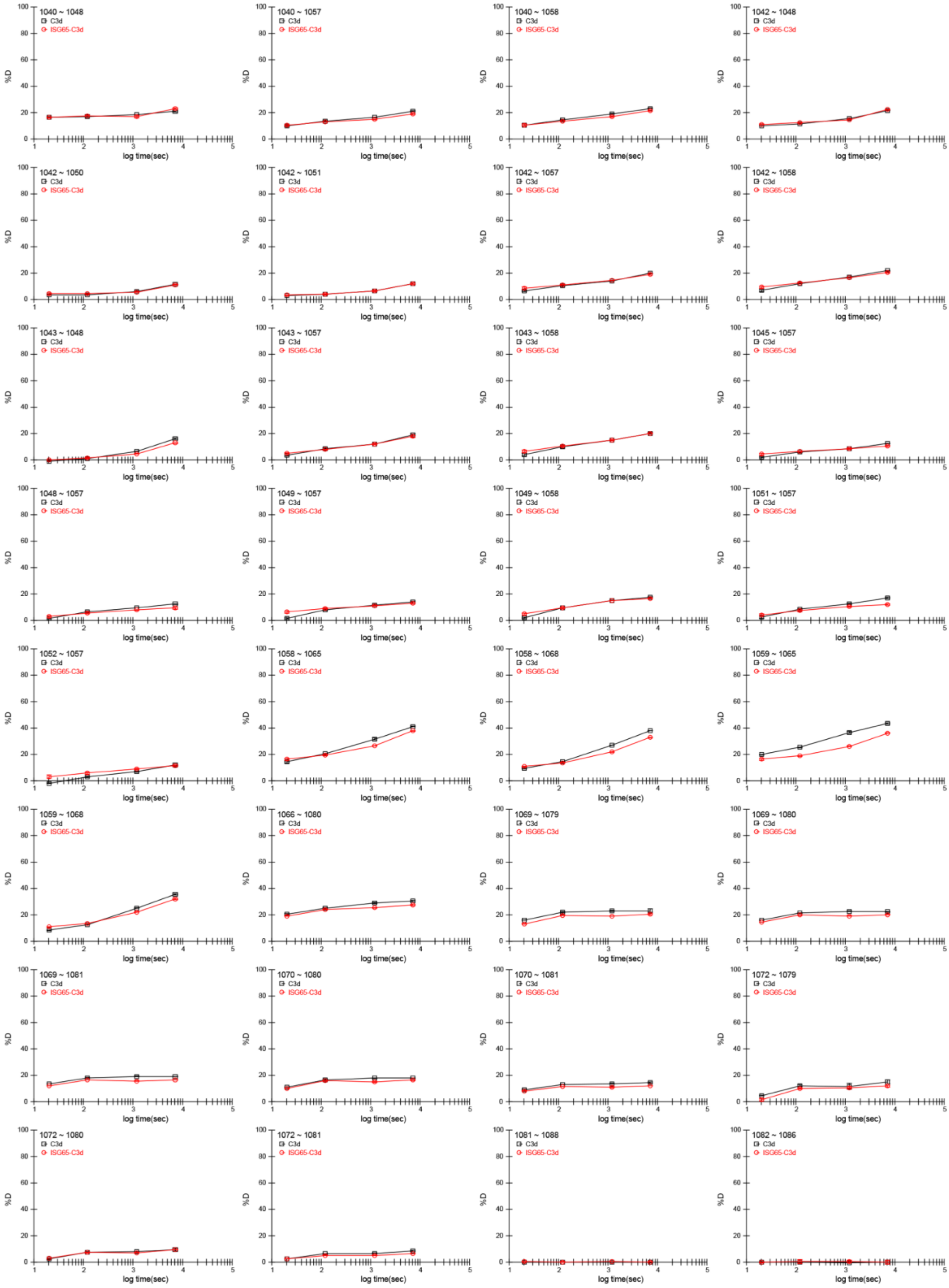
1040 ~ 1048
1040 ~ 1057
1040 ~ 1058
1042 ~ 1048
1042 ~ 1050
1042 ~ 1051
1042 ~ 1057
1042 ~ 1058
1043 ~ 1048
1043 ~ 1057
1043 ~ 1058
1045 ~ 1057
1048 ~ 1057
1049 ~ 1057
1049 ~ 1058
1051 ~ 1057
1052 ~ 1057
1058 ~ 1065
1058 ~ 1068
1059 ~ 1065
1059 ~ 1068
1066 ~ 1080
1069 ~ 1079
1069 ~ 1080
1069 ~ 1081
1070 ~ 1080
1070 ~ 1081
1072 ~ 1079
1072 ~ 1080
1072 ~ 1081
1081 ~ 1088
1082 ~ 1086
C3d
ISG65-C3d
%D
log time(sec)

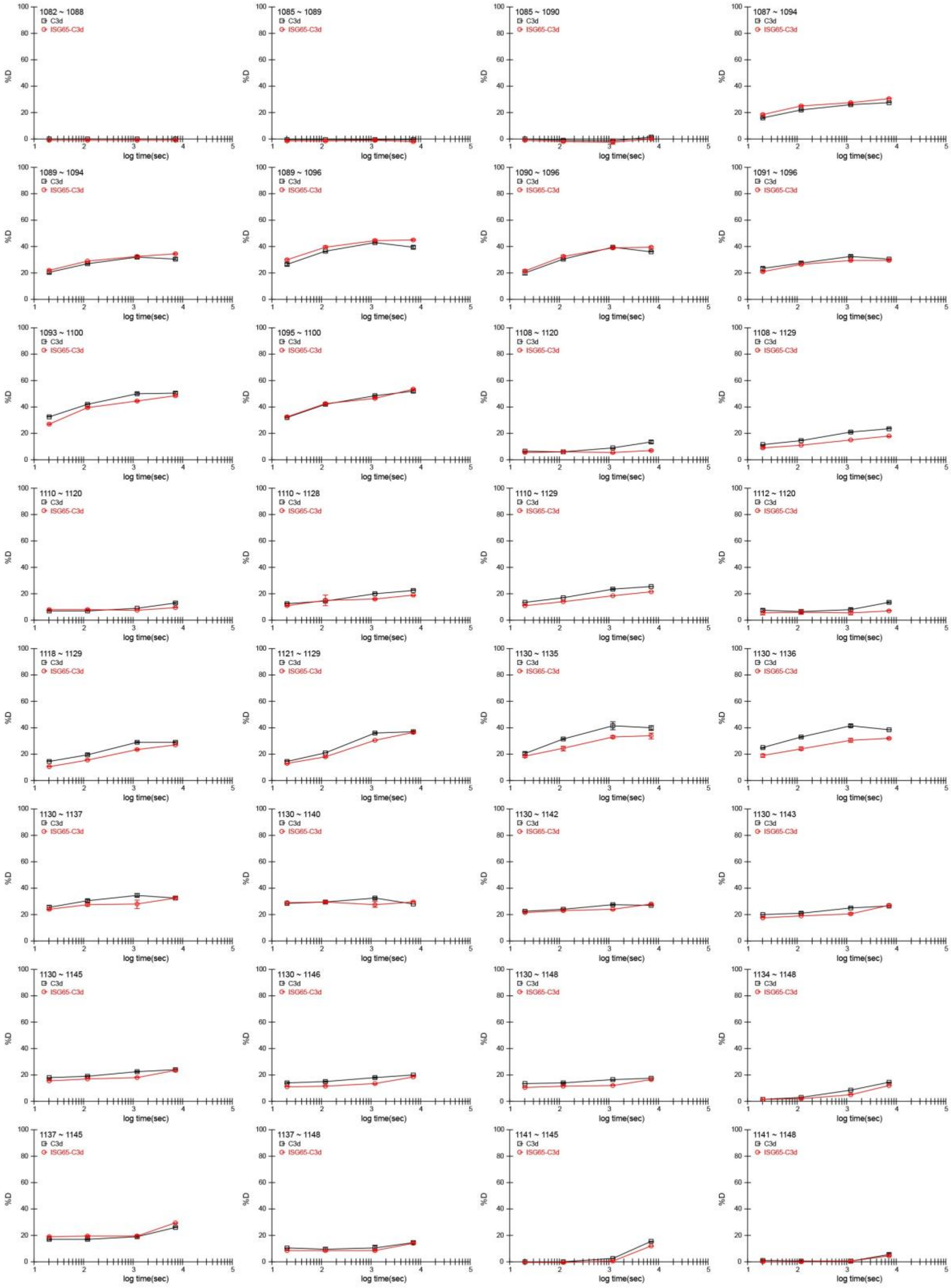
1082 ~ 1088
1085 ~ 1089
1085 ~ 1090
1087 ~ 1094
1089 ~ 1094
1089 ~ 1096
1090 ~ 1096
1091 ~ 1096
1093 ~ 1100
1095 ~ 1100
1108 ~ 1120
1108 ~ 1129
1110 ~ 1120
1110 ~ 1128
1110 ~ 1129
1112 ~ 1120
1118 ~ 1129
1121 ~ 1129
1130 ~ 1135
1130 ~ 1136
1130 ~ 1137
1130 ~ 1140
1130 ~ 1142
1130 ~ 1143
1130 ~ 1145
1130 ~ 1146
1130 ~ 1148
1134 ~ 1148
1137 ~ 1145
1137 ~ 1148
1141 ~ 1145
1141 ~ 1148
C3d
ISG65-C3d
%D
log time(sec)

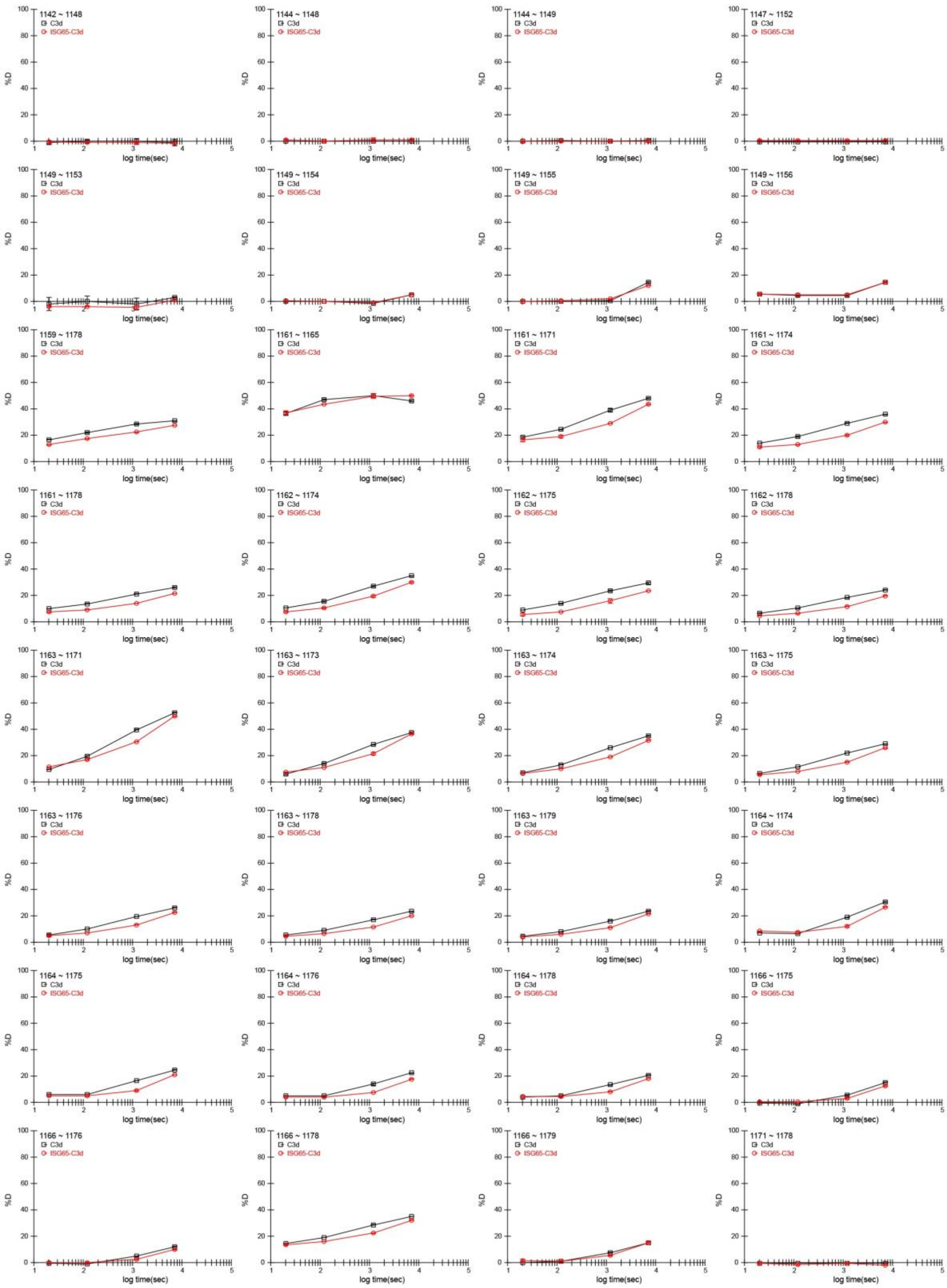
1142 ~ 1148
1144 ~ 1148
1144 ~ 1149
1147 ~ 1152
1149 ~ 1153
1149 ~ 1154
1149 ~ 1155
1149 ~ 1156
1159 ~ 1178
1161 ~ 1165
1161 ~ 1171
1161 ~ 1174
1161 ~ 1178
1162 ~ 1174
1162 ~ 1175
1162 ~ 1178
1163 ~ 1171
1163 ~ 1173
1163 ~ 1174
1163 ~ 1175
1163 ~ 1176
1163 ~ 1178
1163 ~ 1179
1164 ~ 1174
1164 ~ 1175
1164 ~ 1176
1164 ~ 1178
1166 ~ 1175
1166 ~ 1176
1166 ~ 1178
1166 ~ 1179
1171 ~ 1178
C3d
ISG65-C3d
%D
log time(sec)

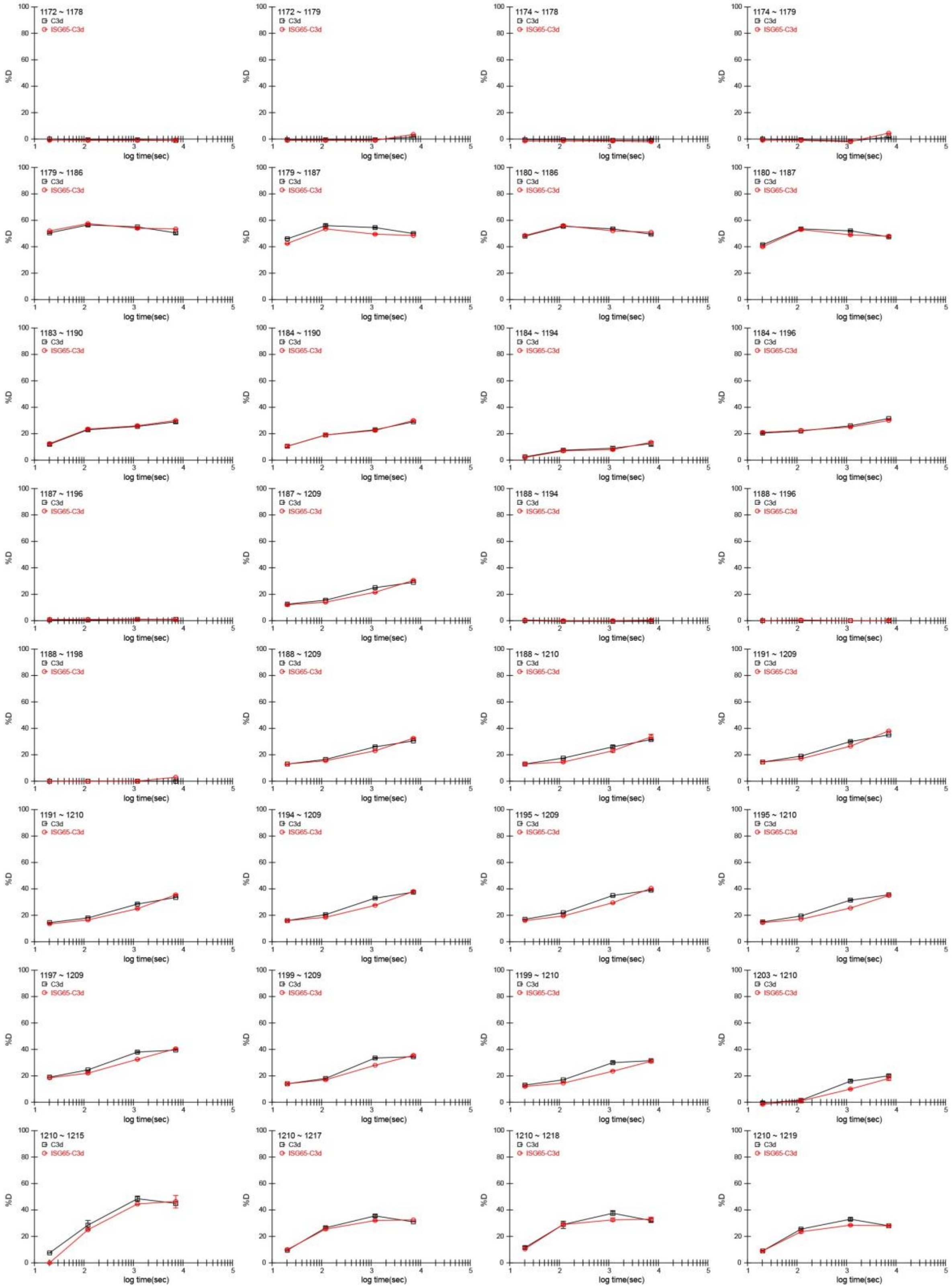

1172 ~ 1178
1172 ~ 1179
1174 ~ 1178
1174 ~ 1179
1179 ~ 1186
1179 ~ 1187
1180 ~ 1186
1180 ~ 1187
1183 ~ 1190
1184 ~ 1190
1184 ~ 1194
1184 ~ 1196
1187 ~ 1196
1187 ~ 1209
1188 ~ 1194
1188 ~ 1196
1188 ~ 1198
1188 ~ 1209
1188 ~ 1210
1191 ~ 1209
1191 ~ 1210
1194 ~ 1209
1195 ~ 1209
1195 ~ 1210
1197 ~ 1209
1199 ~ 1209
1199 ~ 1210
1203 ~ 1210
1210 ~ 1215
1210 ~ 1217
1210 ~ 1218
1210 ~ 1219
C3d
ISG65-C3d
%D
log time(sec)

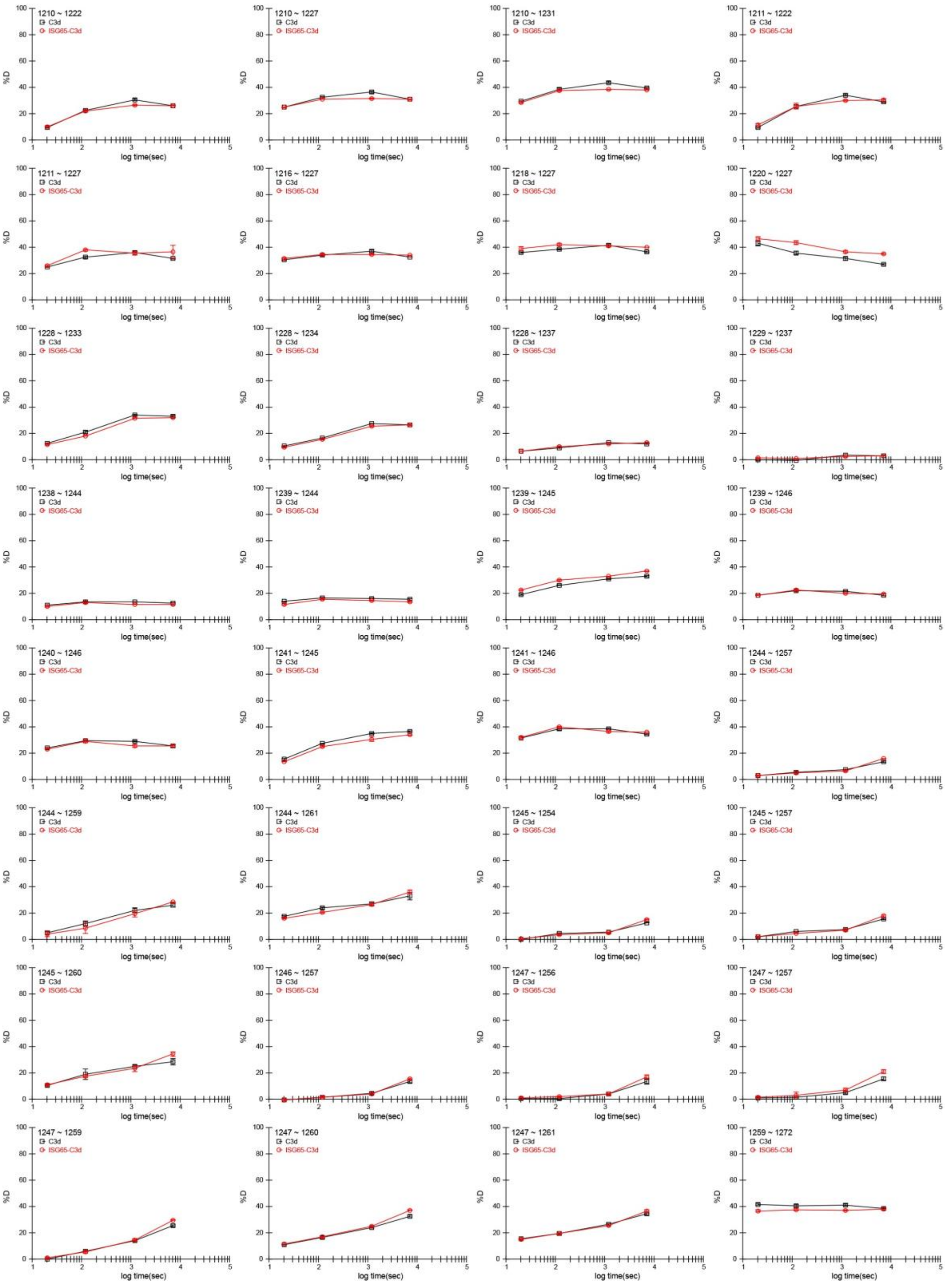
1210 ~ 1222
1210 ~ 1227
1210 ~ 1231
1211 ~ 1222
1211 ~ 1227
1216 ~ 1227
1218 ~ 1227
1220 ~ 1227
1228 ~ 1233
1228 ~ 1234
1228 ~ 1237
1229 ~ 1237
1238 ~ 1244
1239 ~ 1244
1239 ~ 1245
1239 ~ 1246
1240 ~ 1246
1241 ~ 1245
1241 ~ 1246
1244 ~ 1257
1244 ~ 1259
1244 ~ 1261
1245 ~ 1254
1245 ~ 1257
1245 ~ 1260
1246 ~ 1257
1247 ~ 1256
1247 ~ 1257
1247 ~ 1259
1247 ~ 1260
1247 ~ 1261
1259 ~ 1272
C3d
ISG65-C3d
%D
log time(sec)

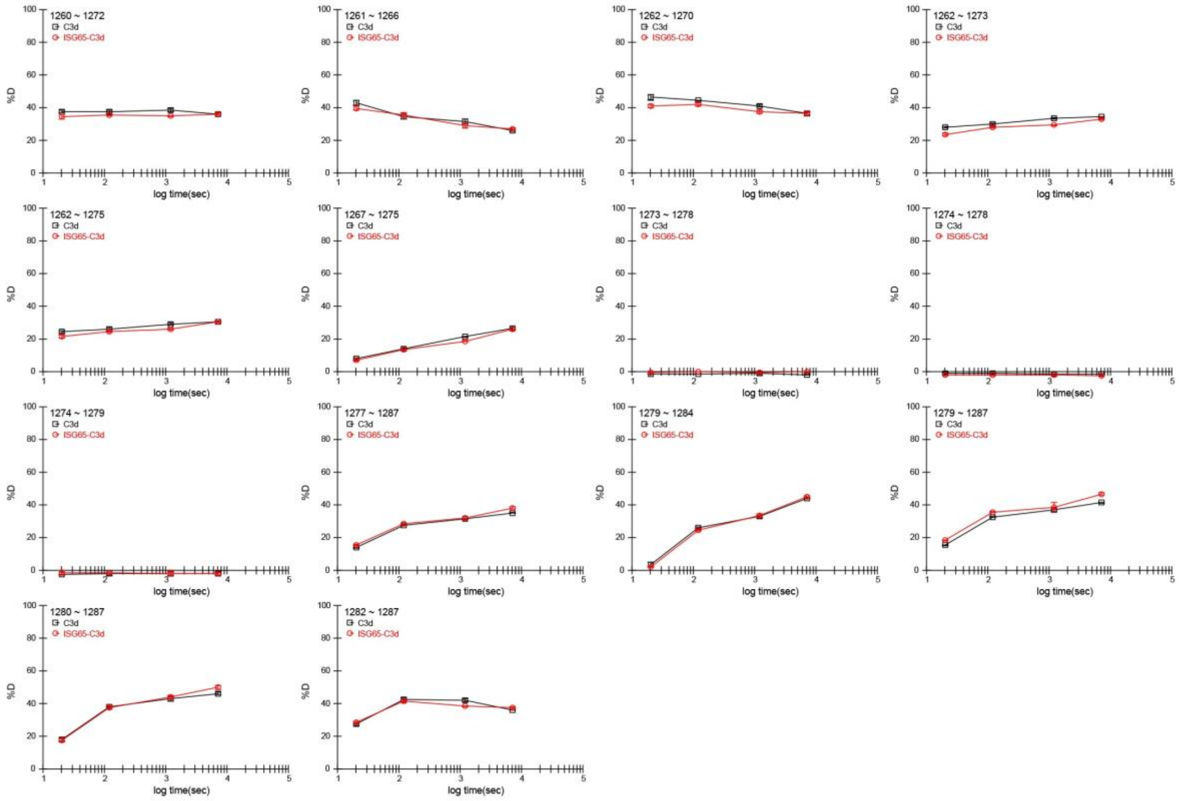

Supplementary References

1 Daniel Kavan, P. M. MSTools—Web based application for visualization and presentation of HXMS data. *International Journal of Mass Spectrometry* **302**, 53-58, doi:doi.org/10.1016/j.ijms.2010.07.030 (2011).

2 Perez-Riverol, Y. *et al.* The PRIDE database resources in 2022: a hub for mass spectrometry-based proteomics evidences. *Nucleic Acids Res* **50**, D543-D552, doi:10.1093/nar/gkab1038 (2022).
