# Supplementary material for: Cryo-EM structures of Trypanosoma brucei gambiense ISG65 with human complement C3 and C3b and their roles in alternative pathway restriction
Source: Nat Commun. 2023 Apr 27;14:2403. doi: 10.1038/s41467-023-37988-7 (PMC10140031; doi:10.1038/s41467-023-37988-7)
Supplement: Supplementary file 6 — Reporting Summary [file 41467_2023_37988_MOESM6_ESM.pdf]

nature portfolio

Corresponding author(s): Sebastian Zoll

Last updated by author(s): Mar 29, 2023

Reporting Summary

Nature Portfolio wishes to improve the reproducibility of the work that we publish. This form provides structure for consistency and transparency in reporting. For further information on Nature Portfolio policies, see our Editorial Policies and the Editorial Policy Checklist.

Statistics

For all statistical analyses, confirm that the following items are present in the figure legend, table legend, main text, or Methods section.

n/a	Confirmed	
☐	☒	The exact sample size (n) for each experimental group/condition, given as a discrete number and unit of measurement
☒	☐	A statement on whether measurements were taken from distinct samples or whether the same sample was measured repeatedly
☐	☒	The statistical test(s) used AND whether they are one- or two-sided<br>*Only common tests should be described solely by name; describe more complex techniques in the Methods section.*
☒	☐	A description of all covariates tested
☒	☐	A description of any assumptions or corrections, such as tests of normality and adjustment for multiple comparisons
☐	☒	A full description of the statistical parameters including central tendency (e.g. means) or other basic estimates (e.g. regression coefficient) AND variation (e.g. standard deviation) or associated estimates of uncertainty (e.g. confidence intervals)
☒	☐	For null hypothesis testing, the test statistic (e.g. F, t, r) with confidence intervals, effect sizes, degrees of freedom and P value noted<br>*Give P values as exact values whenever suitable.*
☒	☐	For Bayesian analysis, information on the choice of priors and Markov chain Monte Carlo settings
☒	☐	For hierarchical and complex designs, identification of the appropriate level for tests and full reporting of outcomes
☒	☐	Estimates of effect sizes (e.g. Cohen's d, Pearson's r), indicating how they were calculated

Our web collection on statistics for biologists contains articles on many of the points above.

Software and code

Policy information about availability of computer code

Data collection

All Software used is either commercial or open-source. No custom software was used.
Single Particle cryoEM data was collected using automated data collection software EPU [version 2.10.0.5] (ThermoFisher Scientific) and SerialEM [version 3.8] software.

Data analysis

All Software used is either commercial or open-source. No custom software was used.
CryoEM data analysis was performed using the cryoSPARC software suite (versions 3.2 and later), Relion 3.1, the crYOLO particle picker (version 1.6.1) (part of the sphire software suite). Model building and refinement was performed using Phenix (v1.19), REFMAC5, Coot (0.9.5) and AlphaFold2.
SAXS data was processed and analysed using the ATSAS package 3.2.1 and BioXTAS RAW 2.1.4
SPR data was analysed using the BIAcore T200 evaluation 2.0 software .
Data analysis of the Haemolytic assay was performed in Graphpad Prism [version 9.4.1 and later].
Proteome Discoverer 2.3 was used for peptide and protein identification after LC-MS.
Fluorescence microscopy images were processed using Fiji (version 2.9.0).
Flow cytometry data was processed using BD FACSDiva software (version 8.0.1)
Intact mass spectra were processed using MassLynx 4.2. The final spectra were created using mMass (5.5.0).
Disulphide mapping MS data were processed using DataAnalysis 5.0 software (Bruker Daltonics) and ProteinScape 4.0
HDX-MS data was analysed using DataAnalysis 4.2 software (Bruker Daltonics) and DeutEx 1.0

For manuscripts utilizing custom algorithms or software that are central to the research but not yet described in published literature, software must be made available to editors and reviewers. We strongly encourage code deposition in a community repository (e.g. GitHub). See the Nature Portfolio guidelines for submitting code & software for further information.

Data

Policy information about availability of data

All manuscripts must include a data availability statement. This statement should provide the following information, where applicable:

- Accession codes, unique identifiers, or web links for publicly available datasets
- A description of any restrictions on data availability
- For clinical datasets or third party data, please ensure that the statement adheres to our policy

Models of the presented complexes have been deposited in the Protein Data Bank under accession code 7ZGJ (ISG65:C3) and 7ZGK (ISG65:C3b). The associated electron density maps have been deposited in the Electron Microscopy Data Bank under accession codes EMD-14707 [https://www.ebi.ac.uk/pdbe/entry/emdb/EMD-14707] (ISG65:C3) and EMD-14708 [https://www.ebi.ac.uk/pdbe/entry/emdb/EMD-14708] (ISG65:C3b). The hybrid model of ISG65 has been deposited in PDBDev under accession code PDBDEV_00000201 [https://pdb-dev.wwpdb.org/entry.html?PDBDEV_00000201]. Starting models of C3b and C3 used in modelling of the complexes are deposited in the Protein Data Bank under accession code 2I07 [http://doi.org/10.2210/pdb2I07/pdb] (C3b) and 2A73 [http://doi.org/10.2210/pdb2A73/pdb] (C3). The SAXS data generated in this study is available in SASBDB under accession codes SASDP99 [https://www.sasbdb.org/data/SASDP99/] (ISG65) and SASDPA9 [https://www.sasbdb.org/data/SASDPA9/] (ISG65:C3d) . The mass spectrometry proteomics data have been deposited to the ProteomeXchange Consortium via the PRIDE partner repository with the dataset identifiers PXD036611 [http://proteomecentral.proteomexchange.org/cgi/GetDataset?ID=PXD036611] (C3 and proteolytic fragments) and PXD033606 [http://proteomecentral.proteomexchange.org/cgi/GetDataset?ID=PXD033606] (Analysis of ISG65 disulfides and HDX-MS).. Flow cytometry standard (FCS) files used in this study are available in the FlowRepository database under accession code FR-FCM-Z5XC [https://flowrepository.org/id/FR-FCM-Z5XC]. Complete Flow cytometry traces are provided in the Supplementary Data 1 file. Peptide mapping and HDX-MS analysis of the ISG65:C3d complex are provided in the Supplementary Data 2 file.
All other source data are provided in the Source Data file.

Human research participants

Policy information about studies involving human research participants and Sex and Gender in Research.

Reporting on sex and gender	n/a, no human research participants were involved in the study.
Population characteristics	n/a, no human research participants were involved in the study.
Recruitment	n/a, no human research participants were involved in the study.
Ethics oversight	n/a, no human research participants were involved in the study.

Note that full information on the approval of the study protocol must also be provided in the manuscript.

Field-specific reporting

Please select the one below that is the best fit for your research. If you are not sure, read the appropriate sections before making your selection.

☒ Life sciences ☐ Behavioural & social sciences ☐ Ecological, evolutionary & environmental sciences

For a reference copy of the document with all sections, see nature.com/documents/nr-reporting-summary-flat.pdf

Life sciences study design

All studies must disclose on these points even when the disclosure is negative.

Sample size	No sample size calculations were performed. For the haemolytic assays it was empirically determined that the sample sizes used, n= 3 (AP50) and n=4 (CH50), produced sufficient signal over the observed variability across the technical replicates (tested by Two-way ANOVA followed by Sidak's multiple comparison test).
Data exclusions	No data were excluded from the analyses.
Replication	For SPR analyses proteins from the same batch were used. Also, a dilution series of analytes was used, allowing for concentration independent measurements and redundancy. For each concentration single measurements were performed, however, the affinity was derived from all concentrations in the experiment to increase confidence in the determined KD.<br><br>For the AP50 and CH50 tests, measurements were carried out at least in triplicates and using test components only from one kit. Erythrocytes were used as fresh as possible and always within the guaranteed shelf life. All attempted replicates were successful.<br><br>All other experiments (immunofluorescence imaging, FACS, SDS PAGE and Western Blots) were performed in three independent replicates. The agreement between independent measurements was sufficiently high to not perform any more measurements as an increase in data quality was not to be expected.
Randomization	Randomization was not relevant to this study as no experiments comparing multiple study groups, and thus potentially involving selection

	bias, were performed.
Blinding	Blinding was not relevant to this study as no experiments posing the risk of observer, participant or confirmation bias were performed.

Reporting for specific materials, systems and methods

We require information from authors about some types of materials, experimental systems and methods used in many studies. Here, indicate whether each material, system or method listed is relevant to your study. If you are not sure if a list item applies to your research, read the appropriate section before selecting a response.

Materials & experimental systems

n/a	Involved in the study	
☐	☒	Antibodies
☐	☒	Eukaryotic cell lines
☒	☐	Palaeontology and archaeology
☒	☐	Animals and other organisms
☒	☐	Clinical data
☒	☐	Dual use research of concern

Methods

n/a	Involved in the study	
☒	☐	ChIP-seq
☐	☒	Flow cytometry
☒	☐	MRI-based neuroimaging

Antibodies

Antibodies used	1. Mouse anti-humanC3a, monoclonal, R&D systems, Clone 354113, Catalog number MAB3677, LOT:YHK0220031<br>2. Goat Anti-mouse HRP conjugate, recombinant, Invitrogen, Catalog number A28177, LOT: RA222815<br>3. Complement C3 Polyclonal Antibody, FITC, Invitrogen, Catalog # PA1-28933, RRID AB_1954681<br>4. Complement Factor Bb Monoclonal Antibody (10-09), Invitrogen, Catalog # MA5-28085, RRID AB_2745075<br>5. Anti-C4 antibody, abcam, Catalog # ab47788<br>6. Complement C5 Polyclonal Antibody, Invitrogen, Catalog # PA5-96933, RRID AB_2808735<br>7. Penta anti-His HRP Antibody, Qiagen, Catalog # 34460
Validation	1. Mouse anti-human C3a detects human Complement Component C3a in direct ELISAs and Western blots. In direct ELISAs and Western blots, no cross-reactivity with recombinant human (rh) Complement Component C5a or rhComplement Component C3d is observed.<br>2. WB,ELISA (secondary Antibody)<br>3. Goat anti-human Complement C3 detects human Complement Component C3 in ICC/IF, Flow Cytometry (10.1016/j.isci.2022.103931)<br>4. Mouse anti-human complement factor Bb detects human Factor Bb in WB, IHC (F), Flow cytometry and ELISA.<br>5. Goat anti-human C4 detects human complement component C4 in ELISA, IHC-P, ICC/IF, WB and IP (10.1084/jem.20092545)<br>6. Rabbit anti-human Complement C5 detects amino acids 1567-1676 of human C5/C5a in WB.<br>7. Penta anti-His HRP Antibody detects internal, N- and C-terminal 6xHis tags in WB & ELISA with negligible cross-reactivity with crude E. coli, yeast mammalian or insect cell lysates.<br><br>For fluorescence microscopy unlabelled antibodies were labelled with Alex Fluor 594 Conjugation kit, ab269822

Eukaryotic cell lines

Policy information about cell lines and Sex and Gender in Research

Cell line source(s)	Trypanosoma brucei gambiense
Authentication	Received as a gift. Authenticated by collaborator Prof Philippe Büscher, Institute of Tropical Medicine, Antwerp, Belgium<br><br>N. Van Reet, P.P. Pyana, S. Deborggraeve, P. Büscher, F. Claes,<br>Trypanosoma brucei gambiense: HMI-9 medium containing methylcellulose and human serum supports the continuous axenic in vitro propagation of the bloodstream form,<br>Experimental Parasitology, Volume 128, Issue 3, 2011, Pages 285-290, ISSN 0014-4894,<br>https://doi.org/10.1016/j.exppara.2011.02.018.
Mycoplasma contamination	not tested
Commonly misidentified lines<br>(See ICLAC register)	n/a

Flow Cytometry

Plots

Confirm that:

- [x] The axis labels state the marker and fluorochrome used (e.g. CD4-FITC).
- [x] The axis scales are clearly visible. Include numbers along axes only for bottom left plot of group (a 'group' is an analysis of identical markers).
- [x] All plots are contour plots with outliers or pseudocolor plots.
- [x] A numerical value for number of cells or percentage (with statistics) is provided.

Methodology

Sample preparation	4x10exp5 T. brucei gambiense cells were harvested from a mid-exponential grown culture and resuspended in 200ul serum-free Hmi-9 supplemented with 1% bovine serum albumin. Uptake assays were carried out with AF594 labelled C3b at a concentration of 50 ug/ml or AF594 labelled human transferrin (T13343, Thermofisher Scientific) as positive control at a concentration of 50 ug/ml, with or without addition of recombinant ISG65 or ISG75 extracellular fragment at a 1.5-fold molar excess (preincubated with C3b for 10 min). Samples were incubated at 37°C and harvested at different timepoints. To analyse the contribution of surface binding, samples were pre-cooled on ice for 10 min before mixing with test proteins and harvested immediately. To demonstrate specific, receptor mediated uptake, we compared C3bAF594 surface binding and uptake at a concentration of 10 ug/ml in the presence and absence of non-labelled C3b at a concentration of 40 ug/ml (adding 80 ug/ml ISG 65 or ISG75, respectively). Cells were harvested by centrifugation (1400 x g, 4°C), washed twice with ice-cold PBS and resuspended in 500 ul PBS. Cells were fixed for 30 min at RT after addition of an equal volume of 4% formaldehyde in PBS, then washed once with PBS and resuspended in a final volume of 300 ul PBS
Instrument	LS Fortessa (BD Biosciences)
Software	BD FACSDiva software v8.0.1
Cell population abundance	approximately 54% of counted particles were consistent with trypanosome cells (singlets), using established gating procedures. Aliquots obtained by cell sorting were inspected microscopically and revealed small particles/cell fragments in the rejected population.
Gating strategy	The gating strategy is shown in great detail in Supplementary Figure 3b,c and Supplementary Data 1. Essentially the intensity cut-off was determined using a negative control.

- [x] Tick this box to confirm that a figure exemplifying the gating strategy is provided in the Supplementary Information.
